# Sustainable-psycho-nutritional intervention programme for a sustainable diet (the ‘NutriSOS’ study) and its effects on eating behaviour, diet quality, nutritional status, physical activity, metabolic biomarkers, gut microbiota and water and carbon footprints in Mexican population: study protocol of an mHealth randomised controlled trial

**DOI:** 10.1017/S0007114523000843

**Published:** 2023-11-28

**Authors:** Mariana Lares-Michel, Fatima Ezzahra Housni, Zyanya Reyes-Castillo, Jesús R. Huertas, Virginia Gabriela Aguilera-Cervantes, Rosa María Michel-Nava

**Affiliations:** 1 Institute of Nutrition and Food Technology ‘José Mataix Verdú’, Biomedical Research Center, University of Granada, Avenida del Conocimiento S/N. Parque Tecnológico de la Salud. Armilla, 18071 Granada, Spain; 2 Instituto de Investigaciones en Comportamiento Alimentario y Nutrición (IICAN), University Center of the South, University of Guadalajara, Av. Enrique Arreola Silva 883, Col. Centro, 49000, Cd. Guzmán, Jalisco, Mexico; 3 Tecnológico Nacional de México, Campus Ciudad Guzmán, Avenida Tecnológico 100, Col. Centro, 49000 Ciudad Guzmán, Mexico

**Keywords:** Sustainable diets, Behaviour change, Eating behaviour, MHealth intervention, Gut microbiota, Diet’s environmental impact, Nutritional education, Environmental education, Mexican population, Water footprint, Carbon footprint

## Abstract

Mexico is going through an environmental and nutritional crisis related to unsustainable dietary behaviours. Sustainable diets could solve both problems together. This study protocol aims to develop a three-stage, 15-week mHealth randomised controlled trial of a sustainable-psycho-nutritional intervention programme to promote Mexican population adherence to a sustainable diet and to evaluate its effects on health and environmental outcomes. In stage 1, the programme will be designed using the sustainable diets, behaviour change wheel and capability, opportunity, motivation, and behaviour (COM-B) models. A sustainable food guide, recipes, meal plans and a mobile application will be developed. In stage 2, the intervention will be implemented for 7 weeks, and a 7-week follow-up period in a young Mexican adults (18–35 years) sample, randomly divided (1:1 ratio) into a control group (*n* 50) and an experimental group (*n* 50), will be divided into two arms at week 8. Outcomes will include health, nutrition, environment, behaviour and nutritional-sustainable knowledge. Additionally, socio-economics and culture will be considered. Thirteen behavioural objectives will be included using successive approaches in online workshops twice a week. The population will be monitored using the mobile application consisting of behavioural change techniques. In stage 3, the effects of the intervention will be assessed using mixed-effects models on dietary intake and quality, nutritional status, physical activity, metabolic biomarkers (serum glucose and lipid profile), gut microbiota composition and dietary water and carbon footprints of the evaluated population. Improvements in health outcomes and a decrease in dietary water and carbon footprints are expected.

The following protocol paper has been written in accordance with the SPIRIT (Standard Protocol Items: Recommendations for Interventional Trials) guidelines^(1)^.

## Introduction

The change from the traditional Mexican diet to a Western diet (i.e. increases in animal and ultra-processed foods intake and decreases in the consumption of fruits, vegetables, whole grains, legumes and nuts), generated by the nutritional transition, has not only propitiated a prevalence of more than 75 % of metabolic alterations (obesity, type 2 diabetes, CVD, dyslipidaemia)^(2–4)^ and in the gut microbiota of the Mexican population^(5)^, but it has also generated the water and carbon footprints of their diet to be among the highest in the world with more than 8000 l per person per day (l p^–1^d^–1^)^(6)^ and 3·9 kg CO_2_eq p^–1^d^–1(7)^, respectively. This is linked to the aggravation of climate change, with increases of more than 1·0°C in the average atmospheric temperatures since the industrial revolution in the year 1760^(8)^ and the current water crisis that Mexico is going through, which affects 85 % of the territory and has been referred to as the worst water drought in history, affecting the water supply of millions of Mexicans^(2,9)^.

One proposal for the joint solution of these problems has been the adoption of sustainable and territorial diets, which in Mexico could be carried out through the recovery of its traditional diet, both pre-Hispanic (milpa diet) and colonised, which, prepared with low-fat culinary techniques and low content of animal products, can be considered as an appropriate option from a nutritional, cultural, economic and environmental perspective, dimensions that must be present for a diet to be considered as sustainable^(2,10–13)^. The traditional Mexican diet consists of a plant-based flexitarian dietary pattern, that is, a diet rich in fruits like papaya, pineapple, and guava, vegetables such as zucchini, tomatoes, nopal (cactus) and chillies, legumes such as beans, whole grains such as maize and healthy fats such as avocado, peanuts and chia; meanwhile, it includes low amounts of dairy products, eggs, poultry, fish and few red meat in special occasions^(11,12,14)^.

This type of diet has been referred to as a low environmental impact dietary pattern that, besides of been accessible to the population and culturally accepted, is linked to the proliferation of butyrate-producing bacteria such as *Akkermansia muciniphila* in the gut microbiota. Also, it has been linked to anti-inflammatory effects, glucose and lipids profile regulation and increased glucose sensitivity, reduced insulin resistance related to acanthosis nigricans and regulated other clinical signs such as blood pressure^(11,15,16)^.

For adopting a particular diet, it is necessary to modify the eating behaviour of the population^(17–19)^. There are multiple strategies to change dietary and unsustainable behaviours in a population, among which intervention programmes based on behaviour change techniques have stood out^(18,20,21)^. However, nationally and internationally, no multidisciplinary intervention programme based on randomised controlled trials (RCT) that aims to promote adequate nutrition to improve health and reduce the environmental impact of dietary behaviours has been reported, considering the population’s cultural, social, economic and psychological aspects.

Based on the above, this study protocol aims to design a three-stage, 15-week intervention programme based on an RCT, following the guide to designing interventions of the behaviour change wheel model^(18)^, which includes both nutritional and sustainability elements, in the sense of considering dietary behaviours of the Mexican population with high environmental impact, and side health effects, in addition to taking into account their social, cultural and economic aspects. Besides to these axes, the basis of behavioural modification intervention programmes is psychology. Therefore, it is proposed to design an RCT of a sustainable-psycho-nutritional intervention programme to promote the population’s adherence to sustainable diets. But, in addition, it is proposed to evaluate its effects on dietary intake and quality, nutritional status, physical activity, metabolic biomarkers (serum glucose and lipid profile), gut microbiota composition and dietary water and carbon footprints in a Mexican population sample. Secondarily, the programme’s effects will be assessed on energy and nutrients intake, clinical aspects (blood pressure, acanthosis nigricans and deficiencies or nutrient excesses) and nutritional-sustainable knowledge. Additionally, socio-economic, cultural and behavioural elements such as motivation, intentions and attitudes will be explored. Because of the effectiveness of technologies used in nutritional^(22,23)^ and environmental^(24,25)^ interventions, the intervention programme will be a digital one, for which a mobile application is being designed to evaluate and monitor the intervened population. Therefore, this will be an mHealth (the use of mobile wireless technologies for public health)^(26)^ intervention programme.

We hypothesised that an RCT of a sustainable-psycho-nutritional intervention digital programme for a sustainable diet could modify the eating behaviour; thus, dietary intake and physical activity of a Mexican population group and guide it towards a sustainable diet, generating increases in diet quality, decreases in the dietary water and carbon footprint’s, glucose levels, total cholesterol, LDL-cholesterol and triglycerides (TG) in the population with levels above recommended and increases in HDL-cholesterol levels in population with low levels. Likewise, we hypothesised that this type of programme could modify the composition of the gut microbiota of the population, promoting the proliferation of bacteria related to metabolic health, and maintain adequate body fat levels and BMI in the population with adequate levels or reduce them in the overweight or obese population. Secondarily, we hypothesised that the programme could maintain blood pressure levels in the healthy population and lower them in people with high blood pressure, reduce the presence of acanthosis nigricans and other deficiencies or nutrient excesses signs and increase the nutritional-sustainable knowledge of the population. Additionally, we hypothesised that the RCT could modify energy and nutrients intake and guide it towards individual sustainable requirements. Besides, it is expected to improve other secondary outcomes such as muscle mass, visceral fat and waist–hip ratio. In an exploratory way, motivation, intentions and attitudes are expected to positively change and orientate eating behaviour towards sustainable diets consumption.

## Methods

It is proposed to carry out a 15-week RCT of a sustainable-psycho-nutritional intervention digital programme for a sustainable diet, with three stages related to each other. According to the study design, it will be an mHealth RCT.

### Stage 1: design of the programme

A sustainable-psycho-nutritional intervention programme for a sustainable diet will be designed based on the characteristics of a sustainable diet for the national context of Mexico, adaptable to any regional context. The intervention will be named ‘NutriSOS: Programa Digital de Intervención Psico-Nutricional-Sostenible para una Dieta Sostenible’, which meaning in English is, ‘NutriSOS: Sustainable-Psycho-Nutritional Digital Intervention Program for a Sustainable Diet’. It will include a mobile application being designed for this project, consisting of a sustainable food guide, sustainable-psycho-nutritional workshops, sustainable recipes, and meal plans, and behavioural change techniques. The programme will be designed based on the sustainable diets model^(13)^ and concept^(10)^, the behaviour change wheel model^(18)^, the capability, opportunity, motivation, and behaviour (COM-B) model^(18)^, the guideline for the development and evaluation of digital behavioural interventions in healthcare^(23)^ and the guide to designing interventions of the behaviour change wheel, which incorporate three stages and eight development steps^(27)^. Stage one will have a year duration (2022–2023).

#### Mobile application design

A mobile application (app) is being developed in collaboration with software and mobile application developers. It will be based on the user-centred methodology^(28)^ and on the guide for the development and evaluation of digital behaviour interventions in the healthcare^(23)^. It will contain the following behaviour change techniques: education and instructions on how to perform a behaviour through workshops and recipes videos^(17,29)^; persuasion by sending messages of risks and benefits (information about health and environmental consequences)^(29,30)^, encouragement and coercion using token economy^(17)^, nudges by messaging^(31)^, self-monitoring by graphical progress viewing^(32,33)^, successive approaches by behavioural objectives addressing^(17)^, social support by a chat and a forum for the participants to share pictures, comments and doubts^(34)^ and guides^(18)^, through a sustainable food guide that will be designed employing linear programming optimisation^(35,36)^ using MATLAB® and Python®, and graphic design programmes such as Canva®, Adobe Illustrator® and BioRender®. This guide will be created based on the recommendations of the FAO for the development of dietary guidelines^(37)^ and the Mexican Food Equivalent System^(38)^. Cut-off points for the token economy system will be established to be charged to the app (online Supplementary Material 1.1). Besides, the mobile application will include an initial clinical history based on sustainability dimensions and will include a section for dietary records and the visualisation of prescribed meal plans by day weeks and meal times, based on the Mexican Equivalent System^(38)^ and pre-quantified menus. This section will include a food list for registering consumptions, which will include food pictures for portions guide. The mobile application database will be charged with a food list and its related nutritional composition data (i.e. energy, macro and micronutrients)^(38,39)^, environmental aspects (i.e. carbon and water footprints), economy data (i.e. food prices) and culture aspects (i.e. the origin of food).

Additionally, educational workshops and meal plans will be designed, following the model of sustainable diets of Johnston *et al.* ^(13)^ and using the Nutriecology® Nutritional-Ecological software and the Nutrimind® Software for diet calculation. Likewise, the guidelines for the prescription of meal plans, menu design and recipes for the Mexican population will be followed^(40)^. Before the mobile application launch, its feasibility, acceptability, quality and usability will be assessed. Also, it will be evaluated following the APEASE criteria, which include evaluating (1) affordability, (2) practicability, (3) effectiveness and cost-effectiveness, (4) acceptability, (5) side effects/safety and (6) equity. For that purpose, a pilot study will be performed in a sample of thirty young adults (18–35 years) that will be recruited at the National Technology of Mexico (TECNM), campus Ciudad Guzmán. Sample size was calculated according to the minimum sample size for pilot studies^(41)^. The APEASE criteria as well as the feasibility, acceptability, quality and usability of the app will be assessed in a validation survey (online Supplementary Material 1.2) designed for this study based on other validated instruments^(42–46)^. The questionnaire will be applied to the sample once they use the mobile application for a period of 2 months.

#### Behaviour change intervention design process

The process of design of the NutriSOS study is shown in Table 1. The process of understanding the behaviour, including the problem definition^(2–4,6,47)^, the selected target behaviours and what needs to be modified from them^(13,17,32,48–50)^, is described. Also, the chosen intervention functions^(51)^ and the selected policy categories from the behaviour change wheel model are presented.

**Table 1.** Design of the randomised controlled trial of a sustainable-psycho-nutritional digital intervention programme according to the behaviour change wheel model and guide for interventions design

Stage 1: Understand the behaviour	
Step 1: Define the problem in behavioural terms	This step will be based on the available scientific evidence on the country’s dietary and environmental situation, which reveals a high prevalence of overweight, obesity and associated pathologies such as type 2 diabetes, dyslipidaemia and hypertension^(2,3)^. Also, high values of dietary water and carbon footprints were found^(2,7)^. These problems are mainly related to inadequate food consumption (which will be further detailed) and lack of physical activity^(2)^
Step 2: Select target behaviour	The target behaviours will be lack of physical activity performance, inadequate consumption of Mexican foods and dishes, fruits and vegetables, whole grains, legumes, seeds and healthy fats, dairy products, eggs, fish and shellfish, chicken, red meat (beef and pork, and, in some cases, goat and lamb) and processed, ultra-processed foods and added and free sugars, as well as foods high in trans and saturated fats
Step 3: Specify the target behaviour	
Step 4: Identify what needs to change	Target behaviours will be established as a goal-setting strategy, which has been reported to be one of the most effective methods for modifying dietary behaviours^(32)^. Also, they will be addressed as successive approximations towards a sustainable diet, another effective behaviour change techniques^(17,18,40)^. The identified target behaviours will be common for all the recruited subjects that will be selected based on certain food consumption habits, frequency of physical activity and other parameters which are detailed in online Supplementary Material Table SM1.5.2. Targets behaviours will be: 1. Increase physical activity 2. Increase the consumption of sustainable Mexican foods and dishes 3. Increase the consumption of fruits and vegetables 4. Increase the consumption of whole grains 5. Increase the consumption of legumes 6. Increase the intake of seeds and healthy fats 7. Reduce dairy consumption 8. Reduce the consumption of eggs 9. Reduce consumption of fish and shellfish 10. Reduce chicken consumption 11. Reduce the consumption of red (beef, pork, goat and lamb) and processed meats 12. Reduce the consumption of ultra-processed foods 13. Reduce the intake of added and free sugars, as well as foods with a high content of trans and saturated fats Although objectives will be common for the participants, into each one, individual advice will be provided according to personal requirements and preferences. For example, in all subjects fruit consumption will be promoted, but for those not consuming papaya, will be direct to increase its consumption, meanwhile those already eating it will be direct to increase the consumption of other fruit, such as pineapple, for example. All foods whose consumption will be promoted will be aligned with the sustainability characteristics for the Mexican context^(12,13)^, the recommendations of a healthy diet provided by the WHO^(50)^ and the model QC7G for food and nutrition education^(48)^, which will specify which foods to select, in which quantities, when to consumed them and how to prepare them. In addition to when to perform physical activity, specifying type and duration^(17)^

Stage 2: Identify intervention options	
Step 5: Intervention functions	The intervention functions from the behaviour change wheel model will be education, persuasion, incentivisation and coercion. These will be verified in relation to the APEASE criterion, Michie *et al.* ^(51)^
Step 6: Policy categories	The policy category of the behaviour change wheel model will be the guidelines. Therefore, a nutritional-sustainable food guide and personalised meal plans, according to individual requirements, preferences and contexts, will be designed, using linear programming optimisation^(35,36)^, based on the FAO recommendations for the development of dietary guidelines^(37)^, and covering the elements of the sustainable diets model^(13)^ adapted to this study (Fig. 1), and according to primary (P), secondary (S) and exploratory (E) outcomes and descriptive (D) variables as follows: 1) Health and Nutrition: 1.1) Food and nutrient needs, food security, accessibility: P: diet quality, and food needs; S: nutrients and energy requirements. 1.2) Well-being and Health: P: BMI and body fat levels, serum glucose and lipids profile, gut microbiota, and physical activity; S: body weight, visceral fat, muscle mass, waist and hip circumferences, as well as waist–hip ratio, blood pressure, acanthosis nigricans and signs of nutrient deficiencies; D: height, and clinical history. 2) Environment: 2.1) Biodiversity, environment and climate: P: dietary carbon footprint. 2.2) “Eco-friendly”, local, and seasonal foods: P: dietary water footprint; D: locally produced and seasonal foods. 3) Economy: 3.1) Equity and fair trade: D: food prices, socio-economic and socio-demographic level of the population. 4) Culture and society: 4.1) Cultural heritage and skills: D: Traditional Mexican diet recipes and foods. 5) Psychology: S: nutritional-sustainable knowledge; E: behavioural elements from the behaviour change wheel and COM-B models, D: adherence to the intervention Subsequently, this nutritional-sustainable food guide will be accompanied by a system of equivalent foods^(38)^ that, in addition to incorporating the food’s nutrients, will integrate their environmental impact by dietary water footprint^(6)^, and carbon footprint^(7,52)^, food prices and cultural characteristics of the main consumed foods in Mexico^(6,12)^ Once sustainability dimensions are calculated, a sustainable recipe book will be developed, including examples of meal plans with individualisation options. These meal plans will be made up of a distribution of equivalent food rations^(38)^ and examples of menus that meet the nutritional and sustainability characteristics of the Mexican population, with adaptation options. The menus and preparation processes will be uploaded to the mobile application

Stage 3: Identify content and implementation options	
Step 7: Behaviour change techniques	Specific behaviour change techniques will be used covering the selected intervention functions as follows: • Education: successive approximations in the workshops addressing the target objectives (instructions on how to perform a behaviour) • Persuasion: nudges, risk and benefits communication (information about health and environmental consequences) • Incentivisation, coercion and monitoring: messaging, remainders and token economy through dietary records by writing and photos on the mobile application • Social support: forum in the mobile app where participants can share pictures of their foods and their physical activity performance. Also, will be able to like the photos of other participants and comment on the photos. Also, a chat for the resolution of doubts between user and administrator will be included in the mobile application
Step 8: Mode of delivery	The mode of delivery will be digital since a mobile application is being designed and the workshops will be delivered by digital platforms in videos^(23)^

**Fig. 1.**
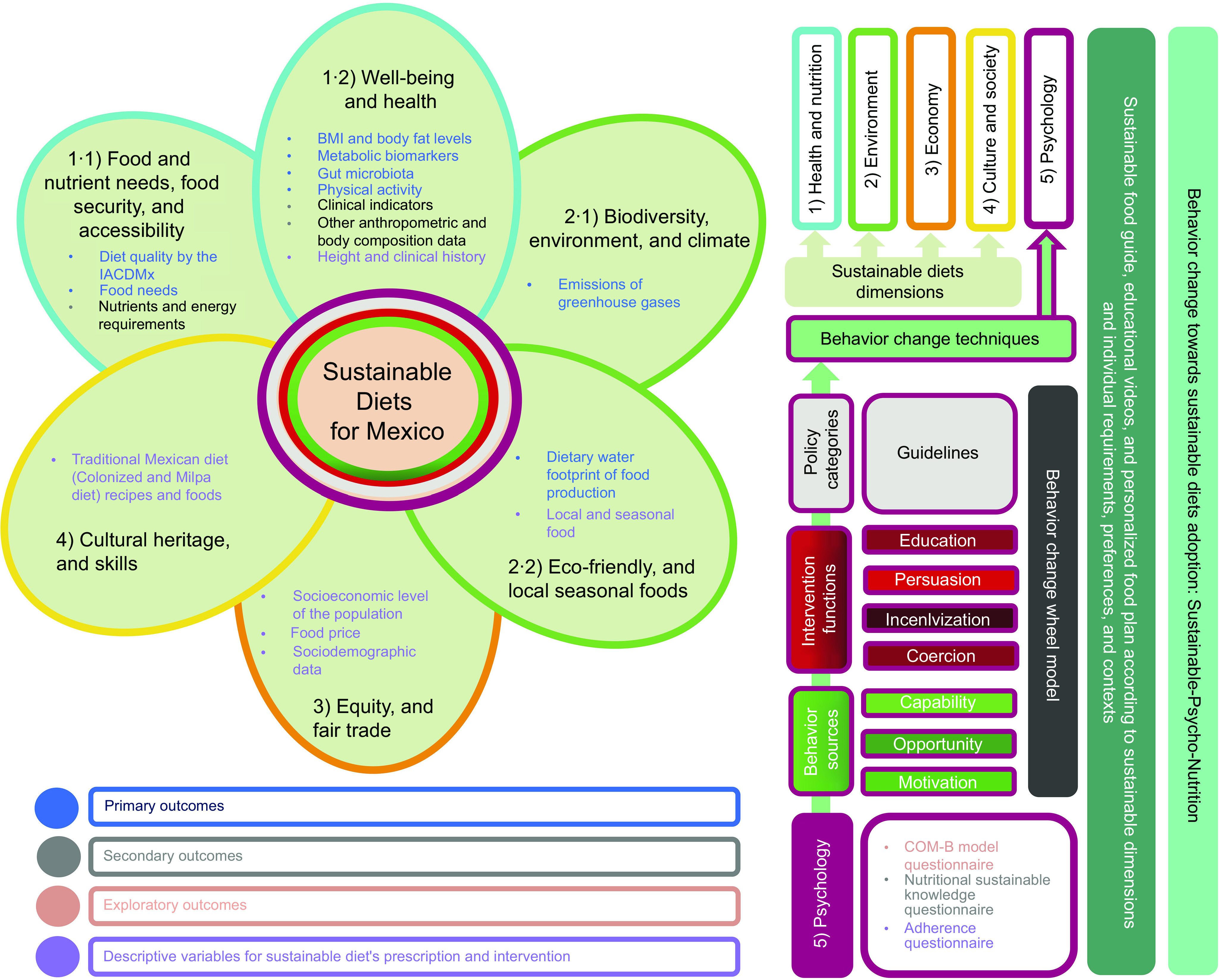
Sustainable-psycho-nutritional approach for the mHealth randomised controlled trial intervention design, based on the sustainable diets model^(13)^, the behaviour change wheel model^(18)^, the COM-B model^(18)^ and the guide for the development and evaluation of digital behaviour interventions in healthcare^(23)^. Own elaboration.

Figure 1 presents a graphical representation of the sustainability aspects considered in the intervention programme, considering all elements of sustainable diets^(13)^. Also, the behavioural approach to be followed is presented according to the behaviour change wheel model^(18,51)^, and all primary, secondary and exploratory outcomes are presented, as well as the descriptive variables of the study (online Supplementary Material 1.3).

### Stage 2: implementation of the mHealth randomised controlled trial of a sustainable-psycho-nutritional intervention programme

#### Study design

The NutriSOS study is a 15-week three-stage mHealth RCT with three points of evaluation: baseline (week 0), monitoring (week 8) and final assessment (week 15). The study will include two parallel groups: a control and an experimental; last will be randomly split into two sub-groups on half of the intervention (week 8). Constant monitoring will be provided based on a mobile application designed for this study. Into NutriSOS, a pilot study is planned to be performed between October 2022 and January 2023. Recruitment is planned to be done during February 2023, and the intervention will be from February to May 2023. The study was designed based on recommendations for group designs in behaviour modification programmes^(53)^. Due to the nature of the study, blinding subjects will not be possible. However, the researcher that will perform the randomised process will be blinded. Also, participant’s numbers will be coded into the databases for analysis to blind the statistical analysis process when possible.

#### Participants, sampling and recruitment

Once the intervention programme has been designed, it will be implemented in a sample of young Mexican adults from the south of Jalisco that will be randomly divided. Since no RCT targeting sustainability outcomes has been previously reported, sample size calculation, based on mean primary outcomes change, is challenging. Therefore, we based on a previous RCT of a multicomponent behavioural intervention to reduce meat consumption including health, nutrition and environmental sustainability elements^(54,55)^. The authors calculated the sample size based on pragmatic considerations due to the lack of research studies directly comparable to theirs. In their study, an initial minimum sample of 100 volunteers was considered to achieve a medium effect size of d = 0·6 with a power of 1-beta = 0·84 and a two-tailed *α* criterion of 0·05^(54)^.

To rectify statistical power according to our primary outcomes, we launched the formula of Sakpal^(56)^ for comparing two means, considering a minimal clinically important difference of 0·5 for each primary outcome. Online Supplementary Material 1.4 shows primary outcomes and the data used for the formula calculation. Other RCT based on plant-based diets^(57,58)^ or meat intake reduction^(55)^ were consulted to verify the minimal clinically important difference and standard deviations, which were among the selected ranges. Studies regarding carbon footprint^(59)^, water footprint^(4)^ and gut microbiota^(60,61)^ were also consulted. Finally, the following formula for calculating sample size was used, considering a dropout rate of 10 %^(56)^:

(1)

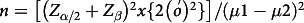




where:

*n* = sample size required in each group

μ1 = mean change in primary outcomes of other similar studies = 5

μ2 = mean change in primary outcomes = 4·5

μ1–μ2 = significant difference = 0·5

ó = standard deviation = 1·195

Z_*α*/2_:1·96 (based on a 5 % of level of significance)

Z_*β*_: 0·84 (based on 80 % of power).

Based on the above formula, the sample size required per group is 45. Hence, total sample size required is 90. Considering a dropout rate of 10 %, total sample size required is 100, which agrees with the RCT of a multicomponent behavioural intervention that was initially considered as the base for sample size estimation in this RCT^(54,55)^. The total sample will be randomly divided into two groups: a control group (*n* 50) and an experimental group (*n* 50), which will be split into two arms for a pilot study at week 8, having at final, three pilot groups. Randomisation will be performed based on a computer-generated randomisation sequence, produced by an independent statistician^(53)^, assigning participants in a 1:1 ratio to an intervention group or a control group.

An initial screening self-responded questionnaire to identify the inclusion and exclusion criteria (online Supplementary Material 1.5, 4, and Figure SM1.5.3) will be digitally shared through social networks, WhatsApp groups and posters at strategic points, such as Universities, gyms and the downtown city. Promotional posters of the NutriSOS study will be posted on social media and strategically at the selected points (online Supplementary Material Figures SM1.5.1 and SM1.5.2). It is expected to initially screen at least 200 subjects, achieving a final sample of 100 participants. After screening, eligible participants will be contacted by email or phone.

We will include a population between 18 and 35 years old, with BMI values from 18·5 to 40, with or without risk factors for developing chronic diseases but without diagnosis with previous pharmacological treatment. The inclusion, exclusion and elimination criteria are detailed in online Supplementary Material Table SM1.5.1. Also, for being included in the study, cut-off points to identify inadequate consumption and physical activity were established (online Supplementary Material Table SM1.5.2). Young adults were selected for this study since they are already considered adults who make autonomous food decisions. Still, at the same time, their young age makes them susceptible to behavioural modification. In addition, they represent parents or future parents, as well as the active population of Mexico, so providing nutritional-sustainable education to these people could generate long-term benefits for their health and that of their families^(25,62,63)^.

#### Procedure

Participants will be evaluated following the nutritional care process model^(64)^. The first evaluation will be performed in both groups in baseline at the Behavioural Feeding and Nutrition Research Institute (IICAN) in Ciudad Guzmán, Jalisco. A certified nutritionist in behavioural science, sustainable nutrition and anthropometry (ISAK level 2) will perform all assessments, following the validated techniques of Suverza and Haua^(65)^, which are the base for nutritional assessment in Mexico, and with the supervision of a multidisciplinary research team conformed by an environmental specialist, two biomedical specialists, a psychologist and a specialist in education and software development. Trained technicians, nutritionists and biological systems engineers will support data collection and analysis, under the supervision of the certified nutritionist and the research team.

Once evaluated, participants will be randomly divided into the experimental and control group. The control group will be invited to continue participating in the study to receive ‘free sustainable nutritional diagnosis’, but they will not be provided with nutritional plans, neither sustainable nutritional videos. They will not also have access to the mobile application. At the end of the study, they will receive free sustainable nutritional recommendations as thanks for their participation. Experimental and control groups will always be cited in different nutritional offices and schedules at IICAN.

For the experimental group, the established behavioural objectives will be individually adapted according to personal requirements. For example, for the objective of increasing fruits and vegetables consumption, a list of fruits and vegetables is going to be established, and according to the initial dietary assessment, specific fruits and vegetables are going to be prescribed in the meal plan of each participant, that is, fruit salad recipe with papaya and pineapple for persons not consuming those, and with blackberries and guava for those already consuming pineapple and papaya, but not those new fruits. The behavioural objectives (Table 1) will be addressed for 7 weeks, considering two objectives per week, thus providing two weekly educational workshops. Also, personalised meal plans will be prescribed according to the linear programming optimisation^(35,36)^ performed at MATLAB® and Python®.

The monitoring of the mobile app’s usability will be done by accessing the app’s database, where all the information of the participants will be saved. All information will be exported in excel sheets, which facilitates the process of monitoring regarding the number of times the participants enter the app, the number of dietary records entered to the app, the number of interactions in the forum and the message sent.

**Fig. 2.**
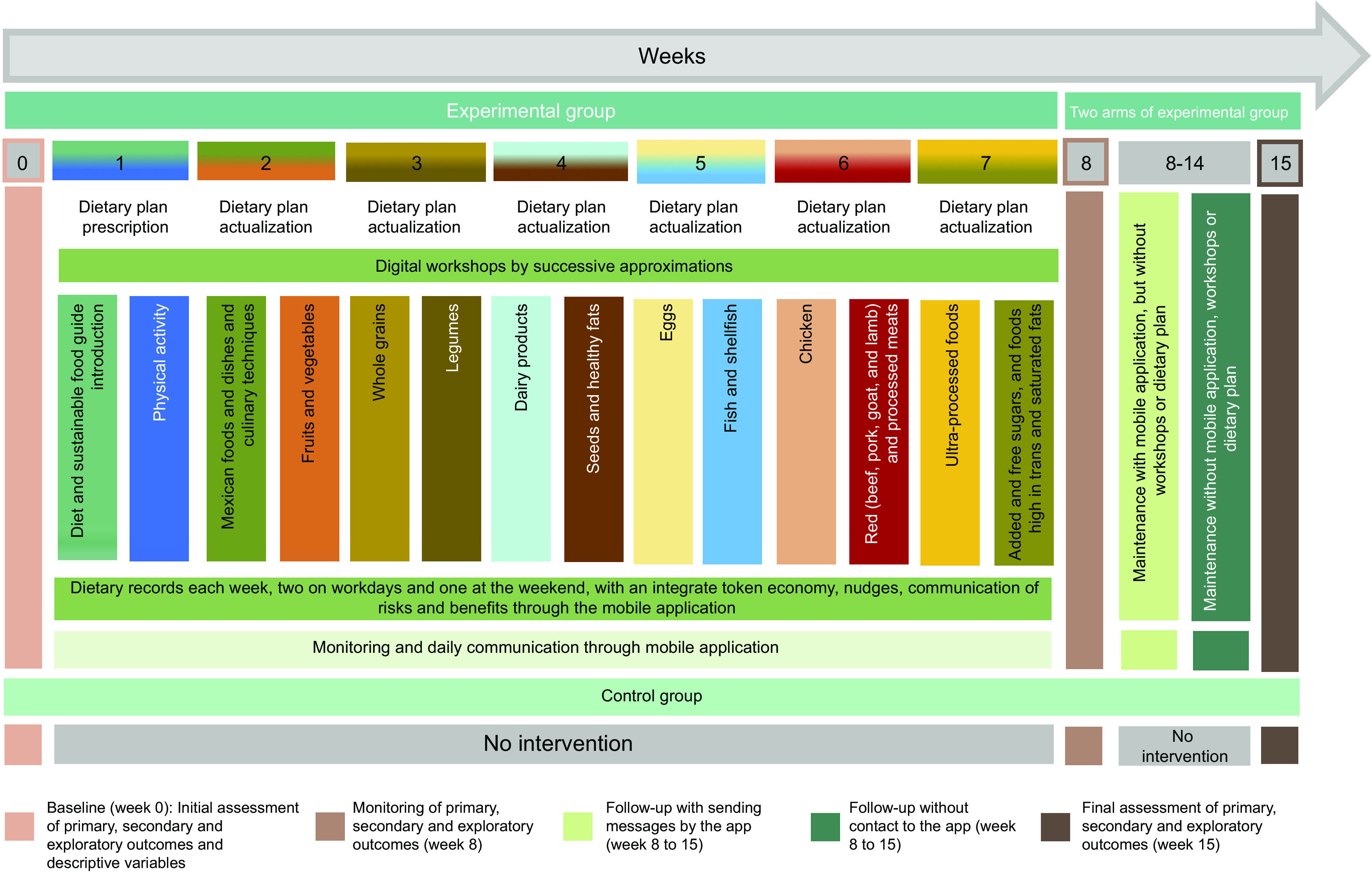
Study design for stage 2. Own elaboration.

After 7 weeks of educational and nutritional intervention, at week 8, a monitoring assessment will be carried out, and a pilot study with only the experimental population will be performed. For that reason, the experimental group will be divided into two sub-groups, where one group will stop being intervened completely (*n* 25), and another will continue to receive messages through the mobile application but will no longer receive educational workshops or meal plans (*n* 25) for 8 weeks. With this, sub-sample > 12 subjects is maintained as suggested by Julious *et al.* ^(41)^ for pilot studies. Finally, at week 15, a final evaluation will be carried out. The study design is shown in Fig. 2.

#### Sustainable-psycho-nutritional outcomes and descriptive variables

The NutriSOS study will include a set of primary, secondary and exploratory outcomes according to the dimensions of sustainable diets, which are presented in Fig. 1 and detailed in online Supplementary Material Table SM1.3.1., SM1.3.2. and SM1.3.3. Additionally, descriptive variables will be included to prescribe sustainable diets properly (online Supplementary Material Table SM1.3.4.). As can be observed, blue font indicates primary outcomes, grey font secondary outcomes, light pink exploratory outcomes and purple descriptive variables. Light blue in the contour lines of the sustainable diet’s model indicates relationships between health and nutrition dimensions^(13)^. Green in the contour lines of the sustainable diet’s model refers to the environment, orange contour line to the economy, yellow contour line to culture and society and magenta contour line to psychological aspects, besides being included in the sustainable diet’s model^(13)^, are related to the behaviour change wheel and COM-B models, including behaviour change techniques^(18)^.

The study outcomes and descriptive variables will be included in a complete clinical history that will be uploaded to the mobile application (online Supplementary Material 1.6). Although it is recommended to include the fewest possible number of outcomes in an RCT^(66)^, sustainable diets comprise several variables in each of their dimensions (nutrition and health, environment, economics, culture and society), for which in this study, the most relevant based on available evidence were selected. Both primary and secondary outcomes, exploratory outcomes and descriptive variables will be evaluated by the certified nutritionists with the supervision of the multidisciplinary research team and with the support of trained technicians, nutritionists and biological systems engineers. Evaluations will be performed following the nutritional care process model, thus identifying the problem of the population, establishing the aetiology and stating the signs and symptoms^(64)^.

##### Primary outcomes (P)

Primary outcomes are fully described in online Supplementary Material Table SM1.3.1 and are presented according to the dimensions shown in Fig. 1. Primary outcomes were selected because of their relevance for sustainable diets, and human and environmental health^(8,10,13)^.

P.1) Health and Nutrition

P.1.1) Food and nutrient needs, food security and accessibility (dietetics)

P.1.1.1) Change from baseline diet quality at weeks 8 and 15. Diet quality was chosen as a primary outcome among dietetical outcomes since it evaluates that the diet satisfies all nutritional needs regarding energy intake, Fe, Ca, fibre and water consumption and also considers the diet to be balanced regarding proteins, lipids and carbohydrates. Additionally, it evaluates the consumption of food groups and sub-groups in recommended amounts and assesses the intake of SFA, PUFA, Na, alcoholic beverages, sugar and Mexican and Western dishes and foods. For the assessment of diet quality, an adaptation of the validated Mexican Diet Quality Index (ICDMx) will be used^(67)^, which is the Alternate Mexican Diet Quality Index (IACDMx). This is automatically calculated in the Nutriecology® software^(68)^ by responding a validated adapted FFQ (online Supplementary Material Table SM1.6.1)^(6,69)^, which will be administered to each participant by the nutritionist of the study (not self-reported). Food replicas, pictures, cups and spoons will be used as reference for portions intake estimations.

P.1.1.2) Change from baseline dietary intake at weeks 8 and 15. The consumption of the food groups considered in the behavioural objectives will also be considered primary outcomes since they are considered key elements for achieving a sustainable diet^(8,10)^. Those include the intake of Mexican foods and dishes, fruits and vegetables, whole grains, legumes, dairy products, seeds and healthy fats, eggs, fish and seafood, chicken, beef, pork, goat, lamb and processed meats, ultra-processed foods, and added and free sugars, and trans and saturated fats. Their consumption will be evaluated through a 24-h recall administrated by the nutritionist in the Nutriecology® software^(68)^, which provides automatic nutritional composition calculations. Tools for portion estimations will also be used (i.e. cups, food pictures). Dietary records will also be used for food groups intake assessment, which the subjects will load to the mobile application each week, at least twice a weekdays and one in weekends (Fig. 2, online Supplementary Material Table SM1.6.1)^(6,69,70)^.

P.1.2) Well-being and health

P.1.2.1) Anthropometric and body composition data

P.1.2.1.1) Change from baseline BMI at weeks 8 and 15, calculated from weight/height^2^ (weight will be parallel taken with body fat and height will be taken from descriptive variables that will be explained later). P.1.2.1.2) Change from baseline body fat percentage at weeks 8 and 15. Although the intervention will not be directed to lose weight (i.e. it will be an isoenergetic dietary prescription), increases of fruits, vegetables, whole grains and legumes consumption and reduces in animal products and ultra-processed foods intake have been related to decreases in BMI and body fat even under *ad libitum* conditions in plan-based diets^(58,71)^. Weight and fat levels will be measured with a bioimpedance scale TANITA BC-601 FITSCAN® with the patients without shoes, with light clothes and in a minimum of 2 h fasting. Women will be asked not to be during their menstrual periods to avoid hydration alterations that can alter bioimpedance results. Also, metallic objects will be removed from the participants during the measures.

P.1.2.2) Biochemical data (biomarkers related to dietary interventions)

P.1.2.2.1) Change from baseline glucose levels at weeks 8 and 15; P.1.2.2.2) Change from baseline LDL-cholesterol levels at weeks 8 and 15; P.1.2.2.3) Change from baseline HDL-cholesterol levels at weeks 8 and 15; P.1.2.2.4) Change from baseline total cholesterol levels at weeks 8 and 15 and P.1.2.2.5) Change from baseline TG levels at weeks 8 and 15. Improvements in both glucose and lipid profiles (TG, LDL, HDL and total cholesterol) have been reported in nutritional interventions^(58,71)^. Biomarkers will be analysed by colorimetric enzymatic methods using the Spinreact® S. A/S A. U (Girona, Spain) laboratory kits for fasting glucose determination (Cat. No. 1001190), total cholesterol (Cat. No. 41022), LDL-cholesterol (Cat. No. BSIS51-E), HDL-cholesterol (Cat. No. BSIS37-E) and TG (Cat. No. 1001313)^(72)^. The reference points for glucose values and lipid profiles will be taken from the regulations in force in Mexico^(73,74)^. Blood samples will be taken by a nurse from the antecubital vein of each participant, following the protocol from the Mexican Health Secretary^(72,75)^. After an overnight fast of 8–12 h, samples will be taken by specialists and will be centrifuged at 3500 rpm for 15 min to separate the serum. Samples will be aliquoted and stored at –20°C until the day of testing. Tourniquets, alcohol swabs, 10 ml syringes and blood collection tubes will be required.

P.1.2.2.6) Change from baseline gut microbiota at weeks 8 and 15. The bacteria related to plant-based healthy diets consumption such as *Lactobacillus* ^(15,76,77)^, *Bifidobacterium* ^(15,76,77)^, *Prevotella copri* ^(78,79)^ and *Faecalibacterium prausnitzii* ^(71,80–83)^ were selected as primary outcomes, as well as *Akkermansia muciniphila*, which is associated with the Mexican pre-Hispanic diet intake^(15,78,80,82,84)^. Those bacteria are also associated with anti-inflammatory effects^(15,78,80,82,84)^ and glucose modulation, especially *Prevotella copri* ^(78,79)^. *Bilophila wadsworthia* ^(81,85)^ and *Streptococcus thermophilus* ^(81,86)^ will also be considered as primary outcomes since the first is related to dairy products, meat and Westernised diets consumption^(76,87)^, and the second to dairy intake^(81,86)^. Firmicutes, Bacteroidetes^(5,76,88)^ and *Clostridium coccoides* ^(89)^ relative abundance will also be considered primary outcomes as Firmicutes and Bacteroidetes are the main bacteria present in the intestine, and the three bacteria are related to obesity^(89)^ and a high fat intake^(90)^. The relative abundance of each bacterium will be determined employing the real-time quantitative PCR (qPCR) method^(5,89)^. Analysis will be performed in faecal samples that will be collected in a sterile stool container using a clean kit, including gloves and a spatula to be delivered to the participants. Once collected, the samples will be divided into aliquots and stored at –80°C, following a method used for the Mexican population^(5,89)^.

To analyse the gut microbiota, first, DNA will be extracted from the collected stool samples, following the protocol for rapid purification of genomic DNA from stool samples. The Qiagen brand commercial kit (1066790ES, USA) will be used. This procedure integrates two stages: lysis and separation of impurities from stool samples, for which Inhibitex Buffer will be used, and DNA purification will be carried out using centrifugation columns. Once the bacterial DNA samples are obtained, they will be stored and labelled in sterile plastic microtubes (Eppendorf 1·6 ml) at a temperature of −80°C until further analysis. Next, the purity of the DNA will be verified, and its concentration will be determined using a NanoDrop Lite spectrophotometer (Thermo Scientific). One microlitre of the DNA stock of each sample will be placed on the lens of the equipment, and the sample will be read at a wavelength of 260 nm for DNA quantification and 280 nm for protein quantification. The purity will be determined by calculating the index performed by the team by dividing the reading at 260 nm by the reading at 280 nm and will be considered acceptable in a range of 1·5 to 2. The concentration of the purified coproDNA sample will be measured by their absorbance ratio of 260/280 nm using the same spectrophotometer. This analysis corresponds to the absorbance index of nucleic acids and provides the final concentration in ng/µl^(5,89,91)^.

Once the previous analyses have been completed, the identification of the gut microbiota will be carried out using the qPCR molecular technique on the StepOne Applied BioSystems platform, using the SYBR Green reagent as DNA detection chemistry. This reagent is an agent that intercalates into the DNA double helix and fluoresces as the DNA copies are synthesised. Therefore, the equipment will record a higher fluorescence signal at a higher concentration of the DNA of interest (bacterial). In this case, the interest analysis will be carried out in the V3-V4 hypervariable region of the bacterial 16S rRNA gene. Specific primers, detailed in online Supplementary Material 1.7, will be used for this analysis and correspond to the selected bacteria as primary outcomes. The exact procedure to follow regarding the qPCR run and the specific conditions and temperatures of the analysis are presented in the investigation of Rodríguez-Lara *et al.* ^(89)^, which will serve as the guide for this research project.

P.1.2.3) Physical activity

P.1.2.3.1) Change from baseline physical activity levels at weeks 8 and 15. This outcome was selected as primary since it is part of the behavioural objectives of the intervention, and the increases in physical activity levels are associated with better health outcomes in dietary interventions^(43,92,93)^. Physical activity levels will be assessed through specific questions about physical activity type, frequency, intensity and duration using the IPAQ Scoring Protocol^(94)^. The related questions are included in the clinical history shown in online Supplementary Material 1.6.

P.2) Environment

P.2.1) Biodiversity, environment and climate

P.2.1.1) Change from baseline dietary carbon footprint at weeks 8 and 15. Carbon footprint was selected as primary outcome, since it is one of the environmental indications most related to climate change and sustainable diets^(8,59)^. It will be calculated using the Life Cycle Assessment method^(7,95,96)^. Specifically, the SHARP-Indicators Database will be used^(52)^, which was recently used for the estimation of the greenhouse gas emissions generated by the Mexican diet^(7)^. Also, the data provided there consider the production, trade and transport of foods. Besides, the calculations are adjusted for consumption amount using conversions factors for production, edible portion, cooking losses and gains, and food losses and waste^(52)^. Calculations will be performed in the Nutriecology® Software^(68)^.

P.2.2) Eco-friendly, local seasonal foods

P.2.2.1) Change from baseline dietary water footprint (total, green, blue and grey) at weeks 8 and 15. Water footprint was selected as primary outcome since it is related to water scarcity problems and sustainable diets^(2,6,13)^. It will be calculated using the Water Footprint Assessment method^(97)^ in its version for Mexico’s context^(6)^. The water footprint of each food and ingredient will be calculated by applying correction factors to convert cooked to uncooked foods and peeled to unpeeled foods. Also, water involved in cooking and food washing will be evaluated. Calculations will be performed using the Nutriecology® Software^(68)^.

#### Secondary outcomes (S)

Secondary outcomes are fully described in online Supplementary Material Table SM1.3.2 and are presented according to the dimensions shown in Fig. 1. Secondary outcomes were selected because of their relevance for sustainable diets prescription and because they are necessary to complement primary outcomes^(8,10,13)^.

S.1) Health and Nutrition

S.1.1) Food and nutrient needs, food security and accessibility (dietetics)

S.1.1.1) Change from baseline energy and nutrient intake at weeks 8 and 15. In addition to evaluating diet quality as a total score, the specific consumption of energy, macro and micronutrients (online Supplementary Material Table SM1.3.2) will be considered as secondary variables since they help to analyse individual dietary adequacy in relationship with the consumption of a sustainable diet^(8,10,59)^. Energy and nutrient intake will be calculated based on the FFQ^(6,69)^, the 24-h recalls and the dietary records (online Supplementary Material Table SM1.6.1)^(6,69,70)^ already applied to assess diet quality and dietary intake. Nutrient calculation will be performed using the Nutriecology® software^(68)^.

S.1.2) Well-being and health

S.1.2.1) Clinical data

S.1.2.1.1) Change from baseline systolic and diastolic blood pressure at weeks 8 and 15, assessed with a Medstar® sphygmomanometer following the Mexican normativity^(98)^; S.1.2.1.2) Change from baseline acanthosis nigricans at weeks 8 and 15 (as a clinical sign of insulin resistance), evaluated by physical exploration, searching for hyperpigmentation and thickening of the skin with velvety in visible flex areas (axilla, anterior ulnar area, and posterior and lateral region of the neck)^(99)^. S.1.2.1.3) Change from signs of nutrient deficiencies or excess at weeks 8 and 15, assessed by clinical exploration regarding hair, nails, mouth, tongue, oedema and mucous membranes appearance^(65)^.

S.1.2.2) Anthropometric and body composition data

S.1.2.2.1) Change from baseline muscle mass at weeks 8 and 15; S.1.2.2.2) Change from baseline visceral fat at weeks 8 and 15; S.1.2.2.3) Change from baseline weight at weeks 8 and 15. All body composition data will be assessed using a bioimpedance scale TANITA BC-601 FITSCAN® following the protocol mentioned above; S.1.2.2.4) Change from baseline waist circumference at weeks 8 and 15, evaluated with a Lufkin® metal tape measure, in the midpoint between the lower rib and the iliac crest, at the end of normal expiration^(4)^; S.1.2.2.5) Change from baseline hips circumference at weeks 8 and 15, evaluated with a Lufkin® metal tape measure, at the most prominent part of hips.

S.5) Psychology (behavioural aspects)

S.5.1) Change from baseline nutritional-sustainable knowledge at weeks 8 and 15, evaluated through a designed questionnaire (online Supplementary Material 1.8) based on the psychological capacity presented in the COM-B model. It includes a series of questions for evaluating nutritional-sustainable knowledge related to the established behavioural objectives. It was designed based on the revised version of the General Nutrition Knowledge Questionnaire, considering scores for each question^(100)^. Its development also considered the recommendations provided in the manual for designing nutritional knowledge questionnaires of the Food and Agriculture Organisation of the United Nations (FAO)^(101)^. Besides, it includes questions on sustainable food consumption^(102)^.

#### Exploratory outcomes (E)

In order to identify changes in motivation, intentions, attitudes, perceived behavioural control and adherence, and subjective social norms of eating a sustainable diet an exploratory analysis will be performed based on the behavioural elements of the COM-B model^(18,54)^. Those elements will also be considered as descriptive variables at baseline for the prescription of sustainable diets (online Supplementary Material Table SM1.3.3).

E.5) Psychology (behavioural aspects)

E.5.1) Psychological aspects from the COM-B model will be evaluated by a structured questionnaire. All dimensions of the COM-B model will be assessed in a designed questionnaire based on Brain *et al.* ^(103)^ (online Supplementary Material 1.9). The questions are related to physical and psychological ability, automatic and reflexive motivation, and physical and social opportunity^(18)^. Questions were adapted according to the context of sustainable diets^(13)^: (1) capacity: knowledge of sustainable nutrition, preparation skills food and preparation capacity; (2) opportunity: time to eat and prepare food, access to food and food storage; (3) motivation: the desire to change eating habits, emotions involved in food consumption and habits that participants are willing to change.

#### Descriptive variables (D)

The study also considered descriptive variables to include all sustainability dimensions in participants assessment and dietary prescription (online Supplementary Material Table SM1.3.4).

D.1) Health and nutrition

D.1.2) Well-being and health

D.1.2.2) Anthropometric and body composition data

D.1.2.2.1) Baseline height will be evaluated with a Smartmet® stadiometer according to the Frankfurt plane with the participant barefoot^(65)^. D.1.2.2.2) A baseline clinical history will be applied to prescribe dietetic plans properly. The clinical history will cover pathologies suffering, pathological family history, sun exposure, sleep habits, medicaments and supplement use. Other factors influencing gut microbiota will also be included, such as hygiene aspects, delivery type and lactation when baby. Additionally, question regarding food preparation, food shopping places, food preferences, allergies and intolerances, and about following specific diets at the moment of the evaluation will be assessed by a questionnaire (online Supplementary Material 1.6).

D.2) Environment

D.2.2) Eco-friendly, local seasonal foods

D.2.2.1) Local and seasonal food

D.2.2.1.1) Identification of produce region origin of the recommended foods in the intervention. Field work in local flea markets and supermarkets will be carried out for that purpose. Also, the databases of supermarkets and official governmental webpages of food produced in Mexico’s regions will be consulted^(104)^.

D.3) Economy

D.3.1) Equity and fair trade

D.3.1.1) The socio-economic baseline level of the population will be evaluated to provide affordable diets according to their economic possibilities. Socio-economic level will be assessed by educational and occupational levels, and economic income according to classifications used for Mexico, which are detailed in online Supplementary Material 1.6 ^(105)^ D.3.1.2) Food prices will be used for the design of the optimised dietary food guide and meal plans, to ensure affordable diets in the participants. The prices of the most consumed foods in Mexico, reported in a recent exploratory study of the Mexican’s diet^(6)^, will be investigated in fieldwork in local flea markets and supermarkets. Besides, the databases of supermarkets will be consulted, as well as the food prices database of the National Institute of Statistics and Geography (INEGI)^(106)^. D.3.1.3) Socio-demographic data will include sex, age, country of origin, residence city, civil status and religion.

D.4) Culture and society

D.4.1) Cultural heritage and skills

D.4.1.1) Food culture related to celebrations and traditions will be investigated in a literature review^(11,12)^. According to the Traditional Mexican diet (colonised and Milpa diet), recipes will be designed to instruct participants about food preparation. Also, both the dietary guideline and meal plans will be developed according to the traditional Mexican diet, and diet plans will be prescribed according to personal preferences, needs and contexts.

### Stage 3: effects of the randomised controlled trial of a sustainable-psycho-nutritional digital intervention programme

As the last stage of this protocol, the corresponding laboratory analyses and statistical tests will be carried out. This stage will be 6 months long and is planned to be done from March to September 2023. Each of the primary, secondary and exploratory outcomes will be analysed according to the ‘Sustainable-psycho-nutritional outcomes and descriptive variables’ section.

#### Evaluation of adherence to the programme

To measure the intervention population’s adherence to the programme and as a descriptive psychological final variable (Fig. 1), an adapted questionnaire will be applied based on that of Gila-Díaz *et al.* ^(107)^, which will consist of the contrast of the recommendations provided with the performance of the behaviours to promote. For example, questions about the type and intensity of physical activity performed and the amount and frequency of consumption of each food included in the behavioural objectives will be included. This questionnaire is shown in online Supplementary Material 1.10.

#### Statistical analyses

The statistical analysis of the intervention will be first performed using an intention-to-treat analysis, that is, analysing the effect of assigning a treatment to a group of patients, in this case, an RCT of a sustainable-psycho-nutritional digital intervention. In that case, the advantages of randomisation are maintained as well as statistical power. However, since dietary interventions are susceptible to have low adherence from the patients^(107)^, a secondary analysis will be performed according to the per-protocol analysis. A per-protocol analysis refers to analysing the effect of successfully following a treatment and not only to be assigned to a treatment (i.e. excluding participants with low adherence to the RCT of a sustainable-psycho-nutritional digital intervention). Therefore, if results obtained from an intention-to-treat analysis and a per-protocol analysis lead to similar conclusions, a robust interpretation can be obtained and it can be proved that the treatment effectively works^(108)^.

Therefore, the following analysis will be performed twice, according to the intention-to-treat and per-protocol analysis. Online Supplementary Material 1.11. shows the statistical analysis to perform according to outcomes, variable types and analytical objectives of the study. The distribution of the data will be analysed with the Kolmogorov Smirnov test. Descriptive analysis will include means, standard deviations, medians, minimum and maximum values. Next, the programme’s effects on the selected outcomes will be assessed by comparing them between evaluations (intra-subject) and between groups according to data distribution. First, the primary and secondary outcomes detailed in online Supplementary Material Table SM1.11 and corresponding to numeric and continuous variables (i.e. plasma glucose levels (mg/dl), dietary water footprint (litres/person), will be compared intra-subject at baseline, week 8 and week 15 in each group (control, experimental and two arms experimental) using a test of ANOVA with a Bonferroni post hoc test, for data normally distributed, or in case of founding non-normal data, using a Friedman test with a Tukey post hoc analysis.

For comparing primary and secondary outcomes corresponding to nominal and ordinal categorical variables (i.e. presence or absence of signs of nutrients deficiencies or excess and level of adherence to the programme, respectively) intra-subjects, the chi-squared test will be used. Following, primary and secondary outcomes considered as numeric and continuous variables will be compared between groups (control, experimental and two arms experimental), at weeks 0, 8 and 15. For data normally distributed, a student’s *t* test for independent samples will be used, and for non-normal data, the Mann–Whitney U test will be performed. The chi-squared test will also compare nominal and ordinal categorical variables between groups. To explore the relationships between the participant’s adherence to the intervention and primary and secondary outcomes detailed in online Supplementary Material Table SM1.11, we plan to construct mixed-effects linear regression models with the level of adherence to the intervention as a categorical independent variable and the primary and secondary outcomes detail in online Supplementary Material Table SM1.11 as continuous numeric dependent variables. According to the results to be obtained, different random or fixed effects could be launched in regard to the outcomes. Also, the adjustment of the data for energy intake and/or sex will be considered, depending on the obtained data. With those analyses it is expected to explore the changes to primary and secondary outcomes that would occur if the experimental group had a high adherence to the intervention. Additional mixed-effects linear regression models will be constructed with the exploratory outcomes according to the results.

Finally, to identify risks and protective factors between the participant’s adherence to the intervention and primary and secondary outcomes (online Supplementary Material Table SM1.11), binary logistic regressions reporting OR will be launched, considering only the control group and the experimental group, and converting all variable to dichotomic according to established cut-off points. For biochemical outcomes such as plasma glucose, TG, total, HDL and LDL-cholesterol levels (mg/dl) the official regulation norms in force in Mexico will be used as a reference^(73,74)^. In the case of gut microbiota, since no established cut-off points exist, we will use median values of the relative abundance of the identified bacteria to establish cut-off points under and over those median values.

The dietary water footprint cut-off points will be established based on the cut-off point of a healthy diet water footprint that is equal to 2714 litres per person per day (l p^–1^d^–1^) only from green and blue water footprint^(4)^. Regarding dietary carbon footprint, the select cut-off point will be a mathematically optimised sustainable diet with a carbon footprint of 2·43 kg CO_2_eq/d^(59)^.

Regarding anthropometric and body composition outcomes, individual cut-off points will be established. For example, for body weight, theoretical weight according to height will be used for everyone, classifying it as ‘under’ or ‘over’. The manual of Suverza and Haua^(65)^ will be used for that classification. Regarding BMI, individuals with values equal to or above 25 will be considered as cases, and people with a BMI below 25, as controls. Adults with a BMI ≤ 18·5–24·9 will be considered into the control group^(109)^. Cases of high body fat percentage will be determined to be > 22 % in men and > 32 % in women^(4)^. Values under those specified will be considered controls^(110)^. A waist circumference above 80 cm in women and 90 cm in men will be used to determine cases when these figures are equal or over, and controls when subjects have a waist circumference less than those indicated above^(4,111)^. Cases and controls regarding visceral fat will be determined with the manuals of the bioimpedance scale TANITA BC-601 FITSCAN®^(112)^, considering levels above or below 12, respectively. Those manuals will also be used for muscle mass, which are specific to men and women and are automatically reported as high, average or low, for which high and average will be defined as controls and low as cases. Based on waist and hips circumferences, the waist–hip ratio will be calculated by dividing waist circumference by hip circumference. Cut-off points will be 0·85 for women and 0·90 for men^(113)^.

Systolic and diastolic blood pressure will be categorised according to the cut-off points established in the Mexican normativity (120/80 mmHg)^(98)^. For energy intake, the individual requirement will be calculated using the Harris and Benedict formula^(40)^ and the basal levels of physical activity obtained in the first assessment of the intervention. For macro and micronutrient intake, the current Mexican tables of reference will be used^(114)^, which are specific to sex and age ranges. Included macronutrients are carbohydrates, fibre, sugar, protein, lipids, SFA, MUFA, PUFA and cholesterol, which cut-off points are detailed in online Supplementary Material Table SM1.11. Micronutrients include Ca, P, Fe, Mg, Na, K, Zn, Se, vitamin A, ascorbic acid, thiamine, riboflavin, niacin, pyridoxine, folic acid and cobalamin. Additionally, ethanol consumption will be considered. Online Supplementary Material Table SM1.11. presents the cut-off points of each variable. For the food groups corresponding to the behavioural objectives of the intervention (i.e. Mexican foods and dishes, fruits and vegetables, whole grains, etc.), the cut-off points previously established in online Supplementary Material Table SM1.5.2 will be used. Statistical analysis will be performed at the STATA V12® programme.

### Ethical and biosafety considerations

This project has already been evaluated and approved by the Ethics Committee of the Center for Studies and Research in Behavior of the University Center for Biological and Agricultural Sciences (CUCBA) from the University of Guadalajara with the number CUCBA/CEIC/CE/002/2022 and by the Technical Research Committee of the University Center of the South (CUSUR), with the number 2021D001. This protocol is registered on Clinical Trials.gov with the ID: NCT05457439. When performing the intervention, the Declaration of Helsinki^(115)^ and biosafety protocols of the Secretary of Health of Mexico^(116)^ will be followed all the time. All participants will sign an informed consent (online Supplementary Material 1.12), and their identities will be protected by the Federal Law on Protection of Personal Data Held by Private Parties^(117)^.

Due to regulations in research with human beings and the COVID-19 pandemic, a strict biosafety protocol will be followed, where mouth covers will always be worn, both by the evaluating staff and by the participants. Likewise, the sample collection personnel will wear protective glasses and sterile gloves, and the work area will constantly be disinfected. In addition, hazardous biological waste will be disposed of in a particular trash can, according to the Ministry of Health recommendations^(118)^.

### Results dissemination

Any substantial change to the protocol and a result report will be submitted to the ethics committee and to Clinical Trials.gov. The results of this RCT will be reported according to the Consolidated Standards of Reporting Trials guidelines^(119)^ and will be submitted for publication to scientific journals, regardless of the outcome. The project results will also be presented at national and international congresses and discussion forums.

## Discussion

By developing this programme, we would be generating the first bases for the Mexican population (and future populations) to achieve a healthy and sustainable diet that can have positive effects on health outcomes and decrease the environmental impact of food consumption, thus addressing two of the main problems afflicting the world population: nutritional and environmental issues. Also, we are proposing a new concept: the sustainable-psycho-nutrition, which is presented as an approach based on behavioural science that integrates the psychological, social, cultural, economic, health, nutritional and environmental aspects of eating behaviour, whose objective is to generate the necessary bases to carry out behavioural change interventions and to guide the eating behaviour of the population, towards sustainable eating behaviours. Within this term, we also launched the nutritional-ecological education, whose objective is to get people to acquire and perform the appropriate behavioural repertoire to determine what, how much, when and how to eat in relation to when, how much and how energy is spent on maintaining or recovering their physical well-being, considering at all times the environmental impact of their behaviours and selecting the most sustainable foods, regarding the environment, culture, economy, preferences, food security, health, nutrition, among other factors^(17)^.

Besides those aspects, the mobile application we are currently developing will be a tool that will facilitate the promotion of sustainable diets first at the national level in Mexico and forward worldwide. Also, we will generate the first sustainable food guide for Mexico’s context that will consider both sustainability and psychological and behavioural aspects. Moreover, the workshops, recipes and meal plans we are creating will serve as tools for the country’s health and environmental sector to promote sustainable diet consumption. Finally, the link between gut microbiota and sustainable diets is a new aspect that this study will characterise for the first time. Because of the growing evidence around gut microbiota and health, an intervention targeting gut microbiota and sustainability aspects could ensure in an easily way human and planetary wellness^(120)^. To conclude, this project intends to bring attention to the importance of considering behavioural interventions and techniques for promoting sustainable diets.

## Supplementary material

For supplementary material accompanying this paper visit https://doi.org/10.1017/S0007114523000843. click here to view supplementary material
