# Supplementary material for: Sustainable-psycho-nutritional intervention programme for a sustainable diet (the ‘NutriSOS’ study) and its effects on eating behaviour, diet quality, nutritional status, physical activity, metabolic biomarkers, gut microbiota and water and carbon footprints in Mexican population: study protocol of an mHealth randomised controlled trial
Source: Br J Nutr. 2023 Mar 30;130(10):1823–38. doi: 10.1017/S0007114523000843 (PMC10587386; doi:10.1017/S0007114523000843)
Supplement: Supplementary file 1 [file S0007114523000843sup.zip › S0007114523000843sup001.docx]

**Online Supplementary Material 1**

**Sustainable-psycho-nutritional intervention program for a sustainable diet (the ‘NutriSOS’ study) and its effects on eating behavior, diet quality, nutritional status, physical activity, metabolic biomarkers, gut microbiota, and water and carbon footprints in Mexican population: study protocol of an mHealth randomized controlled trial**

Mariana Lares-Michel^1,2^*, Fatima Ezzahra Housni^1^, Zyanya Reyes-Castillo^1^, Jesús R. Huertas^2^, Virginia Gabriela Aguilera Cervantes^1^, and Rosa María Michel Nava^3^

1. Instituto de Investigaciones en Comportamiento Alimentario y Nutrición (IICAN), University Center of the South, University of Guadalajara. Address: Av. Enrique Arreola Silva 883, Col. Centro. Zip Code: 49000, Cd. Guzmán, Jalisco, México.

2. Institute of Nutrition and Food Technology “José Mataix Verdú”, Biomedical Research Center, University of Granada, Avenida del Conocimiento S/N. Parque Tecnológico de la Salud. Armilla,18071 Granada, Spain

3. Tecnológico Nacional de México, Campus Ciudad Guzmán. Address: Avenida Tecnológico 100, Col. Centro. Zip Code: 49000. Ciudad Guzmán, México.

***Corresponding author**

Name: Mariana Lares Michel

Current Address: Av. Enrique Arreola Silva 883, Col. Centro. C.P. 49000. Cd. Guzmán, Jalisco. México.

Phone: (+52) 3411017629

E-mail: [marianalares@correo.ugr.es](mailto:marianalares@correo.ugr.es)

ClinicalTrials.gov ID: NCT05457439

Brief Title: Sustainable-psycho-nutritional digital intervention program and its effects on health outcomes and the environment

**Online Supplementary Material 1.1 Token economy system cut-off points**

Table SM1.1.1 Scores according to the frequency of physical activity according to the type of activity

| Physical activity | Frequency for obtaining points | Score |
| --- | --- | --- |
| Cardiovascular exercises (any including walking, jogging, running, aerobics, Zumba or dance, cycling, swimming)  or  Strength or resistance exercises (any including Crossfit, weights, multifunctional  focused exercises (for example, sit-ups, squats, push-ups) | Once a week | 1 |
|  | 2 times a week | 2 |
|  | 3 times a week | 3 |
|  | 4 times a week | 4 |
|  | 5 times a week | 5 |
|  | 6 times a week | 6 |
|  | 7 times a week | 7 |
|  | < once a week | 0 |
| Combination of cardiovascular and strength exercises | Once a week | 1 |
|  | 2 times a week | 2 |
|  | 3 times a week | 3 |
|  | 4 times a week | 4 |
|  | 5 times a week | 5 |
|  | 6 times a week | 6 |
|  | 7 times a week | 7 |
|  | < once a week | 0 |

Table SM1.1.2. Scores according to duration and intensity according to the type of physical activity

| Type | Duration | Intensity | Score |
| --- | --- | --- | --- |
| Cardiovascular exercises (any including walking, jogging, running, aerobics, Zumba or dance, cycling, swimming)  or  Strength or resistance exercises (any including Crossfit, weights, multifunctional  focused exercises (for example, sit-ups, squats, push-ups) | Less than 10 minutes | Mild | 0.5 |
|  | 10 to 29 minutes | Mild | 1 |
|  | 30 to 59 minutes | Mild | 2 |
|  | 60 to 120 minutes | Mild | 3 |
|  | More than 120 minutes, but less than 240 minutes | Mild | 4 |
|  | More than 240 minutes | Mild | 4 points and send a message not to exceed the amount of exercise |
|  | Less than 10 minutes | Moderate | 1 |
|  | 10 to 29 minutes | Moderate | 2 |
|  | 30 to 59 minutes | Moderate | 4 |
|  | 60 to 120 minutes | Moderate | 6 |
|  | More than 120 minutes, but less than 240 minutes | Moderate | 8 |
|  | More than 240 minutes | Moderate | 8 points and send a message not to exceed the amount of exercise |
|  | Less than 10 minutes | Intense | 2 |
|  | 10 to 29 minutes | Intense | 4 |
|  | 30 to 59 minutes | Intense | 8 |
|  | 60 to 120 minutes | Intense | 12 |
|  | More than 120 minutes, but less than 240 minutes | Intense | 16 |
|  | More than 240 minutes | Intense | 16 points and send a message not to exceed the amount of exercise |
| Combination of cardio and strength exercises | Less than 10 minutes of each | Mild | 1 |
|  | 10 to 29 minutes of each | Mild | 2 |
|  | 30 to 59 minutes of each | Mild | 4 |
|  | 60 to 120 minutes of each | Mild | 6 |
|  | More than 120 minutes, but less than 240 minutes of each | Mild | 8 |
|  | More than 240 minutes of each | Mild | 8 points and send a message not to exceed the amount of exercise |
|  | Less than 10 minutes of each | Moderate | 2 |
|  | 10 to 29 minutes of each | Moderate | 4 |
|  | 30 to 59 minutes of each | Moderate | 6 |
|  | 60 to 120 minutes of each | Moderate | 8 |
|  | More than 120 minutes, but less than 240 minutes of each | Moderate | 10 |
|  | More than 240 minutes of each | Moderate | 8 points and send a message not to exceed the amount of exercise |
|  | Less than 10 minutes of each | Intense | 4 |
|  | 10 to 29 minutes of each | Intense | 8 |
|  | 30 to 59 minutes of each | Intense | 16 |
|  | 60 to 120 minutes of each | Intense | 24 |
|  | More than 120 minutes, but less than 240 minutes of each | Intense | 32 |
|  | More than 240 minutes of each | Intense | 32 points and send a message not to exceed the amount of exercise |

Table SM1.1.3. Scores according to the frequency of food consumption by groups

| Food group | Points for consumption frequency | Score |
| --- | --- | --- |
| Mexican food and dishes | Once a week | 1 |
|  | 2 times a week | 2 |
|  | 3 times a week | 3 |
|  | 4 times a week | 4 |
|  | 5 times a week | 5 |
|  | 6 times a week | 6 |
|  | 7 times a week | 7 |
|  | < once a week | 0 |
| Mexican desserts and drinks | Once a week | 1 |
|  | 2 times a week | 0 |
|  | 3 times a week | 0 |
|  | 4 times a week | 0 |
|  | 5 times a week | 0 |
|  | 6 times a week | 0 |
|  | 7 times a week | 0 |
| Fruits, vegetables, legumes, dairy, seeds and healthy fats, eggs | Once a week | 1 |
|  | 2 times a week | 2 |
|  | 3 times a week | 3 |
|  | 4 times a week | 4 |
|  | 5 times a week | 5 |
|  | 6 times a week | 6 |
|  | 7 times a week | 7 |
|  | < once a week | 0 |
| Fish and shellfish, chicken and other white meats | Once a week | 3 |
|  | 2 times a week | 2 |
|  | 3 times a week | 1 |
|  | 4 times a week | 0 |
|  | 5 times a week | 0 |
|  | 6 times a week | 0 |
|  | 7 times a week | 0 |
| Red and processed meats, Ultra-processed foods, Foods high in sugars, trans and saturated fats | 0 times a week | 7 |
|  | Once a week | 1 |
|  | 2 times a week | 0 |
|  | 3 times a week | 0 |
|  | 4 times a week | 0 |
|  | 5 times a week | 0 |
|  | 6 times a week | 0 |
|  | 7 times a week | 0 |

Table SM1.1.4. Scores by frequency of consumption

| Food group | Consumption quantities | Score |
| --- | --- | --- |
| Mexican foods and dishes, fruits, vegetables, legumes, dairy products, seeds and healthy fats, eggs | < recommended portion or > minimum portion | 1 |
|  | Recommended portion | 2 |
|  | < recommended maximum or > recommended portion | 1 |
|  | > the recommended maximum or < the recommended minimum | 0 |
| Fish and shellfish, chicken and other white meats, Red and processed meats, Ultra-processed foods, Foods high in sugars, trans and saturated fats | Minimum portion | 3 |
|  | < Recommended portion > minimum portion | 2 |
|  | > recommended portion < maximum portion | 1 |
|  | > Maximum portion | -1 |

Note: recommend amount of consumption will be determined by the linear optimization programing. An example is shown in Table SM1.1.5.

Table SM1.1.5. Examples of recommended amounts of consumption by food groups, including the target foods of the intervention

|  |  |  | Examples of amounts of consumption | | |
| --- | --- | --- | --- | --- | --- |
| Consumption | Group by objective | Food | Average Recommended Serving | Rang**e** | |
|  |  |  |  | Minimum | Maximum |
| Daily (at least 1 food from the group) | Mexican food and dishes | Chilaquiles with or without egg and/or chicken | 1 cup (160 g) | 80 g | 240 g |
|  |  | Mexican style egg (tomato, onion, chili, nopal) | 1 plate (220 g) | 110 g | 330 g |
|  |  | Quesadilla of corn tortilla (without meat) | 1 piece (60 g) | 30 g | 90 g |
|  |  | Beans taco | 1 piece (60 g) | 30 g | 90 g |
|  |  | Panela cheese and beans torta (not meat) | 1 piece (197 g) | 98.5 | 295.5 g |
|  |  | Chicken tamal (no red meat) | 1 piece (200 g) | 100 g | 300 g |
|  |  | Tamal of ash, chard, or cheese (without red meat) | 1 piece (200 g) | 100 g | 300 g |
|  |  | Sweet tamal (corn, fruits) | 1 piece (172 g) | 86 g | 258 g |
|  |  | Mole with chicken | 1 plate (334 g) | 167 g | 501 g |
|  |  | Chicken or vegetarian (mushroom) pozole | 1 plate (300 g) | 150 g | 450 g |
|  |  | Chicken or cheese flute (no red meat) | 1 piece (75 g) | 37.5 g | 112.5 g |
|  |  | Bean or chicken sope (no red meat) | 1 piece (75 g) | 37.5 g | 112.5 g |
|  |  | Chicken or cheese enchilada (no red meat) | 1 piece (75 g) | 196.5 g | 589.5 g |
|  |  | Fish or chicken taco (no beef or pork) | 1 piece (75 g) | 37.5 g | 112.5 g |
|  |  | Bean burrito (no meat) | 1 piece (100 g) | 50 g | 150 g |
|  |  | Fresh water without sugar | 1 cup (240 ml) | 120 ml | 360 ml |
|  |  | Toast with beans or chicken | 1 big toast (174 g) | 87 g | 261 g |
| 1 time per week |  | Rice pudding | ½ cup (178 g) | 89 g | 267 g |
|  |  | Flan | 1 piece (120 ml) | 60 ml | 180 ml |
|  |  | Capirotada | 1 slice (60 g) | 30 g | 90 g |
|  |  | Sweet bread | 1 piece (63 g) | 31.5 g | 94.5 g |
|  |  | Atole in water | 1 cup (240 ml) | 120 ml | 360 ml |
|  |  | Atole in milk or champurrado | 1 cup (240 ml) | 120 ml | 360 ml |
|  |  | Tejuino | 2 cups (418 ml) | 209 ml | 627 ml |
|  |  | Tuba | 1 cup (240 ml) | 120 ml | 360 ml |
|  |  | Tepache or honey water | 1 cup (240 ml) | 120 ml | 360 ml |

Note: this table will be updated according to the linear optimization programming.

Table SM1.1.5. Continuation: Examples of recommended amounts of consumption by food groups, including the target foods of the intervention

|  |  |  | Examples of amounts of consumption | | |
| --- | --- | --- | --- | --- | --- |
| Consumption | Group by objective | Food | Average Recommended Serving | Rang**e** | |
| Daily (at least 1 food from the group) | Fruits | Papaya | 1 cup (140 g) | 70 g | 210 g |
|  |  | Tuna | 1 piece (70 g) | 35 g | 105 g |
|  |  | Pineapple | 1 cup (165 g) | 82.5 g | 247.5 g |
|  |  | Soursop | ½ piece (175 g) | 87.5 g | 262.5 g |
|  |  | Guava | 2 pieces (83 g) | 41.5 g | 124.5 g |
|  |  | Capulín | 1 cup (32 g) | 16 g | 48 g |
|  |  | Pitaya | 1 piece (50 g) | 25 g | 75 g |
|  |  | Nance | 5 pieces (15 g) | 7.5 g | 22.5 g |
|  |  | Creole plum | 2 pieces (105 g) | 52.5 g | 157.5 g |
| Daily (at least 1 food from the group) | Vegetables | Zucchini | 1 piece (91 g) | 45.5 g | 136.5 g |
|  |  | Pumpkin | ½ cup (110 g) | 55 g | 165 g |
|  |  | Onion | ½ cup (58 g) | 29 g | 87 g |
|  |  | Mushrooms | 1 cup (93 g) | 46.5 g | 139.5 g |
|  |  | Chayote | ½ cup (80 g) | 40 g | 120 g |
|  |  | Spicy chili | 1 cup (30 g) | 15 g | 45 g |
|  |  | Huitlacoche | 1/3 cup (66 g) | 33 g | 99 g |
|  |  | Tomato | 2 pieces (124 g) | 62 g | 186 g |
|  |  | Nopales | 2 pieces (134 g) | 67 g | 201 g |
|  |  | Bell pepper | 1 piece (64 g) | 32 g | 96 g |
|  |  | Poblano chili | 1/2 piece (32 g) | 16 g | 48 g |
|  |  | Quelites | ½ cup (66 g) | 33 g | 99 g |
|  |  | Green tomato | 5 pieces (86 g) | 43 g | 129 g |
|  |  | Purslane | 1 cup (115 g) | 57.5 g | 172.5 g |
| Daily (at least 1 food from the group) | Whole grains | Corn tortilla | 1 piece (30 g) | 15 g | 45 g |
|  |  | Baked corn toast | 2 pieces (20 g) | 10 g | 30 g |
|  |  | Corn dough | 1 ball (45 g) | 22.5 g | 67.5 g |
|  |  | Unsweetened amaranth | ¼ cup (16 g) | 8 g | 24 g |
|  |  | Sweetened amaranth | ¼ cup (16 g) | 8 g | 24 g |
|  |  | Sweet potato | 1/3 cup (69 g) | 34.5 g | 103.5 g |

Note: this table will be updated according to the linear optimization programming.

Table SM1.1.5. Continuation: Examples of recommended amounts of consumption by food groups, including the target foods of the intervention

|  |  |  | Examples of amounts of consumption | | |
| --- | --- | --- | --- | --- | --- |
| Consumption | Group by objective | Food | Average Recommended Serving | Rang**e** | |
| Daily (at least 1 food from the group) | Legumes | Pot beans | ½ cup (86 g) | 43 g | 129 g |
|  |  | Fried beans | 1/3 cup (75 g) | 37.5 g | 112.5 g |
|  |  | Cooked lentils | ½ cup (99 g) | 49.5 g | 148.5 g |
|  |  | Cooked chickpeas | ½ cup (82 g) | 41 g | 123 g |
|  |  | Baked beans | ½ cup (85 g) | 42.5 g | 127.5 g |
| Daily (at least 1 food from the group) | Dairy products | Curd | 3 tablespoons (36 g) | 18 g | 54 g |
|  |  | Fresh cheese | 1 slice (40 g) | 20 g | 60 g |
|  |  | Panela cheese | 1 slice (40 g) | 20 g | 60 g |
|  |  | Natural yogurt | 1 cup (227 g) | 113.5 g | 340.5 |
|  |  | Whole milk | 1 cup (240 ml) | 120 ml | 360 ml |
|  |  | Semi-skimmed milk | 1 cup (240 ml) | 120 ml | 360 ml |
|  |  | Skim milk (light) | 1 cup (240 ml) | 120 ml | 360 ml |
| Daily (at least 1 food from the group) | Seeds and healthy fats | Avocado | 1/3 piece (31 g) | 15.5 g | 46.5 g |
|  |  | Dried beans | ¼ cup (38 g) | 19 g | 57 g |
|  |  | Pumpkin seeds | 2 tablespoons (15 g) | 7.5 g | 22.5 g |
|  |  | Chia seeds | 5 teaspoons (12 g) | 6 g | 18 g |
|  |  | Peanuts | 14 pieces (12 g) | 6 g | 18 g |
|  |  | Extra virgin olive oil | 1 teaspoon (5 g) | 5 ml | 15 ml |
| Daily (at least 1 food from the group) | Eggs | Complete chicken egg | 1 piece (44 g) | 22 g | 66 g |
|  |  | Egg white | 2 pieces (66 g) | 33 g | 99 g |
| 0 to 3 times a week | Fish and shellfish | White fish | 1 portion (40 g) | 0 g | 60 g |
|  |  | Octopus | 1 portion (25 g) | 0 g | 37.5 g |
|  |  | River prawn | 4 pieces (110 g) | 0 g | 165 g |
| 0 to 3 times a week | Chicken and other white meat | Grilled chicken | 1 portion (29 g) | 0 g | 58 g |
|  |  | Chicken in piece | 1/3 piece (29 g) | 0 g | 58 g |
|  |  | Insects (maguey worms, crickets) | 1 portion (35 g) | 0 g | 52.5 g |

Note: this table will be updated according to the linear optimization programming.

Table SM1.1.5. Continuation: Examples of recommended amounts of consumption by food groups, including the target foods of the intervention

|  |  |  | Examples of amounts of consumption | | |
| --- | --- | --- | --- | --- | --- |
| Consumption | Group by objective | Food | Average Recommended Serving | Rang**e** | |
| 0 to 1 time a week | Red and processed meats | Beef | 1 portion (14 g) | 0 g | 60 g |
|  |  | Pork Meat | 1 portion (14 g) | 0 g | 60 g |
|  |  | Rabbit | 1 portion (29 g) | 0 g | 58 g |
|  |  | Pork or turkey ham | 1 slice (10.5 g) | 0 g | 21 g |
|  |  | Pork or turkey sausage | 1 slice (14 g) | 0 g | 60 g |
|  |  | Longaniza, chorizo, pepperoni or salami | 1 portion (14 g) | 0 g | 30 g |
|  |  | Beef or pork tacos | 1 piece (75 g) | 0 g | 112.5 g |
|  |  | Quesadilla with meat, gringa or synchronized (with sausages or meats) | 1 piece (60 g) | 0 g | 90 g |
|  |  | Enchilada with beef or pork | 1 piece (75 g) | 0 g | 112.5 g |
|  |  | Flute with beef or pork | 1 piece (75 g) | 0 g | 112.5 g |
|  |  | Sope with beef or pork | 1 piece (75 g) | 0 g | 112.5 g |
|  |  | Pork pozole | 1 plate (300 g) | 0 g | 450 g |
|  |  | Offal | 1 plate (300 g) | 0 g | 450 g |
|  |  | Birria | 1 plate (200 g) |  |  |
|  |  | Meat tamal | 1 piece (200 g) | 0 g | 300 g |
|  |  | Torta (marinated, meat, drowned) | 1 piece (197 g) | 0 g | 295.5 g |
|  |  | Beef burrito | 1 piece (100 g) | 0 g | 150 g |
|  |  | Toast with beef or pork | 1 large piece of toast (174 g) | 0 g | 261 g |
| 0 to 1 time a week | Ultra-processed foods | Soft drink with sugar | ¼ can (92 ml) | 0 ml | 138 ml |
|  |  | Sugar free soft drink | ¼ can (92 ml) | 0 ml | 138 ml |
|  |  | Packaged juice | 1/3 cup (80 ml) | 0 ml | 120 ml |
|  |  | Packaged cookies | 1 piece (10.5 g) | 0 g | 15.75 g |
|  |  | Box cereals | ½ cup (16 g) | 0 g | 24 g |
|  |  | Instant soup | 1 piece (64 g) | 0 g | 96 g |
|  |  | Packaged cupcakes (pingüinos, chocorroles, gansitos) | 1/2 piece (25 g) | 0 g | 37.5 g |
|  |  | Packaged potato chips or snacks | 6 piece (18 g) | 0 g | 27 g |

Note: this table will be updated according to the linear optimization programming.

Table SM1.1.5. Continuation: Examples of recommended amounts of consumption by food groups including the target foods of the intervention

|  |  |  | Examples of amounts of consumption | | |
| --- | --- | --- | --- | --- | --- |
| Consumption | Group by objective | Food | Average Recommended Serving | Rang**e** | |
| 0 to 1 time a week | Foods high in added and free sugars, trans and saturated fats | Table sugar to sweeten | 1 teaspoon (4 g) | 0 g | 6 g |
|  |  | Fresh water with sugar | 1 cup (240 ml) | 0 g | 360 ml |
|  |  | Industrialized jams | 1 tablespoon (17 g) | 0 g | 25.5 g |
|  |  | Industrialized syrups and honeys | 1 teaspoon (7 g) | 0 g | 10.5 g |
|  |  | Cake or pie (thin slice) | 1 slice (28 g) | 0 g | 32 g |
|  |  | Vegetable oil (not olive or canola) | 1 teaspoon (5 ml) | 0 g | 7.5 ml |
|  |  | Salt added to already prepared foods | 1 pinch (2 g) | 0 g | 3 g |
|  |  | Vegetable shortening | 1 teaspoon (5 g) | 0 g | 7.5 g |
|  |  | Margarine | 1 teaspoon (5 g) | 0 g | 7.5 g |
|  |  | Lard | 1 teaspoon (5 g) | 0 g | 7.5 g |
|  |  | Butter | 1 teaspoon (5 g) | 0 g | 7.5 g |

Note: this table will be updated according to the linear optimization programming.

**Online Supplementary Material 1.2. Feasibility, Acceptability, Quality and Usability Questionnaire**

English version of the feasibility, acceptability, quality and usability questionnaire

Questionnaire available at: <https://forms.gle/3e2zj4zB2SFa4qir7>

For complete written version, see online supplementary material 2.


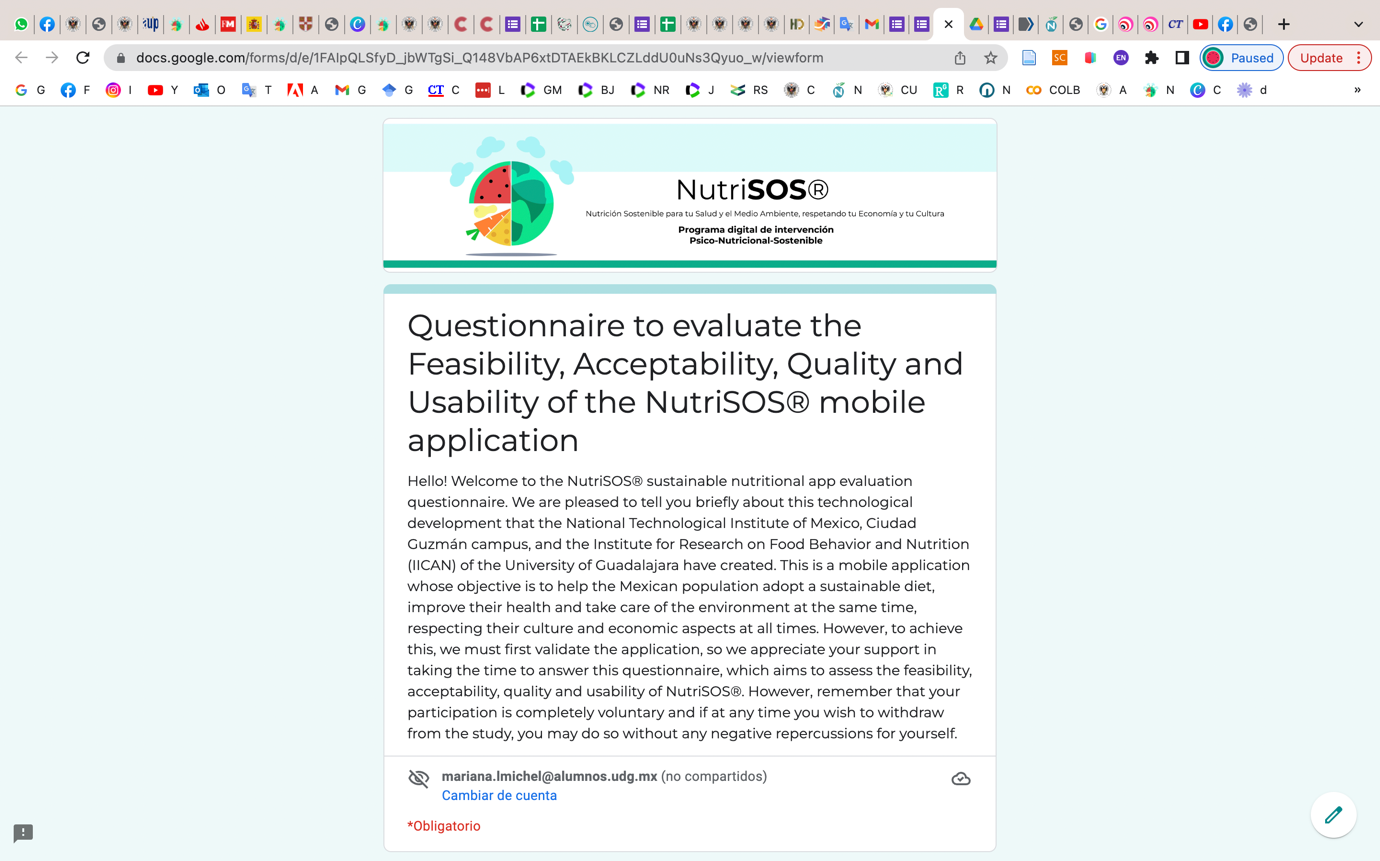


Spanish version of the feasibility, acceptability, quality and usability questionnaire

Questionnaire available at: <https://forms.gle/Vk8TRqsw7pFUTAd66>

For complete written version, see online supplementary material 3.


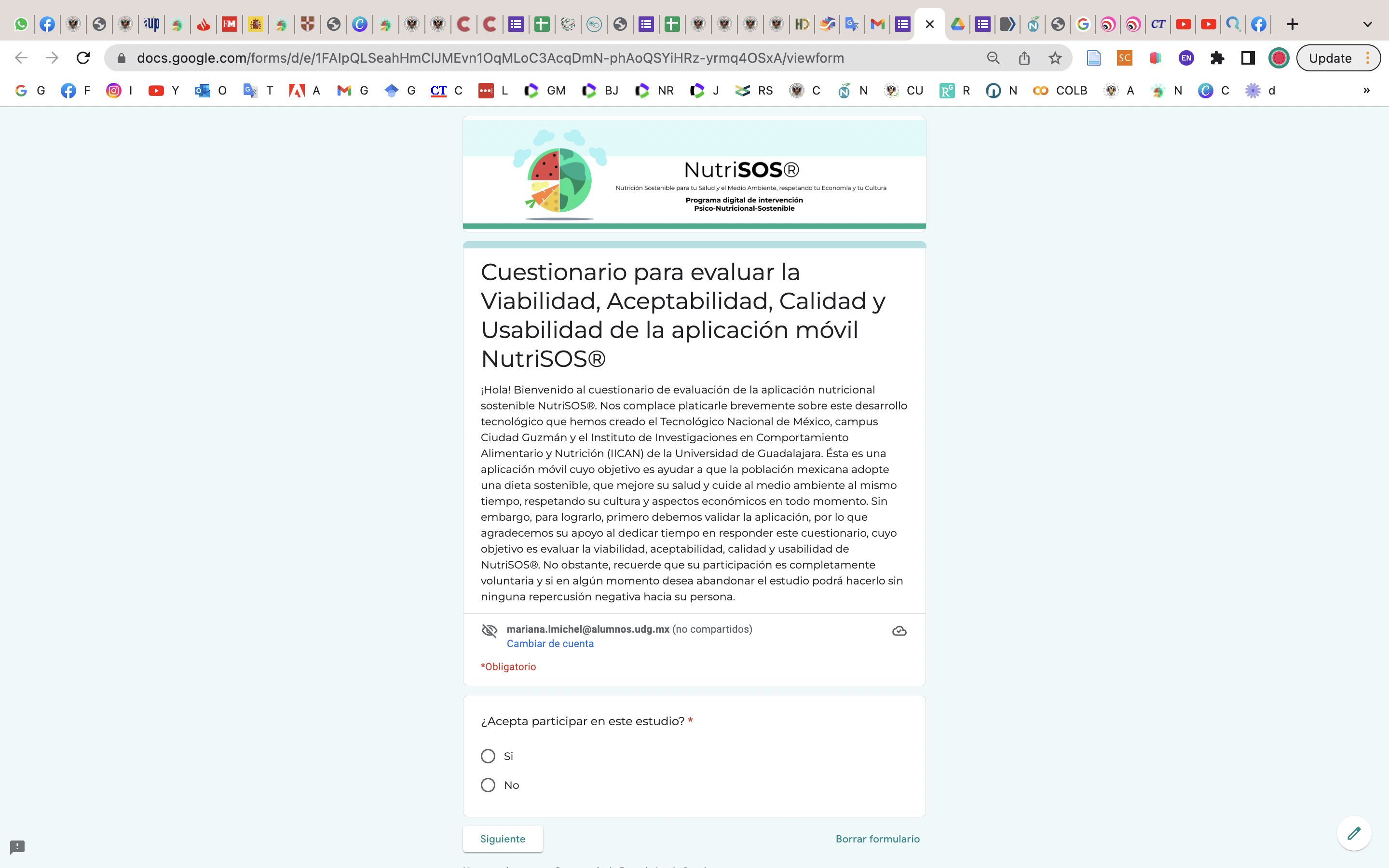


**Online Supplementary Material 1.3. Outcomes of the study**

Table SM1.3.1. Primary outcomes of the study

| **Sustainable diet dimension** | **Primary outcomes** | **Time frame** | **Measurement** |
| --- | --- | --- | --- |
| **P.1) Health and nutrition** | **P.1.1) Dietetics** |  |  |
| **P.1.1) Food and nutrient needs, food security, and accessibility (dietetics)** | P.1.1.1) Change from baseline diet quality at week 8 and 15 | Baseline (week 0), monitoring measure (week 8) and end of intervention (week 15) | For the assessment of diet quality, the Alternate Mexican Diet Quality Index (IACDMx) will be used. This is automatically calculated in the Nutriecology® software by responding a validated adapted Food Frequency Questionnaire (FFQ), which will be administrated to each participant by the nutritionist of the study (not self-reported). |
|  | P.1.1.2) Change from baseline dietary intake at week 8 and 15 |  | Food groups consumption will be evaluated through a 24-hour recall administrated by the nutritionist in the Nutriecology® software, and dietary records that the subjects will load to the application. |
|  | P.1.1.2.1) Change from baseline Mexican foods and dishes intake (g) at week 8 and 15 |  |  |
|  | P.1.1.2.2) Change from baseline Fruits and vegetables (g) consumption at week 8 and 15 |  |  |
|  | P.1.1.2.3) Change from baseline Whole grains (g) intake at week 8 and 15 |  |  |
|  | P.1.1.2.4) Change from baseline Legumes (g) consumption at week 8 and 15 |  |  |
|  | P.1.1.2.5) Change from baseline Dairy products (g) intake at week 8 and 15 |  |  |
|  | P.1.1.2.6) Change from baseline Seeds and healthy fats (g) consumption at week 8 and 15 |  |  |
|  | P.1.1.2.7) Change from baseline Eggs (g) consumption at week 8 and 15 |  |  |
|  | P.1.1.2.8) Change from baseline  Fish and seafood (g) consumption at week 8 and 15 |  |  |
|  | P.1.1.2.9) Change from baseline  Chicken (g)consumption at week 8 and 15 |  |  |
|  | P.1.1.2.10) Change from baseline  Beef, pork, goat, lamb and processed meats (g) intake at week 8 and 15 |  |  |
|  | P.1.1.2.11) Change from baseline  Ultra-processed foods (g) intake at week 8 and 15 |  |  |
|  | P.1.1.2.11) Change from baseline  Added and free sugars, and trans and saturated fats (g) intake at week 8 and 15 |  |  |
|  | **P.1.2.1) Anthropometric and body composition data** |  |  |
| **P.1.2) Well-being and health** | P.1.2.1.1) Change from baseline BMI at week 8 and 15 | Baseline (week 0), monitoring measure (week 8) and end of intervention (week 15) | Calculated from weight/height^2.^ Weight will be taken parallel to body fat and height will be evaluated with a Smartmet® stadiometer according to the Frankfurt plane with the participant barefoot. |
|  | P.1.2.1.2) Change from baseline body fat percentage at week 8 and 15 |  | Weight and fat levels will be measured with a bioimpedance scale TANITA BC-601 FITSCAN® with the patients without shoes, with light clothes, in a minimum 2 hours fasting. Women will be asked not to the during their menstrual periods, for avoiding hydration alterations that can alter bioimpedance results. Also, metallic objects will be removed to the participants during the measures. |
|  | **P.1.2.2) Biochemical data (biomarkers related to dietary interventions)** |  |  |
|  | P.1.2.2.1) Change from baseline glucose levels at week 8 and 15 | Baseline (week 0), monitoring measure (week 8) and end of intervention (week 15) | Biomarkers will be analyzed by colorimetric enzymatic methods, through blood samples that will be taken by a nurse from the antecubital vein of each participant, following the protocol from the Mexican Health Secretary. After an overnight fast of 8 to 12 hours, samples will be taken by specialists and will be centrifuged at 3500 rpm for 15 min to separate the serum. Samples will be aliquoted and stored at -20°C until the day of testing. Tourniquets, alcohol swabs, 10 mL syringes, and blood collection tubes will be required. |
|  | P.1.2.2.2) Change from baseline LDL cholesterol levels at week 8 and 15 |  |  |
|  | P.1.2.2.3) Change from baseline HDL cholesterol levels at week 8 and 15 |  |  |
|  | P.1.2.2.4) Change from baseline total cholesterol levels at week 8 and 15 |  |  |
|  | P.1.2.2.5) Change from baseline triglycerides levels at week 8 and 15 |  |  |

Table SM1.3.1. Continuation. Primary outcomes of the study

| **Sustainable diet dimension** | **Primary outcomes** | **Time frame** | **Measurement** |
| --- | --- | --- | --- |
| **P.1) Health and nutrition** | **P.1.2.2) Biochemical data (biomarkers related to dietary interventions)** |  |  |
| **P.1.2) Well-being and health** | **P.1.2.2.6) Change from baseline gut microbiota at week 8 and 15** | Baseline (week 0), monitoring measure (week 8) and end of intervention (week 15) | The relative abundance of each bacterium will be determined by means of qPCR with specific primers. Analysis will be performed in fecal samples that will be collected in a sterile stool container using a clean kit, including gloves and a spatula to be delivered to the participants. Once collected, the samples will be divided into aliquots and stored at -80 ºC, following a method used for the Mexican population. |
|  | P.1.2.2.6.1) Change from baseline relative abundance of Firmicutes at week 8 and 15 |  |  |
|  | P.1.2.2.6.2) Change from baseline relative abundance of Bacteroidetes at week 8 and 15 |  |  |
|  | P.1.2.2.6.3) Change from baseline relative abundance of Lactobacillus at week 8 and 15 |  |  |
|  | P.1.2.2.6.4) Change from baseline relative abundance of Bifidobacterium at week 8 and 15 |  |  |
|  | P.1.2.2.6.5) Change from baseline relative abundance of Faecalibacterium prausnitzii at week 8 and 15 |  |  |
|  | P.1.2.2.6.6) Change from baseline relative abundance of Akkermansia muciniphila at week 8 and 15 |  |  |
|  | P.1.2.2.6.7) Change from baseline relative abundance of Prevotella copri at week 8 and 15 |  |  |
|  | P.1.2.2.6.8) Change from baseline relative abundance of Bilophila wadsworthia at week 8 and 15 |  |  |
|  | P.1.2.2.6.9) Change from baseline relative abundance of Clostridium coccoides at week 8 and 15 |  |  |
|  | P.1.2.2.6.10) Change from baseline relative abundance of Streptococcus thermophilus at week 8 and 15 |  |  |
|  | **P.1.2.3) Physical activity** |  |  |
|  | P.1.2.3.1) Change from baseline physical activity at week 8 and 15 | Baseline (week 0), monitoring measure (week 8) and end of intervention (week 15) | Physical activity levels will be assessed through specific questions about physical activity type, frequency, intensity, and duration, using the IPAQ Scoring Protocol. |
| **P.2) Environment** | **P.2.1) Biodiversity, environment, and climate** |  |  |
|  | P.2.1.1) Change from baseline dietary carbon footprint at week 8 and 15 | Baseline (week 0), monitoring measure (week 8) and end of intervention (week 15) | Carbon footprint will calculate using the Life Cycle Assessment method. Specifically, the SHARP-Indicators Database will be used. That database was recently used for the estimation of the greenhouse gas emissions generated by the Mexican diet. Also, the data provided there consider the production, trade, and transport of foods. Besides, the calculations were adjusted for consumption amount using conversions factors for production, edible portion, cooking losses and gains, and food losses and waste. Calculations will be performed in the Nutriecology® Software. |
|  | **P.2.2) Eco-friendly, local seasonal foods** |  |  |
|  | P.2.2.1) Change from baseline dietary water footprint (total, green, blue, and grey) at week 8 and 15 | Baseline (week 0), monitoring measure (week 8) and end of intervention (week 15) | Water footprint will be calculated using the Water Footprint Assessment method in its version for Mexico’s context. The water footprint of each food and ingredient will be calculated applying correction factors to convert cooked to uncooked foods and peeled to unpeeled foods. Also, water involved in cooking and food washing will be evaluated. Calculations will be performed using the Nutriecology® Software. |

Table SM1.3.2. Secondary outcomes of the study

| **Sustainable diet dimension** | **Secondary outcomes** | **Time frame** | **Measurement** |
| --- | --- | --- | --- |
| **S.1) Health and Nutrition** | **S.1.1) Food and nutrient needs, food security, and accessibility (dietetics)** |  |  |
| **S.1.1) Food and nutrient needs, food security, and accessibility (dietetics)** | **S.1.1.1) Change from baseline energy and nutrient intake at week 8 and 15** | Baseline (week 0), monitoring measure (week 8) and end of intervention (week 15) | Energy and nutrient intake will be calculated based on the FFQ, the 24-hour recalls, and the dietary records already applied to assess diet quality and dietary intake. Nutrient calculation will be performed using the Nutriecology® software. |
|  | S.1.1.1.1) Change from baseline Energy (Kcal) intake at week 8 and 15 |  |  |
|  | S.1.1.1.2) Change from baseline Fiber (g) intake at week 8 and 15 |  |  |
|  | S.1.1.1.3) Change from baseline Carbohydrates (g) intake at week 8 and 15 |  |  |
|  | S.1.1.1.4) Change from baseline Sugar (g) intake at week 8 and 15 |  |  |
|  | S.1.1.1.5) Change from baseline Protein (g) intake at week 8 and 15 |  |  |
|  | S.1.1.1.6) Change from baseline Protein (g) intake at week 8 and 15 |  |  |
|  | S.1.1.1.7) Change from baseline Lipids (g) intake at week 8 and 15 |  |  |
|  | S.1.1.1.8) Change from baseline Saturated fatty acids (g) intake at week 8 and 15 |  |  |
|  | S.1.1.1.9) Change from baseline Monounsaturated fatty acids (g) intake at week 8 and 15 |  |  |
|  | S.1.1.1.10) Change from baseline Polyunsaturated fatty acids (g) intake at week 8 and 15 |  |  |
|  | S.1.1.1.11) Change from baseline Cholesterol (mg) intake at week 8 and 15 |  |  |
|  | S.1.1.1.12) Change from baseline Calcium (mg) intake at week 8 and 15 |  |  |
|  | S.1.1.1.13) Change from baseline Phosphorus (mg) intake at week 8 and 15 |  |  |
|  | S.1.1.1.14) Change from baseline Iron (mg) intake at week 8 and 15 |  |  |
|  | S.1.1.1.15) Change from baseline Magnesium (mg) intake at week 8 and 15 |  |  |
|  | S.1.1.1.16) Change from baseline Sodium (mg) intake at week 8 and 15 |  |  |
|  | S.1.1.1.17) Change from baseline Potassium (mg) intake at week 8 and 15 |  |  |
|  | S.1.1.1.18) Change from baseline Zinc (mg) intake at week 8 and 15 |  |  |
|  | S.1.1.1.19) Change from baseline Selenium (mg) intake at week 8 and 15 |  |  |
|  | S.1.1.1.20) Change from baseline Vitamin A (µg RE) intake at week 8 and 15 |  |  |
|  | S.1.1.1.21) Change from baseline Ascorbic acid (mg) intake at week 8 and 15 |  |  |
|  | S.1.1.1.22) Change from baseline Thiamine (mg) intake at week 8 and 15 |  |  |
|  | S.1.1.1.23) Change from baseline Riboflavin (mg) intake at week 8 and 15 |  |  |
|  | S.1.1.1.24) Change from baseline Niacin (mg) intake at week 8 and 15 |  |  |
|  | S.1.1.1.25) Change from baseline Pyridoxine (mg) intake at week 8 and 15 |  |  |
|  | S.1.1.1.26) Change from baseline Folic acid (µg) intake at week 8 and 15 |  |  |
|  | S.1.1.1.27) Change from baseline Cobalamin (mg) intake at week 8 and 15 |  |  |
|  | S.1.1.1.28) Change from baseline Ethanol (g)intake at week 8 and 15 |  |  |
|  | S.1.2.1) Clinical data |  |  |
|  | S.1.2.1.1) Change from baseline systolic and diastolic blood pressure at week 8 and 15 | Baseline (week 0), monitoring measure (week 8) and end of intervention (week 15) | Will evaluated by physical exploration, searching for hyperpigmentation and thickening of the skin with velvety in visible flex areas (axilla, anterior ulnar area, and posterior and lateral region of the neck) |
|  | S.1.2.1.3) Change from signs of nutrient deficiencies or excess at week 8 and 15 |  | Will assessed by clinical exploration regarding hair, nails, mouth, tongue, edema, and mucous membranes appearance |

Table SM1.3.2. Continuation. Secondary outcomes of the study

| **Sustainable diet dimension** | **Secondary outcomes** | **Time frame** | **Measurement** |
| --- | --- | --- | --- |
| **S.1) Health and Nutrition** | **S.1.2.2) Anthropometric and body composition data** |  |  |
| **S.1.1) Food and nutrient needs, food security, and accessibility (dietetics)** | S.1.2.2.1) Change from baseline muscle mass at week 8 and 15 | Baseline (week 0), monitoring measure (week 8) and end of intervention (week 15) | Will be evaluated with a bioimpedance scale TANITA BC-601 FITSCAN® |
|  | S.1.2.2.2) Change from baseline visceral fat at week 8 and 15 |  | Will be assessed by a bioimpedance scale TANITA BC-601 FITSCAN® |
|  | S.1.2.2.3) Change from baseline weight at week 8 and 15 |  | Will be evaluated with a bioimpedance scale TANITA BC-601 FITSCAN® |
|  | S.1.2.2.4) Change from baseline waist circumference at week 8 and 15 |  | Will be evaluated with a Lufkin® metal tape measure. |
|  | S.1.2.2.5) Change from baseline hips circumference at week 8 and 15 |  | Will be evaluated with a Lufkin® metal tape measure. |
| **S.5) Psychology (behavioral aspects)** | **S.5) Psychology (behavioral aspects)** |  |  |
|  | S.5.1) Change from Baseline Nutritional-sustainable knowledge at week 8 and 15 | Baseline (week 0), monitoring measure (week 8) and end of intervention (week 15) | Will be evaluated through a designed questionnaire based on the psychological capacity presented in the COM-B model. It includes a series of questions for evaluating nutritional-sustainable knowledge, which are related to the established behavioral objectives, and was designed based on the revised version of the General Nutrition Knowledge Questionnaire, considering scores for each question. Its development also considered the recommendations provided in the manual for designing nutritional knowledge questionnaires of the Food and Agriculture Organization of the United Nations (FAO). Besides, it includes questions on sustainable food consumption. |

Table SM1.3.3. Exploratory outcomes of the study

| **Sustainable diet dimension** | **Secondary outcomes** | **Time frame** | **Measurement** |
| --- | --- | --- | --- |
| **D.5) Psychology (behavioral aspects)** | **D.5) Psychology (behavioral aspects)** |  |  |
|  | D.5.1) Psychological aspects from the COM-B model | Baseline (week 0) | Psychological aspects from the COM-B model will be evaluated by a structure questionnaire. All dimensions of the COM-B model will be assessed in a designed questionnaire based on Brain et al. The questions are related to physical and psychological ability, automatic and reflexive motivation, and physical and social opportunity. Questions were adapted according to the context of sustainable diets: (1) Capacity: knowledge of sustainable nutrition, preparation skills food and preparation capacity; (2) Opportunity: time to eat and prepare food, access to food, and food storage; (3) Motivation: the desire to change eating habits, emotions involved in food consumption and habits that participants are willing to change. |

Table SM1.3.4. Descriptive variables of the study

| **Sustainable diet dimension** | **Secondary outcomes** | **Time frame** | **Measurement** |
| --- | --- | --- | --- |
| **D.1) Health and Nutrition** | **D.1.2.2) Anthropometric and body composition data** |  |  |
| **D.1.2) Well-being and health** | D.1.2.2.1) Height | Baseline (week 0) | Will be evaluated with a Smartmet® stadiometer according to the Frankfurt plane with the participant barefoot |
|  | D.1.2.2.2) Clinical history |  | A baseline clinical history will be applied to properly prescribe dietetic plans. The clinical history will cover pathologies suffering, pathological family history, sun exposure, medicaments, and supplement use. Other factors influencing gut microbiota, such as hygiene aspects, delivery type, and lactation when baby, will also be included. Additionally, question regarding food preparation, food shopping places, food preferences, allergies and intolerances, and about following specific diets at the moment of the evaluation, will be assessed by a questionnaire. |
| **D.3) Economy** | **D.3.1) Equity, and fair trade** |  |  |
|  | **D.3.1.1) Socioeconomic level** | Baseline (week 0) | The Socioeconomic baseline level of the population will be evaluated to provide affordable diets according to their economical possibilities. Socioeconomic level will be assessed by educational level, occupational level, and economic income according to classifications used for Mexico. |
|  | D.3.1.1.1) Educational level |  |  |
|  | D.3.1.1.2) Ocupacional level |  |  |
|  | D.3.1.1.3) Monthly outcome |  |  |
|  | D.3.1.2) Food prices |  | Food prices will be used for the design of the optimized dietary food guide and food plans, to ensure affordable diets in the participants. The prices of the most consumed foods in Mexico, which are reported in a recent exploratory study of the Mexican’s diet, will be investigated in fieldwork in local flea market and supermarkets. Besides, the databases of supermarkets will be consulted, as well as the food prices database of the National Institute of Statistics and Geography (INEGI). |
|  | **D.3.1.3) Sociodemographic data** |  | Sociodemographic data will be evaluated the initial questionnaire. |
|  | D.3.1.3.1) Sex |  |  |
|  | D.3.1.3.2) Age |  |  |
|  | D.3.1.3.3) Country of origin |  |  |
|  | D.3.1.3.4) Residence city |  |  |
|  | D.3.1.3.5) Civil status |  |  |
|  | D.3.1.3.6) Religion |  |  |
| **D.4) Culture and society** | **D.4.1) Cultural heritage, and skills** |  |  |
|  | D.4.1.1) Food culture related to celebrations and traditions | Baseline (week 0) | Food culture related to celebrations and traditions will be investigated in a literature review. According to the Traditional Mexican diet (Colonized and Milpa diet), recipes will be designed to instruct participant about food preparation. Also, both the dietary guideline and food plans will be developed according to the Traditional Mexican diet, and diet plans will be prescribed according to personal preferences, needs and contexts. |

**Online Supplementary Material 1.4. Data for sample size calculation according to primary outcomes**

Table SM1.4.1 Data for sample size calculation according to primary outcomes

| **Sustainable diet dimension** |  | **Confidence level (%)** | **Minimal clinically important difference (μ1-μ2)** | **Standard deviation (ó)** | **Significance level (Zα/2) (5%)** | **Statistical power (80%) (Zβ)** | **Total sample (n)** | **Sample per group (n)** | **n (10% drop outs)** | **Final sample** |
| --- | --- | --- | --- | --- | --- | --- | --- | --- | --- | --- |
|  | **Primary outcomes** |  |  |  |  |  |  |  |  |  |
| **P.1) Health and nutrition** | **P.1.1) Dietetics** |  |  |  |  |  |  |  |  |  |
| **P.1.1) Food and nutrient needs, food security, and accessibility (dietetics)** | P.1.1.1) Diet quality (points) | 95 | 0.5 | 1.195 | 1.96 | 0.84 | 90 | 45 | 50 | 100 |
|  | **P.1.1.2) Change from baseline dietary intake at week 8 and 15 (%)** |  |  |  |  |  |  |  |  |  |
|  | P.1.1.2.1) Change from baseline Mexican foods and dishes intake (portion*) at week 8 and 15 | 95 | 0.5 | 1.195 | 1.96 | 0.84 | 90 | 45 | 50 | 100 |
|  | P.1.1.2.2) Change from baseline Fruits and vegetables (portion*) consumption at week 8 and 15 | 95 | 0.5 | 1.195 | 1.96 | 0.84 | 90 | 45 | 50 | 100 |
|  | P.1.1.2.3) Change from baseline Whole grains (portion*) intake at week 8 and 15 | 95 | 0.5 | 1.195 | 1.96 | 0.84 | 90 | 45 | 50 | 100 |
|  | P.1.1.2.4) Change from baseline Legumes (portion*) consumption at week 8 and 15 | 95 | 0.5 | 1.195 | 1.96 | 0.84 | 90 | 45 | 50 | 100 |
|  | P.1.1.2.5) Change from baseline Dairy products (portion*) intake at week 8 and 15 | 95 | 0.5 | 1.195 | 1.96 | 0.84 | 90 | 45 | 50 | 100 |
|  | P.1.1.2.6) Change from baseline Seeds and healthy fats (portion*) consumption at week 8 and 15 | 95 | 0.5 | 1.195 | 1.96 | 0.84 | 90 | 45 | 50 | 100 |
|  | P.1.1.2.7) Change from baseline Eggs (portion*) consumption at week 8 and 15 | 95 | 0.5 | 1.195 | 1.96 | 0.84 | 90 | 45 | 50 | 100 |
|  | P.1.1.2.8) Change from baseline Fish and seafood (portion*) consumption at week 8 and 15 | 95 | 0.5 | 1.195 | 1.96 | 0.84 | 90 | 45 | 50 | 100 |
|  | P.1.1.2.9) Change from baseline Chicken (portion*) consumption at week 8 and 15 | 95 | 0.5 | 1.195 | 1.96 | 0.84 | 90 | 45 | 50 | 100 |
|  | P.1.1.2.10) Change from baseline Beef, pork, goat, lamb and processed meats (portion*) intake at week 8 and 15 | 95 | 0.5 | 1.195 | 1.96 | 0.84 | 90 | 45 | 50 | 100 |
|  | P.1.1.2.11) Change from baseline Ultra-processed foods (portion*) intake at week 8 and 15 | 95 | 0.5 | 1.195 | 1.96 | 0.84 | 90 | 45 | 50 | 100 |
|  | P.1.1.2.11) Change from baseline Added and free sugars, and trans and saturated fats (portion*) intake at week 8 and 15 | 95 | 0.5 | 1.195 | 1.96 | 0.84 | 90 | 45 | 50 | 100 |
|  | **P.1.2.1) Anthropometric and body composition data** |  |  |  |  |  |  |  |  |  |
| **P.1.2) Well-being and health** | P.1.2.1.1) Change from baseline BMI at week 8 and 15 (kg/cm)^2^ | 95 | 0.5 | 1.195 | 1.96 | 0.84 | 90 | 45 | 50 | 100 |
|  | P.1.2.1.2) Change from baseline body fat percentage at week 8 and 15 (%) | 95 | 0.5 | 1.195 | 1.96 | 0.84 | 90 | 45 | 50 | 100 |
|  | **P.1.2.2) Biochemical data (biomarkers related to dietary interventions)** |  |  |  |  |  |  |  |  |  |
|  | P.1.2.2.1) Change from baseline glucose levels at week 8 and 15 *(mmol/L)* | 95 | 0.5 | 1.195 | 1.96 | 0.84 | 90 | 45 | 50 | 100 |
|  | P.1.2.2.2) Change from baseline LDL cholesterol levels at week 8 and 15 *(mmol/L)* | 95 | 0.5 | 1.195 | 1.96 | 0.84 | 90 | 45 | 50 | 100 |
|  | P.1.2.2.3) Change from baseline HDL cholesterol levels at week 8 and 15 *(mmol/L)* | 95 | 0.5 | 1.195 | 1.96 | 0.84 | 90 | 45 | 50 | 100 |
|  | P.1.2.2.4) Change from baseline total cholesterol levels at week 8 and 15 *(mmol/L)* | 95 | 0.5 | 1.195 | 1.96 | 0.84 | 90 | 45 | 50 | 100 |
|  | P.1.2.2.5) Change from baseline triglycerides levels at week 8 and 15 *(mmol/L)* | 95 | 0.5 | 1.195 | 1.96 | 0.84 | 90 | 45 | 50 | 100 |
|  | **P.1.2.2.6) Change from baseline gut microbiota at week 8 and 15** |  |  |  |  |  |  |  |  |  |
|  | P.1.2.2.6.1) Change from baseline relative abundance of Firmicutes at week 8 and 15 | 95 | 0.5 | 1.195 | 1.96 | 0.84 | 90 | 45 | 50 | 100 |
|  | P.1.2.2.6.2) Change from baseline relative abundance of Bacteroidetes at week 8 and 15 | 95 | 0.5 | 1.195 | 1.96 | 0.84 | 90 | 45 | 50 | 100 |
|  | P.1.2.2.6.3) Change from baseline relative abundance of Lactobacillus at week 8 and 15 | 95 | 0.5 | 1.195 | 1.96 | 0.84 | 90 | 45 | 50 | 100 |
|  | P.1.2.2.6.4) Change from baseline relative abundance of Bifidobacterium at week 8 and 15 | 95 | 0.5 | 1.195 | 1.96 | 0.84 | 90 | 45 | 50 | 100 |
|  | P.1.2.2.6.5) Change from baseline relative abundance of Faecalibacterium prausnitzii at week 8 and 15 | 95 | 0.5 | 1.195 | 1.96 | 0.84 | 90 | 45 | 50 | 100 |
|  | P.1.2.2.6.6) Change from baseline relative abundance of Akkermansia muciniphila at week 8 and 15 | 95 | 0.5 | 1.195 | 1.96 | 0.84 | 90 | 45 | 50 | 100 |
|  | P.1.2.2.6.7) Change from baseline relative abundance of Prevotella copri at week 8 and 15 | 95 | 0.5 | 1.195 | 1.96 | 0.84 | 90 | 45 | 50 | 100 |
|  | P.1.2.2.6.8) Change from baseline relative abundance of Bilophila wadsworthia at week 8 and 15 | 95 | 0.5 | 1.195 | 1.96 | 0.84 | 90 | 45 | 50 | 100 |
|  | P.1.2.2.6.9) Change from baseline relative abundance of Clostridium coccoides at week 8 and 15 | 95 | 0.5 | 1.195 | 1.96 | 0.84 | 90 | 45 | 50 | 100 |
|  | P.1.2.2.6.10) Change from baseline relative abundance of Streptococcus thermophilus at week 8 and 15 | 95 | 0.5 | 1.195 | 1.96 | 0.84 | 90 | 45 | 50 | 100 |
|  | **P.1.2.3) Physical activity** |  |  |  |  |  |  |  |  |  |
|  | P.1.2.3.1) Change from baseline physical activity at week 8 and 15 (points) | 95 | 0.5 | 1.195 | 1.96 | 0.84 | 90 | 45 | 50 | 100 |
| **P.2) Environment** | **P.2.1) Biodiversity, environment, and climate** |  |  |  |  |  |  |  |  |  |
|  | P.2.1.1) Change from baseline dietary carbon footprint at week 8 and 15 (kgCO_2_eq/day) | 95 | 0.5 | 1.195 | 1.96 | 0.84 | 90 | 45 | 50 | 100 |
|  | P.2.2.1) Change from baseline dietary water footprint (total, green, blue, and grey) at week 8 and 15 (m^3^/day) | 95 | 0.5 | 1.195 | 1.96 | 0.84 | 90 | 45 | 50 | 100 |

*Portions based on the Mexican Equivalent System.

**Online Supplementary Material 1.5. Screening for participants selection**

Table SM1.5.1. Inclusion, exclusion, and elimination criteria

| Study Criteria | | |
| --- | --- | --- |
| Inclusion | Exclusion | Elimination |
| Being between 18 and 35 years old | Not signing the informed consent | Not completing the questionnaires |
| Being Mexican | Not accepting to donate blood and/or stool samples | Do not allow the extraction of blood and / or delivery of stool sample |
| Reside in the South of Jalisco for at least 1 year | Not being able to stand up to take anthropometric data | Failure to complete the 7-week intervention or 7-week follow-up |
| Levels of physical activity below what is recommended and what is established as a criterion for inclusion in the study (Table SM2) | Perform levels of physical activity above the minimum established as criteria for inclusion in the study (Table SM2) |  |
| Consuming amounts of food below or above that established as criteria for inclusion in the study or in a lesser or greater frequency than recommended, according to the type of food (Table SM2) | Consume adequate levels of the foods to be promoted in the intervention program (Table SM2) |  |
| Have a Smartphone | Being pregnant or lactating |  |
| Not having consumed antibiotics at least 3 months before the intervention | Suffer from a chronic disease such as type 2 diabetes mellitus, arterial hypertension, dyslipidemia, under medication |  |
| Have a BMI between 18.5 and 40 | Suffering from an autoimmune disease such as type 1 diabetes, hypo or hyperthyroidism |  |
| Not having a medical diagnosis of chronic disease under pharmacological treatment | Having a gastrointestinal disease such as Crohn's disease, ulcerative colitis, etc. |  |
| Not having a medical diagnosis of gastrointestinal disease | Having a mood alteration or a eating disorder |  |
|  | Having used antibiotics less than 3 months ago |  |
|  | Taking antidepressant medications or corticosteroids |  |
|  | Consume probiotics or nutritional supplements, except protein powder |  |

Table SM1.5.2 Cut-off points to establish inadequate consumption and physical activity, to be included in the research

| Physical activity | Inclusion criteria | | | Reference |
| --- | --- | --- | --- | --- |
| Type of physical activity | Light  < 3 times per week  < 30 minutes per day | | |  |
| Weekly frequency |  |  |  |  |
| Minutes per day |  |  |  |  |
| Food group | Consumption to be included in the study | and / or | Frequency of consumption to be included in the study |  |
| Mexican food and dishes | < 100 g p^-1^d^-1^ |  | < 7 times a week | ^(1,2)^ |
| Fruits | < 200 g p^-1^d^-1^ |  | < 7 times a week | ^(3)^ |
| Vegetables | < 300 g p^-1^d^-1^ |  | < 7 times a week | ^(3)^ |
| Whole cereals | < 200 g p^-1^d^-1^ |  | < 7 times a week | ^(3,4)^ |
| Legumes | < 60 g p^-1^d^-1^ |  | < 7 times a week | ^(3,4)^ |
| Seeds and healthy fats | < 80 g p^-1^d^-1^ |  | < 7 times a week | ^(3,4)^ |
| Dairy products | > 250 ml p^-1^d^-1^ |  | > 5 times a week | ^(3,4)^ |
| Eggs | > 25 g p^-1^d^-1^ |  | > 5 times a week | ^(3,4)^ |
| Fish and shellfish | > 28 g p^-1^d^-1^ |  | > 3 times a week | ^(3,4)^ |
| Chicken | > 29 g p^-1^d^-1^ |  | > 3 times a week | ^(3,4)^ |
| Red and processed meats | > 7 g p^-1^d^-1^ |  | > once a week | ^(3)^ |
| Ultra-processed foods | > 10 g p^-1^d^-1^ |  | > once a week | ^(3,4)^ |
| Added and free sugars and foods high in trans and saturated fats | > 31 g p^-1^d^-1^ |  | > once a week | ^(3)^ |

Note:

1. Valerino-Perea S, Lara-Castor L, Armstrong MEG, et al. (2019) Definition of the Traditional Mexican Diet and Its Role in Health: A Systematic Review. Nutrients 11, 2803. Multidisciplinary Digital Publishing Institute.

2. Almaguer González J, García Ramírez H, Padilla Mirazo M, et al. (2019) Fortalecimiento de la salud con comida, ejercicio y buen humor: La dieta de la milpa. Modelo de alimentación mesoamericana saludable y culturalmente pertinente. Secretaría de Salud. Google Docs. https://drive.google.com/file/d/1n05pSVGY09FlzW91Rt8IZk_6J-KIPRJT/view?usp=sharing&usp=embed_facebook (accessed January 2021).

3. Willett W, Rockström J, Loken B, et al. (2019) Food in the Anthropocene: the EAT–Lancet Commission on healthy diets from sustainable food systems. The Lancet 393, 447–492. Elsevier.

4. Lares-Michel M, Housni FE, Aguilera Cervantes VG, et al. (2021) Eat well to fight obesity… and save water: The water footprint of different diets and caloric intake and its relationship with adiposity. Front. Nutr. 8. Frontiers.

Figure SM1.5.1. Invitation poster for participants screening (English version)

Figure SM1.5.2. Invitation poster for participant's screening (Spanish version)

Screening questionnaire for participants selection

English version of the screening questionnaire for participants selection

Questionnaire available at: <https://forms.gle/fBhgbZjQibLhSdkA7>

For complete written version, see online supplementary material 4.


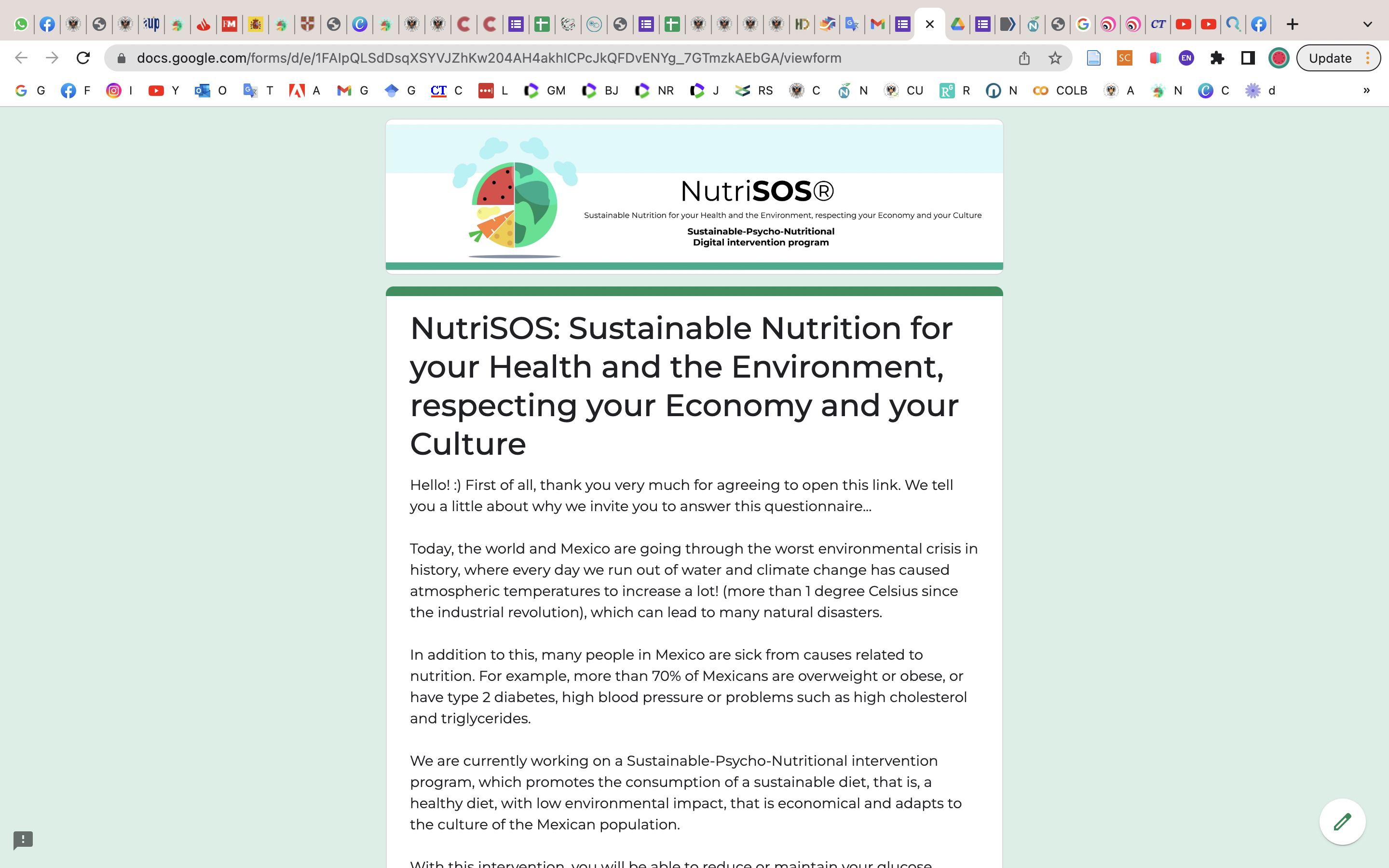


Figure SM1.5.3. Screening questionnaire for participants selection (English version)

Spanish version of the screening questionnaire for participants' selection

Questionnaire available at: <https://forms.gle/kSnwoi8R16YARem96>

For complete written version, see online supplementary material 5.


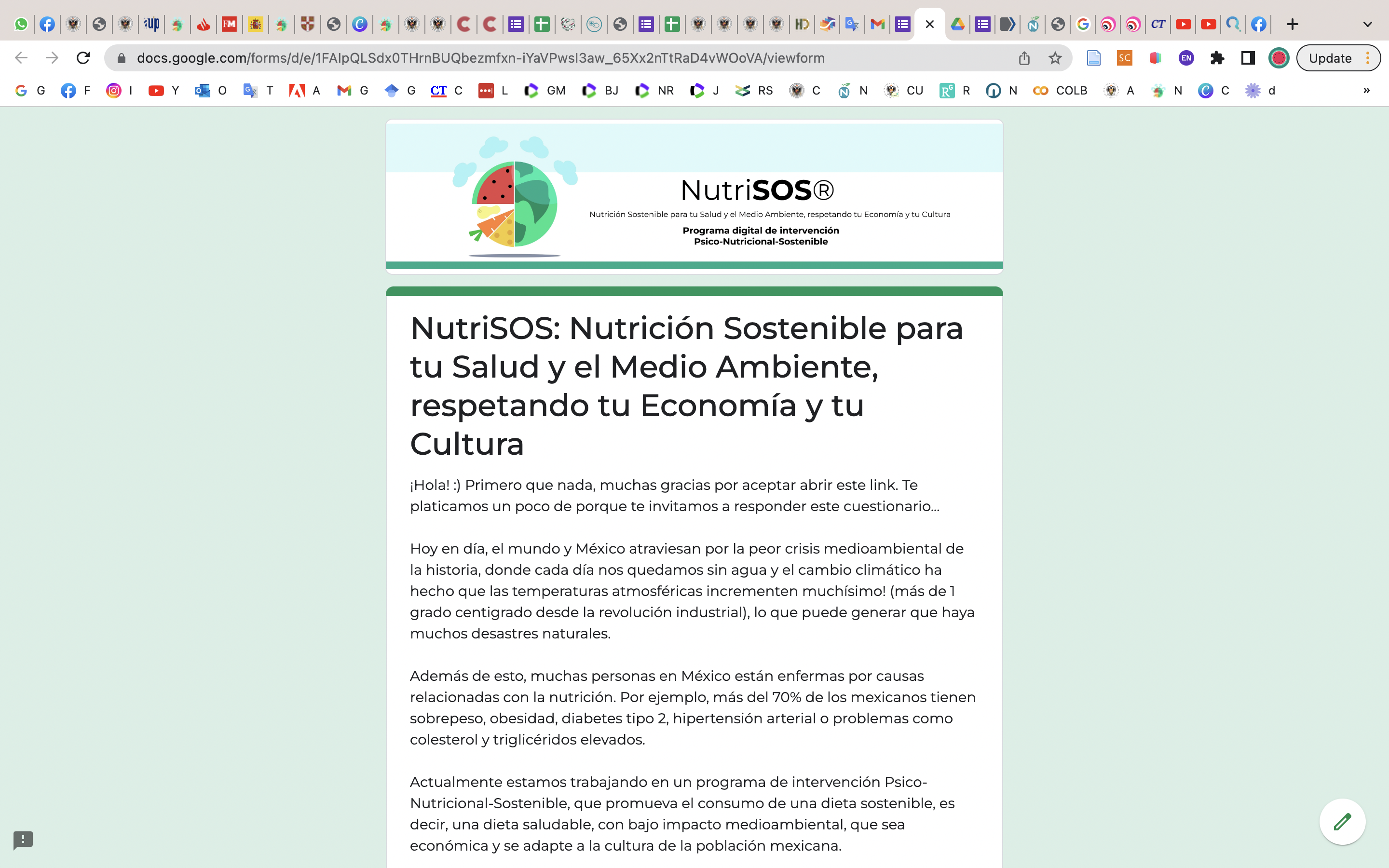


Figure SM1.5.4. Screening questionnaire for participants selection (Spanish version)

**Online Supplementary Material 1.6. Clinical history which will be uploaded to the mobile application (English version)**

Table SM1.6.1 Initial Assessment Questionnaire

| Clinic history | | | | | | | | | | |
| --- | --- | --- | --- | --- | --- | --- | --- | --- | --- | --- |
| General and sociodemographic data | | | | | | | | | | |
| Last name: | | | | Middle name: | | | | Name: | | |
| Cell phone: | | | | | | | Email: | | | |
| Age: | | | | | | | Date of Birth: | | | |
| Sex: | | | | | | | Country of birth: | | | |
| City of residence: | | | | | | | State of birth: | | | |
| Civil status: | | | | | | | Religion: | | | |
| Time living there: | | | | | | | Occupation | | | |
| Less than 1 month | | | | | |  | Officer, director or boss | | |  |
| 1 to 6 months | | | | | |  | Professional or technician | | |  |
| 6 to 12 months | | | | | |  | Auxiliary worker in administrative activities | | |  |
| 1 to 3 years | | | | | |  | Merchant, salesclerk, or sales agent | | |  |
| 3 to 6 years | | | | | |  | Workers in personal and surveillance services | | |  |
| 6 to 9 years | | | | | |  | Worker in agricultural, livestock, forestry, hunting or fishing activities | | |  |
| 10 years or more | | | | | |  | Craft worker | | |  |
| Lifetime | | | | | |  | Industrial machinery operator, assemblers, chauffeur or transport driver | | |  |
|  |  |  |  |  |  |  | Workers in elementary and support activities | | |  |
| Education | | | | | | | Student | | |  |
| Did not study | | | | | |  | Unemployed | | |  |
| Unfinished primary | | | | | |  | Retired | | |  |
| Finished elementary school | | | | | |  | Other (specify): | | |  |
| Unfinished high school | | | | | |  | Days a week that work | | | |
| High school finished | | | | | |  | 1 | | |  |
| Unfinished high school | | | | | |  | 2 | | |  |
| School completed | | | | | |  | 3 | | |  |
| Unfinished technical career | | | | | |  | 4 | | |  |
| Technical career finished | | | | | |  | 5 | | |  |
| Undergraduate student | | | | | |  | 6 | | |  |
| Unfinished degree | | | | | |  | 7 | | |  |
| Completed bachelor's degree | | | | | |  | Modality | | |  |
| Master's degree | | | | | |  | Face-to-face | | |  |
| Doctorate | | | | | |  | Online | | |  |
| Specialty | | | | | |  | Mixed | | |  |
| Other: | | | | | |  | Online until further notice | | |  |
| Individual and family monthly income (pesos per month MXN) | | | Individual expenditure on food per month (MXN pesos) | | | Household expenditure on food per month (MXN pesos) | | | Feeding | |
| –0 - 2,699 |  |  | | |  |  | 0 – 500 | | Favorite foods |  |
| 2,7–0 - 6,799 |  | | –0 - 200 | |  |  | 500 – 1,000 | |  | |
| 6,8–0 - 11,599 |  | | 2–0 - 500 | |  |  | 1,000 – 3,000 | | Foods you don't like | |
| 11,6–0 - 34,999 |  | | 500 – 800 | |  |  | 3,000 – 6,000 | |  | |
| 35,000 - 84,999 |  | | 800 – 1,000 | |  |  | 6,000 – 9,000 | | Food allergies and intolerances | |
| + 85,000 |  | | 1,500- 2,000 | |  |  | 10,0–0 - 13,000 | | Lactose |  |
|  |  | | 2,500 – 3,000 | |  |  | 13,000 – 16,000 | | Seafood |  |
|  |  | | 3,000 – 3,500 | |  |  | 16,000 – 20,000 | | Gluten |  |
|  |  | | + 4,000 | |  |  | + 20,000 | | Fresas |  |
| Diet and physical activity | | | | | | | | | Nuts |  |
| Main place of food purchase | | | Who cooks the food you eat? | | | | Are you currently following a specific diet? | | Peanuts |  |
| Tianguis (local market) |  | | Me | | |  |  |  | Others (specify): |  |
| Market |  | | Mother | | |  | Yes | | |  |
| Retail supermarket (Soriana, Walmart) |  | | Father | | |  | To lose weight | | |  |
| Wholesale supermarket (Sam's, Costco) |  | | Economic Kitchen | | |  | To gain weight | | |  |
| Butcher shop |  | | Restaurants | | |  | Ketogenic | | |  |
| Creamery |  | | I get it as a gift | | |  | Vegetarian (ovo-lacto, ovo or lacto) | | |  |
| Fruit store |  | | Others (specify): | | |  | Intermittent fasting | | |  |
| Others (specify): |  | |  |  |  |  | Vegan | | |  |
|  |  |  |  |  |  |  | Mediterranean | | |  |
|  |  |  |  |  |  |  | DASH or diet to stop hypertension | | |  |
|  |  |  |  |  |  |  | Others (specify): | | |  |

Table SM1.6.1 Continuation: Initial Assessment Questionnaire

| Clinic history | | | | | | |
| --- | --- | --- | --- | --- | --- | --- |
| Physical activity | | | | | | |
| Currently doing physical activity | | | | | Type of activity |  |
| Less than 3 times a week | | | |  | Walking |  |
| 3 or more days of vigorous activity of at least 20 minutes per day | | | |  | Jogging |  |
| 5 or more days of moderate to vigorous activity or walking at least 30 minutes per day | | | |  | Run |  |
| 3 days a week of very vigorous activity for at least 60 minutes a day | | | |  | Aerobics, Zumba, or dance |  |
| 7 days a week of moderate to vigorous activity or even walking for at least 60 minutes per day | | | |  | Cycling |  |
| Times a week | Type of activity | | Minutes a day | | Swimming |  |
| 1 | |  | 10 |  | Crossfit |  |
| 2 | |  | 20 |  | Weightlifting |  |
| 3 | |  | 30 |  | Multifunctional |  |
| 4 | |  | 40 |  | Focused exercises (write which ones, e.g., sit-ups, sit-ups, push-ups) |  |
| 5 | |  | 50 |  | Other: |  |
| 6 | |  | 60 |  |  | |
| 7 | |  | 90 |  |  |  |
| Intensity | | | 120 |  |  |  |
| Mild | |  | 150 |  |  |  |
| Moderate | |  | 180 |  |  |  |
| Intense | |  | 210 |  |  |  |
|  |  |  | 240 or more |  |  |  |
|  |  |  | Other: |  |  |  |
| Anthropometric data | | | | | | |
| Estimated weight for you: | |  | Size in centimeters estimated by you: |  |  |  |
| Height (centimeters): | |  | Weight (kg): |  | BMI: |  |
| Fat percentage: | |  | Muscle mass: |  | Body water: |  |
| Metabolic age: | |  | Metabolic rate: |  | Visceral fat: |  |
| Waist circumference: | |  | Hip circumference: |  | Bone mass: |  |
| Biochemical indicators | | | | Clinical indicators | | |
| Fasting glucose | | |  | Systolic blood pressure | |  |
| Glucose 2 hours after eating | | |  | Diastolic blood pressure | |  |
| Triglycerides | | |  | Acanthosis nigricans | |  |
| Total cholesterol | | |  | Signs and symptoms of nutritional deficiencies | | |
| LDL cholesterol | | |  | Overall status | |  |
| HDL cholesterol | | |  | Very tired | |  |
| Gut microbiota | | |  | Dizziness | |  |
| Comments: | | |  | Very thirsty | |  |
| Gastrointestinal signs and symptoms | | | | Frequent urination | |  |
| Abdominal swelling | | |  | Very hungry | |  |
| How often does it happen? | | |  | Do your feet or hands swell? | |  |
| Diarrhea | | |  | What time of day does it occur? | |  |
| How often does it happen? | | |  | How often does it happen? | |  |
| Constipation | | |  | How many hours do you spend sitting a day? | |  |
| How often does it happen? | | |  | How many hours do you spend standing a day? | |  |
| Reflux | | |  | Nosebleed | |  |
| How often does it happen? | | |  | How often does it happen? | |  |
| Abdominal swelling | | |  | Red spots on your skin or bruises for no reason | |  |
| How often does it happen? | | |  | How often does it happen? | |  |
| Nails | | | Hair | | | |
| Brittle nails | | | Hair loss | | |  |
| Have you recently performed any cosmetic treatment on your nails? | | | Brittle hair Is your hair dyed or undergoing any cosmetic treatment? | | |  |

Table SM1.6.1 Continuation: Initial Assessment Questionnaire

| Clinic history | | | |
| --- | --- | --- | --- |
| Signs and symptoms of nutritional deficiencies | | | |
| Mouth | | Sleep |  |
| Cuts at the corners of your mouth |  | How many hours do you sleep a day? |  |
| How often does it happen? |  | Do you rest at night? |  |
| Tongue swelling |  | Do you wake up during the night? |  |
| How often does it happen? |  | How often does it happen? |  |
| Gum inflammation |  | How many hours do you sleep a day? |  |
| How often does it happen? |  | Do you rest at day? |  |
| Solar exposition | | | |
| How many minutes do you expose yourself to the sun per day? | | Do you cover your skin with long-sleeved clothing, pants, a cap or a hat? | |
| Less than 5 minutes |  | Always |  |
| 5 to 10 minutes |  | Sometimes |  |
| 10 to 15 minutes |  | Never |  |
| 15 to 20 minutes |  | Do you use sunscreen? |  |
| 20 to 30 minutes |  | Yes |  |
| 30 minutes to 1 hour |  | No |  |
| More than 1 hour |  | How many days do you expose yourself to the sun per week? |  |
| Type of delivery |  | 1 |  |
| You were born for: |  | 2 |  |
| Vaginal delivery |  | 3 |  |
| Caesarean section |  | 4 |  |
| Notes: |  | 5 |  |
|  |  | 6 |  |
|  |  | 7 |  |
| Lactation |  | Write for how long | |
| Exclusive breastfeeding |  |  | |
| Formula feeding (if you received only artificial milk as food) |  | Observations: | |
| Mixed breastfeeding (if in addition to breastfeeding you received any artificial milk) |  |  |  |
| Supplemented breastfeeding (if non-dairy solid or liquid foods were received in addition to breastfeeding) |  |  |  |
| Mixed supplemented breastfeeding (if it was a breastfed infant, who also received some artificial milk and solid or liquid foods) |  |  |  |
| Complemented artificial feeding (if when breastfeeding you received artificial milk and solid or liquid foods) |  |  |  |
| Festivities | | | |
| Please write the main holidays you celebrate and on the right side write the main foods you eat on these holidays | | | |
| Festivity | Food eaten on the holiday | | |
|  |  | | |
|  |  | | |
|  |  | | |
|  |  | | |
|  |  | | |
|  |  | | |
|  |  | | |
| Hygiene habits | | | |
| How often do you take a bath? |  | | |
| How many times do you brush your teeth a day? |  | | |
| Is the street where you live paved? |  | | |
| What type of flooring does your house have? |  | | |
| Do you have pets? What type? |  | | |

Table SM1.6.1 Continuation: Initial Assessment Questionnaire

| Personal and family history | | | | | | | | | | | | | |
| --- | --- | --- | --- | --- | --- | --- | --- | --- | --- | --- | --- | --- | --- |
| Do you suffer from any disease? | | | | | | | | | | | | | |
| Mark pathology |  |  |  |  |  |  |  |  |  |  |  |  |  |
|  | Type 2 diabetes | Arterial hypertension | Elevated triglycerides | High cholesterol | Nervous colitis | Cancer | Hypothyroidism | Hyperthyroidism | Rheumatoid arthritis | Chronic renal insufficiency | Heart disease | Depression | Other |
| If you selected the other option, specify which or which |  | | | | | | | | | | | | |
| Does anyone in your family suffer from any disease? | | | | | | | | | | | | | |
| Father |  |  |  |  |  |  |  |  |  |  |  |  |  |
| Mother |  |  |  |  |  |  |  |  |  |  |  |  |  |
| Brother |  |  |  |  |  |  |  |  |  |  |  |  |  |
| Sister |  |  |  |  |  |  |  |  |  |  |  |  |  |
| Paternal grandfather |  |  |  |  |  |  |  |  |  |  |  |  |  |
| Paternal grandmother |  |  |  |  |  |  |  |  |  |  |  |  |  |
| Maternal grandfather |  |  |  |  |  |  |  |  |  |  |  |  |  |
| Maternal grandmother |  |  |  |  |  |  |  |  |  |  |  |  |  |
| Paternal uncle |  |  |  |  |  |  |  |  |  |  |  |  |  |
| Paternal aunt |  |  |  |  |  |  |  |  |  |  |  |  |  |
| Maternal uncle |  |  |  |  |  |  |  |  |  |  |  |  |  |
| Maternal aunt |  |  |  |  |  |  |  |  |  |  |  |  |  |
| Other: |  | | | | | | | | | | | | |
| Do you currently take any medications and/or supplements? | | | | | | | | | | | | | |
| Medicines |  |  |  |  |  |  |  |  |  |  |  |  |  |
|  | Ampicillin | Bezafibrate | Cephalexin | Ciprofloxacin | Clarithromycin | Fluoxetine | Insulin | Levothyroxine | Losartan | Metformin | Omeprazole | Pravastatin | Telmisartan |
| Other. If you selected the other option, specify which or which |  | | | | | | | | | | | | |
| Supplements |  |  |  |  |  |  |  |  |  |  |  |  |  |
|  | Multivitamin | Vitamin D | Probiotics | Prebiotics | Vitamin C | Vitamin E | Iron | Calcium | Omega 3 | Protein powder | Creatine monohydrate | Other |  |

Table SM1.6.1 Continuation: Initial Assessment Questionnaire

| 24-hour recall | | | |
| --- | --- | --- | --- |
| Food time | Menu / Preparation | Ingredient / Food | Quantity |
| Breakfast |  |  |  |
| Hour: |  |  |  |
| Place: |  |  |  |
| Morning snack |  |  |  |
| Hour: |  |  |  |
| Place: |  |  |  |
| Meal |  |  |  |
| Hour: |  |  |  |
| Place: |  |  |  |
| Evening snack |  |  |  |
| Hour: |  |  |  |
| Place: |  |  |  |
| Dinner |  |  |  |
| Hour: |  |  |  |
| Place: |  |  |  |
| Milliliters of water consumed throughout the day: | | | |

Table SM1.6.1 Continuation: Initial Assessment Questionnaire

| **Food frequency questionnaire** | | | | | | | | | | | | | | | | | | | | | | | | | | | | |
| --- | --- | --- | --- | --- | --- | --- | --- | --- | --- | --- | --- | --- | --- | --- | --- | --- | --- | --- | --- | --- | --- | --- | --- | --- | --- | --- | --- | --- |
| For each food, indicate how many times per month or per week you consume it. Subsequently, indicate how many times a day you consume it, the day you take it. For example, if you drink 2 cups (480 milliliters) of whole milk 3 times a week and those 3 days you drink it in the morning and at night, you drink it 3 times a week and 2 times a day, in a quantity of 2 portions. It is also important to consider the summer/winter variation. For example, if you eat ice cream 4 times a week during the 3 summer months, your average consumption is 1 time a week. | | | | | | | | | | | | | | | | | | | | | | | | | | | | |
| **Group** | **Food** | **Portion** | Type, brand, flavor or preparation | **Average consumption last year**  ***Mark 1 option among these 3 frequencies.**  ***If you do not consume it, choose the option never and go to the next food.** | | | | | | | | | | | | | **When you consume it, how many times a day do you do it?**  **(Check 1 option per day)** | | | | **Number of servings** | | | | | | | |
|  |  |  |  | **Times a year** | | | **Times a month** | | | **Times a week** | | | | | | | **Times a day** | | | | **(Mark how many servings you consume each time you eat it)** | | | | | | | |
|  |  |  |  | Never | 1-5 | 6-11 | 1 | 2 | 3 | 1 | 2 | 3 | 4 | 5 | 6 | 7 | 1 | 2 | 3 | 4+ | 1/4 | 1/2 | 1 | 2 | 3 | 4 | 5 | 6+ |
| **Dairy** | 1. Whole milk | 1 cup (240 ml) |  |  |  |  |  |  |  |  |  |  |  |  |  |  |  |  |  |  |  |  |  |  |  |  |  |  |
|  | 2. Semi-skim milk | 1 cup (240 ml) |  |  |  |  |  |  |  |  |  |  |  |  |  |  |  |  |  |  |  |  |  |  |  |  |  |  |
|  | 3. Skim milk | 1 cup (240 ml) |  |  |  |  |  |  |  |  |  |  |  |  |  |  |  |  |  |  |  |  |  |  |  |  |  |  |
|  | 4. Almond milk | 1 cup (240 ml) |  |  |  |  |  |  |  |  |  |  |  |  |  |  |  |  |  |  |  |  |  |  |  |  |  |  |
|  | 5. Soy milk | 1 cup (240 ml) |  |  |  |  |  |  |  |  |  |  |  |  |  |  |  |  |  |  |  |  |  |  |  |  |  |  |
|  | 6. Coconut milk | 1 cup (240 ml) |  |  |  |  |  |  |  |  |  |  |  |  |  |  |  |  |  |  |  |  |  |  |  |  |  |  |
|  | 7. Chocolate flavored milk | 1 cup (240 ml) |  |  |  |  |  |  |  |  |  |  |  |  |  |  |  |  |  |  |  |  |  |  |  |  |  |  |
|  | 8. Packaged milk shake | 1 cup (240 ml) |  |  |  |  |  |  |  |  |  |  |  |  |  |  |  |  |  |  |  |  |  |  |  |  |  |  |
|  | 9. Whole yogurt (sweetened with fruit) | 1 cup (227 g) |  |  |  |  |  |  |  |  |  |  |  |  |  |  |  |  |  |  |  |  |  |  |  |  |  |  |
|  | 10. Low-fat yogurt | 1 cup (240 g) |  |  |  |  |  |  |  |  |  |  |  |  |  |  |  |  |  |  |  |  |  |  |  |  |  |  |
|  | 11. Fermented milk drink | 1 piece (80 g) |  |  |  |  |  |  |  |  |  |  |  |  |  |  |  |  |  |  |  |  |  |  |  |  |  |  |
|  | 12. *Bionico* cream (evaporated milk with cow cream) | ½ cup (120 g) |  |  |  |  |  |  |  |  |  |  |  |  |  |  |  |  |  |  |  |  |  |  |  |  |  |  |
| **Cheeses** | 13. Curd | 3 tablespoons (42 g) |  |  |  |  |  |  |  |  |  |  |  |  |  |  |  |  |  |  |  |  |  |  |  |  |  |  |
|  | 14. Cottage cheese | 3 tablespoons (48 g) |  |  |  |  |  |  |  |  |  |  |  |  |  |  |  |  |  |  |  |  |  |  |  |  |  |  |
|  | 15. Fresh cheese (not in quesadillas) | 1 slice (40 g) |  |  |  |  |  |  |  |  |  |  |  |  |  |  |  |  |  |  |  |  |  |  |  |  |  |  |
|  | 15.1 Panela cheese (not in quesadillas) | 1slice (40 g) |  |  |  |  |  |  |  |  |  |  |  |  |  |  |  |  |  |  |  |  |  |  |  |  |  |  |
|  | 16. Cured cheeses consumed alone (manchego, oaxaca, asadero, mozzarella, cheddar, goat, parmesan, chihuahua), (not in quesadillas) | 1 slice (30 g) |  |  |  |  |  |  |  |  |  |  |  |  |  |  |  |  |  |  |  |  |  |  |  |  |  |  |
| **Animal source foods** | 17. Chicken eggs | 1 piece (60 g) |  |  |  |  |  |  |  |  |  |  |  |  |  |  |  |  |  |  |  |  |  |  |  |  |  |  |
|  | 18. Egg white | 2 pieces (66 g) |  |  |  |  |  |  |  |  |  |  |  |  |  |  |  |  |  |  |  |  |  |  |  |  |  |  |
|  | 19. Chicken with skin (chicken pieces except wings) | 1 piece (200 g) |  |  |  |  |  |  |  |  |  |  |  |  |  |  |  |  |  |  |  |  |  |  |  |  |  |  |
|  | 20. Chicken without skin in pieces | 1 piece (200 g) |  |  |  |  |  |  |  |  |  |  |  |  |  |  |  |  |  |  |  |  |  |  |  |  |  |  |
|  | 20.1 Grilled skinless chicken fillet | 1 ration (90 g) |  |  |  |  |  |  |  |  |  |  |  |  |  |  |  |  |  |  |  |  |  |  |  |  |  |  |
|  | 21. Turkey | 1 ration (108 g) |  |  |  |  |  |  |  |  |  |  |  |  |  |  |  |  |  |  |  |  |  |  |  |  |  |  |
|  | 21.1 Bunny | 1 ration (90 g) |  |  |  |  |  |  |  |  |  |  |  |  |  |  |  |  |  |  |  |  |  |  |  |  |  |  |
|  | 22. Beef steak or steak (stews, griddle) | 1 ration (90 g) |  |  |  |  |  |  |  |  |  |  |  |  |  |  |  |  |  |  |  |  |  |  |  |  |  |  |
|  | 23. Ground beef (meatballs, hash) | 1 ration (99 g) |  |  |  |  |  |  |  |  |  |  |  |  |  |  |  |  |  |  |  |  |  |  |  |  |  |  |
|  | 24. Pork (stews, grilled) | 1 ration (120 g) |  |  |  |  |  |  |  |  |  |  |  |  |  |  |  |  |  |  |  |  |  |  |  |  |  |  |
|  | 25. Sheep meat (al pastor, stews) | 1 ration (111 g) |  |  |  |  |  |  |  |  |  |  |  |  |  |  |  |  |  |  |  |  |  |  |  |  |  |  |
|  | 26. Goat meat (birria) | 1 ration (100 g) |  |  |  |  |  |  |  |  |  |  |  |  |  |  |  |  |  |  |  |  |  |  |  |  |  |  |
|  | 27. Liver or organ meats of chicken, beef or pork (brains, gizzard, heart) | 1 ration (90 g) |  |  |  |  |  |  |  |  |  |  |  |  |  |  |  |  |  |  |  |  |  |  |  |  |  |  |
|  | 28. Turkey or pork ham (besides of used in sandwich) | 1 slice (21 g) |  |  |  |  |  |  |  |  |  |  |  |  |  |  |  |  |  |  |  |  |  |  |  |  |  |  |
|  | 29. Turkey or pork sausage (other than hot dog) | 1 piece (61 g) |  |  |  |  |  |  |  |  |  |  |  |  |  |  |  |  |  |  |  |  |  |  |  |  |  |  |
|  | 30. Processed meats (pepperoni, salami, sausage, chorizo, machaca) | ½ cup (20 g) |  |  |  |  |  |  |  |  |  |  |  |  |  |  |  |  |  |  |  |  |  |  |  |  |  |  |
|  | 31. Bacon | 1 slice (16 g) |  |  |  |  |  |  |  |  |  |  |  |  |  |  |  |  |  |  |  |  |  |  |  |  |  |  |
|  | 32. Pork rinds | ½ cup (24 g) |  |  |  |  |  |  |  |  |  |  |  |  |  |  |  |  |  |  |  |  |  |  |  |  |  |  |
|  | 33. White fish (tilapia, sole) | 1 ration (120 g) |  |  |  |  |  |  |  |  |  |  |  |  |  |  |  |  |  |  |  |  |  |  |  |  |  |  |
|  | 34. Blue fish (salmon, tuna, red snapper) | 1 ration (120 g) |  |  |  |  |  |  |  |  |  |  |  |  |  |  |  |  |  |  |  |  |  |  |  |  |  |  |
|  | 35. Salty fish (cod, dried *charales*) | 1 ration (90 g) |  |  |  |  |  |  |  |  |  |  |  |  |  |  |  |  |  |  |  |  |  |  |  |  |  |  |
|  | 36. Canned fish (tuna, sardine) or shellfish in water | ½ can (55g) |  |  |  |  |  |  |  |  |  |  |  |  |  |  |  |  |  |  |  |  |  |  |  |  |  |  |
|  | 37. Canned fish (tuna, sardine) or shellfish in oil | ½ can (55g) |  |  |  |  |  |  |  |  |  |  |  |  |  |  |  |  |  |  |  |  |  |  |  |  |  |  |
|  | 38. Shellfish (shrimp, prawn) | 10 piece (68 g) |  |  |  |  |  |  |  |  |  |  |  |  |  |  |  |  |  |  |  |  |  |  |  |  |  |  |
|  | 38.1 River prawn | 10 piece (68 g) |  |  |  |  |  |  |  |  |  |  |  |  |  |  |  |  |  |  |  |  |  |  |  |  |  |  |
|  | 39. Octopus or squid | 1 ration (100 g) |  |  |  |  |  |  |  |  |  |  |  |  |  |  |  |  |  |  |  |  |  |  |  |  |  |  |
|  | 40. Oysters, clams, mussels | 4 pieces (58 g) |  |  |  |  |  |  |  |  |  |  |  |  |  |  |  |  |  |  |  |  |  |  |  |  |  |  |
|  | 40.1 Insects (maguey worms, crickets) | 1 portion (35 g) |  |  |  |  |  |  |  |  |  |  |  |  |  |  |  |  |  |  |  |  |  |  |  |  |  |  |
| **Vegetables** | 41. Frozen vegetables | ½ cup (46 g) |  |  |  |  |  |  |  |  |  |  |  |  |  |  |  |  |  |  |  |  |  |  |  |  |  |  |
|  | 42. Spinach, chard or purslane | 1/2 cup (30 g) |  |  |  |  |  |  |  |  |  |  |  |  |  |  |  |  |  |  |  |  |  |  |  |  |  |  |
|  | 43. Broccoli or cauliflower | ½ cup (108 g) |  |  |  |  |  |  |  |  |  |  |  |  |  |  |  |  |  |  |  |  |  |  |  |  |  |  |
|  | 44. Lettuce, cabbage, cabbage or kale | ½ cup (25 g) |  |  |  |  |  |  |  |  |  |  |  |  |  |  |  |  |  |  |  |  |  |  |  |  |  |  |
|  | 45. Red tomato raw or in sauce | 1 piece (60 g) |  |  |  |  |  |  |  |  |  |  |  |  |  |  |  |  |  |  |  |  |  |  |  |  |  |  |
|  | 46. Carrot | ½ piece (64g) |  |  |  |  |  |  |  |  |  |  |  |  |  |  |  |  |  |  |  |  |  |  |  |  |  |  |
|  | 47. Zucchini | 1 piece (91 g) |  |  |  |  |  |  |  |  |  |  |  |  |  |  |  |  |  |  |  |  |  |  |  |  |  |  |
|  | 48. Pumpkin | ½ cup (110 g) |  |  |  |  |  |  |  |  |  |  |  |  |  |  |  |  |  |  |  |  |  |  |  |  |  |  |
|  | 49. Green beans or peas | ½ cup (63 g) |  |  |  |  |  |  |  |  |  |  |  |  |  |  |  |  |  |  |  |  |  |  |  |  |  |  |
|  | 50. Cucumber | 1 piece (104 g) |  |  |  |  |  |  |  |  |  |  |  |  |  |  |  |  |  |  |  |  |  |  |  |  |  |  |
|  | 51. Celery | ½ cup (45 g) |  |  |  |  |  |  |  |  |  |  |  |  |  |  |  |  |  |  |  |  |  |  |  |  |  |  |
|  | 52. Beetroot | ¼ piece (39 g) |  |  |  |  |  |  |  |  |  |  |  |  |  |  |  |  |  |  |  |  |  |  |  |  |  |  |
|  | 53. Eggplant | ½ cup (50 g) |  |  |  |  |  |  |  |  |  |  |  |  |  |  |  |  |  |  |  |  |  |  |  |  |  |  |
|  | 54. Asparagus | 3 piece (45 g) |  |  |  |  |  |  |  |  |  |  |  |  |  |  |  |  |  |  |  |  |  |  |  |  |  |  |
|  | 55. Onion | ¼ cup (29 g) |  |  |  |  |  |  |  |  |  |  |  |  |  |  |  |  |  |  |  |  |  |  |  |  |  |  |
|  | 56. Green tomato, raw or in sauce | 4 pieces (69 g) |  |  |  |  |  |  |  |  |  |  |  |  |  |  |  |  |  |  |  |  |  |  |  |  |  |  |
|  | 57. Mushrooms | ½ cup (70 g) |  |  |  |  |  |  |  |  |  |  |  |  |  |  |  |  |  |  |  |  |  |  |  |  |  |  |
|  | 57.1 Huitlacoche | 1/3 cup (66 g) |  |  |  |  |  |  |  |  |  |  |  |  |  |  |  |  |  |  |  |  |  |  |  |  |  |  |
|  | 58. *Chayote* | ½ cup (80 g) |  |  |  |  |  |  |  |  |  |  |  |  |  |  |  |  |  |  |  |  |  |  |  |  |  |  |
|  | 59. Jicama | ½ cup (60 g) |  |  |  |  |  |  |  |  |  |  |  |  |  |  |  |  |  |  |  |  |  |  |  |  |  |  |
|  | 60. Cooked, raw or roasted *nopales* (cactus) | ½ cup (75g) |  |  |  |  |  |  |  |  |  |  |  |  |  |  |  |  |  |  |  |  |  |  |  |  |  |  |
|  | 61. Bell pepper | 1/2 piece (32 g) |  |  |  |  |  |  |  |  |  |  |  |  |  |  |  |  |  |  |  |  |  |  |  |  |  |  |
|  | 62. Poblano Chile | 1/2 piece (32 g) |  |  |  |  |  |  |  |  |  |  |  |  |  |  |  |  |  |  |  |  |  |  |  |  |  |  |
|  | 62.1 Quelites | ½ cup (66 g) |  |  |  |  |  |  |  |  |  |  |  |  |  |  |  |  |  |  |  |  |  |  |  |  |  |  |
|  | 62.2 Purslane | 1 cup (115 g) |  |  |  |  |  |  |  |  |  |  |  |  |  |  |  |  |  |  |  |  |  |  |  |  |  |  |
|  | 63. Hot peppers (jalapeño, serrano, habanero) | 1 piece (30 g) |  |  |  |  |  |  |  |  |  |  |  |  |  |  |  |  |  |  |  |  |  |  |  |  |  |  |
|  | 64. Garlic | ¼ piece (1 g) |  |  |  |  |  |  |  |  |  |  |  |  |  |  |  |  |  |  |  |  |  |  |  |  |  |  |
|  | 65. Coriander, parsley, oregano, basil, bay leaf | 1 pinch (6 g) |  |  |  |  |  |  |  |  |  |  |  |  |  |  |  |  |  |  |  |  |  |  |  |  |  |  |
| **Fruits** | 66. Orange (not in juice) | 1 piece (76 g) |  |  |  |  |  |  |  |  |  |  |  |  |  |  |  |  |  |  |  |  |  |  |  |  |  |  |
|  | 67. Grapefruit (not in juice) | 1 piece (76 g) |  |  |  |  |  |  |  |  |  |  |  |  |  |  |  |  |  |  |  |  |  |  |  |  |  |  |
|  | 68. Tangerines (not in juice) | 1 piece (76 g) |  |  |  |  |  |  |  |  |  |  |  |  |  |  |  |  |  |  |  |  |  |  |  |  |  |  |
|  | 69. Lemon | 1 piece (38 g) |  |  |  |  |  |  |  |  |  |  |  |  |  |  |  |  |  |  |  |  |  |  |  |  |  |  |
|  | 70. Lime (not in juice) | 1 piece (49 g) |  |  |  |  |  |  |  |  |  |  |  |  |  |  |  |  |  |  |  |  |  |  |  |  |  |  |
|  | 71. Banana (not fried) | 1 piece (108 g) |  |  |  |  |  |  |  |  |  |  |  |  |  |  |  |  |  |  |  |  |  |  |  |  |  |  |
|  | 72. Apple | 1 piece (106 g) |  |  |  |  |  |  |  |  |  |  |  |  |  |  |  |  |  |  |  |  |  |  |  |  |  |  |
|  | 73. Pear | 1 piece (162 g) |  |  |  |  |  |  |  |  |  |  |  |  |  |  |  |  |  |  |  |  |  |  |  |  |  |  |
|  | 74. Strawberries | 6 pieces (68 g) |  |  |  |  |  |  |  |  |  |  |  |  |  |  |  |  |  |  |  |  |  |  |  |  |  |  |
|  | 75. Blueberry | ½ cup (49 g) |  |  |  |  |  |  |  |  |  |  |  |  |  |  |  |  |  |  |  |  |  |  |  |  |  |  |
|  | 76. Raspberry | ½ cup (61 g) |  |  |  |  |  |  |  |  |  |  |  |  |  |  |  |  |  |  |  |  |  |  |  |  |  |  |
|  | 77. Blackberry | ½ cup (72 g) |  |  |  |  |  |  |  |  |  |  |  |  |  |  |  |  |  |  |  |  |  |  |  |  |  |  |
|  | 78. Creole plum | 2 pieces (105 g) |  |  |  |  |  |  |  |  |  |  |  |  |  |  |  |  |  |  |  |  |  |  |  |  |  |  |
|  | 78.1 Capulín | 1 cup (32 g) |  |  |  |  |  |  |  |  |  |  |  |  |  |  |  |  |  |  |  |  |  |  |  |  |  |  |
|  | 78.2 Nance | 5 pieces (15 g) |  |  |  |  |  |  |  |  |  |  |  |  |  |  |  |  |  |  |  |  |  |  |  |  |  |  |
|  | 79. Cherries | 4 pieces (18 g) |  |  |  |  |  |  |  |  |  |  |  |  |  |  |  |  |  |  |  |  |  |  |  |  |  |  |
|  | 80. Peach | 1 piece (77 g) |  |  |  |  |  |  |  |  |  |  |  |  |  |  |  |  |  |  |  |  |  |  |  |  |  |  |
|  | 81. Nectarine | 1 piece (124 g) |  |  |  |  |  |  |  |  |  |  |  |  |  |  |  |  |  |  |  |  |  |  |  |  |  |  |
|  | 82. Watermelon | 1 cup (160 g) |  |  |  |  |  |  |  |  |  |  |  |  |  |  |  |  |  |  |  |  |  |  |  |  |  |  |
|  | 83. Cantaloupe | 1 cup (160 g) |  |  |  |  |  |  |  |  |  |  |  |  |  |  |  |  |  |  |  |  |  |  |  |  |  |  |
|  | 84. Papaya | 1 cup (140 g) |  |  |  |  |  |  |  |  |  |  |  |  |  |  |  |  |  |  |  |  |  |  |  |  |  |  |
|  | 85. Kiwi | 1 piece (76 g) |  |  |  |  |  |  |  |  |  |  |  |  |  |  |  |  |  |  |  |  |  |  |  |  |  |  |
|  | 86. Grapes | 18 pieces (86 g) |  |  |  |  |  |  |  |  |  |  |  |  |  |  |  |  |  |  |  |  |  |  |  |  |  |  |
|  | 87. Mango | ½ piece (110 g) |  |  |  |  |  |  |  |  |  |  |  |  |  |  |  |  |  |  |  |  |  |  |  |  |  |  |
|  | 88. Guava | 2 pieces (83 g) |  |  |  |  |  |  |  |  |  |  |  |  |  |  |  |  |  |  |  |  |  |  |  |  |  |  |
|  | 89. Tuna | 1 piece (70 g) |  |  |  |  |  |  |  |  |  |  |  |  |  |  |  |  |  |  |  |  |  |  |  |  |  |  |
|  | 90. Pitaya | 1 piece (50 g) |  |  |  |  |  |  |  |  |  |  |  |  |  |  |  |  |  |  |  |  |  |  |  |  |  |  |
|  | 91. Guanabana | ½ piece (175 g) |  |  |  |  |  |  |  |  |  |  |  |  |  |  |  |  |  |  |  |  |  |  |  |  |  |  |
|  | 92. Pineapple | 1 cup (165 g) |  |  |  |  |  |  |  |  |  |  |  |  |  |  |  |  |  |  |  |  |  |  |  |  |  |  |
|  | 93. Canned fruit in syrup | ½ cup (124 g) |  |  |  |  |  |  |  |  |  |  |  |  |  |  |  |  |  |  |  |  |  |  |  |  |  |  |
|  | 94. Dehydrated fruit (blueberries, raisins, dates, prune) | 10 pieces (20 g) |  |  |  |  |  |  |  |  |  |  |  |  |  |  |  |  |  |  |  |  |  |  |  |  |  |  |
| **Protein oils** | 95. Almonds | 10 pieces (12 g) |  |  |  |  |  |  |  |  |  |  |  |  |  |  |  |  |  |  |  |  |  |  |  |  |  |  |
|  | 96. Raw peanuts | 14 pieces (12 g) |  |  |  |  |  |  |  |  |  |  |  |  |  |  |  |  |  |  |  |  |  |  |  |  |  |  |
|  | 96.1 Peanuts (Japanese, enchiladas, with salt) | 14 pieces (12 g) |  |  |  |  |  |  |  |  |  |  |  |  |  |  |  |  |  |  |  |  |  |  |  |  |  |  |
|  | 97. Nuts | 7 halvez (10 g) |  |  |  |  |  |  |  |  |  |  |  |  |  |  |  |  |  |  |  |  |  |  |  |  |  |  |
|  | 98. Pistachios, hazelnuts, pine nuts | 18 pieces (13 g) |  |  |  |  |  |  |  |  |  |  |  |  |  |  |  |  |  |  |  |  |  |  |  |  |  |  |
|  | 98.1 Dried beans | 18 pieces (13 g) |  |  |  |  |  |  |  |  |  |  |  |  |  |  |  |  |  |  |  |  |  |  |  |  |  |  |
|  | 99. Sunflower or hemp seeds | 1 tablespoon (10 g) |  |  |  |  |  |  |  |  |  |  |  |  |  |  |  |  |  |  |  |  |  |  |  |  |  |  |
|  | 99.1 Pumpkin seeds | 1 tablespoon (10 g) |  |  |  |  |  |  |  |  |  |  |  |  |  |  |  |  |  |  |  |  |  |  |  |  |  |  |
|  | 100. Chia seeds | 1 teaspoon (3 g) |  |  |  |  |  |  |  |  |  |  |  |  |  |  |  |  |  |  |  |  |  |  |  |  |  |  |
|  | 100.1 Flax | 1 teaspoon (3 g) |  |  |  |  |  |  |  |  |  |  |  |  |  |  |  |  |  |  |  |  |  |  |  |  |  |  |
|  | 101. Peanut or almond butter | 1 tablespoon (15 g) |  |  |  |  |  |  |  |  |  |  |  |  |  |  |  |  |  |  |  |  |  |  |  |  |  |  |
| **Legumes** | 102. Cooked beans | ½ cup (99 g) |  |  |  |  |  |  |  |  |  |  |  |  |  |  |  |  |  |  |  |  |  |  |  |  |  |  |
|  | 103. Refried beans | ½ cup (99 g) |  |  |  |  |  |  |  |  |  |  |  |  |  |  |  |  |  |  |  |  |  |  |  |  |  |  |
|  | 104. Cooked lentils | ½ cup (99 g) |  |  |  |  |  |  |  |  |  |  |  |  |  |  |  |  |  |  |  |  |  |  |  |  |  |  |
|  | 105. Cooked chickpeas | ½ cup (82 g) |  |  |  |  |  |  |  |  |  |  |  |  |  |  |  |  |  |  |  |  |  |  |  |  |  |  |
|  | 105.1 Baked beans | ½ cup (99 g) |  |  |  |  |  |  |  |  |  |  |  |  |  |  |  |  |  |  |  |  |  |  |  |  |  |  |
|  | 106. Chickpea hummus | 4 tablespoon (60 g) |  |  |  |  |  |  |  |  |  |  |  |  |  |  |  |  |  |  |  |  |  |  |  |  |  |  |
|  | 107. Textured soy | 1/2 cup (30 g) |  |  |  |  |  |  |  |  |  |  |  |  |  |  |  |  |  |  |  |  |  |  |  |  |  |  |
| **Fat-free cereal** | 108. Corn tortilla | 1 piece (30 g) |  |  |  |  |  |  |  |  |  |  |  |  |  |  |  |  |  |  |  |  |  |  |  |  |  |  |
|  | 10.8.1 Corn dough | 1 ball (45 g) |  |  |  |  |  |  |  |  |  |  |  |  |  |  |  |  |  |  |  |  |  |  |  |  |  |  |
|  | 109. Corn | 1 piece (116 g) |  |  |  |  |  |  |  |  |  |  |  |  |  |  |  |  |  |  |  |  |  |  |  |  |  |  |
|  | 110. Baked or boiled potato | 1 piece (136 g) |  |  |  |  |  |  |  |  |  |  |  |  |  |  |  |  |  |  |  |  |  |  |  |  |  |  |
|  | 111. Flour tortilla | 1 piece (28 g) |  |  |  |  |  |  |  |  |  |  |  |  |  |  |  |  |  |  |  |  |  |  |  |  |  |  |
|  | 112. Wholemeal flour tortilla | 1 piece (28 g) |  |  |  |  |  |  |  |  |  |  |  |  |  |  |  |  |  |  |  |  |  |  |  |  |  |  |
|  | 113. Bolillo, birote or telera (not in cake) | ½ piece (30 g) |  |  |  |  |  |  |  |  |  |  |  |  |  |  |  |  |  |  |  |  |  |  |  |  |  |  |
|  | 114. White bread or toast white bread (apart from sandwich) | 1 slice (27 g) |  |  |  |  |  |  |  |  |  |  |  |  |  |  |  |  |  |  |  |  |  |  |  |  |  |  |
|  | 115. Whole wheat bread or toast whole wheat bread (apart from sandwich) | 1 slice (25 g) |  |  |  |  |  |  |  |  |  |  |  |  |  |  |  |  |  |  |  |  |  |  |  |  |  |  |
|  | 116. Unsweetened box cereal | 1 cup (25 g) |  |  |  |  |  |  |  |  |  |  |  |  |  |  |  |  |  |  |  |  |  |  |  |  |  |  |
|  | 117. Sweetened box cereal | 1 cup (25 g) |  |  |  |  |  |  |  |  |  |  |  |  |  |  |  |  |  |  |  |  |  |  |  |  |  |  |
|  | 118. Whole wheat box cereal | 1 cup (25 g) |  |  |  |  |  |  |  |  |  |  |  |  |  |  |  |  |  |  |  |  |  |  |  |  |  |  |
|  | 119. Pasta (noodles, macaroni, spaghetti) | ½ cup (60 g) |  |  |  |  |  |  |  |  |  |  |  |  |  |  |  |  |  |  |  |  |  |  |  |  |  |  |
|  | 120. Oatmeal (flaked, cooked in water) | ½ cup (20 g) |  |  |  |  |  |  |  |  |  |  |  |  |  |  |  |  |  |  |  |  |  |  |  |  |  |  |
|  | 121. Cooked oatmeal with milk and sugar (drink or solid) | 1 cup (240 ml) |  |  |  |  |  |  |  |  |  |  |  |  |  |  |  |  |  |  |  |  |  |  |  |  |  |  |
|  | 122. Cooked white rice or Mexican cooked white rice | ½ cup (94 g) |  |  |  |  |  |  |  |  |  |  |  |  |  |  |  |  |  |  |  |  |  |  |  |  |  |  |
|  | 123. Brown or wild rice | ½ cup (94 g) |  |  |  |  |  |  |  |  |  |  |  |  |  |  |  |  |  |  |  |  |  |  |  |  |  |  |
|  | 124. Natural puffed rice or puffed rice cake | ½ cup (17 g) |  |  |  |  |  |  |  |  |  |  |  |  |  |  |  |  |  |  |  |  |  |  |  |  |  |  |
|  | 125. Unsweetened amaranth | ½ cup (32 g) |  |  |  |  |  |  |  |  |  |  |  |  |  |  |  |  |  |  |  |  |  |  |  |  |  |  |
|  | 125.1 Sweetened amaranth or amaranth bar | ½ cup (32 g) |  |  |  |  |  |  |  |  |  |  |  |  |  |  |  |  |  |  |  |  |  |  |  |  |  |  |
|  | 126. Cooked sweet potato | ½ cup (103 g) |  |  |  |  |  |  |  |  |  |  |  |  |  |  |  |  |  |  |  |  |  |  |  |  |  |  |
|  | 127. Sweet potato cooked with sugar | ½ cup (103 g) |  |  |  |  |  |  |  |  |  |  |  |  |  |  |  |  |  |  |  |  |  |  |  |  |  |  |
|  | 128. Quinoa | ½ cup (40 g) |  |  |  |  |  |  |  |  |  |  |  |  |  |  |  |  |  |  |  |  |  |  |  |  |  |  |
|  | 129. Marías or animals shape biscuits | 5 pieces (19 g) |  |  |  |  |  |  |  |  |  |  |  |  |  |  |  |  |  |  |  |  |  |  |  |  |  |  |
|  | 130. Whole meal or fibre biscuits (sweet or salty type) | 1 sweet or 9 salty (45 g) |  |  |  |  |  |  |  |  |  |  |  |  |  |  |  |  |  |  |  |  |  |  |  |  |  |  |
|  | 131. Salty biscuits | 4 pieces (16 g) |  |  |  |  |  |  |  |  |  |  |  |  |  |  |  |  |  |  |  |  |  |  |  |  |  |  |
|  | 132. Baked Maize toast | 1 piece (10 g) |  |  |  |  |  |  |  |  |  |  |  |  |  |  |  |  |  |  |  |  |  |  |  |  |  |  |
| **Fatty cereals**  **Leguminosas**  **Cereales sin grasa** | 133. Fried Maize toast | 1 piece (12 g) |  |  |  |  |  |  |  |  |  |  |  |  |  |  |  |  |  |  |  |  |  |  |  |  |  |  |
|  | 134. Packaged sweet biscuits | 2 piece (21 g) |  |  |  |  |  |  |  |  |  |  |  |  |  |  |  |  |  |  |  |  |  |  |  |  |  |  |
|  | 135. Granola bar | 1 piece (35g) |  |  |  |  |  |  |  |  |  |  |  |  |  |  |  |  |  |  |  |  |  |  |  |  |  |  |
|  | 136. Granola with nuts | 3 tablespoons (21 g) |  |  |  |  |  |  |  |  |  |  |  |  |  |  |  |  |  |  |  |  |  |  |  |  |  |  |
|  | 137. Hot cake or waffle | 1 piece (50 g) |  |  |  |  |  |  |  |  |  |  |  |  |  |  |  |  |  |  |  |  |  |  |  |  |  |  |
|  | 138. Industrialized bread (donuts, shortcakes) | 1 piece (30 g) |  |  |  |  |  |  |  |  |  |  |  |  |  |  |  |  |  |  |  |  |  |  |  |  |  |  |
|  | 139. Industrialized bread with chocolate | 1 piece (50 g) |  |  |  |  |  |  |  |  |  |  |  |  |  |  |  |  |  |  |  |  |  |  |  |  |  |  |
|  | 140. Sweet bread (*conchas, orejas, polvorón*) | 1 piece (63 g) |  |  |  |  |  |  |  |  |  |  |  |  |  |  |  |  |  |  |  |  |  |  |  |  |  |  |
|  | 141. Cake or pay (thick slice) | 1 slice (56 g) |  |  |  |  |  |  |  |  |  |  |  |  |  |  |  |  |  |  |  |  |  |  |  |  |  |  |
|  | 142. Homemade breads or *rosca de reyes* | 1 slice (56 g) |  |  |  |  |  |  |  |  |  |  |  |  |  |  |  |  |  |  |  |  |  |  |  |  |  |  |
|  | 143. Packaged chips | 1 bag (55 g) |  |  |  |  |  |  |  |  |  |  |  |  |  |  |  |  |  |  |  |  |  |  |  |  |  |  |
|  | 144. Packaged Maize snack | 1 bag (58 g) |  |  |  |  |  |  |  |  |  |  |  |  |  |  |  |  |  |  |  |  |  |  |  |  |  |  |
|  | 145. French fries or home fries | 4 pieces (20 g) |  |  |  |  |  |  |  |  |  |  |  |  |  |  |  |  |  |  |  |  |  |  |  |  |  |  |
|  | 146. Wheat churritos | 1 bag (50 g) |  |  |  |  |  |  |  |  |  |  |  |  |  |  |  |  |  |  |  |  |  |  |  |  |  |  |
|  | 147. Popcorn (homemade, microwave, cinema) | ½ bag (40 g) |  |  |  |  |  |  |  |  |  |  |  |  |  |  |  |  |  |  |  |  |  |  |  |  |  |  |
| **Protein oils** | 148. Oil spray | 5 sprays (5 g) |  |  |  |  |  |  |  |  |  |  |  |  |  |  |  |  |  |  |  |  |  |  |  |  |  |  |
|  | 149. Olive oil | 1 teaspoonful (5 g) |  |  |  |  |  |  |  |  |  |  |  |  |  |  |  |  |  |  |  |  |  |  |  |  |  |  |
|  | 150. Avocado oil | 1 teaspoonful (5 g) |  |  |  |  |  |  |  |  |  |  |  |  |  |  |  |  |  |  |  |  |  |  |  |  |  |  |
|  | 151. Maize oil | 1 teaspoonful (5 g) |  |  |  |  |  |  |  |  |  |  |  |  |  |  |  |  |  |  |  |  |  |  |  |  |  |  |
|  | 152. Coconut oil | 1 teaspoonful (5 g) |  |  |  |  |  |  |  |  |  |  |  |  |  |  |  |  |  |  |  |  |  |  |  |  |  |  |
|  | 153. Sunflower oil | 1 teaspoonful (5 g) |  |  |  |  |  |  |  |  |  |  |  |  |  |  |  |  |  |  |  |  |  |  |  |  |  |  |
|  | 154. Soy oil | 1 teaspoonful (5 g) |  |  |  |  |  |  |  |  |  |  |  |  |  |  |  |  |  |  |  |  |  |  |  |  |  |  |
|  | 155. Canola oil | 1 teaspoonful (5 g) |  |  |  |  |  |  |  |  |  |  |  |  |  |  |  |  |  |  |  |  |  |  |  |  |  |  |
|  | 156. Safflower oil | 1 teaspoonful (5 g) |  |  |  |  |  |  |  |  |  |  |  |  |  |  |  |  |  |  |  |  |  |  |  |  |  |  |
|  | 157. Olives | 8 pieces (24 g) |  |  |  |  |  |  |  |  |  |  |  |  |  |  |  |  |  |  |  |  |  |  |  |  |  |  |
|  | 158. Chopped coconut | 1 strip (16 g) |  |  |  |  |  |  |  |  |  |  |  |  |  |  |  |  |  |  |  |  |  |  |  |  |  |  |
|  | 159. Avocado or *guacamole* | ½ pieces (46 g) |  |  |  |  |  |  |  |  |  |  |  |  |  |  |  |  |  |  |  |  |  |  |  |  |  |  |
|  | 160. Mayonnaise, dressing or vinaigrette | 1 teaspoonful (5 g) |  |  |  |  |  |  |  |  |  |  |  |  |  |  |  |  |  |  |  |  |  |  |  |  |  |  |
|  | 161. Margarine | 1 teaspoonful (5 g) |  |  |  |  |  |  |  |  |  |  |  |  |  |  |  |  |  |  |  |  |  |  |  |  |  |  |
|  | 162. Butter | 1 teaspoonful (5 g) |  |  |  |  |  |  |  |  |  |  |  |  |  |  |  |  |  |  |  |  |  |  |  |  |  |  |
|  | 163. Lard | 1 teaspoonful (5 g) |  |  |  |  |  |  |  |  |  |  |  |  |  |  |  |  |  |  |  |  |  |  |  |  |  |  |
|  | 164. Vegetable shortening | 1 teaspoonful (5 g) |  |  |  |  |  |  |  |  |  |  |  |  |  |  |  |  |  |  |  |  |  |  |  |  |  |  |
|  | 165. Cream cheese | 1 tablespoon (15 g) |  |  |  |  |  |  |  |  |  |  |  |  |  |  |  |  |  |  |  |  |  |  |  |  |  |  |
|  | 166. Cream, nata or jocoque | 1 tablespoon (15 g) |  |  |  |  |  |  |  |  |  |  |  |  |  |  |  |  |  |  |  |  |  |  |  |  |  |  |
| **Sugars with and without fat** | 167. Sugar | 1 teaspoonful (4 g) |  |  |  |  |  |  |  |  |  |  |  |  |  |  |  |  |  |  |  |  |  |  |  |  |  |  |
|  | 168. Honey | 1 teaspoonful (7 g) |  |  |  |  |  |  |  |  |  |  |  |  |  |  |  |  |  |  |  |  |  |  |  |  |  |  |
|  | 169. Maple syrup | 1 teaspoonful (7 g) |  |  |  |  |  |  |  |  |  |  |  |  |  |  |  |  |  |  |  |  |  |  |  |  |  |  |
|  | 170. *Cajeta* | 1 tablespoon (15 g) |  |  |  |  |  |  |  |  |  |  |  |  |  |  |  |  |  |  |  |  |  |  |  |  |  |  |
|  | 171. Hazelnut cream | 1 tablespoon (15 g) |  |  |  |  |  |  |  |  |  |  |  |  |  |  |  |  |  |  |  |  |  |  |  |  |  |  |
|  | 172. Condensed milk | 1 tablespoon (15 g) |  |  |  |  |  |  |  |  |  |  |  |  |  |  |  |  |  |  |  |  |  |  |  |  |  |  |
|  | 173. Piloncillo (brown sugar) | 1 portion (10 g) |  |  |  |  |  |  |  |  |  |  |  |  |  |  |  |  |  |  |  |  |  |  |  |  |  |  |
|  | 174. Jams | 1 tablespoon (17 g) |  |  |  |  |  |  |  |  |  |  |  |  |  |  |  |  |  |  |  |  |  |  |  |  |  |  |
|  | 175. Packaged candy (i.e. popsicles, solid sweets, jelly beans) | 1 - 5 pieces (20 g) |  |  |  |  |  |  |  |  |  |  |  |  |  |  |  |  |  |  |  |  |  |  |  |  |  |  |
|  | 176. Chocolates | 1 bar (15 g) |  |  |  |  |  |  |  |  |  |  |  |  |  |  |  |  |  |  |  |  |  |  |  |  |  |  |
|  | 177. Cocoa powder for chocolate milk | 1 tablespoon (15 g) |  |  |  |  |  |  |  |  |  |  |  |  |  |  |  |  |  |  |  |  |  |  |  |  |  |  |
|  | 178. Ice cream or ice cream popsicle | 1 scoop/piece (96 g) |  |  |  |  |  |  |  |  |  |  |  |  |  |  |  |  |  |  |  |  |  |  |  |  |  |  |
|  | 179. Water-based ice cream or ice cold water palette | 1 scoop/piece (65 g) |  |  |  |  |  |  |  |  |  |  |  |  |  |  |  |  |  |  |  |  |  |  |  |  |  |  |
|  | 180. Tapioca (cooked in caramel or milk) | ½ cup (147 g) |  |  |  |  |  |  |  |  |  |  |  |  |  |  |  |  |  |  |  |  |  |  |  |  |  |  |
|  | 181. Gelatin in water or milk | 1 piece (125 ml) |  |  |  |  |  |  |  |  |  |  |  |  |  |  |  |  |  |  |  |  |  |  |  |  |  |  |
|  | 182. Mexican candy (*Ate, cocada, marzipan, palanqueta* (peanut or walnut), milk candy) | 1 piece (28 g) |  |  |  |  |  |  |  |  |  |  |  |  |  |  |  |  |  |  |  |  |  |  |  |  |  |  |
|  | 183. Fried or honeyed bananas | 1 piece (123.5 g) |  |  |  |  |  |  |  |  |  |  |  |  |  |  |  |  |  |  |  |  |  |  |  |  |  |  |
| **Fast food** | 184. Pizza | 1 slice (100 g) |  |  |  |  |  |  |  |  |  |  |  |  |  |  |  |  |  |  |  |  |  |  |  |  |  |  |
|  | 185. Burger | 1 piece (235 g) |  |  |  |  |  |  |  |  |  |  |  |  |  |  |  |  |  |  |  |  |  |  |  |  |  |  |
|  | 186. Hot dog | 1 piece (156 g) |  |  |  |  |  |  |  |  |  |  |  |  |  |  |  |  |  |  |  |  |  |  |  |  |  |  |
|  | 187. Ham sandwich | 1 piece (128 g) |  |  |  |  |  |  |  |  |  |  |  |  |  |  |  |  |  |  |  |  |  |  |  |  |  |  |
|  | 188. Instant soup | 1 piece (64 g) |  |  |  |  |  |  |  |  |  |  |  |  |  |  |  |  |  |  |  |  |  |  |  |  |  |  |
|  | 189. Sushi | 4 rolls (75 g) |  |  |  |  |  |  |  |  |  |  |  |  |  |  |  |  |  |  |  |  |  |  |  |  |  |  |
|  | 190. Chicken wings | 1 piece (51 g) |  |  |  |  |  |  |  |  |  |  |  |  |  |  |  |  |  |  |  |  |  |  |  |  |  |  |
|  | 191. Baguette or panini | 1 piece (208 g) |  |  |  |  |  |  |  |  |  |  |  |  |  |  |  |  |  |  |  |  |  |  |  |  |  |  |
|  | 192. Envelope soups and creams | 1 plate (100 g) |  |  |  |  |  |  |  |  |  |  |  |  |  |  |  |  |  |  |  |  |  |  |  |  |  |  |
| **Mexican food** | 193. Chilaquiles with beef | 1 cup (160 g) |  |  |  |  |  |  |  |  |  |  |  |  |  |  |  |  |  |  |  |  |  |  |  |  |  |  |
|  | 193. Chilaquiles with egg and/or chicken but without beef | 1 cup (160 g) |  |  |  |  |  |  |  |  |  |  |  |  |  |  |  |  |  |  |  |  |  |  |  |  |  |  |
|  | 193.1 Mexican style egg (tomato, onion, chili, nopal) | 1 plate (220 g) |  |  |  |  |  |  |  |  |  |  |  |  |  |  |  |  |  |  |  |  |  |  |  |  |  |  |
|  | 194. Offal | 1 plate (300 g) |  |  |  |  |  |  |  |  |  |  |  |  |  |  |  |  |  |  |  |  |  |  |  |  |  |  |
|  | 195. Corn tortilla quesadillas without meats | 1 piece (60 g) |  |  |  |  |  |  |  |  |  |  |  |  |  |  |  |  |  |  |  |  |  |  |  |  |  |  |
|  | 195.1 Corn tortilla quesadillas with meats | 1 piece (60 g) |  |  |  |  |  |  |  |  |  |  |  |  |  |  |  |  |  |  |  |  |  |  |  |  |  |  |
|  | 196. Flour tortilla quesadillas | 1 piece (60 g) |  |  |  |  |  |  |  |  |  |  |  |  |  |  |  |  |  |  |  |  |  |  |  |  |  |  |
|  | 197. Torta (marinated, meat, drowned) | 1 piece (197 g) |  |  |  |  |  |  |  |  |  |  |  |  |  |  |  |  |  |  |  |  |  |  |  |  |  |  |
|  | 197.1 Panela cake or beans (not meat) | 1 piece (197 g) |  |  |  |  |  |  |  |  |  |  |  |  |  |  |  |  |  |  |  |  |  |  |  |  |  |  |
|  | 198. Meat tamale (beef, pork, chicken) | 1 piece (200 g) |  |  |  |  |  |  |  |  |  |  |  |  |  |  |  |  |  |  |  |  |  |  |  |  |  |  |
|  | 198.1 Chicken tamale | 1 piece (200 g) |  |  |  |  |  |  |  |  |  |  |  |  |  |  |  |  |  |  |  |  |  |  |  |  |  |  |
|  | 198.2 Cheese tamale | 1 piece (200 g) |  |  |  |  |  |  |  |  |  |  |  |  |  |  |  |  |  |  |  |  |  |  |  |  |  |  |
|  | 198.3 Tamale of rajas or ash (beans) | 1 piece (200 g) |  |  |  |  |  |  |  |  |  |  |  |  |  |  |  |  |  |  |  |  |  |  |  |  |  |  |
|  | 199. Sweet tamale (corn, fruits) | 1 piece (172 g) |  |  |  |  |  |  |  |  |  |  |  |  |  |  |  |  |  |  |  |  |  |  |  |  |  |  |
|  | 200. Mole with chicken | 1 plate (334 g) |  |  |  |  |  |  |  |  |  |  |  |  |  |  |  |  |  |  |  |  |  |  |  |  |  |  |
|  | 201. Pork pozole | 1 plate (300 g) |  |  |  |  |  |  |  |  |  |  |  |  |  |  |  |  |  |  |  |  |  |  |  |  |  |  |
|  | 201.1 Chicken pozole | 1 plate (300 g) |  |  |  |  |  |  |  |  |  |  |  |  |  |  |  |  |  |  |  |  |  |  |  |  |  |  |
|  | 201.2 Vegetarian pozole (mushrooms) | 1 plate (300 g) |  |  |  |  |  |  |  |  |  |  |  |  |  |  |  |  |  |  |  |  |  |  |  |  |  |  |
|  | 202. Sopitos with beef or pork | 1 piece (75 g) |  |  |  |  |  |  |  |  |  |  |  |  |  |  |  |  |  |  |  |  |  |  |  |  |  |  |
|  | 202.1 Sopitos of beans or chicken (no red meat) | 1 piece (75 g) |  |  |  |  |  |  |  |  |  |  |  |  |  |  |  |  |  |  |  |  |  |  |  |  |  |  |
|  | 202.1 Enchiladas with beef or pork | 1 piece (75 g) |  |  |  |  |  |  |  |  |  |  |  |  |  |  |  |  |  |  |  |  |  |  |  |  |  |  |
|  | 202.2 Chicken or cheese enchilada (no red meat) | 1 piece (75 g) |  |  |  |  |  |  |  |  |  |  |  |  |  |  |  |  |  |  |  |  |  |  |  |  |  |  |
|  | 202.2 Flautas with beef or pork | 1 piece (75 g) |  |  |  |  |  |  |  |  |  |  |  |  |  |  |  |  |  |  |  |  |  |  |  |  |  |  |
|  | 202.3 Chicken or cheese flautas (no red meat) | 1 piece (75 g) |  |  |  |  |  |  |  |  |  |  |  |  |  |  |  |  |  |  |  |  |  |  |  |  |  |  |
|  | 203. Natural vegetable soups, broths or creams | 1 plate (393 g) |  |  |  |  |  |  |  |  |  |  |  |  |  |  |  |  |  |  |  |  |  |  |  |  |  |  |
|  | 204. Beef tacos | 1 piece (75 g) |  |  |  |  |  |  |  |  |  |  |  |  |  |  |  |  |  |  |  |  |  |  |  |  |  |  |
|  | 204.1 Pork tacos (adobado, al pastor) | 1 piece (75 g) |  |  |  |  |  |  |  |  |  |  |  |  |  |  |  |  |  |  |  |  |  |  |  |  |  |  |
|  | 204.2 Beans taco | 1 piece (60 g) |  |  |  |  |  |  |  |  |  |  |  |  |  |  |  |  |  |  |  |  |  |  |  |  |  |  |
|  | 204.3 Chicken or fish tacos | 1 piece (75 g) |  |  |  |  |  |  |  |  |  |  |  |  |  |  |  |  |  |  |  |  |  |  |  |  |  |  |
|  | 205. Beef burrito | 1 piece (100 g) |  |  |  |  |  |  |  |  |  |  |  |  |  |  |  |  |  |  |  |  |  |  |  |  |  |  |
|  | 205.1 Meatless Bean Burrito | 1 piece (100 g) |  |  |  |  |  |  |  |  |  |  |  |  |  |  |  |  |  |  |  |  |  |  |  |  |  |  |
|  | 205.2 Toast with beef or pork | 1 large toast (174g) |  |  |  |  |  |  |  |  |  |  |  |  |  |  |  |  |  |  |  |  |  |  |  |  |  |  |
|  | 205.2 Toast with beans or chicken | 1 large toast (174g) |  |  |  |  |  |  |  |  |  |  |  |  |  |  |  |  |  |  |  |  |  |  |  |  |  |  |
|  | 206. Rice pudding | ½ cup (178 g) |  |  |  |  |  |  |  |  |  |  |  |  |  |  |  |  |  |  |  |  |  |  |  |  |  |  |
|  | 207. Flan, jericalla, custard | 1 piece (120 ml) |  |  |  |  |  |  |  |  |  |  |  |  |  |  |  |  |  |  |  |  |  |  |  |  |  |  |
|  | 208. Capirotada | 1 slice (60 g) |  |  |  |  |  |  |  |  |  |  |  |  |  |  |  |  |  |  |  |  |  |  |  |  |  |  |
|  | 209. Stuffed or sugared churros | 1 pice (90 g) |  |  |  |  |  |  |  |  |  |  |  |  |  |  |  |  |  |  |  |  |  |  |  |  |  |  |
|  | 210. Corn atole in water | 1 cup (240 ml) |  |  |  |  |  |  |  |  |  |  |  |  |  |  |  |  |  |  |  |  |  |  |  |  |  |  |
|  | 211. Corn atole in milk or champurrado | 1 cup (240 ml) |  |  |  |  |  |  |  |  |  |  |  |  |  |  |  |  |  |  |  |  |  |  |  |  |  |  |
| **Condiments** | 212. Ketchup or canned tomato puree | 1 tablespoon (15 g) |  |  |  |  |  |  |  |  |  |  |  |  |  |  |  |  |  |  |  |  |  |  |  |  |  |  |
|  | 213. Bottled hot sauce | 1 teaspoonful (6 g) |  |  |  |  |  |  |  |  |  |  |  |  |  |  |  |  |  |  |  |  |  |  |  |  |  |  |
|  | 214. Mustard | 1 teaspoonful (5 g) |  |  |  |  |  |  |  |  |  |  |  |  |  |  |  |  |  |  |  |  |  |  |  |  |  |  |
|  | 215. Salt | 1 pinch (2 g) |  |  |  |  |  |  |  |  |  |  |  |  |  |  |  |  |  |  |  |  |  |  |  |  |  |  |
|  | 216. Soy sauce | 1 teaspoonful (5 ml) |  |  |  |  |  |  |  |  |  |  |  |  |  |  |  |  |  |  |  |  |  |  |  |  |  |  |
|  | 217. Vanilla, pepper, cinnamon, ginger, turmeric, curry, cloves, anise | 1 teaspoonful (5 g) |  |  |  |  |  |  |  |  |  |  |  |  |  |  |  |  |  |  |  |  |  |  |  |  |  |  |
|  | 218. Coffee creamer | 1 teaspoonful (5 g) |  |  |  |  |  |  |  |  |  |  |  |  |  |  |  |  |  |  |  |  |  |  |  |  |  |  |
| **Beverages** | 219. Soft drink or drink with sugar | 1 bottle (600 ml) |  |  |  |  |  |  |  |  |  |  |  |  |  |  |  |  |  |  |  |  |  |  |  |  |  |  |
|  | 220. Soft drink or drink without sugar or light | 1 bottle (600 ml) |  |  |  |  |  |  |  |  |  |  |  |  |  |  |  |  |  |  |  |  |  |  |  |  |  |  |
|  | 221. Fresh fruit water with sugar | 1 cup (240 ml) |  |  |  |  |  |  |  |  |  |  |  |  |  |  |  |  |  |  |  |  |  |  |  |  |  |  |
|  | 221.1 Fresh fruit water without sugar | 1 cup (240 ml) |  |  |  |  |  |  |  |  |  |  |  |  |  |  |  |  |  |  |  |  |  |  |  |  |  |  |
|  | 222. Natural fruit juice (orange, grapefruit) | 1 cup (240 ml) |  |  |  |  |  |  |  |  |  |  |  |  |  |  |  |  |  |  |  |  |  |  |  |  |  |  |
|  | 223. Industrialized fruit juice | 1 bottle (240 ml) |  |  |  |  |  |  |  |  |  |  |  |  |  |  |  |  |  |  |  |  |  |  |  |  |  |  |
|  | 224. Sports drink (Gatorade, etc) | 1 bottle (600 ml) |  |  |  |  |  |  |  |  |  |  |  |  |  |  |  |  |  |  |  |  |  |  |  |  |  |  |
|  | 225. Tejuino | 2 cups (418 ml) |  |  |  |  |  |  |  |  |  |  |  |  |  |  |  |  |  |  |  |  |  |  |  |  |  |  |
|  | 225.1 Tuba | 1 cup (240 ml) |  |  |  |  |  |  |  |  |  |  |  |  |  |  |  |  |  |  |  |  |  |  |  |  |  |  |
|  | 225.2 Raspados | 2 cups (418 ml) |  |  |  |  |  |  |  |  |  |  |  |  |  |  |  |  |  |  |  |  |  |  |  |  |  |  |
|  | 226. Soluble or American coffee | 1 cup (240 ml) |  |  |  |  |  |  |  |  |  |  |  |  |  |  |  |  |  |  |  |  |  |  |  |  |  |  |
|  | 227. Coffee pot | 1 cup (240 ml) |  |  |  |  |  |  |  |  |  |  |  |  |  |  |  |  |  |  |  |  |  |  |  |  |  |  |
|  | 228. Cappuccinos or lattes | 1 cup (240 ml) |  |  |  |  |  |  |  |  |  |  |  |  |  |  |  |  |  |  |  |  |  |  |  |  |  |  |
|  | 229. Frappuccinos or milk shakes | 2 cups (480 ml) |  |  |  |  |  |  |  |  |  |  |  |  |  |  |  |  |  |  |  |  |  |  |  |  |  |  |
|  | 230. Chocolate in water | 1 cup (240 ml) |  |  |  |  |  |  |  |  |  |  |  |  |  |  |  |  |  |  |  |  |  |  |  |  |  |  |
|  | 231. Milk chocolate | 1 cup (240 ml) |  |  |  |  |  |  |  |  |  |  |  |  |  |  |  |  |  |  |  |  |  |  |  |  |  |  |
|  | 232. Tea or cinnamon | 1 cup (240 ml) |  |  |  |  |  |  |  |  |  |  |  |  |  |  |  |  |  |  |  |  |  |  |  |  |  |  |
|  | 233. Natural water | 1 cup (240 ml) |  |  |  |  |  |  |  |  |  |  |  |  |  |  |  |  |  |  |  |  |  |  |  |  |  |  |
| **Alcoholic** drinks | 234. Red wine | 1 glass (100 ml) |  |  |  |  |  |  |  |  |  |  |  |  |  |  |  |  |  |  |  |  |  |  |  |  |  |  |
|  | 235. White or rosé wine | 1 glass (100 ml) |  |  |  |  |  |  |  |  |  |  |  |  |  |  |  |  |  |  |  |  |  |  |  |  |  |  |
|  | 236. Beer | 1 can or bottle (356 ml) |  |  |  |  |  |  |  |  |  |  |  |  |  |  |  |  |  |  |  |  |  |  |  |  |  |  |
|  | 237. Spirits: whiskey, vodka, gin, cognac, tequila, mezcal, rum, brandy | 1 shot (60 ml) |  |  |  |  |  |  |  |  |  |  |  |  |  |  |  |  |  |  |  |  |  |  |  |  |  |  |
|  | 238. Punch (pomegranate, raspberry) | 1 glass (80 ml) |  |  |  |  |  |  |  |  |  |  |  |  |  |  |  |  |  |  |  |  |  |  |  |  |  |  |
|  | 239. Rompope | 1 glass (80 ml) |  |  |  |  |  |  |  |  |  |  |  |  |  |  |  |  |  |  |  |  |  |  |  |  |  |  |
|  | 240. Pulque, tepache or honey water | 1 glass (80 ml) |  |  |  |  |  |  |  |  |  |  |  |  |  |  |  |  |  |  |  |  |  |  |  |  |  |  |
| **Others** | 241. Protein supplement | 1 scoop (40 g) |  |  |  |  |  |  |  |  |  |  |  |  |  |  |  |  |  |  |  |  |  |  |  |  |  |  |
|  | 242. Estevia | 1 sachet (3g) |  |  |  |  |  |  |  |  |  |  |  |  |  |  |  |  |  |  |  |  |  |  |  |  |  |  |
|  | 243. Splenda® or Canderel® | 1 sachet (3g) |  |  |  |  |  |  |  |  |  |  |  |  |  |  |  |  |  |  |  |  |  |  |  |  |  |  |
|  | 244. Multivitamin supplements (place mark) | 1 capsule |  |  |  |  |  |  |  |  |  |  |  |  |  |  |  |  |  |  |  |  |  |  |  |  |  |  |
| **Fermented foods** | 245. Kéfir | 1 cup (240 ml) |  |  |  |  |  |  |  |  |  |  |  |  |  |  |  |  |  |  |  |  |  |  |  |  |  |  |
|  | 246. Kombucha (Fermented tea) | 1 cup (240 ml) |  |  |  |  |  |  |  |  |  |  |  |  |  |  |  |  |  |  |  |  |  |  |  |  |  |  |
|  | 247. Tempeh (Fermented soy) | 1 cup (240 ml) |  |  |  |  |  |  |  |  |  |  |  |  |  |  |  |  |  |  |  |  |  |  |  |  |  |  |
|  | 248. Natto (Fermented soy) | 1 cup (240 ml) |  |  |  |  |  |  |  |  |  |  |  |  |  |  |  |  |  |  |  |  |  |  |  |  |  |  |
|  | 249. Miso (Fermented soybean paste) | 1 cup (240 ml) |  |  |  |  |  |  |  |  |  |  |  |  |  |  |  |  |  |  |  |  |  |  |  |  |  |  |
|  | 250. Kimchi (Fermented vegetables) | 1 cup (240 ml) |  |  |  |  |  |  |  |  |  |  |  |  |  |  |  |  |  |  |  |  |  |  |  |  |  |  |
|  | 251. Sourdough bread (Sourdough bread) | 1 cup (240 ml) |  |  |  |  |  |  |  |  |  |  |  |  |  |  |  |  |  |  |  |  |  |  |  |  |  |  |
|  | 252. Saurkraut (Fermented cabbage) | 1 cup (240 ml) |  |  |  |  |  |  |  |  |  |  |  |  |  |  |  |  |  |  |  |  |  |  |  |  |  |  |

Note: foods colored blue corresponds to those that will be included in the intervention program and correspond to those presented in Table A6.5, where the frequency and quantity of consumption to be promoted are specified. FCFQ adapted from: Lares-Michel, M.; Housni, F. E.; Aguilera Cervantes, V. G.; Michel Nava, R. M. Development of a Mexican online nutritional ecologic software for dietary assessment, automatic calculation of diet quality, and dietary environmental impact. *British Journal of Nutrition* (under revision).

Clinical history which will be uploaded to the mobile application (Spanish version)

Table SM1.6.2 Initial Assessment Questionnaire (Spanish version)

| Historia clínica | | | | | | | | | | | |
| --- | --- | --- | --- | --- | --- | --- | --- | --- | --- | --- | --- |
| Datos generales y sociodemográficos | | | | | | | | | | | |
| Apellido Paterno: | | | | Apellido Materno: | | | | | Nombre: | | |
| Celular: | | | | | | | | Correo electrónico: | | | |
| Edad: | | | | | | | | Fecha de nacimiento: | | | |
| Sexo: | | | | | | | | País de nacimiento: | | | |
| Ciudad de residencia: | | | | | | | | Estado de nacimiento: | | | |
| Estado civil: | | | | | | | | Religión: | | | |
| Tiempo viviendo ahí: | | | | | | | | Ocupación | | | |
| Menos de 1 mes | | | | | |  | | Funcionario, director o jefe | | |  |
| 1 a 6 meses | | | | | |  | | Profesionista o técnico | | |  |
| 6 a 12 meses | | | | | |  | | Trabajador auxiliar en actividades administrativas | | |  |
| 1 a 3 años | | | | | |  | | Comerciante, empleado en ventas o agente de ventas | | |  |
| 3 a 6 años | | | | | |  | | Trabajadores en servicios personales y de vigilancia | | |  |
| 6 a 9 años | | | | | |  | | Trabajador en actividades agrícolas, ganaderas, forestales, caza o pesca | | |  |
| 10 años o más | | | | | |  | | Trabajador artesanal | | |  |
| Toda la vida | | | | | |  | | Operador de maquinaria industrial, ensambladores, chofer o conductor de transporte | | |  |
|  |  |  |  |  |  |  |  | Trabajadores en actividades elementales y de apoyo | | |  |
| Educación | | | | | | | | Estudiante | | |  |
| No estudió | | | | | |  | | Desempleado | | |  |
| Primaria inconclusa | | | | | |  | | Jubilado | | |  |
| Primaria terminada | | | | | |  | | Otro (especificar): | | |  |
| Secundaria inconclusa | | | | | |  | | Días a la semana que trabaja | | | |
| Secundaria terminada | | | | | |  | | 1 | | |  |
| Preparatoria inconclusa | | | | | |  | | 2 | | |  |
| Preparatoria terminada | | | | | |  | | 3 | | |  |
| Carrera técnica inconclusa | | | | | |  | | 4 | | |  |
| Carrera técnica terminada | | | | | |  | | 5 | | |  |
| Estudiante de licenciatura | | | | | |  | | 6 | | |  |
| Licenciatura inconclusa | | | | | |  | | 7 | | |  |
| Licenciatura terminada | | | | | |  | | Modalidad | | |  |
| Maestría | | | | | |  | | Presencial | | |  |
| Doctorado | | | | | |  | | En línea | | |  |
| Especialidad | | | | | |  | | Mixta | | |  |
| Otra: | | | | | |  | | En línea hasta nuevo aviso | | |  |
| Ingreso mensual individual y familiar (pesos por mes MXN) | | | Gasto individual en alimentos por mes (pesos MXN) | | | Gasto del hogar en alimentos por mes (pesos MXN) | | | | Alimentación | |
| 0 - 2,699 |  |  | | |  | |  | 0 – 500 | | Alimentos favoritos |  |
| 2,700 - 6,799 |  | | 0 - 200 | |  |  | | 500 – 1,000 | |  | |
| 6,800 - 11,599 |  | | 2–0 - 500 | |  |  | | 1,000 – 3,000 | | Alimentos que no le gustan | |
| 11,600 - 34,999 |  | | 500 – 800 | |  |  | | 3,000 – 6,000 | |  | |
| 35,000 - 84,999 |  | | 800 – 1,000 | |  |  | | 6,000 – 9,000 | | Alergias e intolerancias alimentarias | |
| + 85,000 |  | | 1,500- 2,000 | |  |  | | 10,0–0 - 13,000 | | Lactosa |  |
|  |  | | 2,500 – 3,000 | |  |  | | 13,000 – 16,000 | | Mariscos |  |
|  |  | | 3,000 – 3,500 | |  |  | | 16,000 – 20,000 | | Gluten |  |
|  |  | | + 4,000 | |  |  | | + 20,000 | | Fresas |  |
| Alimentación y actividad física | | | | | | | | | | Nueces |  |
| Principal lugar de compra de alimentos | | | ¿Quién cocina los alimentos que consumes? | | | | | ¿Actualmente estás siguiendo una dieta específica? | | Cacahuates |  |
| Tianguis |  | | Yo | | |  | |  |  | Otros (especificar): |  |
| Mercado |  | | Mamá | | |  | | Si | | |  |
| Supermercado menudeo (Soriana, Waltmart) |  | | Papá | | |  | | Para bajar de peso | | |  |
| Supermercado mayoreo (Sam’s, Costco) |  | | Cocina económica | | |  | | Para subir de peso | | |  |
| Carnicería |  | | Restaurantes | | |  | | Cetogénica | | |  |
| Cremería |  | | Me la regalan | | |  | | Vegetariana (ovo-lacto, ovo o lacto) | | |  |
| Frutería |  | | Otros (especificar): | | |  | | Ayuno intermitente | | |  |
| Otros (especificar): |  | |  |  |  |  |  | Vegana | | |  |
|  |  |  |  |  |  |  |  | Mediterránea | | |  |
|  |  |  |  |  |  |  |  | DASH o dieta para detener la hipertensión | | |  |
|  |  |  |  |  |  |  |  | Otros (especificar): | | |  |

Table SM1.6.2 Initial Assessment Questionnaire (Spanish version)

| Historia clínica | | | | | | | | |
| --- | --- | --- | --- | --- | --- | --- | --- | --- |
| Actividad física | | | | | | | | |
| Actualmente realizas actividad física | | | | | | | Tipo de actividad |  |
| Menos de 3 veces a la semana | | | | | |  | Caminar |  |
| 3 o más días de actividad vigorosa de al menos 20 minutos por día | | | | | |  | Trotar |  |
| 5 o más días de actividad moderada a intensa o caminar al menos 30 minutos por día | | | | | |  | Correr |  |
| 3 días a la semana de actividad muy vigorosa al menos 60 minutos por día | | | | | |  | Aeróbics, zumba o baile |  |
| 7 días a la semana de actividad moderada a intensa o incluso caminata de al menos 60 minutos por día | | | | | |  | Ciclismo |  |
| Veces a la semana | Tipo de actividad | | | | Minutos al día | | Natación |  |
| 1 | | |  | | 10 |  | Crossfit |  |
| 2 | | |  | | 20 |  | Pesas |  |
| 3 | | |  | | 30 |  | Multifuncional |  |
| 4 | | |  | | 40 |  | Ejercicios focalizados (escribir cuales, por ejemplo abdominales, sentadillas, lagartijas) |  |
| 5 | | |  | | 50 |  | Otra: |  |
| 6 | | |  | | 60 |  |  | |
| 7 | | |  | | 90 |  |  |  |
| Intensidad | | | | | 120 |  |  |  |
| Leve | | |  | | 150 |  |  |  |
| Moderada | | |  | | 180 |  |  |  |
| Intensa | |  | 210 |  |  | | | |
|  |  |  | 240 o más |  |  |  |  |  |
|  |  |  | Otra: |  |  |  |  |  |
| Datos antropométricos | | | | | | | | |
| Peso estimado por usted: | | |  | | Talla en centímetros estimada por usted: |  |  |  |
| Talla (centímetros): | | |  | | Peso (kg): |  | IMC: |  |
| Porcentaje de grasa: | | |  | | Masa muscular: |  | Agua corporal: |  |
| Edad metabólica: | | |  | | Tasa metabólica: |  | Grasa visceral: |  |
| Circunferencia de cintura: | | |  | | Circunferencia de cadera: |  | Masa ósea: |  |
| Indicadores bioquímicos | | | | | | Indicadores clínicos | | |
| Glucosa en ayuno | | | | |  | Presión arterial | |  |
| Glucosa 2 horas después de comer | | | | |  | Acantosis nigricans | |  |
| Triglicéridos | | | | |  | Signos y síntomas de deficiencias nutricionales | | |
| Colesterol total | | | | |  | Estado general | |  |
| Colesterol LDL | | | | |  | Mucho cansancio | |  |
| Colesterol HDL | | | | |  | Mareos | |  |
| Microbiota intestinal | | | | |  | Mucha sed | |  |
| Signos y síntomas gastrointestinales | | | | | | Muchas ganas de orinar | |  |
| Inflamación abdominal | | | | |  | Mucha hambre | |  |
| ¿Con qué frecuencia ocurre? | | | | |  | ¿Se hinchan sus pies o manos? | |  |
| Diarrea | | | | |  | ¿A qué hora del día ocurre? | |  |
| ¿Con qué frecuencia ocurre? | | | | |  | ¿Con que frecuencia ocurre? | |  |
| Estreñimiento | | | | |  | ¿Cuántas horas pasa sentado al día? | |  |
| ¿Con qué frecuencia ocurre? | | | | |  | ¿Cuántas horas pasa parado al día? | |  |
| Reflujo | | | | |  | Sangrado de nariz | |  |
| ¿Con qué frecuencia ocurre? | | | | |  | ¿Con qué frecuencia ocurre? | |  |
| Inflamación abdominal | | | | |  | Manchas rojas en su piel o moretes sin motivo | |  |
| ¿Con qué frecuencia ocurre? | | | | |  | ¿Con qué frecuencia ocurre? | |  |
| Uñas | | | | | Cabello | | | |
| Uñas quebradizas | | | | | Caída de cabello | | |  |
| ¿Ha realizado algún tratamiento estético en sus uñas recientemente? | | | | | Cabello quebradizo ¿Tiene su cabello teñido o bajo algún tratamiento estético? | | |  |

Table SM1.6.2 Initial Assessment Questionnaire (Spanish version)

| Historia clínica | | | |
| --- | --- | --- | --- |
| Signos y síntomas de deficiencias nutricionales | | | |
| Boca | | Sueño |  |
| Cortaduras en las comisuras de su boca |  | ¿Cuántas horas duerme al día? |  |
| ¿Con qué frecuencia ocurre? |  | ¿Descansa? |  |
| Inflamación en lengua |  | ¿Despierta durante la noche? |  |
| ¿Con qué frecuencia ocurre? |  | ¿Con qué frecuencia ocurre? |  |
| Inflamación de encías |  | ¿Cuántas horas duerme al día? |  |
| ¿Con qué frecuencia ocurre? |  | ¿Descansa? |  |
| Exposición solar | | | |
| ¿Cuántos minutos te expones al sol al día? | | ¿Cubres tu piel con ropa de manga larga, pantalón, gorra o sombrero? | |
| Menos de 5 minutos |  | Siempre |  |
| 5 a 10 minutos |  | A veces |  |
| 10 a 15 minutos |  | Nunca |  |
| 15 a 20 minutos |  | ¿Utilizas bloqueador solar? |  |
| 20 a 30 minutos |  | Si |  |
| 30 minutos a 1 hora |  | No |  |
| Más de 1 hora |  | ¿Cuántos días te expones al sol a la semana? |  |
| Tipo de parto |  | 1 |  |
| Naciste por: |  | 2 |  |
| Parto vaginal |  | 3 |  |
| Cesárea |  | 4 |  |
| Notas: |  | 5 |  |
|  |  | 6 |  |
|  |  | 7 |  |
| Lactancia |  | Escribir por cuánto tiempo | |
| Lactancia materna exclusiva |  |  | |
| Lactancia artificial (si solo recibió como alimento leche artificial) |  | Observaciones: | |
| Lactancia mixta (si además de la lactancia materna recibió alguna leche artificial) |  |  |  |
| Lactancia materna complementada (si además de la lactancia materna recibió alimentos sólidos o líquidos no lácteos) |  |  |  |
| Lactancia mixta complementada (si fue un lactante amamantado, que además recibió alguna leche artificial y alimentos sólidos o líquidos) |  |  |  |
| Lactancia artificial complementada (si al ser lactante recibía leche artificial y alimentos sólidos o líquidos) |  |  |  |
| Festividades | | | |
| Por favor escriba las principales festividades que celebra y al lado derecho escriba los principales alimentos que consume en estas festividades | | | |
| Festividad | Alimentos consumidos en la festividad | | |
|  |  | | |
|  |  | | |
|  |  | | |
|  |  | | |
|  |  | | |
|  |  | | |
|  |  | | |
| Hábitos de higiene | | | |
| ¿Cada cuando se baña? |  | | |
| ¿Cuántas veces se lava los dientes al día? |  | | |
| La calle por donde vive está pavimentada? |  | | |
| ¿Qué tipo de piso tiene su casa? |  | | |
|  |  | | |
|  |  | | |
|  |  | | |
|  |  | | |
|  |  | | |
|  |  | | |

Table SM1.6.2 Initial Assessment Questionnaire (Spanish version)

| **Antecedentes personales y heredofamiliares** | | | | | | | | | | | | | |
| --- | --- | --- | --- | --- | --- | --- | --- | --- | --- | --- | --- | --- | --- |
| ¿Padeces alguna enfermedad? | | | | | | | | | | | | | |
| Marcar la patología |  |  |  |  |  |  |  |  |  |  |  |  |  |
|  | Diabetes tipo 2 | Hipertensión arterial | Triglicéridos elevados | Colesterol elevado | Colitis nerviosa | Cáncer | Hipotiroidismo | Hipertiroidismo | Artritis reumatoide | Insuficiencia Renal Crónica | Cardiopatía | Depresión | Otra |
| Si seleccionó la opción otra, especificar cual o cuales |  | | | | | | | | | | | | |
| **¿Alguien en tu familia padece alguna enfermedad?** | | | | | | | | | | | | | |
| Padre |  |  |  |  |  |  |  |  |  |  |  |  |  |
| Madre |  |  |  |  |  |  |  |  |  |  |  |  |  |
| Hermano |  |  |  |  |  |  |  |  |  |  |  |  |  |
| Hermana |  |  |  |  |  |  |  |  |  |  |  |  |  |
| Abuelo paterno |  |  |  |  |  |  |  |  |  |  |  |  |  |
| Abuela paterna |  |  |  |  |  |  |  |  |  |  |  |  |  |
| Abuelo materno |  |  |  |  |  |  |  |  |  |  |  |  |  |
| Abuela materna |  |  |  |  |  |  |  |  |  |  |  |  |  |
| Tío paterno |  |  |  |  |  |  |  |  |  |  |  |  |  |
| Tía paterna |  |  |  |  |  |  |  |  |  |  |  |  |  |
| Tío materno |  |  |  |  |  |  |  |  |  |  |  |  |  |
| Tía materna |  |  |  |  |  |  |  |  |  |  |  |  |  |
| Otras: |  | | | | | | | | | | | | |
| ¿Actualmente consumes algún medicamento y/o suplemento? | | | | | | | | | | | | | |
| **Medicamentos** |  |  |  |  |  |  |  |  |  |  |  |  |  |
|  | Ampicilina | Bezafibrato | Cefalexina | Ciprofloxacino | Claritromicina | Fluoxetina | Insulina | Levotiroxina | Lozartan | Metformina | Omeprazol | Pravastatina | Telmisartan |
| **Otra.** Si seleccionó la opción otra, especificar cual o cuales |  | | | | | | | | | | | | |
| **Suplementos** |  |  |  |  |  |  |  |  |  |  |  |  |  |
|  | Multivitamínico | Vitamina D | Probióticos | Prebióticos | Vitamina C | Vitamina E | Hierro | Calcio | Omega 3 | Proteína en polvo | Creatina monohidratada | Otros |  |

Table SM1.6.2 Initial Assessment Questionnaire (Spanish version)

| **Recordatorio de 24 horas** | | | |
| --- | --- | --- | --- |
| **Tiempo de comida** | **Menú / Preparación** | **Ingrediente / Alimento** | **Cantidad** |
| **Desayuno** |  |  |  |
| Hora: |  |  |  |
| Lugar: |  |  |  |
| **Colación** |  |  |  |
| Hora: |  |  |  |
| Lugar: |  |  |  |
| **Comida** |  |  |  |
| Hora: |  |  |  |
| Lugar: |  |  |  |
| **Colación** |  |  |  |
| Hora: |  |  |  |
| Lugar: |  |  |  |
| **Cena** |  |  |  |
| Hora: |  |  |  |
| Lugar: |  |  |  |
| **Mililitros de agua consumida en todo el día:** | | | |

Table SM1.6.2 Initial Assessment Questionnaire (Spanish version)

| **Cuestionario de frecuencia de consumo de alimentos** | | | | | | | | | | | | | | | | | | | | | | | | | | | | |
| --- | --- | --- | --- | --- | --- | --- | --- | --- | --- | --- | --- | --- | --- | --- | --- | --- | --- | --- | --- | --- | --- | --- | --- | --- | --- | --- | --- | --- |
| Para cada alimento, señale cuentas veces al mes o a la semana lo consume. Posteriormente, indique cuantas veces al día lo consume, el día en que lo ingiere. Por ejemplo, si usted toma 2 tazas (480 mililitros) de leche entera 3 veces a la semana y esos 3 días la consume en la mañana y en la noche, usted la consume 3 veces a la semana y 2 veces al día, en una cantidad de 2 porciones. También es importante considerar la variación verano/invierno. Por ejemplo, si usted consume helados 4 veces a la semana los 3 meses de verano, su consumo promedio es de 1 vez a la semana. | | | | | | | | | | | | | | | | | | | | | | | | | | | | |
| **Grupo** | **Alimento** | **Porción** | Tipo, marca, sabor o preparación | **Consumo medio durante el año pasado**  *Marque 1 opción de entre estas 3 frecuencias.  *Si no lo consume, elija la opción nunca y pase al siguiente alimento. | | | | | | | | | | | | | **Cuando lo consume, ¿cuántas veces al día lo hace?**  **(Marque 1 opción al día)** | | | | **Número de porciones** | | | | | | | |
|  |  |  |  | **Veces al año** | | | **Veces al mes** | | | **Veces a la semana** | | | | | | | **Veces al día** | | | | **(Marque cuantas porciones consume cada vez que lo come)** | | | | | | | |
|  |  |  |  | Nunca | 1-5 | 6-11 | 1 | 2 | 3 | 1 | 2 | 3 | 4 | 5 | 6 | 7 | 1 | 2 | 3 | 4+ | 1/4 | 1/2 | 1 | 2 | 3 | 4 | 5 | 6+ |
| **Leches** | 1. Leche entera | 1 taza (240 ml) |  |  |  |  |  |  |  |  |  |  |  |  |  |  |  |  |  |  |  |  |  |  |  |  |  |  |
|  | 2. Leche semidescremada | 1 taza (240 ml) |  |  |  |  |  |  |  |  |  |  |  |  |  |  |  |  |  |  |  |  |  |  |  |  |  |  |
|  | 3. Leche descremada (light) | 1 taza (240 ml) |  |  |  |  |  |  |  |  |  |  |  |  |  |  |  |  |  |  |  |  |  |  |  |  |  |  |
|  | 4. Leche de almendra | 1 taza (240 ml) |  |  |  |  |  |  |  |  |  |  |  |  |  |  |  |  |  |  |  |  |  |  |  |  |  |  |
|  | 5. Leche de soya (especificar si natural o de sabor) | 1 taza (240 ml) |  |  |  |  |  |  |  |  |  |  |  |  |  |  |  |  |  |  |  |  |  |  |  |  |  |  |
|  | 6. Leche de coco | 1 taza (240 ml) |  |  |  |  |  |  |  |  |  |  |  |  |  |  |  |  |  |  |  |  |  |  |  |  |  |  |
|  | 7. Leche preparada de sabor (chocolate, fresa) | 1 taza (240 ml) |  |  |  |  |  |  |  |  |  |  |  |  |  |  |  |  |  |  |  |  |  |  |  |  |  |  |
|  | 8. Licuado de leche envasado | 1 taza (240 ml) |  |  |  |  |  |  |  |  |  |  |  |  |  |  |  |  |  |  |  |  |  |  |  |  |  |  |
|  | 9. Yogurt entero (especificar si natural o de sabor) | 1 taza (227 g) |  |  |  |  |  |  |  |  |  |  |  |  |  |  |  |  |  |  |  |  |  |  |  |  |  |  |
|  | 10. Yogurt descremado (light natural o de sabor) | 1 taza (240 g) |  |  |  |  |  |  |  |  |  |  |  |  |  |  |  |  |  |  |  |  |  |  |  |  |  |  |
|  | 11. Bebida láctea fermentada (yakult) | 1 pieza (80 g) |  |  |  |  |  |  |  |  |  |  |  |  |  |  |  |  |  |  |  |  |  |  |  |  |  |  |
|  | 12. Crema para biónico (leche evaporada con crema) | ½ taza (120 g) |  |  |  |  |  |  |  |  |  |  |  |  |  |  |  |  |  |  |  |  |  |  |  |  |  |  |
| **Quesos** | 13. Requesón | 3 cucharadas (42 g) |  |  |  |  |  |  |  |  |  |  |  |  |  |  |  |  |  |  |  |  |  |  |  |  |  |  |
|  | 14. Queso cotagge | 3 cucharadas (48 g) |  |  |  |  |  |  |  |  |  |  |  |  |  |  |  |  |  |  |  |  |  |  |  |  |  |  |
|  | 15. Queso fresco (no en quesadillas) | 1 rebanada (40 g) |  |  |  |  |  |  |  |  |  |  |  |  |  |  |  |  |  |  |  |  |  |  |  |  |  |  |
|  | 15.1 Queso panela (no en quesadillas) | 1 rebanada (40 g) |  |  |  |  |  |  |  |  |  |  |  |  |  |  |  |  |  |  |  |  |  |  |  |  |  |  |
|  | 16. Quesos curados consumidos solos (manchego, oaxaca, mozzarella, amarillo, cabra) (no en quesadillas) | 1 rebanada (30 g) |  |  |  |  |  |  |  |  |  |  |  |  |  |  |  |  |  |  |  |  |  |  |  |  |  |  |
|  | 17. Huevos de gallina | 1 pieza (60 g) |  |  |  |  |  |  |  |  |  |  |  |  |  |  |  |  |  |  |  |  |  |  |  |  |  |  |
|  | 18. Clara de huevo | 2 piezas (66 g) |  |  |  |  |  |  |  |  |  |  |  |  |  |  |  |  |  |  |  |  |  |  |  |  |  |  |
|  | 19. Pollo con piel (piezas de pollo excepto alitas) | 1 pieza (200 g) |  |  |  |  |  |  |  |  |  |  |  |  |  |  |  |  |  |  |  |  |  |  |  |  |  |  |
|  | 20. Pollo sin piel en piezas | 1 pieza (200 g) |  |  |  |  |  |  |  |  |  |  |  |  |  |  |  |  |  |  |  |  |  |  |  |  |  |  |
|  | 20.1 Pollo sin piel en filete a la plancha | 1 ración (90 g) |  |  |  |  |  |  |  |  |  |  |  |  |  |  |  |  |  |  |  |  |  |  |  |  |  |  |
|  | 21. Pavo | 1 ración (108 g) |  |  |  |  |  |  |  |  |  |  |  |  |  |  |  |  |  |  |  |  |  |  |  |  |  |  |
|  | 21.1 Conejo | 1 ración (90 g) |  |  |  |  |  |  |  |  |  |  |  |  |  |  |  |  |  |  |  |  |  |  |  |  |  |  |
|  | 22. Carne de res en filete o bistec (guisos, plancha) | 1 ración (90 g) |  |  |  |  |  |  |  |  |  |  |  |  |  |  |  |  |  |  |  |  |  |  |  |  |  |  |
|  | 23. Carne de res molida (albóndigas, picadillo) | 1 ración (99 g) |  |  |  |  |  |  |  |  |  |  |  |  |  |  |  |  |  |  |  |  |  |  |  |  |  |  |
|  | 24. Carne de cerdo (guisos, a la plancha) | 1 ración (120 g) |  |  |  |  |  |  |  |  |  |  |  |  |  |  |  |  |  |  |  |  |  |  |  |  |  |  |
|  | 25. Carne de borrego (al pastor, guisos) | 1 ración (111 g) |  |  |  |  |  |  |  |  |  |  |  |  |  |  |  |  |  |  |  |  |  |  |  |  |  |  |
| **Alimentos de origen animal** | 26. Carne de chivo (birria) | 1 ración (100 g) |  |  |  |  |  |  |  |  |  |  |  |  |  |  |  |  |  |  |  |  |  |  |  |  |  |  |
|  | 27. Hígado o vísceras de pollo, res o cerdo (sesos, molleja, corazón) Especificar animal | 1 ración (90 g) |  |  |  |  |  |  |  |  |  |  |  |  |  |  |  |  |  |  |  |  |  |  |  |  |  |  |
|  | 28. Jamón de pavo o cerdo (aparte de sándwich) | 1 rebanada (21 g) |  |  |  |  |  |  |  |  |  |  |  |  |  |  |  |  |  |  |  |  |  |  |  |  |  |  |
|  | 29. Salchicha de pavo o cerdo (aparte de hot dog) | 1 pieza (61 g) |  |  |  |  |  |  |  |  |  |  |  |  |  |  |  |  |  |  |  |  |  |  |  |  |  |  |
|  | 30. Carnes procesadas (peperoni, salami, longaniza, chorizo, machaca) | ½ taza (20 g) |  |  |  |  |  |  |  |  |  |  |  |  |  |  |  |  |  |  |  |  |  |  |  |  |  |  |
|  | 31. Tocino | 1 rebanada (16 g) |  |  |  |  |  |  |  |  |  |  |  |  |  |  |  |  |  |  |  |  |  |  |  |  |  |  |
|  | 32. Chicharrón de cerdo | ½ taza (24 g) |  |  |  |  |  |  |  |  |  |  |  |  |  |  |  |  |  |  |  |  |  |  |  |  |  |  |
|  | 33. Pescado blanco (tilapia, lenguado) | 1 ración (120 g) |  |  |  |  |  |  |  |  |  |  |  |  |  |  |  |  |  |  |  |  |  |  |  |  |  |  |
|  | 34. Pescado azul (salmón, atún, huachinango) | 1 ración (120 g) |  |  |  |  |  |  |  |  |  |  |  |  |  |  |  |  |  |  |  |  |  |  |  |  |  |  |
|  | 35. Pescados salados (bacalao, charales secos) | 1 ración (90 g) |  |  |  |  |  |  |  |  |  |  |  |  |  |  |  |  |  |  |  |  |  |  |  |  |  |  |
|  | 36. Pescado (atún) o mariscos enlatados en agua | ½ lata (55g) |  |  |  |  |  |  |  |  |  |  |  |  |  |  |  |  |  |  |  |  |  |  |  |  |  |  |
|  | 37. Pescado (atún) o mariscos enlatados en aceite | ½ lata (55g) |  |  |  |  |  |  |  |  |  |  |  |  |  |  |  |  |  |  |  |  |  |  |  |  |  |  |
|  | 38. Crustáceos (camarones) | 10 piezas (68 g) |  |  |  |  |  |  |  |  |  |  |  |  |  |  |  |  |  |  |  |  |  |  |  |  |  |  |
|  | 38.1 Langostino de rio | 10 piezas (68 g) |  |  |  |  |  |  |  |  |  |  |  |  |  |  |  |  |  |  |  |  |  |  |  |  |  |  |
|  | 39. Pulpo | 1 ración (100 g) |  |  |  |  |  |  |  |  |  |  |  |  |  |  |  |  |  |  |  |  |  |  |  |  |  |  |
|  | 40. Ostiones, almejas, mejillones | 4 piezas (58 g) |  |  |  |  |  |  |  |  |  |  |  |  |  |  |  |  |  |  |  |  |  |  |  |  |  |  |
|  | 40.1 Insectos (gusanos de maguey, grillos) | 1 porción (35 g) |  |  |  |  |  |  |  |  |  |  |  |  |  |  |  |  |  |  |  |  |  |  |  |  |  |  |
| **Vegetales** | 41. Verduras congeladas | ½ taza (46 g) |  |  |  |  |  |  |  |  |  |  |  |  |  |  |  |  |  |  |  |  |  |  |  |  |  |  |
|  | 42. Espinacas, acelgas o verdolagas (especificar) | 1/2 taza (30 g) |  |  |  |  |  |  |  |  |  |  |  |  |  |  |  |  |  |  |  |  |  |  |  |  |  |  |
|  | 43. Brócoli o coliflor | ½ taza (108 g) |  |  |  |  |  |  |  |  |  |  |  |  |  |  |  |  |  |  |  |  |  |  |  |  |  |  |
|  | 44. Lechuga, repollo, col o kale (especificar) | ½ taza (25 g) |  |  |  |  |  |  |  |  |  |  |  |  |  |  |  |  |  |  |  |  |  |  |  |  |  |  |
|  | 45. Jitomate o tomate rojo crudo o en salsa | 1 pieza (60 g) |  |  |  |  |  |  |  |  |  |  |  |  |  |  |  |  |  |  |  |  |  |  |  |  |  |  |
|  | 46. Zanahoria | ½ pieza (64g) |  |  |  |  |  |  |  |  |  |  |  |  |  |  |  |  |  |  |  |  |  |  |  |  |  |  |
|  | 47. Calabacita | 1 pieza (91 g) |  |  |  |  |  |  |  |  |  |  |  |  |  |  |  |  |  |  |  |  |  |  |  |  |  |  |
|  | 48. Calabaza cocida | ½ taza (110 g) |  |  |  |  |  |  |  |  |  |  |  |  |  |  |  |  |  |  |  |  |  |  |  |  |  |  |
|  | 49. Ejotes o chicharos | ½ taza (63 g) |  |  |  |  |  |  |  |  |  |  |  |  |  |  |  |  |  |  |  |  |  |  |  |  |  |  |
|  | 50. Pepino | 1 pieza (104 g) |  |  |  |  |  |  |  |  |  |  |  |  |  |  |  |  |  |  |  |  |  |  |  |  |  |  |
|  | 51. Apio | ½ taza (45 g) |  |  |  |  |  |  |  |  |  |  |  |  |  |  |  |  |  |  |  |  |  |  |  |  |  |  |
|  | 52. Betabel | ¼ pieza (39 g) |  |  |  |  |  |  |  |  |  |  |  |  |  |  |  |  |  |  |  |  |  |  |  |  |  |  |
|  | 53. Berenjena | ½ taza (50 g) |  |  |  |  |  |  |  |  |  |  |  |  |  |  |  |  |  |  |  |  |  |  |  |  |  |  |
|  | 54. Espárragos | 3 piezas (45 g) |  |  |  |  |  |  |  |  |  |  |  |  |  |  |  |  |  |  |  |  |  |  |  |  |  |  |
|  | 55. Cebolla | ¼ taza (29 g) |  |  |  |  |  |  |  |  |  |  |  |  |  |  |  |  |  |  |  |  |  |  |  |  |  |  |
|  | 56. Tomate verde crudo o en salsa | 4 piezas (69 g) |  |  |  |  |  |  |  |  |  |  |  |  |  |  |  |  |  |  |  |  |  |  |  |  |  |  |
|  | 57. Champiñones o setas | ½ taza (70 g) |  |  |  |  |  |  |  |  |  |  |  |  |  |  |  |  |  |  |  |  |  |  |  |  |  |  |
|  | 57.1 Huitlacoche | 1/3 taza (66 g) |  |  |  |  |  |  |  |  |  |  |  |  |  |  |  |  |  |  |  |  |  |  |  |  |  |  |
|  | 58. Chayote | ½ taza (80 g) |  |  |  |  |  |  |  |  |  |  |  |  |  |  |  |  |  |  |  |  |  |  |  |  |  |  |
|  | 59. Jícama | ½ taza (60 g) |  |  |  |  |  |  |  |  |  |  |  |  |  |  |  |  |  |  |  |  |  |  |  |  |  |  |
|  | 60. Nopales cocidos, crudos o asados | ½ taza (75g) |  |  |  |  |  |  |  |  |  |  |  |  |  |  |  |  |  |  |  |  |  |  |  |  |  |  |
|  | 61. Pimiento morrón | 1/2 pieza (32 g) |  |  |  |  |  |  |  |  |  |  |  |  |  |  |  |  |  |  |  |  |  |  |  |  |  |  |
|  | 62. Chile poblano | 1/2 pieza (32 g) |  |  |  |  |  |  |  |  |  |  |  |  |  |  |  |  |  |  |  |  |  |  |  |  |  |  |
|  | 62.1 Quelites | ½ taza (66 g) |  |  |  |  |  |  |  |  |  |  |  |  |  |  |  |  |  |  |  |  |  |  |  |  |  |  |
|  | 62.2 Verdolagas | 1 taza (115 g) |  |  |  |  |  |  |  |  |  |  |  |  |  |  |  |  |  |  |  |  |  |  |  |  |  |  |
|  | 63. Chiles picantes (jalapeño, serrano, habanero) | 1 pieza (30 g) |  |  |  |  |  |  |  |  |  |  |  |  |  |  |  |  |  |  |  |  |  |  |  |  |  |  |
|  | 64. Ajo | ¼ pieza (1 g) |  |  |  |  |  |  |  |  |  |  |  |  |  |  |  |  |  |  |  |  |  |  |  |  |  |  |
|  | 65. Cilantro, perejil, orégano, albahaca, laurel | 1 pizca (6 g) |  |  |  |  |  |  |  |  |  |  |  |  |  |  |  |  |  |  |  |  |  |  |  |  |  |  |
| **Frutas** | 66. Naranja (no en jugo) | 1 pieza (76 g) |  |  |  |  |  |  |  |  |  |  |  |  |  |  |  |  |  |  |  |  |  |  |  |  |  |  |
|  | 67. Toronja (no en jugo) | 1 pieza (76 g) |  |  |  |  |  |  |  |  |  |  |  |  |  |  |  |  |  |  |  |  |  |  |  |  |  |  |
|  | 68. Mandarinas (no en jugo) | 1 pieza (76 g) |  |  |  |  |  |  |  |  |  |  |  |  |  |  |  |  |  |  |  |  |  |  |  |  |  |  |
|  | 69. Limón | 1 pieza (38 g) |  |  |  |  |  |  |  |  |  |  |  |  |  |  |  |  |  |  |  |  |  |  |  |  |  |  |
|  | 70. Limas (no en jugo) | 1 pieza (49 g) |  |  |  |  |  |  |  |  |  |  |  |  |  |  |  |  |  |  |  |  |  |  |  |  |  |  |
|  | 71. Plátano (no frito) | 1 pieza (108 g) |  |  |  |  |  |  |  |  |  |  |  |  |  |  |  |  |  |  |  |  |  |  |  |  |  |  |
|  | 72. Manzana | 1 pieza (106 g) |  |  |  |  |  |  |  |  |  |  |  |  |  |  |  |  |  |  |  |  |  |  |  |  |  |  |
|  | 73. Pera | 1 pieza (162 g) |  |  |  |  |  |  |  |  |  |  |  |  |  |  |  |  |  |  |  |  |  |  |  |  |  |  |
|  | 74. Fresas | 6 piezas (68 g) |  |  |  |  |  |  |  |  |  |  |  |  |  |  |  |  |  |  |  |  |  |  |  |  |  |  |
|  | 75. Arándano | ½ taza (49 g) |  |  |  |  |  |  |  |  |  |  |  |  |  |  |  |  |  |  |  |  |  |  |  |  |  |  |
|  | 76. Frambuesa | ½ taza (61 g) |  |  |  |  |  |  |  |  |  |  |  |  |  |  |  |  |  |  |  |  |  |  |  |  |  |  |
|  | 77. Zarzamora | ½ taza (72 g) |  |  |  |  |  |  |  |  |  |  |  |  |  |  |  |  |  |  |  |  |  |  |  |  |  |  |
|  | 78. Ciruela criolla | 2 piezas (105 g) |  |  |  |  |  |  |  |  |  |  |  |  |  |  |  |  |  |  |  |  |  |  |  |  |  |  |
|  | 78.1 Capulín | 1 taza (32 g) |  |  |  |  |  |  |  |  |  |  |  |  |  |  |  |  |  |  |  |  |  |  |  |  |  |  |
|  | 78.2 Nance | 5 piezas (15 g) |  |  |  |  |  |  |  |  |  |  |  |  |  |  |  |  |  |  |  |  |  |  |  |  |  |  |
|  | 79. Cerezas | 4 piezas (18 g) |  |  |  |  |  |  |  |  |  |  |  |  |  |  |  |  |  |  |  |  |  |  |  |  |  |  |
|  | 80. Durazno | 1 pieza (77 g) |  |  |  |  |  |  |  |  |  |  |  |  |  |  |  |  |  |  |  |  |  |  |  |  |  |  |
|  | 81. Nectarina | 1 pieza (124 g) |  |  |  |  |  |  |  |  |  |  |  |  |  |  |  |  |  |  |  |  |  |  |  |  |  |  |
|  | 82. Sandía | 1 taza (160 g) |  |  |  |  |  |  |  |  |  |  |  |  |  |  |  |  |  |  |  |  |  |  |  |  |  |  |
|  | 83. Melón | 1 taza (160 g) |  |  |  |  |  |  |  |  |  |  |  |  |  |  |  |  |  |  |  |  |  |  |  |  |  |  |
|  | 84. Papaya | 1 taza (140 g) |  |  |  |  |  |  |  |  |  |  |  |  |  |  |  |  |  |  |  |  |  |  |  |  |  |  |
|  | 85. Kiwi | 1 pieza (76 g) |  |  |  |  |  |  |  |  |  |  |  |  |  |  |  |  |  |  |  |  |  |  |  |  |  |  |
|  | 86. Uvas | 18 piezas (86 g) |  |  |  |  |  |  |  |  |  |  |  |  |  |  |  |  |  |  |  |  |  |  |  |  |  |  |
|  | 87. Mango | ½ pieza (110 g) |  |  |  |  |  |  |  |  |  |  |  |  |  |  |  |  |  |  |  |  |  |  |  |  |  |  |
|  | 88. Guayaba | 2 piezas (83 g) |  |  |  |  |  |  |  |  |  |  |  |  |  |  |  |  |  |  |  |  |  |  |  |  |  |  |
|  | 89. Tuna | 1 pieza (70 g) |  |  |  |  |  |  |  |  |  |  |  |  |  |  |  |  |  |  |  |  |  |  |  |  |  |  |
|  | 90. Pitaya | 1 pieza (50 g) |  |  |  |  |  |  |  |  |  |  |  |  |  |  |  |  |  |  |  |  |  |  |  |  |  |  |
|  | 91. Guanábana | ½ pieza (175 g) |  |  |  |  |  |  |  |  |  |  |  |  |  |  |  |  |  |  |  |  |  |  |  |  |  |  |
|  | 92. Piña | 1 taza (165 g) |  |  |  |  |  |  |  |  |  |  |  |  |  |  |  |  |  |  |  |  |  |  |  |  |  |  |
|  | 93. Fruta en almíbar | ½ taza (124 g) |  |  |  |  |  |  |  |  |  |  |  |  |  |  |  |  |  |  |  |  |  |  |  |  |  |  |
|  | 94. Fruta deshidratada (arándanos, pasitas, dátiles, ciruela pasa) | 10 piezas (20 g) |  |  |  |  |  |  |  |  |  |  |  |  |  |  |  |  |  |  |  |  |  |  |  |  |  |  |
| **Aceites con proteína** | 95. Almendras | 10 piezas (12 g) |  |  |  |  |  |  |  |  |  |  |  |  |  |  |  |  |  |  |  |  |  |  |  |  |  |  |
|  | 96. Cacahuates sin procesar | 14 piezas (12 g) |  |  |  |  |  |  |  |  |  |  |  |  |  |  |  |  |  |  |  |  |  |  |  |  |  |  |
|  | 96.1 Cacahuates (japoneses, enchilados, con sal) | 14 piezas (12 g) |  |  |  |  |  |  |  |  |  |  |  |  |  |  |  |  |  |  |  |  |  |  |  |  |  |  |
|  | 97. Nueces | 7 mitades (10 g) |  |  |  |  |  |  |  |  |  |  |  |  |  |  |  |  |  |  |  |  |  |  |  |  |  |  |
|  | 98. Pistaches, avellanas, piñones | 18 piezas (13 g) |  |  |  |  |  |  |  |  |  |  |  |  |  |  |  |  |  |  |  |  |  |  |  |  |  |  |
|  | 98.1 Habas secas | 18 piezas (13 g) |  |  |  |  |  |  |  |  |  |  |  |  |  |  |  |  |  |  |  |  |  |  |  |  |  |  |
|  | 99. Semillas de girasol o hemp | 1 cucharada (10 g) |  |  |  |  |  |  |  |  |  |  |  |  |  |  |  |  |  |  |  |  |  |  |  |  |  |  |
|  | 99.1 Semillas de calabaza | 1 cucharada (10 g) |  |  |  |  |  |  |  |  |  |  |  |  |  |  |  |  |  |  |  |  |  |  |  |  |  |  |
|  | 100. Chía | 1 cucharadita (3 g) |  |  |  |  |  |  |  |  |  |  |  |  |  |  |  |  |  |  |  |  |  |  |  |  |  |  |
|  | 100.1 Linaza | 1 cucharadita (3 g) |  |  |  |  |  |  |  |  |  |  |  |  |  |  |  |  |  |  |  |  |  |  |  |  |  |  |
|  | 101. Crema de cacahuate o almendra | 1 cucharada (15 g) |  |  |  |  |  |  |  |  |  |  |  |  |  |  |  |  |  |  |  |  |  |  |  |  |  |  |
| **Leguminosas** | 102. Frijoles cocidos | ½ taza (99 g) |  |  |  |  |  |  |  |  |  |  |  |  |  |  |  |  |  |  |  |  |  |  |  |  |  |  |
|  | 103. Frijoles refritos | ½ taza (99 g) |  |  |  |  |  |  |  |  |  |  |  |  |  |  |  |  |  |  |  |  |  |  |  |  |  |  |
|  | 104. Lentejas cocidas | ½ taza (99 g) |  |  |  |  |  |  |  |  |  |  |  |  |  |  |  |  |  |  |  |  |  |  |  |  |  |  |
|  | 105. Garbanzos cocidos | ½ taza (82 g) |  |  |  |  |  |  |  |  |  |  |  |  |  |  |  |  |  |  |  |  |  |  |  |  |  |  |
|  | 105.1 Habas cocidas | ½ taza (99 g) |  |  |  |  |  |  |  |  |  |  |  |  |  |  |  |  |  |  |  |  |  |  |  |  |  |  |
|  | 106. Hummus de garbanzo | 4 cucharadas (60 g) |  |  |  |  |  |  |  |  |  |  |  |  |  |  |  |  |  |  |  |  |  |  |  |  |  |  |
|  | 107. Soya texturizada | 1/2 taza (30 g) |  |  |  |  |  |  |  |  |  |  |  |  |  |  |  |  |  |  |  |  |  |  |  |  |  |  |
| **Cereales sin grasa** | 108. Tortilla de maíz | 1 pieza (30 g) |  |  |  |  |  |  |  |  |  |  |  |  |  |  |  |  |  |  |  |  |  |  |  |  |  |  |
|  | 10.8.1 Masa de maíz | 1 bolita (45 g) |  |  |  |  |  |  |  |  |  |  |  |  |  |  |  |  |  |  |  |  |  |  |  |  |  |  |
|  | 109. Elote | 1 pieza (116 g) |  |  |  |  |  |  |  |  |  |  |  |  |  |  |  |  |  |  |  |  |  |  |  |  |  |  |
|  | 110. Papa asada o cocida | 1 papa (136 g) |  |  |  |  |  |  |  |  |  |  |  |  |  |  |  |  |  |  |  |  |  |  |  |  |  |  |
|  | 111. Tortilla de harina | 1 pieza (28 g) |  |  |  |  |  |  |  |  |  |  |  |  |  |  |  |  |  |  |  |  |  |  |  |  |  |  |
|  | 112. Tortilla de harina integral | 1 pieza (28 g) |  |  |  |  |  |  |  |  |  |  |  |  |  |  |  |  |  |  |  |  |  |  |  |  |  |  |
|  | 113. Bolillo, birote o telera (no en torta) | ½ pieza (30 g) |  |  |  |  |  |  |  |  |  |  |  |  |  |  |  |  |  |  |  |  |  |  |  |  |  |  |
|  | 114. Pan blanco de caja o tostado (aparte de sándwich) | 1 rebanada (27 g) |  |  |  |  |  |  |  |  |  |  |  |  |  |  |  |  |  |  |  |  |  |  |  |  |  |  |
|  | 115. Pan integral de caja o tostado (aparte de sándwich) | 1 rebanada (25 g) |  |  |  |  |  |  |  |  |  |  |  |  |  |  |  |  |  |  |  |  |  |  |  |  |  |  |
|  | 116. Cereal de caja (special k, corn flakes) | 1 taza (25 g) |  |  |  |  |  |  |  |  |  |  |  |  |  |  |  |  |  |  |  |  |  |  |  |  |  |  |
|  | 117. Cereal de caja azucarado (chococrispis, zucaritas) | 1 taza (25 g) |  |  |  |  |  |  |  |  |  |  |  |  |  |  |  |  |  |  |  |  |  |  |  |  |  |  |
|  | 118. Cereal de caja integral (all bran, muesli) | 1 taza (25 g) |  |  |  |  |  |  |  |  |  |  |  |  |  |  |  |  |  |  |  |  |  |  |  |  |  |  |
|  | 119. Pasta (fideos, macarrones, espagueti) | ½ taza (60 g) |  |  |  |  |  |  |  |  |  |  |  |  |  |  |  |  |  |  |  |  |  |  |  |  |  |  |
|  | 120. Avena (en hojuelas, cocida en agua) | ½ taza (20 g) |  |  |  |  |  |  |  |  |  |  |  |  |  |  |  |  |  |  |  |  |  |  |  |  |  |  |
|  | 121. Avena cocida con leche y azúcar (bebida o sólida) | 1 taza (240 ml) |  |  |  |  |  |  |  |  |  |  |  |  |  |  |  |  |  |  |  |  |  |  |  |  |  |  |
|  | 122. Arroz blanco cocido | ½ taza (94 g) |  |  |  |  |  |  |  |  |  |  |  |  |  |  |  |  |  |  |  |  |  |  |  |  |  |  |
|  | 123. Arroz integral o salvaje | ½ taza (94 g) |  |  |  |  |  |  |  |  |  |  |  |  |  |  |  |  |  |  |  |  |  |  |  |  |  |  |
|  | 124. Arroz inflado natural o tortita de arroz inflado | ½ taza (17 g) |  |  |  |  |  |  |  |  |  |  |  |  |  |  |  |  |  |  |  |  |  |  |  |  |  |  |
|  | 125. Amaranto sin endulzar | ½ taza (32 g) |  |  |  |  |  |  |  |  |  |  |  |  |  |  |  |  |  |  |  |  |  |  |  |  |  |  |
|  | 125.1 Amaranto endulzado o barra de amaranto | ½ taza (32 g) |  |  |  |  |  |  |  |  |  |  |  |  |  |  |  |  |  |  |  |  |  |  |  |  |  |  |
|  | 126. Camote cocido | ½ taza (103 g) |  |  |  |  |  |  |  |  |  |  |  |  |  |  |  |  |  |  |  |  |  |  |  |  |  |  |
|  | 127. Camote cocido con azúcar | ½ taza (103 g) |  |  |  |  |  |  |  |  |  |  |  |  |  |  |  |  |  |  |  |  |  |  |  |  |  |  |
|  | 128. Quínoa | ½ taza (40 g) |  |  |  |  |  |  |  |  |  |  |  |  |  |  |  |  |  |  |  |  |  |  |  |  |  |  |
|  | 129. Galletas Marías o de animalitos | 5 piezas (19 g) |  |  |  |  |  |  |  |  |  |  |  |  |  |  |  |  |  |  |  |  |  |  |  |  |  |  |
|  | 130. Galletas integrales o con fibra (dulce: tipo la integral®, o salada: tipo habaneras®) | 1 dulce o 9 saladas (45 g) |  |  |  |  |  |  |  |  |  |  |  |  |  |  |  |  |  |  |  |  |  |  |  |  |  |  |
|  | 131. Galletas saladas (tipo saladitas®) | 4 piezas (16 g) |  |  |  |  |  |  |  |  |  |  |  |  |  |  |  |  |  |  |  |  |  |  |  |  |  |  |
|  | 132. Tostada de maíz horneada (tipo sanissimo®) | 1 pieza (10 g) |  |  |  |  |  |  |  |  |  |  |  |  |  |  |  |  |  |  |  |  |  |  |  |  |  |  |
| **Cereales con grasa**  **Leguminosas**  **Cereales sin grasa** | 133. Tostada de maíz frita (botanera, raspada) | 1 pieza (12 g) |  |  |  |  |  |  |  |  |  |  |  |  |  |  |  |  |  |  |  |  |  |  |  |  |  |  |
|  | 134. Galletas dulces (sándwich tipo príncipe®, emperador®, chispas de chocolate, canelitas®, etc) | 2 piezas (21 g) |  |  |  |  |  |  |  |  |  |  |  |  |  |  |  |  |  |  |  |  |  |  |  |  |  |  |
|  | 135. Barra de granola | 1 pieza (35g) |  |  |  |  |  |  |  |  |  |  |  |  |  |  |  |  |  |  |  |  |  |  |  |  |  |  |
|  | 136. Granola con frutos secos | 3 cucharadas (21 g) |  |  |  |  |  |  |  |  |  |  |  |  |  |  |  |  |  |  |  |  |  |  |  |  |  |  |
|  | 137. Hot cake o waffle | 1 pieza (50 g) |  |  |  |  |  |  |  |  |  |  |  |  |  |  |  |  |  |  |  |  |  |  |  |  |  |  |
|  | 138. Pan industrializado (donas, mantecadas®, submarinos®, etc) | 1 pieza (30 g) |  |  |  |  |  |  |  |  |  |  |  |  |  |  |  |  |  |  |  |  |  |  |  |  |  |  |
|  | 139. Pan industrializado con chocolate (chocorroles®, gansito®, negrito®, etc) | 1 pieza (50 g) |  |  |  |  |  |  |  |  |  |  |  |  |  |  |  |  |  |  |  |  |  |  |  |  |  |  |
|  | 140. Pan dulce (conchas, orejas, polvorón) | 1 pieza (63 g) |  |  |  |  |  |  |  |  |  |  |  |  |  |  |  |  |  |  |  |  |  |  |  |  |  |  |
|  | 141. Pastel o pay (rebanada gruesa) | 1 rebanada (56 g) |  |  |  |  |  |  |  |  |  |  |  |  |  |  |  |  |  |  |  |  |  |  |  |  |  |  |
|  | 142. Panes caseros | 1 rebanada (56 g) |  |  |  |  |  |  |  |  |  |  |  |  |  |  |  |  |  |  |  |  |  |  |  |  |  |  |
|  | 143. Papas fritas (tipo sabritas®, chips®, etc) | 1 bolsa (55 g) |  |  |  |  |  |  |  |  |  |  |  |  |  |  |  |  |  |  |  |  |  |  |  |  |  |  |
|  | 144. Botana de maíz (tipo doritos®, fritos®, etc) | 1 bolsa (58 g) |  |  |  |  |  |  |  |  |  |  |  |  |  |  |  |  |  |  |  |  |  |  |  |  |  |  |
|  | 145. Papas a la francesa o papas fritas caseras | 4 piezas (20 g) |  |  |  |  |  |  |  |  |  |  |  |  |  |  |  |  |  |  |  |  |  |  |  |  |  |  |
|  | 146. Churritos de trigo | 1 bolsa (50 g) |  |  |  |  |  |  |  |  |  |  |  |  |  |  |  |  |  |  |  |  |  |  |  |  |  |  |
|  | 147. Palomitas de maíz (caseras, microondas, cine) | ½ bolsa (40 g) |  |  |  |  |  |  |  |  |  |  |  |  |  |  |  |  |  |  |  |  |  |  |  |  |  |  |
| **Aceites con proteína** | 148. Aceite en spray (especificar de que tipo) | 5 disparos (5 g) |  |  |  |  |  |  |  |  |  |  |  |  |  |  |  |  |  |  |  |  |  |  |  |  |  |  |
|  | 149. Aceite de oliva (especificar si extra virgen u otro) | 1 cucharadita (5 g) |  |  |  |  |  |  |  |  |  |  |  |  |  |  |  |  |  |  |  |  |  |  |  |  |  |  |
|  | 150. Aceite de aguacate | 1 cucharadita (5 g) |  |  |  |  |  |  |  |  |  |  |  |  |  |  |  |  |  |  |  |  |  |  |  |  |  |  |
|  | 151. Aceite de maíz (p. ej. Cristal®, Mazola®) | 1 cucharadita (5 g) |  |  |  |  |  |  |  |  |  |  |  |  |  |  |  |  |  |  |  |  |  |  |  |  |  |  |
|  | 152. Aceite de coco | 1 cucharadita (5 g) |  |  |  |  |  |  |  |  |  |  |  |  |  |  |  |  |  |  |  |  |  |  |  |  |  |  |
|  | 153. Aceite de girasol (p. ej. 1 2 3®) | 1 cucharadita (5 g) |  |  |  |  |  |  |  |  |  |  |  |  |  |  |  |  |  |  |  |  |  |  |  |  |  |  |
|  | 154. Aceite de soya (p. ej. Nutrioloi®) | 1 cucharadita (5 g) |  |  |  |  |  |  |  |  |  |  |  |  |  |  |  |  |  |  |  |  |  |  |  |  |  |  |
|  | 155. Aceite de canola (p. ej. Canoil®, Capullo®) | 1 cucharadita (5 g) |  |  |  |  |  |  |  |  |  |  |  |  |  |  |  |  |  |  |  |  |  |  |  |  |  |  |
|  | 156. Aceite de cártamo (p. ej. Oléico®, | 1 cucharadita (5 g) |  |  |  |  |  |  |  |  |  |  |  |  |  |  |  |  |  |  |  |  |  |  |  |  |  |  |
|  | 157. Aceitunas | 8 piezas (24 g) |  |  |  |  |  |  |  |  |  |  |  |  |  |  |  |  |  |  |  |  |  |  |  |  |  |  |
|  | 158. Coco picado | 1 tira (16 g) |  |  |  |  |  |  |  |  |  |  |  |  |  |  |  |  |  |  |  |  |  |  |  |  |  |  |
|  | 159. Aguacate | ½ pieza (46 g) |  |  |  |  |  |  |  |  |  |  |  |  |  |  |  |  |  |  |  |  |  |  |  |  |  |  |
|  | 160. Mayonesa, aderezo o vinagreta | 1 cucharadita (5 g) |  |  |  |  |  |  |  |  |  |  |  |  |  |  |  |  |  |  |  |  |  |  |  |  |  |  |
|  | 161. Margarina | 1 cucharadita (5 g) |  |  |  |  |  |  |  |  |  |  |  |  |  |  |  |  |  |  |  |  |  |  |  |  |  |  |
|  | 162. Mantequilla | 1 cucharadita (5 g) |  |  |  |  |  |  |  |  |  |  |  |  |  |  |  |  |  |  |  |  |  |  |  |  |  |  |
|  | 163. Manteca de cerdo | 1 cucharadita (5 g) |  |  |  |  |  |  |  |  |  |  |  |  |  |  |  |  |  |  |  |  |  |  |  |  |  |  |
|  | 164. Manteca vegetal | 1 cucharadita (5 g) |  |  |  |  |  |  |  |  |  |  |  |  |  |  |  |  |  |  |  |  |  |  |  |  |  |  |
|  | 165. Queso crema | 1 cucharada (15 g) |  |  |  |  |  |  |  |  |  |  |  |  |  |  |  |  |  |  |  |  |  |  |  |  |  |  |
|  | 166. Crema, nata o jocoque | 1 cucharada (15 g) |  |  |  |  |  |  |  |  |  |  |  |  |  |  |  |  |  |  |  |  |  |  |  |  |  |  |
| **Azúcares con y sin grasa**  **Leguminosas**  **Cereales sin grasa** | 167. Azúcar | 1 cucharadita (4 g) |  |  |  |  |  |  |  |  |  |  |  |  |  |  |  |  |  |  |  |  |  |  |  |  |  |  |
|  | 168. Miel de abeja | 1 cucharadita (7 g) |  |  |  |  |  |  |  |  |  |  |  |  |  |  |  |  |  |  |  |  |  |  |  |  |  |  |
|  | 169. Miel o jarabe de maple | 1 cucharadita (7 g) |  |  |  |  |  |  |  |  |  |  |  |  |  |  |  |  |  |  |  |  |  |  |  |  |  |  |
|  | 170. Cajeta | 1 cucharada (15 g) |  |  |  |  |  |  |  |  |  |  |  |  |  |  |  |  |  |  |  |  |  |  |  |  |  |  |
|  | 171. Crema de avellanas (Por ejemplo, Nutella®) | 1 cucharada (15 g) |  |  |  |  |  |  |  |  |  |  |  |  |  |  |  |  |  |  |  |  |  |  |  |  |  |  |
|  | 172. Leche condensada (Por ejemplo Lechera®) | 1 cucharada (15 g) |  |  |  |  |  |  |  |  |  |  |  |  |  |  |  |  |  |  |  |  |  |  |  |  |  |  |
|  | 173. Piloncillo | 1 porción (10 g) |  |  |  |  |  |  |  |  |  |  |  |  |  |  |  |  |  |  |  |  |  |  |  |  |  |  |
|  | 174. Mermeladas | 1 cucharada (17 g) |  |  |  |  |  |  |  |  |  |  |  |  |  |  |  |  |  |  |  |  |  |  |  |  |  |  |
|  | 175. Dulces envasados (paletas, dulces macizos, gomitas, pulparindo®, bubulubu®, etc) | 1 - 5 piezas (20 g) |  |  |  |  |  |  |  |  |  |  |  |  |  |  |  |  |  |  |  |  |  |  |  |  |  |  |
|  | 176. Chocolates | 1 barra chica (15 g) |  |  |  |  |  |  |  |  |  |  |  |  |  |  |  |  |  |  |  |  |  |  |  |  |  |  |
|  | 177. Chocomilk® o cacao en polvo | 1 cucharada (15 g) |  |  |  |  |  |  |  |  |  |  |  |  |  |  |  |  |  |  |  |  |  |  |  |  |  |  |
|  | 178. Helado o paleta helada de crema | 1 bola/pieza (96 g) |  |  |  |  |  |  |  |  |  |  |  |  |  |  |  |  |  |  |  |  |  |  |  |  |  |  |
|  | 179. Helado o paleta helada de agua | 1 bola/pieza (65 g) |  |  |  |  |  |  |  |  |  |  |  |  |  |  |  |  |  |  |  |  |  |  |  |  |  |  |
|  | 180. Tapioca (cocida en caramelo o en leche) | ½ taza (147 g) |  |  |  |  |  |  |  |  |  |  |  |  |  |  |  |  |  |  |  |  |  |  |  |  |  |  |
|  | 181. Gelatina en agua o en leche (especificar) | 1 pieza (125 ml) |  |  |  |  |  |  |  |  |  |  |  |  |  |  |  |  |  |  |  |  |  |  |  |  |  |  |
|  | 182. Dulces mexicanos (Ate, cocada, mazapán, palanqueta (cacahuate o nuez), dulce de leche) | 1 pieza (28 g) |  |  |  |  |  |  |  |  |  |  |  |  |  |  |  |  |  |  |  |  |  |  |  |  |  |  |
|  | 183. Plátanos fritos o enmielados | 1 pieza (123.5 g) |  |  |  |  |  |  |  |  |  |  |  |  |  |  |  |  |  |  |  |  |  |  |  |  |  |  |
| **Comida rápida** | 184. Pizza | 1 rebanada (100 g) |  |  |  |  |  |  |  |  |  |  |  |  |  |  |  |  |  |  |  |  |  |  |  |  |  |  |
|  | 185. Hamburguesa | 1 pieza (235 g) |  |  |  |  |  |  |  |  |  |  |  |  |  |  |  |  |  |  |  |  |  |  |  |  |  |  |
|  | 186. Hot dog | 1 pieza (156 g) |  |  |  |  |  |  |  |  |  |  |  |  |  |  |  |  |  |  |  |  |  |  |  |  |  |  |
|  | 187. Sándwich (especificar si de jamón u otro y si pan integral o blanco) | 1 pieza (128 g) |  |  |  |  |  |  |  |  |  |  |  |  |  |  |  |  |  |  |  |  |  |  |  |  |  |  |
|  | 188. Sopas instantáneas | 1 pieza (64 g) |  |  |  |  |  |  |  |  |  |  |  |  |  |  |  |  |  |  |  |  |  |  |  |  |  |  |
|  | 189. Sushi | 4 rollitos (75 g) |  |  |  |  |  |  |  |  |  |  |  |  |  |  |  |  |  |  |  |  |  |  |  |  |  |  |
|  | 190. Alitas de pollo | 1 alita (51 g) |  |  |  |  |  |  |  |  |  |  |  |  |  |  |  |  |  |  |  |  |  |  |  |  |  |  |
|  | 191. Baguette o panini | 1 pieza (208 g) |  |  |  |  |  |  |  |  |  |  |  |  |  |  |  |  |  |  |  |  |  |  |  |  |  |  |
|  | 192. Sopas y cremas de sobre | 1 plato (100 g) |  |  |  |  |  |  |  |  |  |  |  |  |  |  |  |  |  |  |  |  |  |  |  |  |  |  |
| **Comida mexicana** | 193. Chilaquiles con carne de res | 1 taza (160 g) |  |  |  |  |  |  |  |  |  |  |  |  |  |  |  |  |  |  |  |  |  |  |  |  |  |  |
|  | 193. Chilaquiles con huevo y/o pollo pero sin carne res | 1 taza (160 g) |  |  |  |  |  |  |  |  |  |  |  |  |  |  |  |  |  |  |  |  |  |  |  |  |  |  |
|  | 193.1 Huevo a la mexicana (jitomate, cebolla, chile, nopal) | 1 plato (220 g) |  |  |  |  |  |  |  |  |  |  |  |  |  |  |  |  |  |  |  |  |  |  |  |  |  |  |
|  | 194. Menudo de res | 1 plato (300 g) |  |  |  |  |  |  |  |  |  |  |  |  |  |  |  |  |  |  |  |  |  |  |  |  |  |  |
|  | 195. Quesadillas de tortilla de maíz sin carnes | 1 pieza (60 g) |  |  |  |  |  |  |  |  |  |  |  |  |  |  |  |  |  |  |  |  |  |  |  |  |  |  |
|  | 195.1 Quesadillas de tortilla de maíz con carnes | 1 pieza (60 g) |  |  |  |  |  |  |  |  |  |  |  |  |  |  |  |  |  |  |  |  |  |  |  |  |  |  |
|  | 196. Quesadillas de tortilla de harina | 1 pieza (60 g) |  |  |  |  |  |  |  |  |  |  |  |  |  |  |  |  |  |  |  |  |  |  |  |  |  |  |
|  | 197. Tortas (adobada, carne, ahogada) | 1 pieza (197 g) |  |  |  |  |  |  |  |  |  |  |  |  |  |  |  |  |  |  |  |  |  |  |  |  |  |  |
|  | 197.1 Torta de panela o frijoles (no de carne) | 1 pieza (197 g) |  |  |  |  |  |  |  |  |  |  |  |  |  |  |  |  |  |  |  |  |  |  |  |  |  |  |
|  | 198. Tamal de carne (res, cerdo, pollo) | 1 pieza (200 g) |  |  |  |  |  |  |  |  |  |  |  |  |  |  |  |  |  |  |  |  |  |  |  |  |  |  |
|  | 198.1 Tamal de pollo | 1 pieza (200 g) |  |  |  |  |  |  |  |  |  |  |  |  |  |  |  |  |  |  |  |  |  |  |  |  |  |  |
|  | 198.2 Tamal de queso | 1 pieza (200 g) |  |  |  |  |  |  |  |  |  |  |  |  |  |  |  |  |  |  |  |  |  |  |  |  |  |  |
|  | 198.3 Tamal de rajas o ceniza (frijoles) | 1 pieza (200 g) |  |  |  |  |  |  |  |  |  |  |  |  |  |  |  |  |  |  |  |  |  |  |  |  |  |  |
|  | 199. Tamal dulce (elote, frutas) | 1 pieza (172 g) |  |  |  |  |  |  |  |  |  |  |  |  |  |  |  |  |  |  |  |  |  |  |  |  |  |  |
|  | 200. Mole con pollo | 1 plato (334 g) |  |  |  |  |  |  |  |  |  |  |  |  |  |  |  |  |  |  |  |  |  |  |  |  |  |  |
|  | 201. Pozole de cerdo | 1 plato (300 g) |  |  |  |  |  |  |  |  |  |  |  |  |  |  |  |  |  |  |  |  |  |  |  |  |  |  |
|  | 201.1 Pozole de pollo | 1 plato (300 g) |  |  |  |  |  |  |  |  |  |  |  |  |  |  |  |  |  |  |  |  |  |  |  |  |  |  |
|  | 201.2 Pozole vegetariano (champiñones) | 1 plato (300 g) |  |  |  |  |  |  |  |  |  |  |  |  |  |  |  |  |  |  |  |  |  |  |  |  |  |  |
|  | 202. Sopitos con carne de res o cerdo | 1 pieza (75 g) |  |  |  |  |  |  |  |  |  |  |  |  |  |  |  |  |  |  |  |  |  |  |  |  |  |  |
|  | 202.1 Sopitos de frijoles o pollo (no carne roja) | 1 pieza (75 g) |  |  |  |  |  |  |  |  |  |  |  |  |  |  |  |  |  |  |  |  |  |  |  |  |  |  |
|  | 202.1 Enchiladas con carne de res o cerdo | 1 pieza (75 g) |  |  |  |  |  |  |  |  |  |  |  |  |  |  |  |  |  |  |  |  |  |  |  |  |  |  |
|  | 202.2 Enchilada de pollo o queso (no carne roja) | 1 pieza (75 g) |  |  |  |  |  |  |  |  |  |  |  |  |  |  |  |  |  |  |  |  |  |  |  |  |  |  |
|  | 202.2 Flautas con carne de res o cerdo | 1 pieza (75 g) |  |  |  |  |  |  |  |  |  |  |  |  |  |  |  |  |  |  |  |  |  |  |  |  |  |  |
|  | 202.3 Flautas de pollo o queso (no carne roja) | 1 pieza (75 g) |  |  |  |  |  |  |  |  |  |  |  |  |  |  |  |  |  |  |  |  |  |  |  |  |  |  |
|  | 203. Sopas, caldos o cremas de vegetales naturales | 1 plato (393 g) |  |  |  |  |  |  |  |  |  |  |  |  |  |  |  |  |  |  |  |  |  |  |  |  |  |  |
|  | 204. Tacos de res | 1 pieza (75 g) |  |  |  |  |  |  |  |  |  |  |  |  |  |  |  |  |  |  |  |  |  |  |  |  |  |  |
|  | 204.1 Tacos de cerdo (adobada, al pastor) | 1 pieza (75 g) |  |  |  |  |  |  |  |  |  |  |  |  |  |  |  |  |  |  |  |  |  |  |  |  |  |  |
|  | 204.2 Taco de frijoles | 1 pieza (60 g) |  |  |  |  |  |  |  |  |  |  |  |  |  |  |  |  |  |  |  |  |  |  |  |  |  |  |
|  | 204.3 Taco de pollo o pescado | 1 pieza (75 g) |  |  |  |  |  |  |  |  |  |  |  |  |  |  |  |  |  |  |  |  |  |  |  |  |  |  |
|  | 205. Burrito de carne | 1 pieza (100 g) |  |  |  |  |  |  |  |  |  |  |  |  |  |  |  |  |  |  |  |  |  |  |  |  |  |  |
|  | 205.1 Burrito de frijoles sin carne | 1 pieza (100 g) |  |  |  |  |  |  |  |  |  |  |  |  |  |  |  |  |  |  |  |  |  |  |  |  |  |  |
|  | 205.2 Tostada con carne de res o cerdo | 1 tostada grande (174 g) |  |  |  |  |  |  |  |  |  |  |  |  |  |  |  |  |  |  |  |  |  |  |  |  |  |  |
|  | 205.2 Tostada con frijoles o pollo | 1 tostada grande (174 g) |  |  |  |  |  |  |  |  |  |  |  |  |  |  |  |  |  |  |  |  |  |  |  |  |  |  |
|  | 206. Arroz con leche | ½ taza (178 g) |  |  |  |  |  |  |  |  |  |  |  |  |  |  |  |  |  |  |  |  |  |  |  |  |  |  |
|  | 207. Flan, jericalla, natilla | 1 pieza (120 ml) |  |  |  |  |  |  |  |  |  |  |  |  |  |  |  |  |  |  |  |  |  |  |  |  |  |  |
|  | 208. Capirotada | 1 rebanada (60 g) |  |  |  |  |  |  |  |  |  |  |  |  |  |  |  |  |  |  |  |  |  |  |  |  |  |  |
|  | 209. Churros rellenos o azucarados | 1 churro (90 g) |  |  |  |  |  |  |  |  |  |  |  |  |  |  |  |  |  |  |  |  |  |  |  |  |  |  |
|  | 210. Atole de maíz en agua | 1 taza (240 ml) |  |  |  |  |  |  |  |  |  |  |  |  |  |  |  |  |  |  |  |  |  |  |  |  |  |  |
|  | 211. Atole de maíz en leche o champurrado | 1 taza (240 ml) |  |  |  |  |  |  |  |  |  |  |  |  |  |  |  |  |  |  |  |  |  |  |  |  |  |  |
| **Condimentos** | 212. Salsa cátsup o puré de tomate enlatado | 1 cucharada (15 g) |  |  |  |  |  |  |  |  |  |  |  |  |  |  |  |  |  |  |  |  |  |  |  |  |  |  |
|  | 213. Salsa picante embotellada | 1 cucharadita (6 g) |  |  |  |  |  |  |  |  |  |  |  |  |  |  |  |  |  |  |  |  |  |  |  |  |  |  |
|  | 214. Mostaza | 1 cucharadita (5 g) |  |  |  |  |  |  |  |  |  |  |  |  |  |  |  |  |  |  |  |  |  |  |  |  |  |  |
|  | 215. Sal | 1 pizca (2 g) |  |  |  |  |  |  |  |  |  |  |  |  |  |  |  |  |  |  |  |  |  |  |  |  |  |  |
|  | 216. Salsa de soya | 1 cucharadita (5 ml) |  |  |  |  |  |  |  |  |  |  |  |  |  |  |  |  |  |  |  |  |  |  |  |  |  |  |
|  | 217. Vainilla, pimienta, canela, jengibre, cúrcuma, curry, clavo de olor, anís | 1 cucharadita (5 g) |  |  |  |  |  |  |  |  |  |  |  |  |  |  |  |  |  |  |  |  |  |  |  |  |  |  |
|  | 218. Crema para café | 1 cucharadita (5 g) |  |  |  |  |  |  |  |  |  |  |  |  |  |  |  |  |  |  |  |  |  |  |  |  |  |  |
| **Bebidas** | 219. Refresco o bebida con azúcar | 1 botella (600 ml) |  |  |  |  |  |  |  |  |  |  |  |  |  |  |  |  |  |  |  |  |  |  |  |  |  |  |
|  | 220. Refresco o bebida sin azúcar o light | 1 botella (600 ml) |  |  |  |  |  |  |  |  |  |  |  |  |  |  |  |  |  |  |  |  |  |  |  |  |  |  |
|  | 221. Agua fresca de frutas con azúcar | 1 taza (240 ml) |  |  |  |  |  |  |  |  |  |  |  |  |  |  |  |  |  |  |  |  |  |  |  |  |  |  |
|  | 221.1 Agua fresca de frutas sin azúcar | 1 taza (240 ml) |  |  |  |  |  |  |  |  |  |  |  |  |  |  |  |  |  |  |  |  |  |  |  |  |  |  |
|  | 222. Jugo de fruta natural (naranja, toronja) | 1 taza (240 ml) |  |  |  |  |  |  |  |  |  |  |  |  |  |  |  |  |  |  |  |  |  |  |  |  |  |  |
|  | 223. Jugo de fruta industrializado | 1 botella (240 ml) |  |  |  |  |  |  |  |  |  |  |  |  |  |  |  |  |  |  |  |  |  |  |  |  |  |  |
|  | 224. Bebida para deportista (Gatorade®, etc) | 1 botella (600 ml) |  |  |  |  |  |  |  |  |  |  |  |  |  |  |  |  |  |  |  |  |  |  |  |  |  |  |
|  | 225. Tejuino | 2 tazas (418 ml) |  |  |  |  |  |  |  |  |  |  |  |  |  |  |  |  |  |  |  |  |  |  |  |  |  |  |
|  | 225.1 Tuba | 1 taza (240 ml) |  |  |  |  |  |  |  |  |  |  |  |  |  |  |  |  |  |  |  |  |  |  |  |  |  |  |
|  | 225.2 Raspados | 2 tazas (418 ml) |  |  |  |  |  |  |  |  |  |  |  |  |  |  |  |  |  |  |  |  |  |  |  |  |  |  |
|  | 226. Café soluble o americano | 1 taza (240 ml) |  |  |  |  |  |  |  |  |  |  |  |  |  |  |  |  |  |  |  |  |  |  |  |  |  |  |
|  | 227. Café de olla | 1 taza (240 ml) |  |  |  |  |  |  |  |  |  |  |  |  |  |  |  |  |  |  |  |  |  |  |  |  |  |  |
|  | 228. Capuchinos o lattes | 1 taza (240 ml) |  |  |  |  |  |  |  |  |  |  |  |  |  |  |  |  |  |  |  |  |  |  |  |  |  |  |
|  | 229. Frapuchinos o malteadas con leche | 2 tazas (480 ml) |  |  |  |  |  |  |  |  |  |  |  |  |  |  |  |  |  |  |  |  |  |  |  |  |  |  |
|  | 230. Chocolate en agua | 1 taza (240 ml) |  |  |  |  |  |  |  |  |  |  |  |  |  |  |  |  |  |  |  |  |  |  |  |  |  |  |
|  | 231. Chocolate en leche | 1 taza (240 ml) |  |  |  |  |  |  |  |  |  |  |  |  |  |  |  |  |  |  |  |  |  |  |  |  |  |  |
|  | 232. Té o canela | 1 taza (240 ml) |  |  |  |  |  |  |  |  |  |  |  |  |  |  |  |  |  |  |  |  |  |  |  |  |  |  |
|  | 233. Agua natural | 1 taza (240 ml) |  |  |  |  |  |  |  |  |  |  |  |  |  |  |  |  |  |  |  |  |  |  |  |  |  |  |
| **Bebidas alcohólicas** | 234. Vino tinto | 1 copa (100 ml) |  |  |  |  |  |  |  |  |  |  |  |  |  |  |  |  |  |  |  |  |  |  |  |  |  |  |
|  | 235. Vino blanco o rosado | 1 copa (100 ml) |  |  |  |  |  |  |  |  |  |  |  |  |  |  |  |  |  |  |  |  |  |  |  |  |  |  |
|  | 236. Cerveza | 1 lata o botella (356 ml) |  |  |  |  |  |  |  |  |  |  |  |  |  |  |  |  |  |  |  |  |  |  |  |  |  |  |
|  | 237. Destilados: whisky, vodka, ginebra, coñac, tequila, mezcal, ron, brandy | 1 caballito (60 ml) |  |  |  |  |  |  |  |  |  |  |  |  |  |  |  |  |  |  |  |  |  |  |  |  |  |  |
|  | 238. Ponche (granada, frambuesa) | 1 copa (80 ml) |  |  |  |  |  |  |  |  |  |  |  |  |  |  |  |  |  |  |  |  |  |  |  |  |  |  |
|  | 239. Rompope | 1 copa (80 ml) |  |  |  |  |  |  |  |  |  |  |  |  |  |  |  |  |  |  |  |  |  |  |  |  |  |  |
|  | 240. Pulque, tepache o agua miel | 1 copa (80 ml) |  |  |  |  |  |  |  |  |  |  |  |  |  |  |  |  |  |  |  |  |  |  |  |  |  |  |
| **Otros** | 241. Suplemento proteico | 1 scoop (40 g) |  |  |  |  |  |  |  |  |  |  |  |  |  |  |  |  |  |  |  |  |  |  |  |  |  |  |
|  | 242. Estevia | 1 sobre (3 g) |  |  |  |  |  |  |  |  |  |  |  |  |  |  |  |  |  |  |  |  |  |  |  |  |  |  |
|  | 243. Splenda® o Canderel® | 1 sobre (3 g) |  |  |  |  |  |  |  |  |  |  |  |  |  |  |  |  |  |  |  |  |  |  |  |  |  |  |
|  | 244. Suplementos multivitamínicos (colocar marca) | 1 cápsula |  |  |  |  |  |  |  |  |  |  |  |  |  |  |  |  |  |  |  |  |  |  |  |  |  |  |
| **Alimentos fermentados** | 245. Kéfir | 1 taza (240 ml) |  |  |  |  |  |  |  |  |  |  |  |  |  |  |  |  |  |  |  |  |  |  |  |  |  |  |
|  | 246. Kombucha (Té fermentado) | 1 taza (240 ml) |  |  |  |  |  |  |  |  |  |  |  |  |  |  |  |  |  |  |  |  |  |  |  |  |  |  |
|  | 247. Tempeh (soya fermentada) | 1 taza (240 ml) |  |  |  |  |  |  |  |  |  |  |  |  |  |  |  |  |  |  |  |  |  |  |  |  |  |  |
|  | 248. Natto (soya fermentada) | 1 taza (240 ml) |  |  |  |  |  |  |  |  |  |  |  |  |  |  |  |  |  |  |  |  |  |  |  |  |  |  |
|  | 249. Miso (Pasta de soya fermentada) | 1 taza (240 ml) |  |  |  |  |  |  |  |  |  |  |  |  |  |  |  |  |  |  |  |  |  |  |  |  |  |  |
|  | 250. Kimchi (Vegetales fermentados) | 1 taza (240 ml) |  |  |  |  |  |  |  |  |  |  |  |  |  |  |  |  |  |  |  |  |  |  |  |  |  |  |
|  | 251. Pan sourdough (pan de masa madre) | 1 taza (240 ml) |  |  |  |  |  |  |  |  |  |  |  |  |  |  |  |  |  |  |  |  |  |  |  |  |  |  |
| Nota: los alimentos coloreados de azul corresponden a aquellos que serán incluidos en el programa de intervención y corresponden a los presentados en la Tabla A6.5, donde se especifica la frecuencia y cantidad de consumo a promover. | 252. Saurkraut (Col fermentada) | 1 taza (240 ml) |  |  |  |  |  |  |  |  |  |  |  |  |  |  |  |  |  |  |  |  |  |  |  |  |  |  |

**Online Supplementary Material 1.7. Primers for bacteria identification in the qPCR analysis**

Table SM1.7. Primers for bacteria identification in the qPCR analysis

| Bacteria | Name | Primer  (5’-3’) | Number of bases | Sequence | Notes | Reference |
| --- | --- | --- | --- | --- | --- | --- |
| Firmicutes | 928F-Firm | Forward | 21 | 5'-TGAAACTCAAAGGAATTGACG-3' | Y = C or T 🡪 C | Chávez-Carbajal et al. (2019) y De Gregoris et al. (2011) |
|  | 1040FirmR | Reverse | 17 | 5'-ACCATGCACCACCTGTC-3' |  |  |
| Bacteroidetes | 798cfbF | Forward | 20 | 5'-CAAACAGGATTAGATACCCT-3' | R = A or G 🡪 A |  |
|  | Cfb967R | Reverse | 19 | 5'-GGTAAGGTTCCTCGCGTAT-3' |  |  |
| Universal | 926F | Forward | 20 | 5'-AAACTCAAAGGAATTGACGG-3' | K = G or T 🡪 G |  |
|  | 1062R | Reverse | 18 | 5'-CTCACAACACGAGCTGAC-3' | R = A or G 🡪 A |  |
| *Lactobacillus* |  | Forward | 19 | 5'-AGCAGTAGGGAATCTTCCA-3' |  | Avila‐Nava et al. (2017) |
|  |  | Reverse | 17 | 5'-CACCGCTACACATGGAG-3' |  |  |
| *Bifidobacterium* |  | Forward | 18 | 5'-TCGCGTCCGGTGTGAAAG-3' | C |  |
|  |  | Reverse | 17 | 5'-CCACATCCAGCATCCAC-3' | A |  |
| *Faecalibacterium prausnitzii* | FPR-2F | Forward | 19 | 5'-GGAGGAAGAAGGTCTTCGG-3' | Amplicon 248 | Ramirez-Farias et al. (2008) |
|  | Fprau645R | Reverse | 21 | 5'-AATTCCGCCTACCTCTGCACT-3' |  |  |
| *Akkermansia muciniphila* |  | Forward | 20 | 5’-CAGCACGTGAAGGTGGGGAC-3’ |  | Dao et al. (2016) |
|  |  | Reverse | 20 | 5’-CCTTGCGGTTGGCTTCAGAT-3’ |  |  |
| *Prevotella copri* |  | Forward | 20 | 5'-CCGGACTCCTGCCCCTGCAA-3' |  | Scher et al. (2013) |
|  |  | Reverse | 20 | 5'-GTTGCGCCAGGCACTGCGAT-3' |  |  |
| *Bilophila wadsworthia* |  | Forward | 26 | 5'-CAACGTCCCCACCATCAAGTTCTCTG-3' |  | Laue et al. (2006) |
|  |  | Reverse | 26 | 5'-TGAATTCGCGGAAGGAGCGAGAGGTC-3' |  |  |
| *Clostridium coccoides* (*Eubacterium* rectale group and *Lachnospiraceae* family) |  | Forward | 19 | 5’-CGGTACCTGACTAAGAAGC-3’ | 429 pb | Rodríguez Lara (2022) |
|  |  | Reverse | 19 | 5’-AGTTTCATTCTTGCGAACG-3’ | 429 pb |  |
| *Streptococcus thermophilus* | St1 | Forward | 22 | 5'-TTATTTGAAAGGGGCAATTGCT-3' | Posición: 195–216 | Furet et al. (2004) |
|  | St2 | Reverse | 21 | 5'-GTGAACTTTCCACTCTCACAC-3' | Posición: 474–454 |  |

Bases modified according to “Re: What are Y, M, W and D in primer design?”, by A. Gund, Nadine, 2014. <https://www.researchgate.net/post/What_are_Y_M_W_and_D_in_primer_design/538c1577d11b8ba27a8b45df/citation/download>.

**References of Table SM1.7.**

Avila-Nava, A., Noriega, L. G., Tovar, A. R., Granados, O., Perez‐Cruz, C., Pedraza‐Chaverri, J., & Torres, N. (2017). Food combination based on a pre‐hispanic Mexican diet decreases metabolic and cognitive abnormalities and gut microbiota dysbiosis caused by a sucrose‐enriched high‐fat diet in rats. Molecular Nutrition & Food Research, 61(1), 1501023. 10.1002/mnfr.201501023

Chávez-Carbajal, A., Nirmalkar, K., Pérez-Lizaur, A., Hernández-Quiroz, F., Ramírez-del-Alto, S., García-Mena, J., & Hernández-Guerrero, C. (2019). Gut microbiota and predicted metabolic pathways in a sample of Mexican women affected by obesity and obesity plus metabolic syndrome. International Journal of Molecular Sciences, 20(438), 1-18. 10.3390/ijms20020438

Dao, M. C., Everard, A., Aron-Wisnewsky, J., Sokolovska, N., Prifti, E., Verger, E. O., Kayser, B. D., Levenez, F., Chilloux, J., Hoyles, L., MICRO-Obes Consortium, Dumas, M. E., Rizkalla, S. W., Doré, J., Cani, P. D., & Clément, K. (2016). Akkermansia muciniphila and improved metabolic health during a dietary intervention in obesity: Relationship with gut microbiome richness and ecology. Gut, 65(3), 426-436. 10.1136/gutjnl-2014-308778

De Gregoris, T. B., Aldred, N., Clare, A. S., & Burgess, J. G. (2011). Improvement of phylum-and class-specific primers for real-time PCR quantification of bacterial taxa. Journal of Microbiological Methods, 86(3), 351-356. https://doi.org/10.1016/j.mimet.2011.06.010

Furet, J. P., Quénée, P., & Tailliez, P. (2004). Molecular quantification of lactic acid bacteria in fermented milk products using real-time quantitative PCR. International Journal of Food Microbiology, 97(2), 197-207. 10.1016/j.ijfoodmicro.2004.04.020

Laue, H., Smits, T. H., Schumacher, U. K., Claros, M. C., Hartemink, R., & Cook, A. M. (2006). Identification of Bilophila wadsworthia by specific PCR which targets the taurine: pyruvate aminotransferase gene. FEMS Microbiology Letters, 261(1), 74-79. 10.1111/j.1574-6968.2006.00335.x

Ramirez-Farias, C., Slezak, K., Fuller, Z., Duncan, A., Holtrop, G., & Louis, P. (2008). Effect of inulin on the human gut microbiota: stimulation of Bifidobacterium adolescentis and Faecalibacterium prausnitzii. British Journal of Nutrition, 101(4), 541-550.

Rodríguez-Lara, A., Plaza-Díaz, J., López-Uriarte, P., Vázquez-Aguilar, A., Reyes-Castillo, Z., & Álvarez-Mercado, A. I. (2022). Fiber consumption mediates differences in several gut microbes in a subpopulation of young mexican adults. Nutrients, 14(6), 1-15. https://doi.org/10.3390/nu14061214

Scher, J. U., Sczesnak, A., Longman, R. S., Segata, N., Ubeda, C., Bielski, C., Rostron, T., Cerundolo, V., Pamer, E. G., Abramson, S. B., Huttenhower, C., & Littman, D. R. (2013). Expansion of intestinal Prevotella copri correlates with enhanced susceptibility to arthritis. Elife, 5(2), 1-20. 10.7554/eLife.01202.001

Online Supplementary Material 1.8. Nutritional-sustainable knowledge questionnaire

English version of the questionnaire is available at: <https://forms.gle/LwrCuwP348iNJJqx5>

For complete written version, see online supplementary material 6.


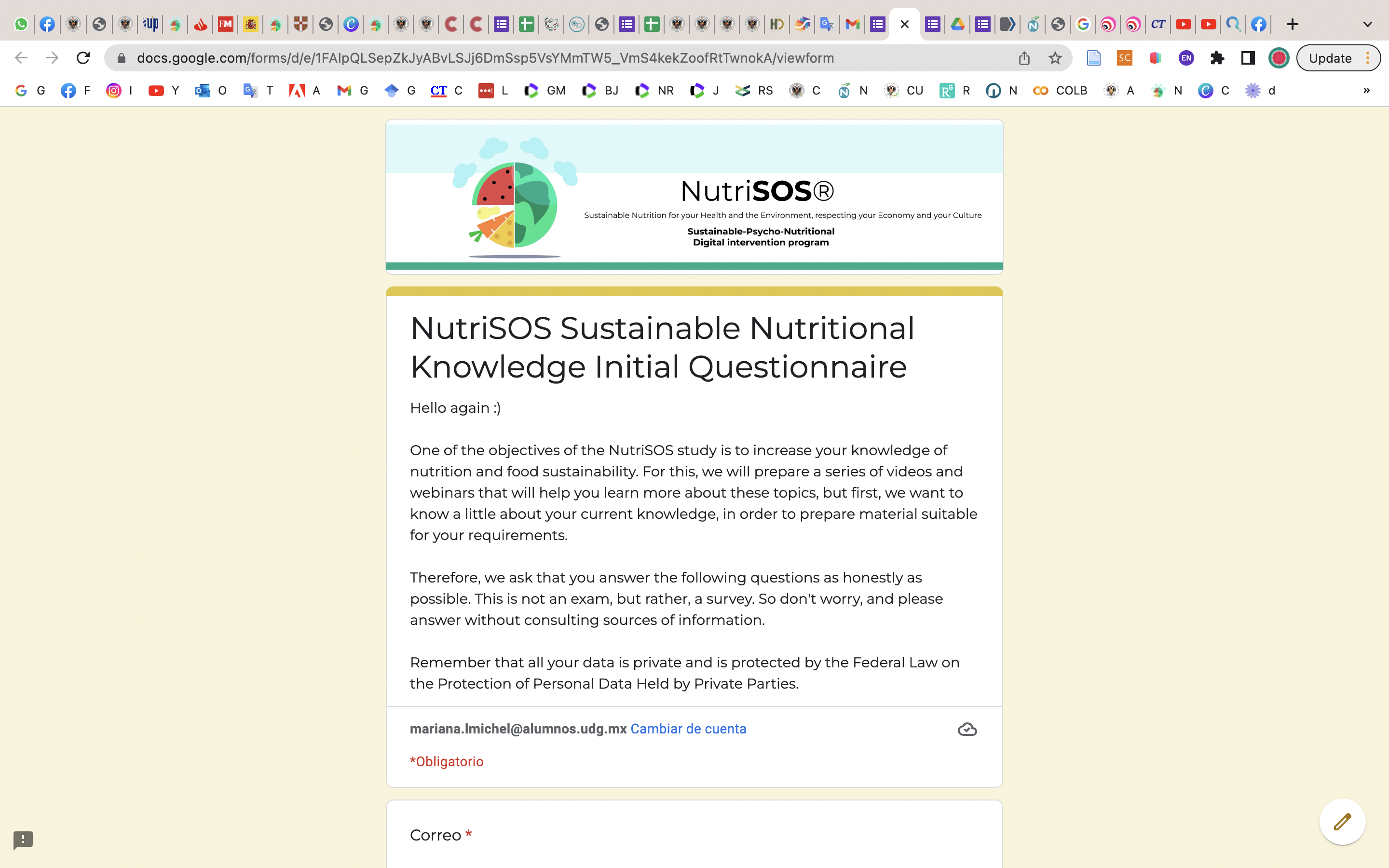


Figure SM1.8.1. Nutritional-sustainable knowledge questionnaire (English version)

Nutritional-sustainable knowledge questionnaire

Spanish version of the questionnaire is available at: <https://forms.gle/JLTjPGzwqVz1ChQ6A>

For complete written version, see online supplementary material 7


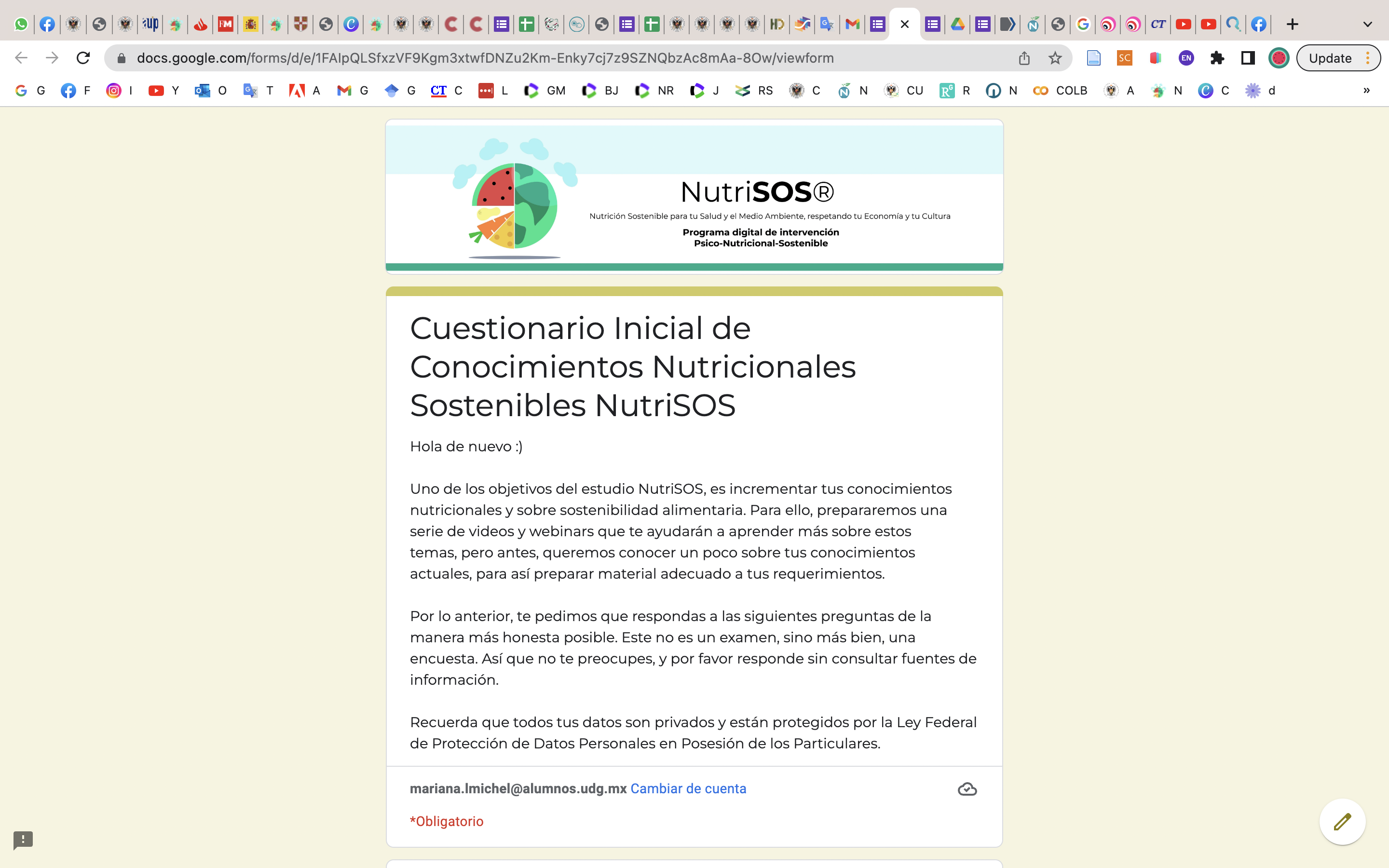


Figure SM1.8.2. Nutritional-sustainable knowledge questionnaire (Spanish version)

Online Supplementary Material 1.9. COM-B questionnaire

COM-B questionnaire

English version of the questionnaire is available at: <https://forms.gle/GmDydHz2cNomSuss5>

For complete written version, see online supplementary material 8.


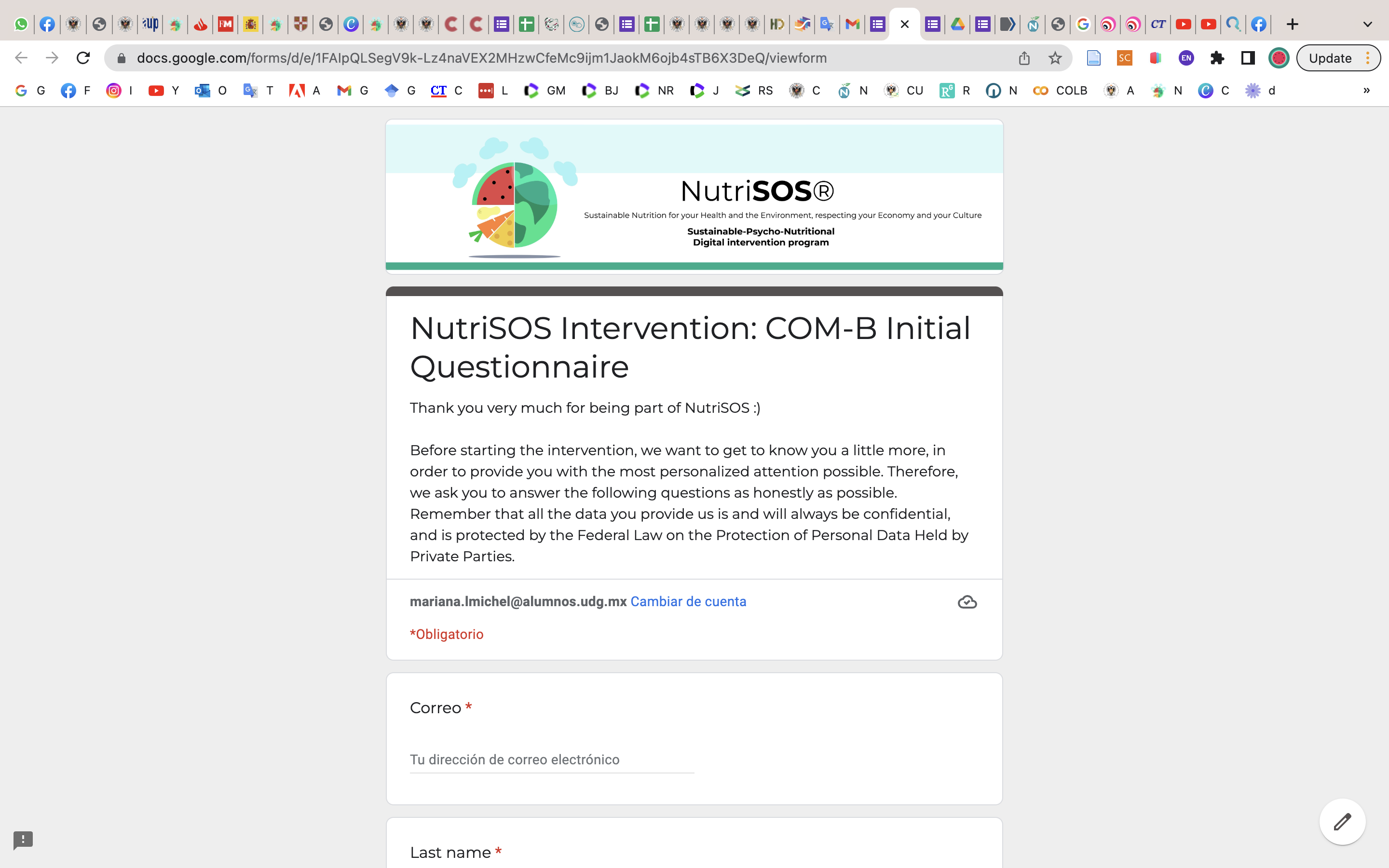


Figure SM1.9.1 COM-B questionnaire (English version)

COM-B questionnaire

Spanish version of the questionnaire is available at: <https://forms.gle/xiPi16x5ePgJxXtDA>

For complete written version, see online supplementary material 9.


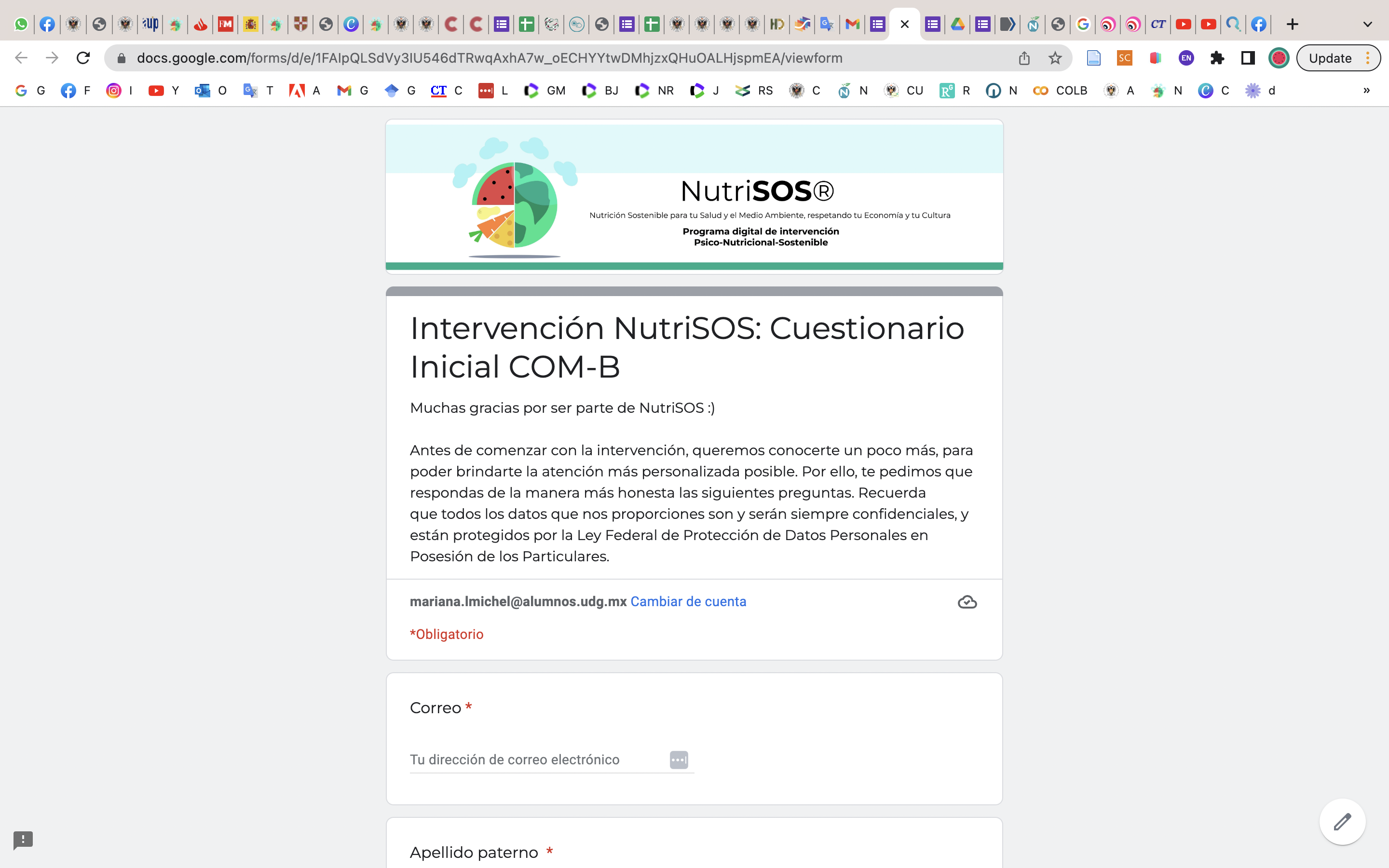


Figure SM1.9.2 COM-B questionnaire (Spanish version)

Online Supplementary Material 1.10. Adherence questionnaire

Adherence questionnaire

English version of the questionnaire is available at: <https://forms.gle/qT5hEr3QyXCcDmm7A>

For complete written version, see online supplementary material 10


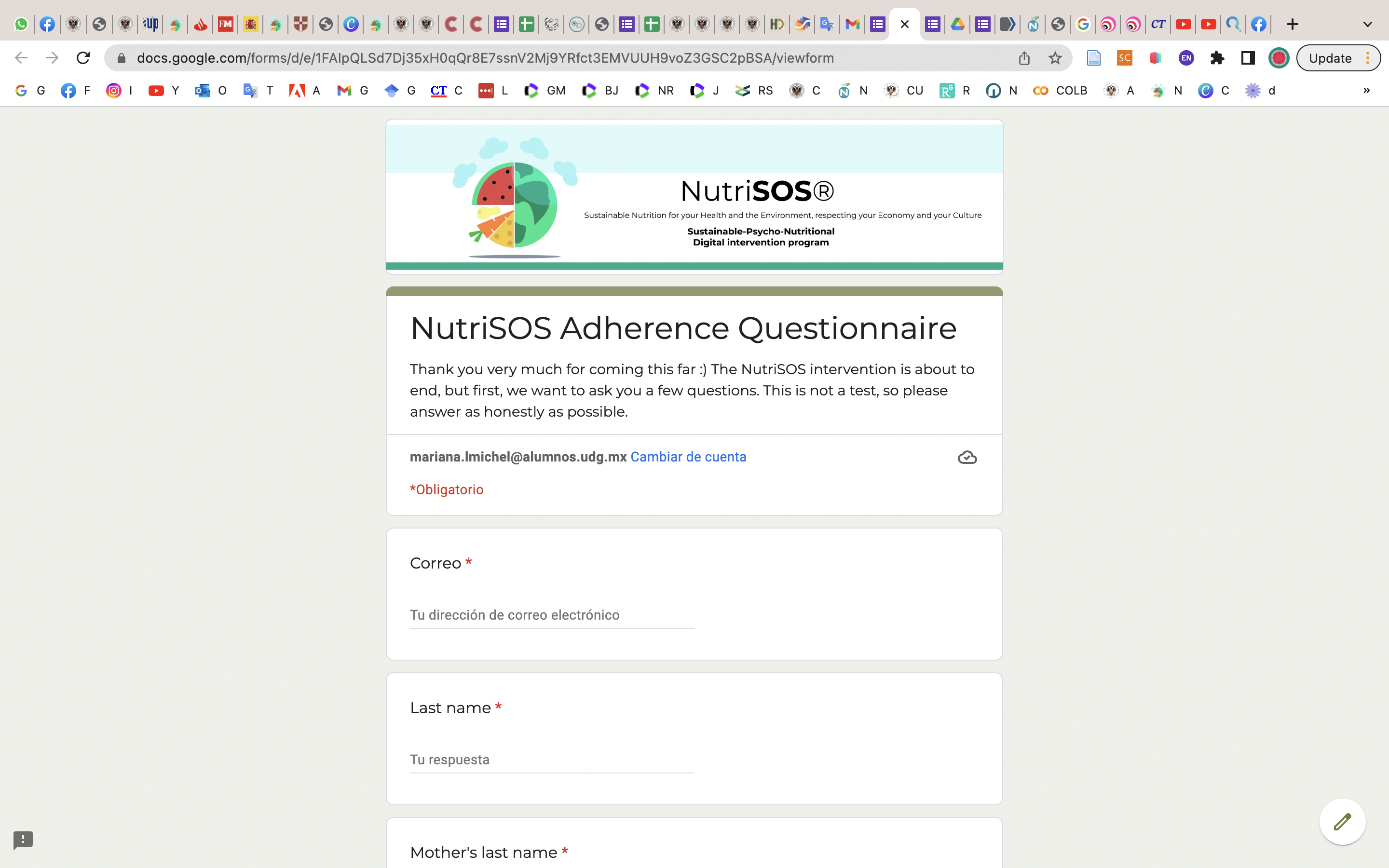


Figure SM1.10.1. Nutritional-sustainable knowledge questionnaire (English version)

Adherence questionnaire

Spanish version of the questionnaire is available at: <https://forms.gle/XdDZjqfHvKZKt7rG9>

For complete written version, see online supplementary material 11


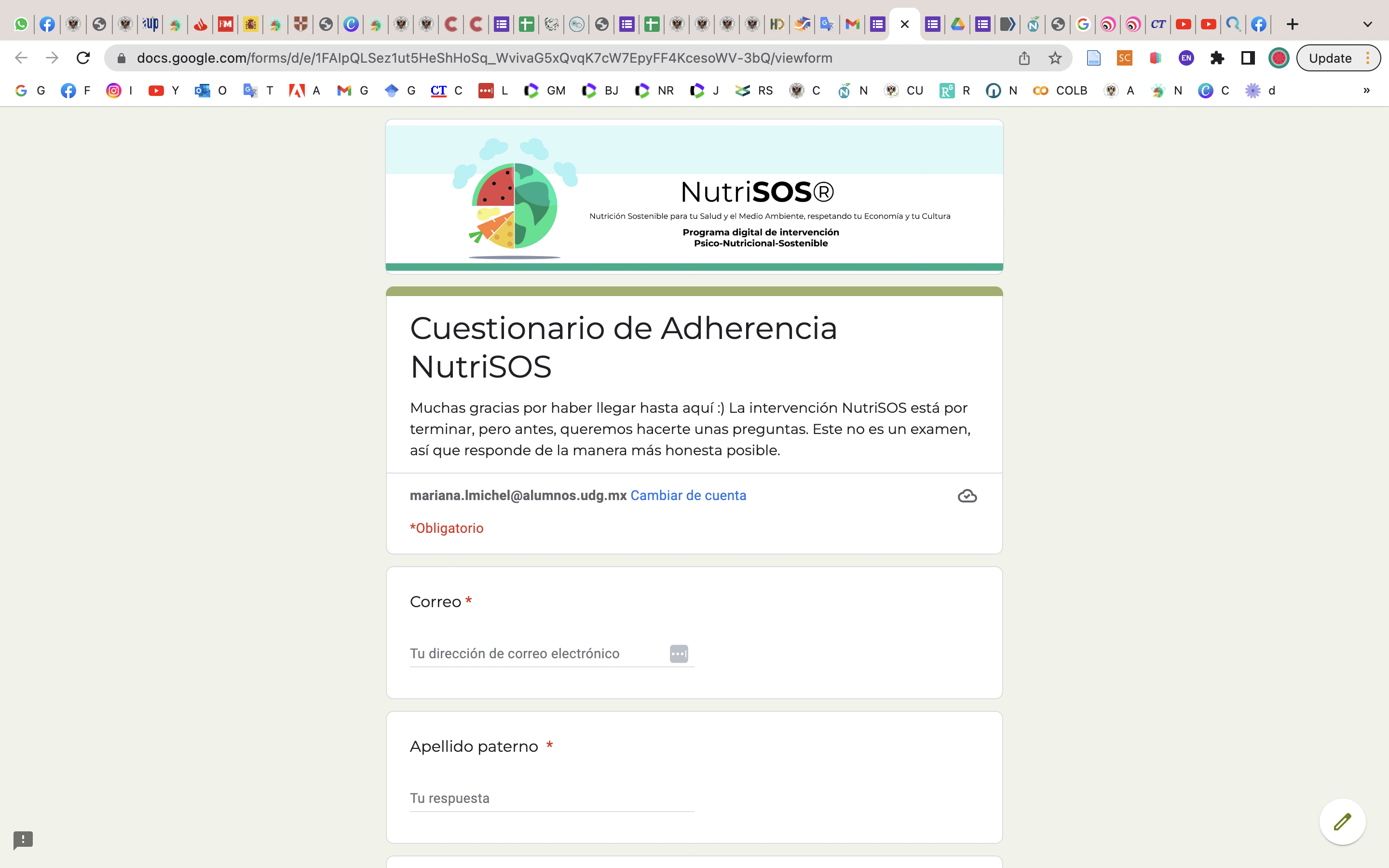


Figure SM1.10.2. Nutritional-sustainable knowledge questionnaire (Spanish version)

Online Supplementary Material 1.11. Statistical analysis according to outcomes, variable types and analytical objectives of the study

Table SM1.11 Statistical analysis according to outcomes, variable types and analytical objectives of the study

| Outcome | Variable | Analytical objective | Statistical test to use | |
| --- | --- | --- | --- | --- |
|  |  |  | Normal data | Non-normal data |
|  |  | Compare baseline, week 8 and week 15 in each group (control, experimental, and two arms experimental) | | |
| Primary | Numeric and continuous | Plasma glucose levels (mg/dl) | ANOVA with Bonferroni post-hoc | Friedman with Tukey post hoc |
| Primary | Numeric and continuous | Plasma triglycerides levels (mg/dl) |  |  |
| Primary | Numeric and continuous | Plasma Total Cholesterol Levels (mg/dl) |  |  |
| Primary | Numeric and continuous | Plasma LDL Cholesterol levels (mg/dl) |  |  |
| Primary | Numeric and continuous | Plasma HDL Cholesterol levels (mg/dl) |  |  |
| Primary | Numeric and continuous | Firmicutes (relative abundance) |  |  |
| Primary | Numeric and continuous | Bacteroidetes (relative abundance) |  |  |
| Primary | Numeric and continuous | Universal (relative abundance) |  |  |
| Primary | Numeric and continuous | *Lactobacillus* (relative abundance) |  |  |
| Primary | Numeric and continuous | *Bifidobacterium* (relative abundance) |  |  |
| Primary | Numeric and continuous | *Faecalibacterium prausnitzii* (relative abundance) |  |  |
| Primary | Numeric and continuous | *Akkermansia muciniphila* (relative abundance) |  |  |
| Primary | Numeric and continuous | *Prevotella copri* (relative abundance) |  |  |
| Primary | Numeric and continuous | *Bilophila wadsworthia* (relative abundance) |  |  |
| Primary | Numeric and continuous | *Clostridium coccoides* (relative abundance) |  |  |
| Primary | Numeric and continuous | *Streptococcus thermophilus* (relative abundance) |  |  |
| Primary | Numeric and continuous | Dietary water footprint (liters/person) |  |  |
| Primary | Numeric and continuous | Dietary carbon footprint (CO_2_eq/person) |  |  |
| Secondary | Numeric and continuous | Body weight (kg) |  |  |
| Primary | Numeric and continuous | Body Fat Percentage (%) |  |  |
| Secondary | Numeric and continuous | Muscle mass (%) |  |  |
| Secondary | Numeric and continuous | Visceral fat (%) |  |  |
| Primary | Numeric and continuous | Body Mass Index (kg/cm^2^) |  |  |
| Secondary | Numeric and continuous | Waist circumference (cm) |  |  |
| Secondary | Numeric and continuous | Hips circumference (cm) |  |  |
| Secondary | Numeric and continuous | Systolic blood pressure (mm Hg) |  |  |
| Secondary | Numeric and continuous | Diastolic blood pressure (mm Hg) |  |  |
| Secondary | Numeric and continuous | Energy (Kcal) |  |  |
| Secondary | Numeric and continuous | Fiber (g) |  |  |
| Secondary | Numeric and continuous | Carbohydrates (g) |  |  |
| Secondary | Numeric and continuous | Sugar (g) |  |  |
| Secondary | Numeric and continuous | Protein (g) |  |  |
| Secondary | Numeric and continuous | Lipids (g) |  |  |
| Secondary | Numeric and continuous | Saturated fatty acids (g) |  |  |
| Secondary | Numeric and continuous | Monounsaturated fatty acids (g) |  |  |
| Secondary | Numeric and continuous | Polyunsaturated fatty acids (g) |  |  |
| Secondary | Numeric and continuous | Cholesterol (mg) |  |  |
| Secondary | Numeric and continuous | Calcium (mg) |  |  |
| Secondary | Numeric and continuous | Phosphorus (mg) |  |  |
| Secondary | Numeric and continuous | Iron (mg) |  |  |
| Secondary | Numeric and continuous | Magnesium (mg) |  |  |
| Secondary | Numeric and continuous | Sodium (mg) |  |  |
| Secondary | Numeric and continuous | Potassium (mg) |  |  |
| Secondary | Numeric and continuous | Zinc (mg) |  |  |
| Secondary | Numeric and continuous | Selenium (mg) |  |  |
| Secondary | Numeric and continuous | Vitamin A (µg RE) |  |  |
| Secondary | Numeric and continuous | Ascorbic acid (mg) |  |  |
| Secondary | Numeric and continuous | Thiamine (mg) |  |  |
| Secondary | Numeric and continuous | Riboflavin (mg) |  |  |
| Secondary | Numeric and continuous | Niacin (mg) |  |  |
| Secondary | Numeric and continuous | Pyridoxine (mg) |  |  |
| Secondary | Numeric and continuous | Folic acid (µg) |  |  |
| Secondary | Numeric and continuous | Cobalamin (mg) |  |  |
| Secondary | Numeric and continuous | Ethanol (g) |  |  |
| Primary | Numeric and continuous | Mexican foods and dishes intake (g) |  |  |
| Primary | Numeric and continuous | Fruits and vegetables (g) |  |  |
| Primary | Numeric and continuous | Whole grains (g) |  |  |
| Primary | Numeric and continuous | Legumes (g) |  |  |
| Primary | Numeric and continuous | Dairy products (g) |  |  |
| Primary | Numeric and continuous | Seeds and healthy fats (g) |  |  |
| Primary | Numeric and continuous | Eggs (g) |  |  |
| Primary | Numeric and continuous | Fish and seafood (g) |  |  |
| Primary | Numeric and continuous | Chicken (g) |  |  |
| Primary | Numeric and continuous | Beef, pork, goat, lamb and processed meats (g) |  |  |
| Primary | Numeric and continuous | Ultra-processed foods (g) |  |  |
| Primary | Numeric and continuous | Added and free sugars, and trans and saturated fats (g) |  |  |
| Primary | Numeric and continuous | Diet quality (total score) |  |  |

Table SM1.11 Continuation. Statistical analysis according to outcomes, variable types and analytical objectives of the study

| Outcome | Variable | Analytical objective | Statistical test to use | |
| --- | --- | --- | --- | --- |
|  |  |  | Normal data | Non-normal data |
|  |  | Compare baseline, week 8 and week 15 in each group (control, experimental, and two arms experimental) | | |
| Secondary | Categoric nominal | Signs of nutrients deficiencies or excess (presence or absence) |  | |
| Secondary | Categoric nominal | Acanthosis nigricans (presence or absence) | Chi square | |
| Secondary | Categoric ordinal | Nutritional-sustainable knowledge (level) |  |  |
| Descriptive | Categoric ordinal | Adherence to the program (level) |  |  |
| Primary | Categoric ordinal | Physical activity (level) |  | |
|  |  | Compare between control, experimental and two arms experimental groups at week 0, 8 and 15 | | |
| Primary | Numeric and continuous | Plasma glucose levels (mg/dl) | Student's t test for independent samples | Mann-Whitney U Test |
| Primary | Numeric and continuous | Plasma triglycerides levels (mg/dl) |  |  |
| Primary | Numeric and continuous | Plasma Total Cholesterol Levels (mg/dl) |  |  |
| Primary | Numeric and continuous | Plasma LDL Cholesterol levels (mg/dl) |  |  |
| Primary | Numeric and continuous | Plasma HDL Cholesterol levels (mg/dl) |  |  |
| Primary | Numeric and continuous | Firmicutes (relative abundance) |  |  |
| Primary | Numeric and continuous | Bacteroidetes (relative abundance) |  |  |
| Primary | Numeric and continuous | Universal (relative abundance) |  |  |
| Primary | Numeric and continuous | *Lactobacillus* (relative abundance) |  |  |
| Primary | Numeric and continuous | *Bifidobacterium* (relative abundance) |  |  |
| Primary | Numeric and continuous | *Faecalibacterium prausnitzii* (relative abundance) |  |  |
| Primary | Numeric and continuous | *Akkermansia muciniphila* (relative abundance) |  |  |
| Primary | Numeric and continuous | *Prevotella copri* (relative abundance) |  |  |
| Primary | Numeric and continuous | *Bilophila wadsworthia* (relative abundance) |  |  |
| Primary | Numeric and continuous | *Clostridium coccoides* (relative abundance) |  |  |
| Primary | Numeric and continuous | *Streptococcus thermophilus* (relative abundance) |  |  |
| Primary | Numeric and continuous | Dietary water footprint (liters/person) |  |  |
| Primary | Numeric and continuous | Dietary carbon footprint (CO_2_eq/person) |  |  |
| Secondary | Numeric and continuous | Body weight (kg) |  |  |
| Primary | Numeric and continuous | Body Fat Percentage (%) |  |  |
| Secondary | Numeric and continuous | Muscle mass (%) |  |  |
| Secondary | Numeric and continuous | Visceral fat (%) |  |  |
| Primary | Numeric and continuous | Body Mass Index (kg/cm^2^) |  |  |
| Secondary | Numeric and continuous | Waist circumference (cm) |  |  |
| Secondary | Numeric and continuous | Hips circumference (cm) |  |  |
| Secondary | Numeric and continuous | Systolic blood pressure (mm Hg) |  |  |
| Secondary | Numeric and continuous | Diastolic blood pressure (mm Hg) |  |  |
| Secondary | Numeric and continuous | Energy (Kcal) |  |  |
| Secondary | Numeric and continuous | Fiber (g) |  |  |
| Secondary | Numeric and continuous | Carbohydrates (g) |  |  |
| Secondary | Numeric and continuous | Sugar (g) |  |  |
| Secondary | Numeric and continuous | Protein (g) |  |  |
| Secondary | Numeric and continuous | Lipids (g) |  |  |
| Secondary | Numeric and continuous | Saturated fatty acids (g) |  |  |
| Secondary | Numeric and continuous | Monounsaturated fatty acids (g) |  |  |
| Secondary | Numeric and continuous | Polyunsaturated fatty acids (g) |  |  |
| Secondary | Numeric and continuous | Cholesterol (mg) |  |  |
| Secondary | Numeric and continuous | Calcium (mg) |  |  |
| Secondary | Numeric and continuous | Phosphorus (mg) |  |  |
| Secondary | Numeric and continuous | Iron (mg) |  |  |
| Secondary | Numeric and continuous | Magnesium (mg) |  |  |
| Secondary | Numeric and continuous | Sodium (mg) |  |  |
| Secondary | Numeric and continuous | Potassium (mg) |  |  |
| Secondary | Numeric and continuous | Zinc (mg) |  |  |
| Secondary | Numeric and continuous | Selenium (mg) |  |  |
| Secondary | Numeric and continuous | Vitamin A (µg RE) |  |  |
| Secondary | Numeric and continuous | Ascorbic acid (mg) |  |  |
| Secondary | Numeric and continuous | Thiamine (mg) |  |  |
| Secondary | Numeric and continuous | Riboflavin (mg) |  |  |
| Secondary | Numeric and continuous | Niacin (mg) |  |  |
| Secondary | Numeric and continuous | Pyridoxine (mg) |  |  |
| Secondary | Numeric and continuous | Folic acid (µg) |  |  |
| Secondary | Numeric and continuous | Cobalamin (mg) |  |  |
| Secondary | Numeric and continuous | Ethanol (g) |  |  |
| Primary | Numeric and continuous | Mexican foods and dishes intake (g) |  |  |
| Primary | Numeric and continuous | Fruits and vegetables (g) |  |  |
| Primary | Numeric and continuous | Whole grains (g) |  |  |
| Primary | Numeric and continuous | Legumes (g) |  |  |
| Primary | Numeric and continuous | Dairy products (g) |  |  |
| Primary | Numeric and continuous | Seeds and healthy fats (g) |  |  |
| Primary | Numeric and continuous | Eggs (g) |  |  |
| Primary | Numeric and continuous | Fish and seafood (g) |  |  |
| Primary | Numeric and continuous | Chicken (g) |  |  |
| Primary | Numeric and continuous | Beef, pork, goat, lamb and processed meats (g) |  |  |
| Primary | Numeric and continuous | Ultra-processed foods (g) |  |  |
| Primary | Numeric and continuous | Added and free sugars, and trans and saturated fats (g) |  |  |
| Primary | Numeric and continuous | Diet quality (total score) |  |  |
| Secondary | Categoric nominal | Signs of nutrients deficiencies or excess (presence or absence) |  | |
| Secondary | Categoric nominal | Acanthosis nigricans (presence or absence) | Chi square | |
| Secondary | Categoric ordinal | Nutritional-sustainable knowledge (level) |  |  |
| Descriptive | Categoric ordinal | Adherence to the program (level) |  |  |
| Primary | Categoric ordinal | Physical activity (level) |  | |

Table SM1.11 Continuation. Statistical analysis according to outcomes, variable types and analytical objectives of the study

| Outcome | Variable | Analytical objective | Statistical test to use | |
| --- | --- | --- | --- | --- |
|  |  |  | Normal data | Non-normal data |
|  |  | To explore the relationships between the participant’s adherence to the intervention and primary and secondary outcomes | | |
| Primary | Independent categorical | Adherence to the program | Mixed effects linear regression models | Mixed effects linear regression models (with logarithmic transformation of data) |
|  | **Dependent continuous** |  |  |  |
| Primary | Numeric and continuous | Plasma glucose levels (mg/dl) |  |  |
| Primary | Numeric and continuous | Plasma triglycerides levels (mg/dl) |  |  |
| Primary | Numeric and continuous | Plasma Total Cholesterol Levels (mg/dl) |  |  |
| Primary | Numeric and continuous | Plasma LDL Cholesterol levels (mg/dl) |  |  |
| Primary | Numeric and continuous | Plasma HDL Cholesterol levels (mg/dl) |  |  |
| Primary | Numeric and continuous | Firmicutes (relative abundance) |  |  |
| Primary | Numeric and continuous | Bacteroidetes (relative abundance) |  |  |
| Primary | Numeric and continuous | Universal (relative abundance) |  |  |
| Primary | Numeric and continuous | *Lactobacillus* (relative abundance) |  |  |
| Primary | Numeric and continuous | *Bifidobacterium* (relative abundance) |  |  |
| Primary | Numeric and continuous | *Faecalibacterium prausnitzii* (relative abundance) |  |  |
| Primary | Numeric and continuous | *Akkermansia muciniphila* (relative abundance) |  |  |
| Primary | Numeric and continuous | *Prevotella copri* (relative abundance) |  |  |
| Primary | Numeric and continuous | *Bilophila wadsworthia* (relative abundance) |  |  |
| Primary | Numeric and continuous | *Clostridium coccoides* (relative abundance) |  |  |
| Primary | Numeric and continuous | *Streptococcus thermophilus* (relative abundance) |  |  |
| Primary | Numeric and continuous | Dietary water footprint (liters/person) |  |  |
| Primary | Numeric and continuous | Dietary carbon footprint (CO_2_eq/person) |  |  |
| Secondary | Numeric and continuous | Body weight (kg) |  |  |
| Primary | Numeric and continuous | Body Fat Percentage (%) |  |  |
| Secondary | Numeric and continuous | Muscle mass (%) |  |  |
| Secondary | Numeric and continuous | Visceral fat (%) |  |  |
| Primary | Numeric and continuous | Body Mass Index (kg/cm^2^) |  |  |
| Secondary | Numeric and continuous | Waist circumference (cm) |  |  |
| Secondary | Numeric and continuous | Hips circumference (cm) |  |  |
| Secondary | Numeric and continuous | Systolic blood pressure (mm Hg) |  |  |
| Secondary | Numeric and continuous | Diastolic blood pressure (mm Hg) |  |  |
| Secondary | Numeric and continuous | Energy (Kcal) |  |  |
| Secondary | Numeric and continuous | Fiber (g) |  |  |
| Secondary | Numeric and continuous | Carbohydrates (g) |  |  |
| Secondary | Numeric and continuous | Sugar (g) |  |  |
| Secondary | Numeric and continuous | Protein (g) |  |  |
| Secondary | Numeric and continuous | Lipids (g) |  |  |
| Secondary | Numeric and continuous | Saturated fatty acids (g) |  |  |
| Secondary | Numeric and continuous | Monounsaturated fatty acids (g) |  |  |
| Secondary | Numeric and continuous | Polyunsaturated fatty acids (g) |  |  |
| Secondary | Numeric and continuous | Cholesterol (mg) |  |  |
| Secondary | Numeric and continuous | Calcium (mg) |  |  |
| Secondary | Numeric and continuous | Phosphorus (mg) |  |  |
| Secondary | Numeric and continuous | Iron (mg) |  |  |
| Secondary | Numeric and continuous | Magnesium (mg) |  |  |
| Secondary | Numeric and continuous | Sodium (mg) |  |  |
| Secondary | Numeric and continuous | Potassium (mg) |  |  |
| Secondary | Numeric and continuous | Zinc (mg) |  |  |
| Secondary | Numeric and continuous | Selenium (mg) |  |  |
| Secondary | Numeric and continuous | Vitamin A (µg RE) |  |  |
| Secondary | Numeric and continuous | Ascorbic acid (mg) |  |  |
| Secondary | Numeric and continuous | Thiamine (mg) |  |  |
| Secondary | Numeric and continuous | Riboflavin (mg) |  |  |
| Secondary | Numeric and continuous | Niacin (mg) |  |  |
| Secondary | Numeric and continuous | Pyridoxine (mg) |  |  |
| Secondary | Numeric and continuous | Folic acid (µg) |  |  |
| Secondary | Numeric and continuous | Cobalamin (mg) |  |  |
| Secondary | Numeric and continuous | Ethanol (g) |  |  |
| Primary | Numeric and continuous | Mexican foods and dishes intake (g) |  |  |
| Primary | Numeric and continuous | Fruits and vegetables (g) |  |  |
| Primary | Numeric and continuous | Whole grains (g) |  |  |
| Primary | Numeric and continuous | Legumes (g) |  |  |
| Primary | Numeric and continuous | Dairy products (g) |  |  |
| Primary | Numeric and continuous | Seeds and healthy fats (g) |  |  |
| Primary | Numeric and continuous | Eggs (g) |  |  |
| Primary | Numeric and continuous | Fish and seafood (g) |  |  |
| Primary | Numeric and continuous | Chicken (g) |  |  |
| Primary | Numeric and continuous | Beef, pork, goat, lamb and processed meats (g) |  |  |
| Primary | Numeric and continuous | Ultra-processed foods (g) |  |  |
| Primary | Numeric and continuous | Added and free sugars, and trans and saturated fats (g) |  |  |
| Primary | Numeric and continuous | Diet quality (total score) |  |  |
| Secondary | Numeric and continuous | Signs of nutrients deficiencies or excess (total score) |  |  |
| Secondary | Numeric and continuous | Acanthosis nigricans (total score) |  |  |
| Secondary | Numeric and continuous | Nutritional-sustainable knowledge (total score) |  |  |
| Primary | Numeric and continuous | Physical activity (total score) |  |  |
| Primary | Numeric and continuous | COM-B behavioral aspects (total score) |  |  |

Table SM1.11 Continuation. Statistical analysis according to outcomes, variable types and analytical objectives of the study

| Outcome | Variables | Analytical objective | Statistical test to use | |
| --- | --- | --- | --- | --- |
|  |  |  | Normal data | Non-normal data |
|  |  | To identify risks and protective factors between the participant’s adherence to the intervention and primary and secondary outcomes | | |
| Primary | Independent dichotomic | Adherence to the program (adequate or inadequate adherence) | Binary Logistic Regression Reporting Odds Ratios | Binary logistic regression reporting Odds Ratios (with logarithmic transformation of data) |
|  |  |  |  |  |
| Primary | Dependent dichotomic | Plasma glucose levels (under or over 100 mg/dl) |  |  |
| Primary | Dependent dichotomic | Plasma triglycerides levels (under or over 150 mg/dl) |  |  |
| Primary | Dependent dichotomic | Plasma Total Cholesterol Levels (under or over 200 mg/dl) |  |  |
| Primary | Dependent dichotomic | Plasma LDL Cholesterol levels (under or over 160 mg/dl) |  |  |
| Primary | Dependent dichotomic | Plasma HDL Cholesterol levels (under or over 40 mg/dl) |  |  |
| Primary | Dependent dichotomic | Firmicutes (under or over median values of Relative Abundance [RA] of each group) |  |  |
| Primary | Dependent dichotomic | Bacteroidetes (under or over median values of RA of each group) |  |  |
| Primary | Dependent dichotomic | Universal (under or over median values of RA of each group) |  |  |
| Primary | Dependent dichotomic | *Lactobacillus* (under or over median values of RA of each group) |  |  |
| Primary | Dependent dichotomic | *Bifidobacterium* (under or over median values of RA of each group) |  |  |
| Primary | Dependent dichotomic | *Faecalibacterium prausnitzii* (under or over median values of RA of each group) |  |  |
| Primary | Dependent dichotomic | *Akkermansia muciniphila* (under or over median values of RA of each group) |  |  |
| Primary | Dependent dichotomic | *Prevotella copri* (under or over median values of RA of each group) |  |  |
| Primary | Dependent dichotomic | *Bilophila wadsworthia* (under or over median values of RA of each group) |  |  |
| Primary | Dependent dichotomic | *Clostridium coccoides* (under or over median values of RA of each group) |  |  |
| Primary | Dependent dichotomic | *Streptococcus thermophilus* (under or over median values of RA of each group) |  |  |
| Primary | Dependent dichotomic | Dietary water footprint (2,714 Liters per person per day (L p^−1^d^−1^) |  |  |
| Primary | Dependent dichotomic | Dietary carbon footprint (2.43 kg CO_2_eq/day) |  |  |
| Secondary | Dependent dichotomic | Body weight (under or over theoretical body weight) |  |  |
| Primary | Dependent dichotomic | Body Fat Percentage (under or over 22% in men and 32% in women) |  |  |
| Secondary | Dependent dichotomic | Muscle mass (high and average as controls and low as cases) |  |  |
| Secondary | Dependent dichotomic | Visceral fat (under or over 12) |  |  |
| Primary | Dependent dichotomic | Body Mass Index (under or over 25 kg/cm^2^) |  |  |
| Secondary | Dependent dichotomic | Waist circumference (under or over 90 cm in men and 80 cm in women) |  |  |
| Secondary | Dependent dichotomic | Waist-Hips ratio (under or over 0.90 cm in men and 0.85 cm in women) |  |  |
| Secondary | Dependent dichotomic | Systolic blood pressure (under or over 120 mm Hg) |  |  |
| Secondary | Dependent dichotomic | Diastolic blood pressure (under or over 80 mm Hg) |  |  |
| Secondary | Dependent dichotomic | Energy (under or over energy requirements in Kcal/day) |  |  |
| Secondary | Dependent dichotomic | Fiber (under or over 35 g in men and 30 in women per day) |  |  |
| Secondary | Dependent dichotomic | Carbohydrates (under or over 130 g per day) |  |  |
| Secondary | Dependent dichotomic | Sugar (under or over 10% from energy intake per day) |  |  |
| Secondary | Dependent dichotomic | Protein (under or over 0.8 g/kg of body weight per day) |  |  |
| Secondary | Dependent dichotomic | Lipids (under or over 30% from energy intake per day) |  |  |
| Secondary | Dependent dichotomic | Saturated fatty acids (under or over 7% from energy intake per day) |  |  |
| Secondary | Dependent dichotomic | Monounsaturated fatty acids (under or over 15% from energy intake per day) |  |  |
| Secondary | Dependent dichotomic | Polyunsaturated fatty acids (under or over 8% from energy intake per day) |  |  |
| Secondary | Dependent dichotomic | Cholesterol (under or over 300 mg per day) |  |  |
| Secondary | Dependent dichotomic | Calcium (under or over 900 mg per day) |  |  |
| Secondary | Dependent dichotomic | Phosphorus (under or over 560 mg per day) |  |  |
| Secondary | Dependent dichotomic | Iron (under or over 21 mg for women and 15 mg for men, per day) |  |  |
| Secondary | Dependent dichotomic | Magnesium (under or over 250 mg for women and 320 mg for men, per day) |  |  |
| Secondary | Dependent dichotomic | Sodium (under or over 1600 mg per day) |  |  |
| Secondary | Dependent dichotomic | Potassium (under or over 4700 mg per day) |  |  |
| Secondary | Dependent dichotomic | Zinc (under or over 11 mg for women and 15 mg for men, per day) |  |  |
| Secondary | Dependent dichotomic | Selenium (under or over 48 mg per day) |  |  |
| Secondary | Dependent dichotomic | Vitamin A (under or over 570 mg for women and 730 mg for men, per day) |  |  |
| Secondary | Dependent dichotomic | Ascorbic acid (under or over 75 mg for women and 84 mg for men, per day) |  |  |
| Secondary | Dependent dichotomic | Thiamine (under or over 0.9 mg for women and 1 mg for men, per day) |  |  |
| Secondary | Dependent dichotomic | Riboflavin (under or over 0.9 mg for women and 1.1 mg for men, per day) |  |  |
| Secondary | Dependent dichotomic | Niacin (under or over 12 mg for women and 13 mg for men, per day) |  |  |
| Secondary | Dependent dichotomic | Pyridoxine (under or over 1.3 mg per day) |  |  |
| Secondary | Dependent dichotomic | Folic acid (under or over 460 mg per day) |  |  |
| Secondary | Dependent dichotomic | Cobalamin (under or over 2.4 mg per day) |  |  |
| Secondary | Dependent dichotomic | Ethanol (under or over 14.4 mg per day) |  |  |
| Primary | Dependent dichotomic | Mexican foods and dishes intake (under or over 100 g per day) |  |  |
| Primary | Dependent dichotomic | Fruits and vegetables (under or over 200 g of fruits and 300 g of vegetables, per day) |  |  |
| Primary | Dependent dichotomic | Whole grains (under or over 200 g per day) |  |  |
| Primary | Dependent dichotomic | Legumes (under or over 60 g per day) |  |  |
| Primary | Dependent dichotomic | Dairy products (under or over 250 g per day) |  |  |
| Primary | Dependent dichotomic | Seeds and healthy fats (under or over 80 g per day) |  |  |
| Primary | Dependent dichotomic | Eggs (under or over 25 g per day) |  |  |
| Primary | Dependent dichotomic | Fish and seafood (under or over 28 g per day) |  |  |
| Primary | Dependent dichotomic | Chicken (under or over 29 g per day) |  |  |
| Primary | Dependent dichotomic | Beef, pork, goat, lamb and processed meats (under or over 7 g per day) |  |  |
| Primary | Dependent dichotomic | Ultra-processed foods (under or over 10 g per day) |  |  |
| Primary | Dependent dichotomic | Added and free sugars, and trans and saturated fats (under or over 31 g per day) |  |  |
| Primary | Dependent dichotomic | Diet quality (under or over median value) |  |  |
| Secondary | Dependent dichotomic | Signs of nutrients deficiencies or excess (presence or absence) |  |  |
| Secondary | Dependent dichotomic | Acanthosis nigricans (presence or absence) |  |  |
| Secondary | Dependent dichotomic | Nutritional-sustainable knowledge (under or over median value) |  |  |
| Primary | Dependent dichotomic | Physical activity (high and medium as controls and low as cases) |  |  |

Online Supplementary Material 1.12

**Informed consent for study participants (English version)**


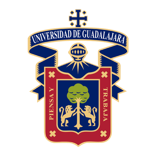


Official title:

Sustainable-psycho-nutritional intervention program for a sustainable diet (the ‘NutriSOS’ study) and its effects on eating behavior, diet quality, nutritional status, physical activity, metabolic biomarkers, gut microbiota, and water and carbon footprints in Mexican population: study protocol of an mHealth randomized controlled trial

**
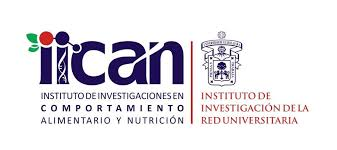
**Brief Title:

Sustainable-psycho-nutritional intervention program and its effects on health outcomes and the environment

#
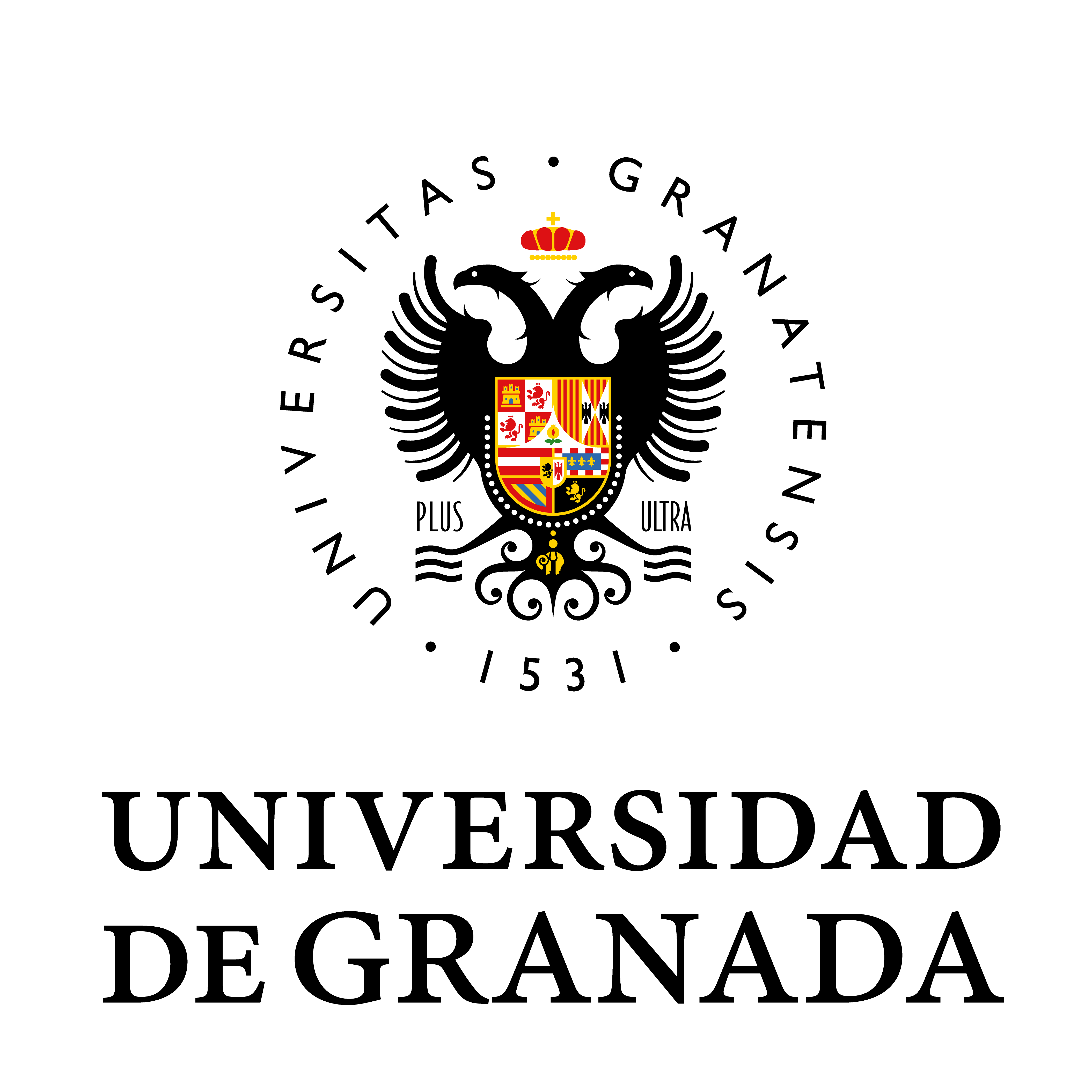
Responsible institution:

# Research Institute in Eating Behavior and Nutrition (Instituto de Investigaciones en Comportamiento Alimentario y Nutrición (IICAN), University Center of the South, University of Guadalajara. Address: Av. Enrique Arreola Silva 883, Col. Centro. Zip Code: 49000, Cd. Guzmán, Jalisco, México.

**
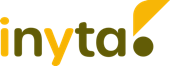
**Collaborating institutions:

Institute of Nutrition and Food Technology “José Mataix Verdú”, Biomedical Research Center, University of Granada, Avenida del Conocimiento S/N. Parque Tecnológico de la Salud. Armilla,18071 Granada, Spain


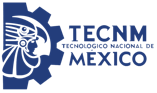


National Technological Institute of Mexico (Tecnológico Nacional de México, Campus Ciudad Guzmán) Address: Avenida Tecnológico 100, Col. Centro. Zip Code: 49000. Ciudad Guzmán, México.


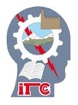


Zapotlán el Grande, Jalisco. Date:________________

By means of the present, I ______________________________________________________, I agree to participate in the research project entitled: **"** **Sustainable-psycho-nutritional intervention program for a sustainable diet (the ‘NutriSOS’ study) and its effects on eating behavior, diet quality, nutritional status, physical activity, metabolic biomarkers, gut microbiota, and water and carbon footprints in Mexican population: study protocol of an mHealth randomized controlled trial"**. I am aware that the objective of this study is to design a sustainable-psycho-nutritional intervention program and evaluate its effect on biological and environmental indicators in the Mexican population. and that the information collected will be analyzed for non-profit research purposes which will lead to publications in scientific journals. In addition, I am aware that at no time will I receive financial remuneration for my participation.

I have been informed that my participation will consist of an evaluation of the following aspects. Anthropometric data: weight, height, waist and hip circumference. To do this I must take off my shoes and uncover my waist for less than 3 minutes. Biochemical data: blood sample and stool. For this, a specialist will take blood from my arm, so I will have to discover it and maintain a 12-hour overnight fast. Likewise, I will be given a sterile container to place a 3 cm^3^ stool sample that I could be asked to preserve at the fridge if I a am not able to deliver it to the researcher as soon as I collected it. Clinical data: blood pressure measurement and superficial examination (e.g., face, neck, feet, hands), so I will uncover my left arm and allow the specialist to check my eyes, nails, hair, neck, and mouth, and, if necessary, the folds of my armpits. Dietary data: completion of dietary records and food frequency questionnaire. So, I will report the amounts of food I consume per day and on average per week, month, or year. I will be asked to write it down in paper, a mobile application, or a computer. Also, I may be asked to send photos of my food intake or uploaded into a mobile application. I am also aware that I will respond to a survey where I will provide sociodemographic and socioeconomic data, such as my educational level, my employment, a range of monthly income in Mexican pesos, among other data. In addition, I have been informed that I will have to use a mobile application that will help me improve my diet, both in relation to my nutrition and the environment. I know that the intervention in which I agree to participate will last 15 weeks, in which I will allow the extraction of blood and measurement of my weight, waist, and hip circumference on three occasions, as well as the visual physical examination and the taking of my blood pressure. I also know that I will deliver stool samples on a weekly basis, during the 15 weeks and that within the mobile application that I will use, I will be asked to record my food consumption on a daily basis, incorporating photographs and specifying the amounts of food consumed.

Additionally, I know that I may not be involved in the study but only evaluated. In addition, I know that the intervention, if it is part of it, will last 7 weeks, and additionally, there will be a 7-week follow-up period where I will be told whether or not I will be able to use the mobile application.

I know that the study will be carried out at the Centro Universitario del Sur, specifically at the Institute for Research in Eating Behavior and Nutrition (IICAN), as well as at the Laboratory of Biomedicine and Biotechnology for Health. In addition, it has been explained to me that my participation in this study is voluntary and I will not receive any financial compensation for it. However, I know that, if I wish, I will be able to obtain a psycho-nutritional-sustainable diagnosis, and furthermore, with my participation I will benefit from obtaining nutritional counseling at the end of the intervention.

Finally, I confirm that I have been informed that the responsible researchers will maintain the confidentiality of my personal data based on the guidelines established by the Federal Law on Protection of Personal Data Held by Private Parties.

Therefore, I declare that:

1. I have read or had read to me the information provided.

2. I have had the opportunity to ask and all my doubts and questions have been satisfactorily clarified.

3. I voluntarily consent to participate in this research.

4. I understand that I have the right to withdraw at any time I choose without it affecting me in any way.

5. I have been informed that my refusal to participate will not cause me any problems.

6. I have been informed that the researchers undertake to answer any questions I may have during and after the data collection process.

7. I am aware that the main investigator will provide me with a diagnosis of my nutritional status and will provide me with nutritional guidance, if I wish.

8. I am aware that measurements will be taken on my waist and hips, so I will have to uncover my abdomen. In addition, I know that I will have to remove my shoes, socks and metallic accessories, including the belt, earrings, among other artifacts, when the main researcher of the study asked me to do so, before getting on the scale to be weighed and before being measured on the stadiometer.

9. I consent to a blood sample being taken and am aware that I will submit a stool sample on a weekly basis.

10. I agree to allow my blood pressure to be taken, visual examination of my neck, eyes, mouth, hair, nails and, if applicable, armpit folds. I also agree to fill in the questionnaires that the main researcher provides me, always answering as honestly as possible.

11. I agree to use a mobile application where I know that I will provide information, which will be confidential, but will help me improve my diet, both in relation to my nutrition and the environment. Likewise, and if I wish, I will be able to interact with other users within the mobile application and thus provide us with support.

12. I accept that my data will be used in this and future studies, where my identity will always be protected.

If you have doubts or queries regarding participation in this study, you can contact the researcher responsible for this study, Mtra. Mariana Lares Michel, who is a doctoral student at the Institute for Research in Eating Behavior and Nutrition (IICAN). Her contact details are as follows: Av. Enrique Arreola Silva 883, Centro, C.P. 49000, Cd. Guzmán, Jalisco, Mexico, telephone: 3411017629. email: [mariana.lmichel@alumnos.udg.mx](mailto:mariana.lmichel@alumnos.udg.mx) Also, you can contact Dr. Fatima Ezzahra Housni, who is the responsible researcher for this study at ClinicalTrials.gov. Her contact details are as follows: Av. Enrique Arreola Silva 883, Centro, C.P. 49000, Cd. Guzmán, Jalisco, Mexico, Tel.: (+52) 341 5752222 ext. 46142. email: [fatima.housni@cusur.udg.mx](mailto:fatima.housni@cusur.udg.mx)

Sincerely

Place and date

_______________________________________________________

Name and signature of the participant

Telephone:__________________________________________________________________________

Email:_______________________________________________________________________________

Name and signature of the Witness 1: _______________________________________________________

Address and telephone: ___________________________________________________________________

Type of relationship with the participant: _____________________________________________________

Name and signature of the Witness 2: _______________________________________________________

Address and telephone: ___________________________________________________________________

Type of relationship with the participant: _____________________________________________________

I have explained to the participant the objective, justification and procedures of this research, as well as the risks and benefits that their participation implies. In turn, I have answered all the questions, doubts and clarifications that he has made me. Finally, I declare that I respect the guidelines and regulations corresponding to research on human beings.

_____________________________ _____________________________

Name and signature of the investigator Name and signature of the applicator

**Consentimiento informado para participantes (Spanish version)**


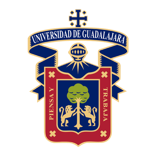


Título oficial

Programa de intervención psico-nutricional-sostenible para una dieta sustentable (estudio 'NutriSOS') y sus efectos sobre el comportamiento alimentario, calidad de la dieta, estado nutricional, actividad física, biomarcadores metabólicos, microbiota intestinal y huella hídrica y de carbono en población mexicana: estudio protocolo de un ensayo controlado aleatorizado de mHealth

**
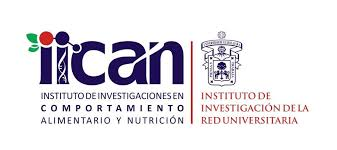
**Título breve:

Programa de intervención psico-nutricional-sostenible y sus efectos sobre la salud y el medio ambiente

#
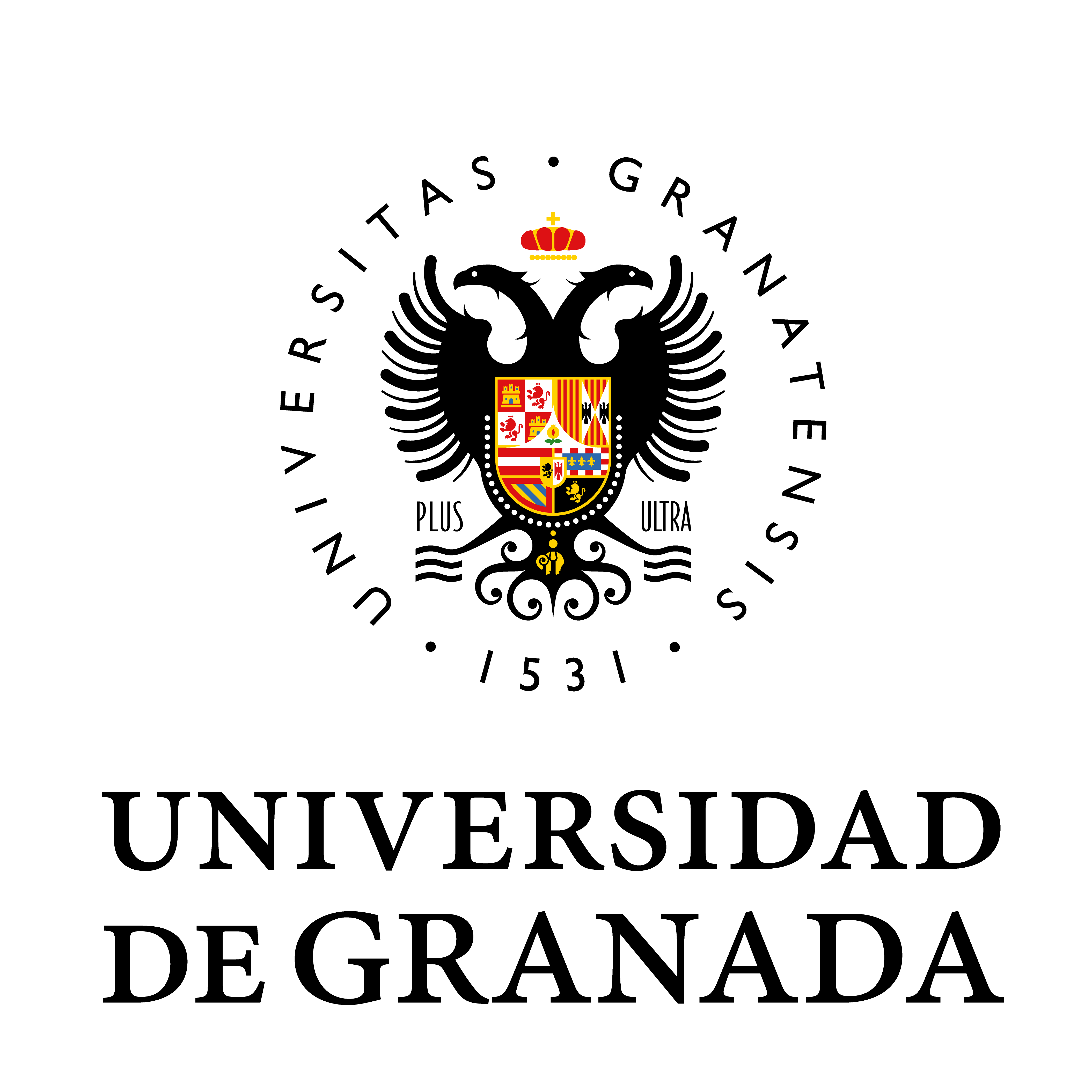
Instituciones responsables

# Instituto de Investigaciones en Comportamiento Alimentario y Nutrición (IICAN), Centro Universitario del Sur, Universidad de Guadalajara. Dirección: Av. Enrique Arreola Silva 883, Col. Centro. Código postal: 49000, Cd. Guzmán, Jalisco, México.

**
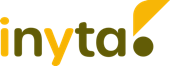
**Instituciones colaboradoras

Instituto de Nutrición y Tecnología de Alimentos “José Mataix Verdú”, Centro de Investigación Biomédica (CIBM), Universidad de Granada, Avenida del Conocimiento S/N. Parque Tecnológico de la Salud. Armilla,18071 Granada, España


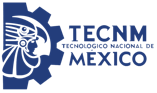


Tecnológico Nacional de México, Campus Ciudad Guzmán. Dirección Avenida Tecnológico 100, Col. Centro. Código postal: 49000. Ciudad Guzmán, México.


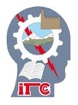


Zapotlán el Grande, Jalisco. Fecha:________________

Por medio de la presente, **yo** ______________________________________________________, acepto participar en el proyecto de investigación titulado: **“Programa de intervención psico-nutricional-sostenible para una dieta sustentable (estudio 'NutriSOS') y sus efectos sobre el comportamiento alimentario, calidad de la dieta, estado nutricional, actividad física, biomarcadores metabólicos, microbiota intestinal y huella hídrica y de carbono en población mexicana: estudio protocolo de un ensayo controlado aleatorizado de mHealth”**. Estoy enterado (a) de que el objetivo de este estudio es diseñar un programa de intervención psico-nutricional-sostenible y evaluar su efecto sobre indicadores biológicos y medioambientales en población mexicana. y que la información recabada será analizada con fines de investigación no lucrativos qué conllevarán a publicaciones en revistas científicas. Además, tengo presente que en ningún momento recibiré una remuneración económica por mi participación.

Se me ha informado que mi participación consistirá en una evaluación de los siguientes aspectos. Datos antropométricos: peso, estatura, circunferencia de cintura y cadera. Para ello deberé descalzarme y descubrir mi cintura por menos de 3 minutos. Datos bioquímicos: muestra de sangre y heces fecales. Para ello, un especialista extraerá sangre de mi brazo, por lo que deberé descubrirlo y mantener un ayuno nocturno de 12 horas. Así mimo, se me brindará un recipiente estéril para colocar una muestra de 3 cm^3^ de heces fecales. Datos clínicos: toma de presión arterial y exploración superficial (p.ej. rostro, cuello, pies, manos), por lo que descubriré mi brazo izquierdo y permitiré que el especialista revise mis ojos, uñas, cabello, cuello y boca, y, de ser necesario, los pliegues de mis axilas. Datos dietéticos: llenado de registros dietéticos y cuestionario de frecuencia de consumo de alimentos. Por lo que reportaré las cantidades de alimentos que consumo por día y en promedio por semana, mes o año. También tengo conocimiento de que responderé a una encuesta donde proporcionaré datos sociodemográficos y socioeconómicos, como mi nivel educativo, mi empleo, un rango de ingreso mensual en pesos mexicanos, entre otros datos. Además, se me ha informado que deberé utilizar una aplicación móvil que me ayudará a mejorar mi alimentación, tanto con relación a mi nutrición como al medio ambiente. Se que la intervención en la que acepto participar tendrá una duración de 15 semanas, en las cuales permitiré la extracción de sangre y medición de mi peso, circunferencia de cintura y cadera en tres ocasiones, así como la exploración física visual y la toma de mi presión arterial. También se que entregaré muestras de heces de manera semanal, durante las 15 semanas y que dentro de la aplicación móvil que utilizaré, se me solicitará que registre mi consumo de alimentos de manera diaria, incorporando fotografías y especificando las cantidades consumidas de alimentos.

Adicionalmente, se que puedo no ser intervenido en el estudio sino únicamente evaluado. Además, sé que la intervención, en caso de que forme parte de ella, tendrá una duración de 7 semanas, y adicionalmente, existirá un periodo de seguimiento de 7 semanas donde se me indicará si podré usar o no la aplicación móvil.

Conozco que el estudio se realizará en el Centro Universitario del Sur, específicamente en el Instituto de Investigaciones en Comportamiento Alimentario y Nutrición (IICAN), así como en el Laboratorio de Biomedicina y Biotecnología para la Salud. Además, se me ha explicado que mi participación en este estudio es voluntaria y no recibiré ningún tipo de remuneración económica por ella. Sin embargo, se que, si así lo deseo, podré obtener un diagnóstico psico-nutricional-sostenible, y además, con mi participación me beneficiaré al obtener asesoría nutricional al final de la intervención.

Finalmente, confirmo que se me ha informado que los investigadores responsables mantendrán la confidencialidad de mis datos personales en función de los lineamientos establecidos por la Ley Federal de Protección de Datos Personales en Posesión de los Particulares.

Por consiguiente, declaro qué:

1. He leído o me han leído la información proporcionada.
2. He tenido la oportunidad de preguntar y me han aclarado satisfactoriamente todas mis dudas y preguntas.
3. Consiento voluntariamente a participar en esta investigación.
4. Entiendo que tengo derecho a retirarme en cualquier momento que lo decida sin que me afecte de ninguna manera.
5. Se me ha informado que mi negación a participar no me ocasionará ningún problema.
6. Se me ha informado que los investigadores se comprometen a responder a cualquier duda que tenga durante y después del proceso de recolección de la información.
7. Estoy enterado (a) que el investigador principal me brindará un diagnostico de mi estado nutricional y me brindará orientación nutricional, si así lo deseo.
8. Estoy enterado (a) de que se realizarán mediciones en mi cintura y cadera, por lo que deberé descubrir mi abdomen. Además, se que deberé retirar mis zapatos, calcetines y accesorios metálicos, incluido el cinturón, aretes, entre otros artefactos, cuando la investigadora principal del estudio me solicité que lo haga, previo a subir a la báscula para que me pesen y previó a ser medido en el estadímetro.
9. Consiento que sea tomada una muestra de sangre y estoy enterado (a) que entregaré una muestra de heces fecales de manera semanal.
10. Estoy de acuerdo en permitir que se tome mi presión arterial, se explore visualmente mi cuello, ojos, boca, cabello, uñas y en dado caso, pliegues de axilas. Además, estoy de acuerdo en rellenar los cuestionarios que el investigador principal me proporciones, respondiendo siempre de la manera más honesta posible.
11. Acepto utilizar una aplicación móvil donde se que proporcionaré información, la cual será confidencial, pero me ayudará a mejorar mi alimentación, tanto con relación a mi nutrición como al medio ambiente. Así mismo, y si lo deseo, podré interactuar con otros usuarios dentro de la aplicación móvil y así brindarnos apoyo.
12. Acepto que mis datos sean utilizados en este y en futuros estudios, donde siempre será protegida mi identidad.

Si tuviera dudas o consultas respecto de la participación en este estudio puede contactar a la investigadora responsable de este estudio Mtra. Mariana Lares Michel, quien es estudiante de doctorado en el Instituto de Investigaciones en Comportamiento Alimentario y Nutrición (IICAN). Sus datos de contacto son los siguientes: Av. Enrique Arreola Silva 883, Centro, C.P. 49000, Cd. Guzmán, Jalisco, México, 3411017629 correo electrónico: [mariana.lmichel@alumnos.udg.mx](mailto:mariana.lmichel@alumnos.udg.mx)

Atentamente

Lugar y fecha

_______________________________________________________

Nombre y firma del participante

Teléfono: _______________________________________________________________________

Correo electrónico: _______________________________________________________________________

Nombre y firma del Testigo 1 Nombre y firma del Testigo 2

Dirección y teléfono: Dirección y teléfono:

Tipo de relación con el participante: Tipo de relación con el participante:

He explicado al participante el objetivo, justificación y procedimientos de la presente investigación, al igual que los riesgos y beneficios que implica su participación. A su vez, he contestado todas las preguntas, dudas y aclaraciones que me ha realizado. Finalmente, declaro que respeto los lineamentos y la normatividad correspondiente a la investigación en seres humanos.

_____________________________ _____________________________

Nombre y firma del investigador Nombre y firma del aplicador
