# Supplementary material for: Sustainable-psycho-nutritional intervention programme for a sustainable diet (the ‘NutriSOS’ study) and its effects on eating behaviour, diet quality, nutritional status, physical activity, metabolic biomarkers, gut microbiota and water and carbon footprints in Mexican population: study protocol of an mHealth randomised controlled trial
Source: Br J Nutr. 2023 Mar 30;130(10):1823–38. doi: 10.1017/S0007114523000843 (PMC10587386; doi:10.1017/S0007114523000843)
Supplement: Supplementary file 1 [file S0007114523000843sup.zip › S0007114523000843sup002.zip!/Online Supplementary Material 2 Feasibility, Acceptability, Quality and Usability Questionnaire English.pdf]

Questionnaire to evaluate the Feasibility, Acceptability, Quality and Usability of the NutriSOS® mobile application

Hello! Welcome to the NutriSOS® sustainable nutritional app evaluation questionnaire. We are pleased to tell you briefly about this technological development that the National Technological Institute of Mexico, Ciudad Guzmán campus, and the Institute for Research on Food Behavior and Nutrition (IICAN) of the University of Guadalajara have created. This is a mobile application whose objective is to help the Mexican population adopt a sustainable diet, improve their health and take care of the environment at the same time, respecting their culture and economic aspects at all times. However, to achieve this, we must first validate the application, so we appreciate your support in taking the time to answer this questionnaire, which aims to assess the feasibility, acceptability, quality and usability of NutriSOS®. However, remember that your participation is completely voluntary and if at any time you wish to withdraw from the study, you may do so without any negative repercussions for yourself.

*Obligatorio

1. Do you agree to participate in this study? *

Marca solo un óvalo.

- ( ) Yes
- ( ) No

Participant data

2. Name *

____

3. Last name *

4. Mother's last name *

5. Age *

6. Sex *

Marca solo un óvalo.

- Male
- Female
- Prefer not to say
- Otro: ____

7. Did you complete the initial registration within the NutriSOS® app? *

Marca solo un óvalo.

- Yes
- No

Viability

Below are some questions related to the feasibility of the NutriSOS® sustainable nutritional mobile application.

Please answer as honestly as possible, remember there are no right or wrong answers.

8. Section 1. Getting Started: Was it easy to sign up for the app? *

Marca solo un óvalo.

- Yes
- No

9. If your answer was no, describe why

____
____
____
____
____

10. Section 1. Getting started: Was it easy to log in once registered? *

Marca solo un óvalo.

- Yes
- No
- More or less

11. Section 2.1. General Data Questionnaire: Were the questions easy to answer? *

Marca solo un óvalo.

- Nothing easy
- A bit
- Normal
- Easy
- Very easy

12. Section 2.1. General Data Questionnaire: Were there any questions that were difficult to answer? *

Marca solo un óvalo.

- Yes
- No

13. If your answer was yes, mention which one and please describe why it was so

14. Section 2.1. General Data Questionnaire: Did you find it boring to answer the questions about general data? *

Marca solo un óvalo.

- Not boring
- Very little
- Moderately boring
- Very boring
- Extremely boring

15. Section 2.2. Quiz About You: Were the questions easy to answer? *

Marca solo un óvalo.

- Nothing easy
- A bit
- Normal
- Easy
- Very easy

16. Section 2.2. Questionnaire About You: Were there any questions that were difficult to answer? *

Marca solo un óvalo.

- Yes
- No

17. If your answer was yes, mention which one and please describe why it was so

18. Section 2.2. Questionnaire About You: Did you find it boring to answer the questions about yourself? *

Marca solo un óvalo.

- Not boring
- Very little
- Moderately boring
- Very boring
- Extremely boring

19. Section 2.3. Clinical History Questionnaire: Were the questions easy to answer? *

Marca solo un óvalo.

- Nothing easy
- A bit
- Normal
- Easy
- Very easy

20. Section 2.3. Medical History Questionnaire: Were there any questions that were difficult to answer? *

Marca solo un óvalo.

- Yes
- No

21. If your answer was yes, mention which one and please describe why it was so

____

____

____

____

____

22. Section 2.3: Medical History Questionnaire: Did you find answering the medical history questions boring? *

Marca solo un óvalo.

- Not boring
- A bit
- Moderately boring
- Very boring
- Extremely boring

23. Section 2.4: Questionnaire Socio-Economic Level: Were the questions easy to answer? *

Marca solo un óvalo.

- Yes
- No

24. Section 2.4: Questionnaire Socio-Economic Level: Were there any questions that were difficult to answer? *

Marca solo un óvalo.

- Yes
- No

25. If your answer was yes, mention which one and please describe why it was so

____
____
____
____
____

26. Section 2.4: Questionnaire Socio-Economic Level: Did you find answering the questions about socio-economic level boring? *

Marca solo un óvalo.

- Not boring
- A bit
- moderately boring
- Very boring
- Extremely boring

27. Section 2.5: Questionnaire Diet: Was it easy to answer the questions about diet? *

Marca solo un óvalo.

- Yes
- No

28. Section 2.5: Diet Questionnaire: Were there any questions that were difficult to answer? *

Marca solo un óvalo.

- Yes
- No

29. If your answer was yes, mention which one and please describe why it was so

____
____
____
____
____

30. Section 2.5: Food Questionnaire: Did you find it boring to answer the questions about food? *

Marca solo un óvalo.

- Not boring
- A bit
- Moderately boring
- Very boring
- Extremely boring

31. Section 2.6. 24 hour reminder: Was it easy to enter the food? *

Marca solo un óvalo.

- Very easy
- Simple
- Normal
- A little complicated
- Complicated

32. Section 2.6. 24-hour reminder: Was there any aspect that you found difficult to answer? *

Marca solo un óvalo.

- Yes
- No

33. If your answer was yes, mention which one and please describe why it was so

____

____

____

____

____

34. Section 2.6. 24-hour reminder: Did you find it boring to answer the 24-hour reminder, that is, enter the food you ate the day before the evaluation? *

Marca solo un óvalo.

- Not boring
- A bit
- Moderately boring
- Very boring
- Extremely boring

35. Section 3. Interactive Sustainable Nutritional Food Guide: Are the colors of the pyramid nice to look at? *

Marca solo un óvalo.

- They are not nice
- They are fine
- They are nice

36. Section 4. Meal plan: Is there anything you would change in the meal plan section? *

Marca solo un óvalo.

- Yes
- No

37. If your answer was yes, mention what and please describe the modification

____
____
____
____
____

38. Section 5 . Dietary records: Was it easy to enter the food? *

Marca solo un óvalo.

- Very easy
- Moderately easy
- Complicated

39. Section 5. Dietary records: Was there any aspect that you found difficult to answer? *

Marca solo un óvalo.

- Yes
- No

40. If your answer was yes, mention which one and please describe why it was so

41. Section 6. Recipes: Was it easy to enter the recipe section? *

Marca solo un óvalo.

- Yes
- More or less
- No

42. Section 6. Recipes: Is the content of the videos clear? *

Marca solo un óvalo.

- Yes
- More or less
- No

43. Section 6. Recipes: Would you change anything in the videos? *

Marca solo un óvalo.

- Yes
- No

44. If your answer was yes, mention what and please describe the modification

45. Section 6. Prescriptions: Would you change anything about the prescriptions? *

Marca solo un óvalo.

- Yes
- No

46. If your answer was yes, mention what and please describe the modification

47. Section 7. Learning: Is the content of the videos clear? *

Marca solo un óvalo.

- It's not clear at all
- Don't answer all my questions
- Is clear
- It's pretty clear

48. Section 7. Learning: Would you change anything in the videos? *

Marca solo un óvalo.

- Yes
- No

49. If your answer was yes, mention what and please describe the modification

____
____
____
____
____

50. Section 7. Learning: Do you find the information provided useful for learning about sustainable nutrition? *

Marca solo un óvalo.

- Little useful
- Useful
- Very useful

51. Section 8. Community: Do you find it nice to use the community? *

Marca solo un óvalo.

- Little nice
- Regular
- Nice
- Very nice

52. Section 8. Community: Would you be willing to upload photos of the food you eat? *

Marca solo un óvalo.

- Yes
- No

53. Section 8. Community: Would you like to see pictures of the diet of others who have similar sustainable nutrition goals to yours? *

Marca solo un óvalo.

- I would not like to see
- Very little
- Regular
- I would like
- I'd love to

54. Section 8. Community: Would you be willing to interact with other users within the community by liking, commenting, or uploading new photos? *

Marca solo un óvalo.

- Yes
- No

55. Section 9. Messaging: Is it easy for you to send and receive messages? *

Marca solo un óvalo.

- Yes
- No

56. Section 9. Messages: Is there anything you would change in the messages section? *

Marca solo un óvalo.

- Yes
- No

57. If your answer was yes, mention what and please describe the modification

____
____
____
____
____

58. Section 10. Profile: Is it easy for you to edit your profile picture? *

Marca solo un óvalo.

- Yes
- More or less
- No

59. Section 10. Profile: Do the evolution graphs seem easy to interpret? *

Marca solo un óvalo.

- Very simple
- Regular
- Complicated

60. Section 10. Profile: Is there anything you would change in the profile section? *

Marca solo un óvalo.

- Yes
- No
- Maybe

61. If your answer was yes, mention what and please describe the modification

62. Section 11. Reminders: Is it nice to receive reminders in the application? *

Marca solo un óvalo.

- Nice
- Little nice
- Moderately annoying
- Very annoying
- Extremely annoying

63. Section 11. Reminders: Do you find reminders helpful? *

Marca solo un óvalo.

- Little useful
- Regular
- Very useful

64. Section 11. Reminders: Is there anything about the reminders in the app that you would like to change? *

Marca solo un óvalo.

- Yes
- No

65. If your answer was yes, mention what and please describe the modification

Acceptability

Below are some questions related to the acceptability of the NutriSOS® sustainable nutritional mobile application.

Please answer as honestly as possible, remember there are no right or wrong answers.

66. Section 1. Design: Is the font size adequate? *

Marca solo un óvalo.

- Yes
- No
- More or less

67. Section 1. Design: Do the colors of the app look right to you? *

Marca solo un óvalo.

- Yes
- No
- More or less

68. Section 1. Design: Is there anything you would change about the app? *

Marca solo un óvalo.

- Yes
- No

69. If your answer was yes, mention what and please describe the modification

70. Section 2. Use of the application: Would you continue to use the application? *

Marca solo un óvalo.

- Not at all
- Don't know
- Definitely

71. Please describe the reason for your answer

72. Section 2. Using the app: Bored using the app? *

Marca solo un óvalo.

- Not at all
- A bit
- Regular
- Quite

73. Section 2. Using the app: Have you ever felt frustrated using the app? *

Marca solo un óvalo.

- Never
- Sometimes
- All the time

Usability

Below are some questions related to the usability of the NutriSOS® sustainable nutritional mobile application.

Please answer as honestly as possible, remember there are no right or wrong answers.

74. Would you use the app frequently? *

Marca solo un óvalo.

- Not at all
- Don't know
- Definitely

75. If your answer was I don't know, please describe why.

____
____
____
____
____

76. I find the app unnecessarily complicated *

Marca solo un óvalo.

Not complicated at all

1 ⬭

2 ⬭

3 ⬭

4 ⬭

5 ⬭

Quite complicated

77. I think the app is easy to use *

Marca solo un óvalo.

	Not easy at all
1	◯
2	◯
3	◯
4	◯
5	◯
	Quite easy

78. Need advice for the use of the application *

Marca solo un óvalo.

◯ Never
◯ Sometimes
◯ All the time

79. The functionalities of the application are properly integrated *

Marca solo un óvalo.

◯ No
◯ More or less
◯ Yes

80. The app had many inconsistencies *

Marca solo un óvalo.

	Not at all inconsistent
1	⬭
2	⬭
3	⬭
4	⬭
5	⬭
	Pretty inconsistent

81. I think most people could learn to use the app quickly *

Marca solo un óvalo.

	Not at all
1	◯
2	◯
3	◯
4	◯
5	◯
	Definitely

82. I feel comfortable using the application *

Marca solo un óvalo.

	Nothing comfortable
1	⬭
2	⬭
3	⬭
4	⬭
5	⬭
	Definitely comfortable

83. I need to learn a lot of things before I can use the app *

Marca solo un óvalo.

I don't need to learn anything

1 ⬭

2 ⬭

3 ⬭

4 ⬭

5 ⬭

I need to learn a lot

Quality

Below are some questions related to the quality of the NutriSOS® sustainable nutritional mobile application.

Please answer as honestly as possible, remember there are no right or wrong answers.

84. Section 1. Interaction: Is the app fun/entertaining to use? *

Marca solo un óvalo.

	Nothing entertaining
1	◯
2	◯
3	◯
4	◯
5	◯
	Very entertaining

85. Section 1. Interaction: Does the application present interesting information compared to other similar ones? *

Marca solo un óvalo.

	Nothing interesting
1	◯
2	◯
3	◯
4	◯
5	◯
	Very interesting

86. Section 1. Interaction: Do you allow customization in settings and preferences? *

Marca solo un óvalo.

Does not allow it	
1	◯
2	◯
3	◯
4	◯
5	◯
Allows you to customize any feature	

87. Section 1. Interaction: Allow input of information, generate recommendations and provide feedback? *

Marca solo un óvalo.

Low level of feedback and interaction

1 ◯

2 ◯

3 ◯

4 ◯

5 ◯

High level of feedback and interaction

88. Section 1. Interaction: Is the content of the application appropriate for the target audience? *

Marca solo un óvalo.

	Completely inappropriate
1	◯
2	◯
3	◯
4	◯
5	◯
	Designed for the target audience

89. Section 2. Functionality: How smoothly do the components and the application in general work? *

Marca solo un óvalo.

The app is broken
1 ◯
2 ◯
3 ◯
4 ◯
5 ◯
Works perfect, no problems found

90. Section 2. Functionality: Navigation between the different sections of the application makes sense *

Marca solo un óvalo.

No connection, difficult navigation	
1	⬭
2	⬭
3	⬭
4	⬭
5	⬭
Intuitive and easy to navigate	

91. Section 2. Functionality: The interactions: clicks, scrolls, swipes of the application make sense *

Marca solo un óvalo.

There is no consistency

1 ◯

2 ◯

3 ◯

4 ◯

5 ◯

Intuitive and consistent

92. Section 3. Aesthetics: Is the quality of the graphic resources presented in the application adequate? *

Marca solo un óvalo.

	It looks like it was made by amateurs
1	◯
2	◯
3	◯
4	◯
5	◯
	Made by professionals

93. Section 3. Aesthetics: How good does the app look? *

Marca solo un óvalo.

	Ugly
1	◯
2	◯
3	◯
4	◯
5	◯
	Excellent

94. Section 4. Information: Is the content of the application well written and is it relevant to the purpose of the application? *

Marca solo un óvalo.

	Irrelevant
1	⬭
2	⬭
3	⬭
4	⬭
5	⬭
	Quite appropriate

95. Section 4. Information: Is the information presented understandable and concise? *

Marca solo un óvalo.

	Minimal or excessive
1	◯
2	◯
3	◯
4	◯
5	◯
	Understandable, concise and just the necessary

96. Section 4. Information: Is the visual explanation of concepts through graphs, images, videos, among others, clear and correct? *

Marca solo un óvalo.

	Nothing clear
1	◯
2	◯
3	◯
4	◯
5	◯
	Perfectly clear and correct

97. Section 4. Information: Does the information presented in the application seem reliable? *

Marca solo un óvalo.

Lacks credibility
1 ⬭
2 ⬭
3 ⬭
4 ⬭
5 ⬭
Definitely reliable

98. Section 5. Subjective Quality: I would recommend the application to people who could use it *

Marca solo un óvalo.

I would not recommend it

1 ◯

2 ◯

3 ◯

4 ◯

5 ◯

I would recommend it

99. Section 5. Subjective Quality: How many times do you think you would use the application in the coming months if you consider it relevant to you? *

Marca solo un óvalo.

- ◯ None
- ◯ 1 to 2
- ◯ 3 to 5
- ◯ 10 to 50
- ◯ More than 50

100. Section 5. Subjective Quality: Overall, how would you rate the app? *

Marca solo un óvalo.

One of the worst apps I've ever used

1 ⬭

2 ⬭

3 ⬭

4 ⬭

5 ⬭

One of the best apps I've ever used

101. Section 6. Impact on health: The application increased my awareness of the importance of taking care of my health *

Marca solo un óvalo.

	Strongly disagree
1	◯
2	◯
3	◯
4	◯
5	◯
	Totally agree

102. Section 6. Impact on health: The application increased my knowledge and understanding of health care *

Marca solo un óvalo.

	Strongly disagree
1	⬭
2	⬭
3	⬭
4	⬭
5	⬭
	Totally agree

103. Section 6. Impact on health: The application changed my behavior and attitudes that can help me improve my health *

Marca solo un óvalo.

	Strongly disagree
1	◯
2	◯
3	◯
4	◯
5	◯
	Totally agree

104. Section 6. Health impact: The app increased my intention/motivation to improve my healthy behaviors *

Marca solo un óvalo.

	Strongly disagree
1	⬭
2	⬭
3	⬭
4	⬭
5	⬭
	Totally agree

105. Section 6. Impact on health: The application motivated me to seek help with behaviors that affect my health (if I need it) *

Marca solo un óvalo.

	Strongly disagree
1	⬭
2	⬭
3	⬭
4	⬭
5	⬭
	Totally agree

106. Section 6. Impact on the environment: The application increased my awareness of the importance of caring for the environment *

Marca solo un óvalo.

	Strongly disagree
1	◯
2	◯
3	◯
4	◯
5	◯
	Totally agree

107. Section 6. Impact on the environment: The application increased my knowledge and understanding of caring for the environment *

Marca solo un óvalo.

Strongly disagree

1 ◯

2 ◯

3 ◯

4 ◯

5 ◯

Totally agree

108. Section 6. Impact on the environment: The application changed my behavior and attitudes that can help me reduce my environmental impact *

Marca solo un óvalo.

Strongly disagree

1 ◯

2 ◯

3 ◯

4 ◯

5 ◯

Totally agree

109. Section 6. Impact on the environment: The application increased my intention/motivation to reduce my unsustainable behaviors *

Marca solo un óvalo.

	Strongly disagree
1	◯
2	◯
3	◯
4	◯
5	◯
	Totally agree

110. Section 6. Impact on health: The application motivated me to seek help about behaviors that affect the environment *

Marca solo un óvalo.

	Strongly disagree
1	◯
2	◯
3	◯
4	◯
5	◯
	Totally agree

111. Feedback about the application

____
____
____
____
____

Este contenido no ha sido creado ni aprobado por Google.

Google Formularios
