# Supplementary material for: Sustainable-psycho-nutritional intervention programme for a sustainable diet (the ‘NutriSOS’ study) and its effects on eating behaviour, diet quality, nutritional status, physical activity, metabolic biomarkers, gut microbiota and water and carbon footprints in Mexican population: study protocol of an mHealth randomised controlled trial
Source: Br J Nutr. 2023 Mar 30;130(10):1823–38. doi: 10.1017/S0007114523000843 (PMC10587386; doi:10.1017/S0007114523000843)
Supplement: Supplementary file 1 [file S0007114523000843sup.zip › S0007114523000843sup002.zip!/Online Supplementary Material 3 Feasibility, Acceptability, Quality and Usability Questionnaire Spanish.pdf]

Cuestionario para evaluar la Viabilidad, Aceptabilidad, Calidad y Usabilidad de la aplicación móvil NutriSOS®

¡Hola! Bienvenido al cuestionario de evaluación de la aplicación nutricional sostenible NutriSOS®. Nos complace platicarle brevemente sobre este desarrollo tecnológico que hemos creado el Tecnológico Nacional de México, campus Ciudad Guzmán y el Instituto de Investigaciones en Comportamiento Alimentario y Nutrición (IICAN) de la Universidad de Guadalajara. Ésta es una aplicación móvil cuyo objetivo es ayudar a que la población mexicana adopte una dieta sostenible, que mejore su salud y cuide al medio ambiente al mismo tiempo, respetando su cultura y aspectos económicos en todo momento. Sin embargo, para lograrlo, primero debemos validar la aplicación, por lo que agradecemos su apoyo al dedicar tiempo en responder este cuestionario, cuyo objetivo es evaluar la viabilidad, aceptabilidad, calidad y usabilidad de NutriSOS®. No obstante, recuerde que su participación es completamente voluntaria y si en algún momento desea abandonar el estudio podrá hacerlo sin ninguna repercusión negativa hacia su persona.

*Obligatorio

1. ¿Acepta participar en este estudio? *

Marca solo un óvalo.

- Si
- No

Datos del participante

2. Nombre *

3. Apellido Paterno *

____

4. Apellido Materno *

____

5. Edad *

____

6. Sexo *

Marca solo un óvalo.

- ◯ Masculino
- ◯ Femenino
- ◯ Prefiero no decirlo
- ◯ Otro: ______________________

7. ¿Completó el registro inicial dentro la aplicación NutriSOS®? *

Marca solo un óvalo.

- ◯ Sí
- ◯ No

Viabilidad

A continuación se presentan algunas preguntas relacionadas con la viabilidad de la aplicación móvil nutricional sostenible NutriSOS®.

Por favor conteste de la manera más honesta posible, recuerde que no hay respuestas correctas o incorrectas.

8. Sección 1. Inicio: ¿Fue sencillo registrarse en la aplicación? *

Marca solo un óvalo.

- Sí
- No

9. Si su respuesta fue no, describa el porqué.

10. Sección 1. Inicio: ¿Fue sencillo iniciar sesión una vez registrado? *

Marca solo un óvalo.

- Sí
- No
- Más o menos

11. Sección 2.1. Cuestionario Datos Generales: ¿Las preguntas fueron sencillas de responder? *

Marca solo un óvalo.

- Nada fáciles
- Un poco
- Normal
- Fáciles
- Muy fáciles

12. Sección 2.1. Cuestionario Datos Generales: ¿Hubo alguna pregunta que resultara complicada de responder? *

Marca solo un óvalo.

- Sí
- No

13. Si su respuesta fue sí, mencione cuál y por favor describa porqué fue así

____
____
____
____
____

14. Sección 2.1. Cuestionario Datos Generales: ¿Le pareció aburrido responder a las preguntas sobre datos generales? *

Marca solo un óvalo.

- Nada aburrido
- Muy poco
- Moderadamente aburrido
- Muy aburrido
- Extremadamente aburrido

15. Sección 2.2. Cuestionario Sobre Ti: ¿Las preguntas fueron sencillas de responder? *

Marca solo un óvalo.

- Nada fáciles
- Un poco
- Normal
- Fáciles
- Muy fáciles

16. Sección 2.2. Cuestionario Sobre Ti: ¿Hubo alguna pregunta que resultara complicada de responder? *

Marca solo un óvalo.

- Sí
- No

17. Si su respuesta fue sí, mencione cuál y por favor describa porqué fue así

18. Sección 2.2. Cuestionario Sobre Ti: ¿Le pareció aburrido responder a las preguntas sobre usted? *

Marca solo un óvalo.

- Nada aburrido
- Muy poco
- Moderadamente aburrido
- Muy aburrido
- Extremadamente aburrido

19. Sección 2.3. Cuestionario Historia Clínica: ¿Las preguntas fueron sencillas de responder? *

Marca solo un óvalo.

- Nada fáciles
- Un poco
- Normal
- Fáciles
- Muy fáciles

20. Sección 2.3. Cuestionario Historia Clínica: ¿Hubo alguna pregunta que resultara complicada de responder? *

Marca solo un óvalo.

- Sí
- No

21. Si su respuesta fue sí, mencione cuál y por favor describa porqué fue así

22. Sección 2.3: Cuestionario Historia Clínica: ¿Le pareció aburrido responder a las preguntas sobre historia clínica? *

Marca solo un óvalo.

- Nada aburrido
- Muy poco
- Moderadamente aburrido
- Muy aburrido
- Extremadamente aburrido

23. Sección 2.4: Cuestionario Nivel Socio-Económico: ¿Las preguntas fueron sencillas de responder? *

Marca solo un óvalo.

- Sí
- No

24. Sección 2.4: Cuestionario Nivel Socio-Económico: ¿Hubo alguna pregunta que resultara complicada de responder? *

Marca solo un óvalo.

- Sí
- No

25. Si su respuesta fue sí, mencione cuál y por favor describa porqué fue así

26. Sección 2.4: Cuestionario Nivel Socio-Económico: ¿Le pareció aburrido responder a las preguntas sobre nivel socio-económico? *

Marca solo un óvalo.

- Nada aburrido
- Muy poco
- Moderadamente aburrido
- Muy aburrido
- Extremadamente aburrido

27. Sección 2.5: Cuestionario Alimentación: ¿Fue sencillo responder a las preguntas sobre alimentación? *

Marca solo un óvalo.

- Sí
- No

28. Sección 2.5: Cuestionario Alimentación: ¿Hubo alguna pregunta que resultara complicada de responder? *

Marca solo un óvalo.

- Sí
- No

29. Si su respuesta fue sí, mencione cuál y por favor describa porqué fue así

30. Sección 2.5: Cuestionario Alimentación: ¿Le pareció aburrido responder a las preguntas sobre alimentación? *

Marca solo un óvalo.

- Nada aburrido
- Muy poco
- Moderadamente aburrido
- Muy aburrido
- Extremadamente aburrido

31. Sección 2.6. Recordatorio 24 horas: ¿Fue sencillo ingresar los alimentos? *

Marca solo un óvalo.

- Muy sencillo
- Sencillo
- Normal
- Un poco complicado
- Complicado

32. Sección 2.6. Recordatorio 24 horas: ¿Hubo algún aspecto que le pareciera complicado de contestar? *

Marca solo un óvalo.

- Sí
- No

33. Si su respuesta fue sí, mencione cuál y por favor describa porqué fue así

34. Sección 2.6. Recordatorio 24 horas: ¿Le pareció aburrido responder el recordatorio de 24 horas, es decir, ingresar los alimentos que consumió el día previo a la evaluación? *

Marca solo un óvalo.

- Nada aburrido
- Muy poco
- Moderadamente aburrido
- Muy aburrido
- Extremadamente aburrido

35. Sección 3. Guía alimentaria nutricional sostenible interactiva: ¿Los colores de la pirámide son agradables de observar? *

Marca solo un óvalo.

- No son agradables
- Están bien
- Son agradables

36. Sección 4. Plan alimenticio: ¿Hay algo que modificaría en la sección de plan alimenticio? *

Marca solo un óvalo.

- Sí
- No

37. Si su respuesta fue sí, mencione qué y por favor describa la modificación

38. Sección 5 . Registros dietéticos: ¿Fue sencillo ingresar los alimentos? *

Marca solo un óvalo.

- Muy sencillo
- Moderadamente sencillo
- Complicado

39. Sección 5. Registros dietéticos: ¿Hubo algún aspecto que le pareciera complicado de contestar? *

Marca solo un óvalo.

- Sí
- No

40. Si su respuesta fue sí, mencione cuál y por favor describa porqué fue así

41. Sección 6. Recetas: ¿Fue sencillo ingresar a la sección de recetas? *

Marca solo un óvalo.

- Sí
- Más o menos
- No

42. Sección 6. Recetas: ¿El contenido de los vídeos es claro? *

Marca solo un óvalo.

- Sí
- Más o menos
- No

43. Sección 6. Recetas: ¿Modificaría algo en los vídeos? *

Marca solo un óvalo.

- Sí
- No

44. Si su respuesta fue sí, mencione qué y por favor describa la modificación

45. Sección 6. Recetas: ¿Modificaría algo sobre las recetas? *

Marca solo un óvalo.

- Sí
- No

46. Si su respuesta fue sí, mencione qué y por favor describa la modificación

47. Sección 7. Aprendiendo: ¿El contenido de los vídeos es claro? *

Marca solo un óvalo.

- No es nada claro
- No responde todas mis preguntas
- Es claro
- Es bastante claro

48. Sección 7. Aprendiendo: ¿Modificaría algo en los vídeos? *

Marca solo un óvalo.

- Sí
- No

49. Si su respuesta fue sí, mencione qué y por favor describa la modificación

50. Sección 7. Aprendiendo: ¿Considera que la información proporcionada es útil para aprender sobre nutrición sostenible? *

Marca solo un óvalo.

- Poco útil
- Útil
- Muy útil

51. Sección 8. Comunidad: ¿Le parece agradable utilizar la comunidad? *

Marca solo un óvalo.

- Poco agradable
- Regular
- Agradable
- Muy agradable

52. Sección 8. Comunidad: ¿Estaría dispuesto a subir fotografías sobre los alimentos que consume? *

Marca solo un óvalo.

- Sí
- No

53. Sección 8. Comunidad: ¿Le gustaría ver fotografías de la alimentación de otras personas que tienen objetivos nutricionales sostenibles similares a los suyos? *

Marca solo un óvalo.

- No me gustaría ver
- Muy poco
- Regular
- Me gustaría
- Me encantaría

54. Sección 8. Comunidad: ¿Estaría dispuesto a interactuar con otros usuarios dentro de la comunidad por medio de me gusta, comentarios o subiendo nuevas fotografías? *

Marca solo un óvalo.

- Sí
- No

55. Sección 9. Mensajes: ¿Le parece sencillo enviar y recibir mensajes? *

Marca solo un óvalo.

- Sí
- No

56. Sección 9. Mensajes: ¿Hay algo que modificaría en la sección de mensajes? *

Marca solo un óvalo.

- Sí
- No

57. Si su respuesta fue sí, mencione qué y por favor describa la modificación

58. Sección 10. Perfil: ¿Le parece sencillo editar su foto de perfil? *

Marca solo un óvalo.

- Sí
- Más o menos
- No

59. Sección 10. Perfil: ¿Las gráficas de evolución le parecen fáciles de interpretar? *

Marca solo un óvalo.

- Muy sencillas
- Regular
- Complicadas

60. Sección 10. Perfil: ¿Hay algo que modificaría en la sección de perfil? *

Marca solo un óvalo.

- Sí
- No
- Tal vez

61. Si su respuesta fue sí, mencione qué y por favor describa la modificación

62. Sección 11. Recordatorios: ¿Le parece agradable recibir recordatorios en la aplicación? *

Marca solo un óvalo.

- Agradable
- Poco agradable
- Moderadamente molesto
- Muy molesto
- Extremadamente molesto

63. Sección 11. Recordatorios: ¿Le parecen útiles los recordatorios'? *

Marca solo un óvalo.

- Poco útiles
- Regular
- Muy útiles

64. Sección 11. Recordatorios: ¿Hay algo sobre los recordatorios en la aplicación que le gustaría modificar? *

Marca solo un óvalo.

- Sí
- No

65. Si su respuesta fue sí, mencione qué y por favor describa la modificación

Aceptabilidad

A continuación se presentan algunas preguntas relacionadas con la aceptabilidad de la aplicación móvil nutricional sostenible NutriSOS®.

Por favor conteste de la manera más honesta posible, recuerde que no hay respuestas correctas o incorrectas.

66. Sección 1. Diseño: ¿El tamaño de la letra le parece adecuado? *

Marca solo un óvalo.

- Sí
- No
- Más o menos

67. Sección 1. Diseño: ¿Los colores de la aplicación le parecen adecuados? *

Marca solo un óvalo.

- Sí
- No
- Más o menos

68. Sección 1. Diseño: ¿Hay algo que cambiaría de la aplicación? *

Marca solo un óvalo.

- Sí
- No

69. Si su respuesta fue sí, mencione qué y por favor describa la modificación

70. Sección 2. Uso de la aplicación: ¿Seguiría usando la aplicación? *

Marca solo un óvalo.

- Para nada
- No lo sé
- Definitivamente

71. Por favor describa el porqué de su respuesta

72. Sección 2. Uso de la aplicación: ¿Le aburrió usar la aplicación? *

Marca solo un óvalo.

- Para nada
- Un poco
- Regular
- Bastante

73. Sección 2. Uso de la aplicación: ¿En algún momento se sintió frustrado usando la aplicación? *

Marca solo un óvalo.

- Nunca
- En ocasiones
- Todo el tiempo

Usabilidad

A continuación se presentan algunas preguntas relacionadas con la usabilidad de la aplicación móvil nutricional sostenible NutriSOS®.

Por favor conteste de la manera más honesta posible, recuerde que no hay respuestas correctas o incorrectas.

74. ¿Usaría la aplicación con frecuencia? *

Marca solo un óvalo.

- Para nada
- No lo sé
- Definitivamente

75. Si su respuesta fue no lo sé, por favor describa el porqué

76. Encuentro la aplicación innecesariamente complicada *

Marca solo un óvalo.

	Para nada complicada
1	◯
2	◯
3	◯
4	◯
5	◯
	Bastante complicada

77. Creo que la aplicación es fácil de usar *

Marca solo un óvalo.

Para nada fácil

1 ◯
2 ◯
3 ◯
4 ◯
5 ◯

Bastante fácil

78. Necesita asesoramiento para el uso de la aplicación *

Marca solo un óvalo.

◯ Nunca
◯ En ocasiones
◯ Todo el tiempo

79. Las funcionalidades de la aplicación se integran adecuadamente *

Marca solo un óvalo.

◯ No
◯ Más o menos
◯ Sí

80. La aplicación tenía muchas inconsistencias *

Marca solo un óvalo.

Para nada inconsistente

1 ○

2 ○

3 ○

4 ○

5 ○

Bastante inconsistente

81. Considero que la mayoría de las personas podrían aprender a usar la aplicación rápidamente *

Marca solo un óvalo.

Para nada

1 ◯

2 ◯

3 ◯

4 ◯

5 ◯

Definitivamente

82. Me siento cómodo utilizando la aplicación *

Marca solo un óvalo.

	Nada cómodo
1	◯
2	◯
3	◯
4	◯
5	◯
	Definitivamente cómodo

83. Necesito aprender muchas cosas antes de poder utilizar la aplicación *

Marca solo un óvalo.

	No necesito aprender nada
1	◯
2	◯
3	◯
4	◯
5	◯
	Necesito aprender mucho

Calidad

A continuación se presentan algunas preguntas relacionadas con la calidad de la aplicación móvil nutricional sostenible NutriSOS®.

Por favor conteste de la manera más honesta posible, recuerde que no hay respuestas correctas o incorrectas.

84. Sección 1. Interacción: ¿La aplicación es divertida/entretenida de usar? *

Marca solo un óvalo.

Nada entretenida

1 ◯

2 ◯

3 ◯

4 ◯

5 ◯

Muy entretenida

85. Sección 1. Interacción: ¿La aplicación presenta información interesante en comparación con otras similares? *

Marca solo un óvalo.

Nada interesante

1 ⬭

2 ⬭

3 ⬭

4 ⬭

5 ⬭

Muy interesante

86. Sección 1. Interacción: ¿Permite la personalización en configuración y preferencias? *

Marca solo un óvalo.

	No la permite
1	◯
2	◯
3	◯
4	◯
5	◯
	Permite personalizar cualquier característica

87. Sección 1. Interacción: ¿Permite ingresar información, genera recomendaciones y provee retroalimentación? *

Marca solo un óvalo.

Bajo nivel de retroalimentación e interacción
1 ◯
2 ◯
3 ◯
4 ◯
5 ◯
Gran nivel de retroalimentación e interacción

88. Sección 1. Interacción: ¿El contenido de la aplicación es apropiado para el público objetivo? *

Marca solo un óvalo.

Completamente inapropiado
1
2
3
4
5

Diseñado para la audiencia objetivo

89. Sección 2. Funcionalidad: ¿Qué tan fluido funcionan los componentes y en general la aplicación? *

Marca solo un óvalo.

	La aplicación está rota
1	◯
2	◯
3	◯
4	◯
5	◯
	Funciona perfecto, sin problemas encontrados

90. Sección 2. Funcionalidad: La navegación entre las diferentes secciones de la aplicación tiene sentido *

Marca solo un óvalo.

	No hay conexión, navegación díficil
1	◯
2	◯
3	◯
4	◯
5	◯
	Intuitivo y fácil de navegar

91. Sección 2. Funcionalidad: Las interacciones: clics, scroll, swipes de la aplicación tienen sentido *

Marca solo un óvalo.

	No hay consistencia
1	◯
2	◯
3	◯
4	◯
5	◯
	Intuitivo y consistente

92. Sección 3. Estética: ¿Es adecuada la calidad de los recursos gráficos presentados en la aplicación? *

Marca solo un óvalo.

Parece hecha por aficionados

1 ◯

2 ◯

3 ◯

4 ◯

5 ◯

Hecha por profesionales

93. Sección 3. Estética: ¿Qué tan bien luce la aplicación? *

Marca solo un óvalo.

Fea

1 ◯

2 ◯

3 ◯

4 ◯

5 ◯

Excelente

94. Sección 4. Información: ¿El contenido de la aplicación está bien escrito y es relevante para el objetivo de ésta? *

Marca solo un óvalo.

Irrelevante

1 ○

2 ○

3 ○

4 ○

5 ○

Bastante apropiada

95. Sección 4. Información: ¿La información presentada es comprensible y concisa? *

Marca solo un óvalo.

Mínima o excesiva

1 ⬭

2 ⬭

3 ⬭

4 ⬭

5 ⬭

Comprensible, concisa y justo la necesaria

96. Sección 4. Información: ¿La explicación visual de conceptos mediante gráficas, imágenes, vídeos, entre otros es clara y correcta? *

Marca solo un óvalo.

	Nada clara
1	◯
2	◯
3	◯
4	◯
5	◯
	Perfectamente clara y correcta

97. Sección 4. Información: ¿La información presentada en la aplicación parece confiable? *

Marca solo un óvalo.

	Carece de credibilidad
1	◯
2	◯
3	◯
4	◯
5	◯
	Definitivamente confiable

98. Sección 5. Calidad Subjetiva: Recomendaría la aplicación con personas a las que les podría servir *

Marca solo un óvalo.

	No la recomendaría
1	◯
2	◯
3	◯
4	◯
5	◯
	La recomendaría

99. Sección 5. Calidad Subjetiva: ¿Cuántas veces cree que usaría la aplicación en los próximos meses si la considera relevante para usted? *

Marca solo un óvalo.

- ◯ Ninguna
- ◯ De 1 a 2
- ◯ De 3 a 5
- ◯ De 10 a 50
- ◯ Más de 50

100. Sección 5. Calidad Subjetiva: En general, ¿Cómo calificas la aplicación? *

Marca solo un óvalo.

	Una de las peores aplicaciones que he usado
1	◯
2	◯
3	◯
4	◯
5	◯
	Una de las mejores aplicaciones que he usado

101. Sección 6. Impacto a la salud: La aplicación aumentó mi conciencia sobre la importancia de cuidar mi salud *

Marca solo un óvalo.

	En total desacuerdo
1	◯
2	◯
3	◯
4	◯
5	◯
	Totalmente de acuerdo

102. Sección 6. Impacto a la salud: La aplicación aumentó mi conocimiento y entendimiento sobre el cuidado de la salud *

Marca solo un óvalo.

	En total desacuerdo
1	
2	
3	
4	
5	
	Totalmente de acuerdo

103. Sección 6. Impacto a la salud: La aplicación cambió mi comportamiento y actitudes que pueden ayudarme a mejorar mi salud *

Marca solo un óvalo.

	En total desacuerdo
1	◯
2	◯
3	◯
4	◯
5	◯
	Totalmente de acuerdo

104. Sección 6. Impacto a la salud: La aplicación incrementó mi intención/motivación para mejorar mis conductas saludables *

Marca solo un óvalo.

En total desacuerdo

1 ⬭

2 ⬭

3 ⬭

4 ⬭

5 ⬭

Totalmente de acuerdo

105. Sección 6. Impacto a la salud: La aplicación me motivó a buscar ayuda sobre las conductas que afectan mi salud (en caso de que la necesite) *

Marca solo un óvalo.

En total desacuerdo

1 ◯

2 ◯

3 ◯

4 ◯

5 ◯

Totalmente de acuerdo

106. Sección 6. Impacto al medio ambiente: La aplicación aumentó mi conciencia sobre la importancia de cuidar el medio ambiente *

Marca solo un óvalo.

	En total desacuerdo
1	◯
2	◯
3	◯
4	◯
5	◯
	Totalmente de acuerdo

107. Sección 6. Impacto al medio ambiente: La aplicación aumentó mi conocimiento y entendimiento sobre el cuidado del medio ambiente *

Marca solo un óvalo.

En total desacuerdo

1 ◯

2 ◯

3 ◯

4 ◯

5 ◯

Totalmente de acuerdo

108. Sección 6. Impacto al medio ambiente: La aplicación cambió mi comportamiento y actitudes que pueden ayudarme a disminuir mi impacto medioambiental *

Marca solo un óvalo.

En total desacuerdo

1 ⬭

2 ⬭

3 ⬭

4 ⬭

5 ⬭

Totalmente de acuerdo

109. Sección 6. Impacto al medio ambiente: La aplicación incrementó mi intención/motivación para disminuir mis conductas insostenibles *

Marca solo un óvalo.

En total desacuerdo

1 ⬭

2 ⬭

3 ⬭

4 ⬭

5 ⬭

Totalmente de acuerdo

110. Sección 6. Impacto a la salud: La aplicación me motivó a buscar ayuda sobre las conductas que afectan al medio ambiente *

Marca solo un óvalo.

	En total desacuerdo
1	⬭
2	⬭
3	⬭
4	⬭
5	⬭
	Totalmente de acuerdo

111. Comentarios sobre la aplicación

____
____
____
____
____

Este contenido no ha sido creado ni aprobado por Google.

Google Formularios
