# Supplementary material for: Sustainable-psycho-nutritional intervention programme for a sustainable diet (the ‘NutriSOS’ study) and its effects on eating behaviour, diet quality, nutritional status, physical activity, metabolic biomarkers, gut microbiota and water and carbon footprints in Mexican population: study protocol of an mHealth randomised controlled trial
Source: Br J Nutr. 2023 Mar 30;130(10):1823–38. doi: 10.1017/S0007114523000843 (PMC10587386; doi:10.1017/S0007114523000843)
Supplement: Supplementary file 1 [file S0007114523000843sup.zip › S0007114523000843sup002.zip!/Online Supplementary Material 4. Initial questionnaire NutriSo English.pdf]

NutriSOS: Sustainable Nutrition for your Health and the Environment, respecting your Economy and your Culture

Hello! :) First of all, thank you very much for agreeing to open this link. We tell you a little about why we invite you to answer this questionnaire...

Today, the world and Mexico are going through the worst environmental crisis in history, where every day we run out of water and climate change has caused atmospheric temperatures to increase a lot! (more than 1 degree Celsius since the industrial revolution), which can lead to many natural disasters.

In addition to this, many people in Mexico are sick from causes related to nutrition. For example, more than 70% of Mexicans are overweight or obese, or have type 2 diabetes, high blood pressure or problems such as high cholesterol and triglycerides.

We are currently working on a Sustainable-Psycho-Nutritional intervention program, which promotes the consumption of a sustainable diet, that is, a healthy diet, with low environmental impact, that is economical and adapts to the culture of the Mexican population.

With this intervention, you will be able to reduce or maintain your glucose, cholesterol, triglycerides, and intestinal microbiota at optimal levels. In addition, you will be able to help reduce water scarcity, by reducing the water used in the production of the food we eat (water footprint), and help combat climate change, by reducing the greenhouse gases generated by production. of food (carbon footprint).

For this project, we have designed and developed a mobile application that will be very helpful, the NutriSOS App. Therefore, if you are selected to participate in this study, you will have FREE nutritional advice!!!

Help us with your answer, and thank you for your participation, for your health and that of the planet!

*Obligatorio

1. Having said the above, we want to ask you, would you accept to answer a series of questions to find out if you can participate in our study? *

   Marca solo un óvalo.

   - ◯ Yes, I agree to answer the questionnaire
   - ◯ No, I do not agree to answer the questionnaire

2. If you have the characteristics we are looking for in our study, would you like to participate in it? *

   Marca solo un óvalo.

   - ◯ Yes
   - ◯ No

General questions

Please answer as honestly as possible. All the information you share with us is confidential and is protected by the Federal Law on the Protection of Personal Data Held by Private Parties.

3. You consider yourself... *

Marca solo un óvalo.

- Man
- Woman
- I prefer not to say
- Indefinite sex
- Otro: ________________

4. How old are you? *

____

5. What is your job? *

Selecciona todos los que correspondan.

- [ ] Student
- [ ] Housewife
- [ ] Officer, director or boss
- [ ] Professional or technician
- [ ] Auxiliary worker in administrative activities
- [ ] Merchant, sales clerk, or sales agent
- [ ] Workers in personal and surveillance services
- [ ] Worker in agricultural, livestock, forestry, hunting or fishing activities
- [ ] Craft worker
- [ ] Industrial machinery operator, assemblers, chauffeur or transport driver
- [ ] Workers in elementary and support activities
- [ ] Unemployed
- [ ] Retired
- [ ] Otro: ________________

6. Up to what school grade did you attend? *

Marca solo un óvalo.

- I did not study
- Unfinished primary
- Primary finished
- Unfinished high school
- High school finished
- Unfinished preparatory school
- Finished preparatory school
- Unfinished technical career
- Technical career finished
- Undergraduate student
- Unfinished degree
- Completed bachelor's degree
- Masters
- PhD
- Specialty
- Otro: ____________________

7. On average, what is your monthly income in MXN pesos? *

Marca solo un óvalo.

- 0 - 2,699
- 2,700 - 6,799
- 6,800 - 11,599
- 11,600 - 34,999
- 35,000 - 84,999
- + 85,000

8. Do you have a cell phone that allows you to install mobile applications? *

Marca solo un óvalo.

- Yes
- No

9. If you answered yes... Is it Android or iOS (IPhone)? *

Marca solo un óvalo.

- Android
- iOS (IPhone)
- Otro: ____________________

10. Please mention the name of the city or town where you were born (include the country if it is outside of Mexico, please) *

11. What city or town do you live in? *

12. How long have you lived there? *

Marca solo un óvalo.

- Less than 1 month
- 1 to 6 months
- 6 to 12 months
- 1 to 3 years
- 3 to 6 years
- 6 to 9 years
- 10 years or more
- Lifetime

Nutritional clinical history

Now, we want to know a little more about your health. Please answer honestly. Remember that all your information is confidential.

13. How much do you weigh in kilograms? *

14. How tall are you in centimeters? *

15. Do you suffer from any chronic disease, such as type 2 diabetes mellitus, high blood pressure, dyslipidemia (high cholesterol and triglycerides)? *

Marca solo un óvalo.

- Yes
- No

16. If you answered yes, please mention which one *

Selecciona todos los que correspondan.

- [ ] Type 2 diabetes
- [ ] Arterial hypertension
- [ ] High cholesterol
- [ ] Elevated triglycerides
- [ ] I do not suffer from any of these diseases
- [ ] Otro: ____________________

17. If you answered yes, mention if you take any medication and indicate which one, please *

Selecciona todos los que correspondan.

- [ ] Metformin
- [ ] Insulin
- [ ] Losartan
- [ ] Telmisartan
- [ ] Bezafibrate
- [ ] I don't take any of those drugs
- [ ] Otro: ____________________

18. Do you suffer from any autoimmune disease such as type 1 diabetes, hypo or hyperthyroidism, rheumatoid arthritis? *

Marca solo un óvalo.

- ( ) Yes
- ( ) No

19. If you answered yes, please mention which one *

Selecciona todos los que correspondan.

- [ ] Type 1 diabetes
- [ ] Hypothyroidism
- [ ] Hyperthyroidism
- [ ] Rheumatoid arthritis
- [ ] I do not suffer from any of these diseases
- [ ] Otro: ____________________

20. If you answered yes, mention if you take any medication and indicate which one, please *

Selecciona todos los que correspondan.

- [ ] Metformin
- [ ] Insulin
- [ ] Levotiroxine
- [ ] Iodine
- [ ] ibuprofen
- [ ] I don't take any of those drugs
- [ ] Otro: ____________________

21. Have you ever been diagnosed with any gastrointestinal disease, such as gastritis, chronic constipation, Crohn's disease, ulcerative colitis? *

Marca solo un óvalo.

- ( ) Yes
- ( ) No

22. If you answered yes, please mention which one *

Selecciona todos los que correspondan.

- [ ] Gastritis
- [ ] Nervous colitis
- [ ] Ulcerative colitis
- [ ] Chronic constipation
- [ ] Crohn's disease
- [ ] I do not suffer from any of these diseases
- [ ] Otro: ____________________

23. If you answered yes, mention if you take any medication and indicate which one, please *

Selecciona todos los que correspondan.

- [ ] Omeprazole
- [ ] Pantoprazole
- [ ] Trimebutine
- [ ] Mesalazine
- [ ] Cinitapride
- [ ] I don't take any of those drugs
- [ ] Otro: ____________________

24. Have you ever been diagnosed with a mood disorder such as depression or anxiety, or an eating disorder such as anorexia or bulimia? *

Marca solo un óvalo.

- ( ) Yes
- ( ) No

25. If you answered yes, please mention which one *

Selecciona todos los que correspondan.

- [ ] Depression
- [ ] Anxiety
- [ ] Bipolar disorder
- [ ] Anorexia nervosa
- [ ] Bulimia nerviosa
- [ ] Binge eating disorder
- [ ] I do not suffer from any of these alterations
- [ ] Otro: ______________________

26. If you answered yes, mention if you take any medication and indicate which one, please *

Selecciona todos los que correspondan.

- [ ] Fluoxetine
- [ ] Sertraline
- [ ] Clonazepam
- [ ] CBD
- [ ] I don't take any of those drugs
- [ ] Otro: ______________________

27. Do you currently take any supplements? For example, probiotics, omega 3, multivitamin, protein powder *

Marca solo un óvalo.

- ( ) Yes
- ( ) No

28. If you answered yes, please mention which one *

Selecciona todos los que correspondan.

- [ ] Omega 3
- [ ] Probiotics
- [ ] Prebiotics
- [ ] Synbiotics
- [ ] Multivitamin
- [ ] Vitamina D
- [ ] Vitamin C
- [ ] Vitamin B12 or B complex
- [ ] Vitamin E
- [ ] Iron
- [ ] Calcium
- [ ] Protein powder
- [ ] Creatine monohydrate
- [ ] I don't take any of those supplements
- [ ] Otro: ______________________

29. If you answered yes, please mention which brand(s), especially if you consume PROBIOTICS or PREBIOTICS (otherwise, write no) *

30. Do you usually take medications without a prescription, such as paracetamol, ibuprofen, ketorolac, omeprazole? *

Marca solo un óvalo.

- Yes
- No

31. If you answered yes, please mention which one(s) *

Selecciona todos los que correspondan.

- Paracetamol
- Omeprazole
- Laxatives
- Ibuprofen
- Naproxen
- Metamizol
- Ketorolac
- I don't take any of those drugs
- Otro:

32. If you answered yes, mention how often, please *

Marca solo un óvalo.

- Every day, more than once a day
- Every day, once a day
- 4 to 6 times a week
- 2 to 3 times a week
- 1 time per week
- 1 time every 15 days
- 1 time a month
- 1 time every 2 months
- Every 6 months
- Rarely
- I don't take any of those drugs
- Otro:

33. In the last three months have you taken any type of antibiotic? *

Marca solo un óvalo.

- Yes
- No

34. If you answered yes, please mention which one *

Selecciona todos los que correspondan.

- [ ] Ampicillin
- [ ] Cephalexin
- [ ] Ciprofloxacin
- [ ] Clarithromycin
- [ ] Ceftriaxone
- [ ] I have not used any of these medications in the last year
- [ ] Otro: ____________________

35. If you answered yes, mention how long ago you consumed the last cycle *

Marca solo un óvalo.

- 1 week ago
- 1 month ago
- 2 months ago
- 3 months ago
- 4 months ago
- 6 months ago
- 1 year ago
- More than 1 year ago
- I have not used any of these medications in the last year
- Otro: ____________________

36. If your gender is female, are you currently pregnant? (If your gender is male, put no) *

Marca solo un óvalo.

- Yes
- No

37. If your gender is female, are you currently breastfeeding? (If your gender is male, put no) *

Marca solo un óvalo.

- Yes
- No

38. Do you know if you are allergic or intolerant to any food? For example, allergic to peanuts or lactose intolerant *

Marca solo un óvalo.

- Yes
- No
- I do not know

39. If you answered yes, mention which food(s) please *

Selecciona todos los que correspondan.

- Dairy (Lactose)
- Seafood
- Gluten
- Strawberries or berries
- Peanuts
- Nuts
- I am not allergic to any food
- Otro: ____________________

Diet and physical activity history

In this case, we want to know a little more about your diet and the physical activity you do. Remember that you will not be judged by your answers, and they are all confidential.

40. Are you currently following a specific diet? *

Marca solo un óvalo.

- Yes
- No

41. If you answered yes, please mention which one

Selecciona todos los que correspondan.

- To lose weight
- To gain weight
- Ketogenic
- Vegetarian (ovo-lacto, ovo or lacto)
- Vegan (do not consume any food of animal origin, or honey)
- Intermittent fasting
- Mediterranean
- DASH or dietary approach to stop hypertension
- I don't follow any specific diet
- Otro: ____________________

42. How long have you been following this diet? *

Marca solo un óvalo.

- 1 week
- 2 week
- 1 month
- 2 months
- 3 months
- 6 months
- 1 year
- 3 years
- 5 years
- I have followed it all my life
- I don't follow any particular type of diet
- Otro: ____________

43. Who cooks the food you eat? *

Selecciona todos los que correspondan.

- [ ] Me
- [ ] Some relative (mom, dad, aunt, uncle, grandmother, grandfather)
- [ ] Economic Kitchen
- [ ] Restaurants
- [ ] Somebody give it to me
- [ ] Home helper or cook
- [ ] Otro: ____________

44. From 1 to 5, do you often experience anxiety about eating specific foods? *

Marca solo un óvalo.

No anxiety

1 ◯

2 ◯

3 ◯

4 ◯

5 ◯

A lot of anxiety

45. If you experience anxiety, please mention what foods generate it *

46. During the past year, how often have you eaten the following food groups? Foods in parentheses are examples. Please include all the food you eat. If you consume several within a group, add them please. For example, if you eat Mexican-style eggs on Monday and pozole on Sunday, you consume Mexican foods and dishes twice a week. *

Marca solo un óvalo por fila.

	Never or almost never	1 to 3 times a month	1 time per week	2 to 4 times a week	5 to 6 times a week	Once a day	2 to 3 times a day	4 to 6 times a day
**Mexican food and dishes (chilaquiles, Mexican style egg, menudo, quesadillas, tacos, sopes, tortas, tamles, flutes, toast, rice pudding, capitorada, tejuino)**	◯	◯	◯	◯	◯	◯	◯	◯
**Fruits (criolla plum, capulín, nance, papaya, guava, prickly pear, pitaya, soursop, pineapple, apple)**	◯	◯	◯	◯	◯	◯	◯	◯
**Vegetables (tomato, zucchini, squash, onion, green tomato, mushrooms, huitlacoche, chayote, jicama)**	◯	◯	◯	◯	◯	◯	◯	◯
**Whole grains and tubers (corn tortilla, amaranth, sweet potato, rice, pasta, whole wheat bread)**	◯	◯	◯	◯	◯	◯	◯	◯

**Legumes (beans, lentils, chickpeas)**								

47. During the past year, how often have you eaten the following food groups? Foods in parentheses are examples. Please include all the food you eat. If you consume several within a group, add them please. For example, if you eat Mexican-style eggs on Monday and pozole on Sunday, you consume Mexican foods and dishes twice a week. *

Marca solo un óvalo por fila.

	Never or almost never	1 to 3 times a month	1 time per week	2 to 4 times a week	5 to 6 times a week	Once a day	2 to 3 times a day	4 to 6 times a day	Mo tha tim a c
**Seeds and healthy fats (avocado, olive oil, chia, peanuts, pumpkin seed)**									
**Dairy products (milk, yogurt, cottage cheese, fresh cheese, panela**									
**Eggs (whole chicken, egg whites)**									
**Fish and shellfish (tilapia, leguado, octopus, river prawn)**									
**Chicken (in fillet, in pieces)**									

48. During the past year, how often have you eaten the following food groups? Foods in parentheses are examples. Please include all the food you eat. If you consume several within a group, add them please. For example, if you eat Mexican-style eggs on Monday and pozole on Sunday, you consume Mexican foods and dishes twice a week. *

Marca solo un óvalo por fila.

	Never or almost never	1 to 3 times a month	1 time per week	2 to 4 times a week	5 to 6 times a week	Once a day	2 to 3 times a day	4 to 6 times a day	[illegible]
**Red and processed meats (beef, pork, sausage, ham)**	◯	◯	◯	◯	◯	◯	◯	◯	
**Ultra-processed foods (soda, cookies, box cereal, instant soups, cupcakes, chips, snack chips)**	◯	◯	◯	◯	◯	◯	◯	◯	
**Added sugar and foods high in trans and saturated fats (table sugar, fresh sweetened water, honey, jams, vegetable shortening, margarine, butter)**	◯	◯	◯	◯	◯	◯	◯	◯	
**Alcoholic beverages (beer, tequila, red wine, pulque)**	◯	◯	◯	◯	◯	◯	◯	◯	
**Insects (crickets, worms)**	◯	◯	◯	◯	◯	◯	◯	◯	
**Salt**	◯	◯	◯	◯	◯	◯	◯	◯	

49. On average, what portion of foods, dishes or drinks do you usually consume each time you consume them PER DAY? For example, if you eat 3 tortillas at breakfast, lunch and dinner, you eat 9 pieces per day *

Selecciona todos los que correspondan.

	0	1/4 piece, cup, or portion	1/2 piece, cup, or portion	1 piece, cup, tablespoon or portion	2 pieces, cups, tablespoons or portions	3 pieces, cups, tablespoons or portions	[illegible]
**Tacos, sopes, enchiladas, flautas**	☐	☐	☐	☐	☐	☐	
**Tamales, tortas, tostadas**	☐	☐	☐	☐	☐	☐	
**Pozole, menudo, mole**	☐	☐	☐	☐	☐	☐	
**Fruits in pieces (apple, orange)**	☐	☐	☐	☐	☐	☐	
**Chopped fruit or small pieces (melon, papaya, grapes)**	☐	☐	☐	☐	☐	☐	

50. On average, what portion of foods, dishes or drinks do you usually consume each time you consume them PER DAY? For example, if you eat 3 tortillas at breakfast, lunch and dinner, you eat 9 pieces per day *

Selecciona todos los que correspondan.

	0	1/4 piece, cup, or portion	1/2 piece, cup, or portion	1 piece, cup, tablespoon or portion	2 pieces, cups, tablespoons or portions	3 pieces, cups, tablespoons or portions	[illegible] ta[illegible] o[illegible]
**Vegetables in pieces (tomato, cucumber)**	☐	☐	☐	☐	☐	☐	
**Vegetables in cups (lettuce, spinach)**	☐	☐	☐	☐	☐	☐	
**Cereals in pieces (corn tortillas, box bread)**	☐	☐	☐	☐	☐	☐	
**Cereals in cups (rice, pasta)**	☐	☐	☐	☐	☐	☐	
**Legumes (beans, lentils)**	☐	☐	☐	☐	☐	☐	

51. On average, what portion of foods, dishes or drinks do you usually consume each time you consume them PER DAY? For example, if you eat 3 tortillas at breakfast, lunch and dinner, you eat 9 pieces per day *

Selecciona todos los que correspondan.

	0	1/4 piece, cup, or portion	1/2 piece, cup, or portion	1 piece, cup, tablespoon or portion	2 pieces, cups, tablespoons or portions	3 pieces, cups, tablespoons or portions	4 [illegible] tabl[illegible] or [illegible]
**Milk and yoghurt**	☐	☐	☐	☐	☐	☐	
**Cheeses**	☐	☐	☐	☐	☐	☐	
**Nuts and peanuts**	☐	☐	☐	☐	☐	☐	
**Chia and pumpkin seeds**	☐	☐	☐	☐	☐	☐	
**Egg and egg whites**	☐	☐	☐	☐	☐	☐	

52. On average, what portion of foods, dishes or drinks do you usually consume each time you consume them PER DAY? For example, if you eat 3 tortillas at breakfast, lunch and dinner, you eat 9 pieces per day *

Selecciona todos los que correspondan.

	0	1/4 piece, cup, or portion	1/2 piece, cup, or portion	1 piece, cup, tablespoon or portion	2 pieces, cups, tablespoons or portions	3 pieces, cups, tablespoons or portions	4 p c table or p [illegible]
**Fish (80 g palm-sized portion)**	☐	☐	☐	☐	☐	☐	
**Seafood (shrimp, octopus)**	☐	☐	☐	☐	☐	☐	
**Chicken (piece or portion of 80 g of the size of the palm of the hand)**	☐	☐	☐	☐	☐	☐	
**Beef or pork (palm-sized 80g portion)**	☐	☐	☐	☐	☐	☐	
**Sweet bread (concha)**	☐	☐	☐	☐	☐	☐	

53. On average, what portion of foods, dishes or drinks do you usually consume each time you consume them PER DAY? For example, if you eat 3 tortillas at breakfast, lunch and dinner, you eat 9 pieces per day *

Selecciona todos los que correspondan.

	0	1/4 piece, cup, or portion	1/2 piece, cup, or portion	1 piece, cup, tablespoon or portion	2 pieces, cups, tablespoons or portions	3 pieces, cups, tablespoons or portions	4 [illegible]
**Sugar, jams, sweets**	☐	☐	☐	☐	☐	☐	
**Soft drinks, fruit juices**	☐	☐	☐	☐	☐	☐	
**Alcoholic drinks**	☐	☐	☐	☐	☐	☐	
**Natural water**	☐	☐	☐	☐	☐	☐	
**Salt**	☐	☐	☐	☐	☐	☐	

54. Do you do physical activity? For example, walking, jogging, running, aerobics, Zumba, dancing, cycling, swimming, crossfit, weights, etc. *

Marca solo un óvalo.

- ◯ Yes
- ◯ No

55. How many days a week do you do physical activity? (for example, walking, running, swimming, weights) *

Marca solo un óvalo.

- ◯ 0
- ◯ 1
- ◯ 2
- ◯ 3
- ◯ 4
- ◯ 5
- ◯ 6
- ◯ 7

56. What kind of physical activity do you practice? *

Selecciona todos los que correspondan.

- [ ] To walk
- [ ] Jogging
- [ ] Run
- [ ] Aerobics, Zumba or dance
- [ ] Cycling
- [ ] Swimming
- [ ] Crossfit
- [ ] Weight lifting
- [ ] Multifunctional
- [ ] Calisthenics
- [ ] Focused exercises (write which ones, e.g. sit-ups, sit-ups, push-ups)
- [ ] I do not do physical activity
- [ ] Otro: ____________________

57. How many minutes of physical activity do you do per day, each time you do it? *

Marca solo un óvalo.

- ( ) 0
- ( ) 10
- ( ) 20
- ( ) 30
- ( ) 40
- ( ) 50
- ( ) 60
- ( ) 90
- ( ) 120
- ( ) 150
- ( ) 180
- ( ) 210
- ( ) 240 or more
- ( ) Otro: ____________________

58. At what intensity do you do it? *

Marca solo un óvalo.

- ( ) Mild (no labored breathing)
- ( ) Moderate (shakes breath)
- ( ) Intense (shakes your breath a lot)
- ( ) Otro: ____________________

Study details

In order to know your levels of glucose, cholesterol, triglycerides, gut microbiota and thus analyze your health, it is necessary that you provide us with some biological samples, therefore, we want to ask you the following.

59. Would you be willing to allow a peripheral blood sample (from your arm) to be drawn to find out your glucose, triglyceride and cholesterol levels? *

Marca solo un óvalo.

- Yes
- No

60. Would you be willing to give us a stool sample (the size of a walnut), to know the composition of your gut microbiota? *

Marca solo un óvalo.

- Yes
- No

61. Would you be willing to attend two 10-minute sessions twice a week via Google Meet? Remember that these are sessions to improve your health and that of the planet *

Marca solo un óvalo.

- Yes
- No

62. Would you be willing to download a mobile application where you receive text messages and have to write down the food you eat and take pictures of it? *

Marca solo un óvalo.

- Yes
- No

63. Finally, please write your cell phone number to contact you if you are selected to participate in this study. Remember that this information is and will always be confidential. *

____

64. Also please share your email *

____

Este contenido no ha sido creado ni aprobado por Google.

Google Formularios
