# Supplementary material for: Sustainable-psycho-nutritional intervention programme for a sustainable diet (the ‘NutriSOS’ study) and its effects on eating behaviour, diet quality, nutritional status, physical activity, metabolic biomarkers, gut microbiota and water and carbon footprints in Mexican population: study protocol of an mHealth randomised controlled trial
Source: Br J Nutr. 2023 Mar 30;130(10):1823–38. doi: 10.1017/S0007114523000843 (PMC10587386; doi:10.1017/S0007114523000843)
Supplement: Supplementary file 1 [file S0007114523000843sup.zip › S0007114523000843sup002.zip!/Online Supplementary Material 5. Initial questionnaire NutriSo Spanish.pdf]

NutriSOS: Nutrición Sostenible para tu Salud y el Medio Ambiente, respetando tu Economía y tu Cultura

¡Hola! :) Primero que nada, muchas gracias por aceptar abrir este link. Te platicamos un poco de porque te invitamos a responder este cuestionario...

Hoy en día, el mundo y México atraviesan por la peor crisis medioambiental de la historia, donde cada día nos quedamos sin agua y el cambio climático ha hecho que las temperaturas atmosféricas incrementen muchísimo! (más de 1 grado centigrado desde la revolución industrial), lo que puede generar que haya muchos desastres naturales.

Además de esto, muchas personas en México están enfermas por causas relacionadas con la nutrición. Por ejemplo, más del 70% de los mexicanos tienen sobrepeso, obesidad, diabetes tipo 2, hipertensión arterial o problemas como colesterol y triglicéridos elevados.

Actualmente estamos trabajando en un programa de intervención Psico-Nutricional-Sostenible, que promueva el consumo de una dieta sostenible, es decir, una dieta saludable, con bajo impacto medioambiental, que sea económica y se adapte a la cultura de la población mexicana.

Con esta intervención, podrás disminuir o mantener en niveles óptimos, tu glucosa, colesterol, triglicéridos, y microbiota intestinal. Además, podrás ayudar a reducir la escacez de agua, al disminuir el agua que se gasta en la producción de los alimentos que comemos (huella hídrica), y ayudar a combatir el cambio climático, al disminuir los gases de efecto invernadero generados por la producción de alimentos (huella de carbono).

Para este proyecto, hemos diseñado y desarrollado una aplicación móvil que será de mucha ayuda, la App NutriSOS®. Por lo que, si eres seleccionado para participar en este estudio, tendrás asesoria nutricional GRATIS!!!

Ayudanos con tu respuesta y gracias por tu participación, ¡por tu salud y por la del planeta!

*Obligatorio

1. Una vez dicho lo anterior, queremos preguntarte, ¿aceptarías responder a una serie de preguntas para saber si puedes participar en nuestro estudio? *

   Marca solo un óvalo.

   - ( ) Si, acepto responder el cuestionario
   - ( ) No, no acepto responder el cuestionario

Preguntas generales

Por favor, responde de la manera más honesta. Toda la información que nos compartas es confidencial y está protegida por la Ley
Federal de Protección de Datos Personales en Posesión de los Particulares.

2. Nombre *

   ____

3. Apellido paterno *

4. Apellido materno *

5. Te consideras: *

Marca solo un óvalo.

- Hombre
- Mujer
- Prefiero no decirlo
- Sexo indefinido
- Otro: ______________________

6. ¿Cuántos años tienes? *

7. ¿A qué te dedicas? *

Selecciona todos los que correspondan.

- Estudiante
- Ama de casa
- Funcionario, director o jefe
- Profesionista o técnico
- Trabajador auxiliar en actividades administrativas
- Comerciante, empleado en ventas o agente de ventas
- Trabajadores en servicios personales y de vigilancia
- Trabajador en actividades agrícolas, ganaderas, forestales, caza o pesca
- Trabajador artesanal
- Operador de maquinaria industrial, ensambladores, chofer o conductor de transporte
- Trabajadores en actividades elementales y de apoyo
- Desempleado
- Jubilado
- Otro: ______________________

8. ¿Hasta que grado escolar cursaste? *

Marca solo un óvalo.

- No estudié
- Primaria inconclusa
- Primaria terminada
- Secundaria inconclusa
- Secundaria terminada
- Preparatoria inconclusa
- Preparatoria terminada
- Carrera técnica inconclusa
- Carrera técnica terminada
- Estudiante de licenciatura
- Licenciatura inconclusa
- Licenciatura terminada
- Maestría
- Doctorado
- Especialidad
- Otro: ______________________

9. En promedio, ¿cuál es tu ingreso mensual en pesos MXN? *

Marca solo un óvalo.

- 0 - 2,699
- 2,700 - 6,799
- 6,800 - 11,599
- 11,600 - 34,999
- 35,000 - 84,999
- + 85,000

10. ¿Tienes un celular que permita instalar aplicaciones móviles? *

Marca solo un óvalo.

- Si
- No

11. Si respondiste que si... ¿Es Android o iOS (IPhone)? *

Marca solo un óvalo.

- Android
- iOS (IPhone)
- Otro: ______________________

12. Menciona por favor el nombre de la ciudad o pueblo donde naciste (incluye el país si es fuera de México, por favor) *

____
____
____
____
____

13. ¿En qué ciudad o pueblo vives? *

____

14. ¿Desde hace cuánto tiempo vives ahí? *

Marca solo un óvalo.

- Menos de 1 mes
- 1 a 6 meses
- 6 a 12 meses
- 1 a 3 años
- 3 a 6 años
- 6 a 9 años
- 10 años o más
- Toda la vida

Historia clínica nutricional

Ahora, queremos conocer un poco más sobre tu salud. Por favor, responde honestamente. Recuerda que toda tu información es confidencial.

15. ¿Cuánto pesas en kilogramos? *

____

16. ¿Cuánto mides en centímetros? *

____

17. ¿Padeces alguna enfermedad crónica, como diabetes mellitus tipo 2, hipertensión arterial, dislipidemias (colesterol y triglicéridos elevados)? *

Marca solo un óvalo.

- Si
- No

18. Si respondiste que si, menciona cuál por favor *

Selecciona todos los que correspondan.

- [ ] Diabetes tipo 2
- [ ] Hipertensión arterial
- [ ] Colesterol elevado
- [ ] Triglicéridos elevados
- [ ] No padezco ninguna de esas enfermedades
- [ ] Otro: ______________________

19. Si respondiste que si, menciona si consumes algún medicamento y señala cuál, por favor *

Selecciona todos los que correspondan.

- [ ] Metformina
- [ ] Insulina
- [ ] Lozartan
- [ ] Telmisartan
- [ ] Bezafibrato
- [ ] No consumo ninguno de esos medicamentos
- [ ] Otro: ______________________

20. ¿Padeces alguna enfermedad autoinmune como diabetes tipo 1, hipo o hipertiroidismo, artritis reumatoide? *

Marca solo un óvalo.

- ( ) Si
- ( ) No

21. Si respondiste que si, menciona cuál por favor *

Selecciona todos los que correspondan.

- [ ] Diabetes tipo 1
- [ ] Hipotiroidismo
- [ ] Hipertiroidismo
- [ ] Artritis reumatoide
- [ ] No padezco ninguna de esas enfermedades
- [ ] Otro: ______________________

22. Si respondiste que si, menciona si tomas algún medicamento y señala cuál, por favor *

Selecciona todos los que correspondan.

- [ ] Metformina
- [ ] Insulina
- [ ] Levotiroxina
- [ ] Yodo
- [ ] Ibuprofeno
- [ ] No consumo ninguno de esos medicamentos
- [ ] Otro: ______________________

23. ¿Alguna vez te han diagnosticado con alguna enfermedad gastrointestinal, como gastritis, estreñimiento crónico, enfermedad de Crohn, colitis ulcerativa? *

Marca solo un óvalo.

- ( ) Si
- ( ) No

24. Si respondiste que si, menciona cuál por favor *

Selecciona todos los que correspondan.

- [ ] Gastritis
- [ ] Colitis nerviosa
- [ ] Colitis ulcerativa
- [ ] Estreñimiento crónico
- [ ] Enfermedad de Crohn
- [ ] No padezco ninguna de esas enfermedades
- [ ] Otro: ______________________

25. Si respondiste que si, menciona si tomas algún medicamento y señala cuál, por favor *

Selecciona todos los que correspondan.

- [ ] Omeprazol
- [ ] Pantoprazol
- [ ] Trimebutina
- [ ] Mesalazina
- [ ] Cinitaprida
- [ ] No consumo ninguno de esos medicamentos
- [ ] Otro: ______________________

26. ¿Alguna vez te han diagnosticado con algún trastorno del estado de ánimo como depresión o ansiedad, o con algún trastorno de la conducta alimentaria, como anorexia o bulimia? *

Marca solo un óvalo.

- Sí
- No

27. Si respondiste que si, menciona cuál por favor *

Selecciona todos los que correspondan.

- Depresión
- Ansiedad
- Trastorno bipolar
- Anorexia nerviosa
- Bulimia nerviosa
- Trastorno por atracón
- No padezco ninguna de esas alteraciones
- Otro: ____________________

28. Si respondiste que si, menciona si tomas algún medicamento y señala cuál, por favor *

Selecciona todos los que correspondan.

- Fluoxetina
- Sertralina
- Clonazepam
- CBD
- No consumo ninguno de esos medicamentos
- Otro: ____________________

29. ¿Actualmente consumes algún suplemento? Por ejemplo, probióticos, omega 3, multivitaminico, proteína en polvo *

Marca solo un óvalo.

- Si
- No

30. Si respondiste que si, menciona cuál por favor *

Selecciona todos los que correspondan.

- [ ] Omega 3
- [ ] Probióticos
- [ ] Prebióticos
- [ ] Sinbióticos
- [ ] Multivitaminico
- [ ] Vitamina D
- [ ] Vitamina C
- [ ] Vitamina B12 o complejo B
- [ ] Vitamina E
- [ ] Hierro
- [ ] Calcio
- [ ] Proteína en polvo
- [ ] Creatina monohidratada
- [ ] No consumo ninguno de esos suplementos
- [ ] Otro: ______________________

31. Si respondiste que si, menciona que marca(s) por favor, especialmente si consumes PROBIÓTICOS o PREBIÓTICOS (sino, escribe no) *

____
____
____
____
____

32. ¿Acostumbras tomar medicamentos sin prescripción médica, como paracetamol, ibuprofeno, ketorolaco, omeprazol? *

Marca solo un óvalo.

- ( ) Si
- ( ) No

33. Si respondiste que si, menciona cuál(es) por favor *

Selecciona todos los que correspondan.

- [ ] Paracetamol
- [ ] Omeprazol
- [ ] Laxtantes
- [ ] Ibuprofeno
- [ ] Naproxeno
- [ ] Metamizol
- [ ] Ketorolaco
- [ ] No consumo ninguno de esos medicamentos
- [ ] Otro: ______________________

34. Si respondiste que si, menciona que tan frecuente, por favor *

Marca solo un óvalo.

- Todos días días, más de una vez al día
- Todos días días, una vez al día
- 4 a 6 veces por semana
- 2 a 3 veces por semana
- 1 vez por semana
- 1 vez cada 15 días
- 1 vez al mes
- 1 vez cada 2 meses
- Cada 6 meses
- Casi nunca
- No consumo ninguno de esos medicamentos
- Otro: ______________________

35. ¿En los últimos tres meses has consumido algún tipo de antibiótico? *

Marca solo un óvalo.

- Sí
- No

36. Si respondiste que si, menciona cuál por favor *

Selecciona todos los que correspondan.

- [ ] Ampicilina
- [ ] Cefalexina
- [ ] Ciprofloxacino
- [ ] Claritromicina
- [ ] Ceftriaxona
- [ ] No he consumido ninguno de esos medicamentos en el último año
- [ ] Otro: ______________________

37. Si respondiste que si, menciona hace cuánto tiempo consumiste el último ciclo *

Marca solo un óvalo.

- Hace 1 semana
- Hace 1 mes
- Hace 2 meses
- Hace 3 meses
- Hace 4 meses
- Hace 6 meses
- Hace 1 año
- Hace más de 1 año
- No he consumido ninguno de esos medicamentos en el último año
- Otro: ________________

38. Si tu sexo es femenino ¿Actualmente estás embarazada? (Si tu sexo es masculino, coloca no) *

Marca solo un óvalo.

- Sí
- No

39. Si tu sexo es femenino ¿Actualmente estás amamantando? (Si tu sexo es masculino, coloca no) *

Marca solo un óvalo.

- Sí
- No

40. ¿Sabes si eres alérgico o intolerante a algún alimento? Por ejemplo, alérgico al cacahuate o intolerante a la lactosa *

Marca solo un óvalo.

- Sí
- No
- No lo sé

41. Si respondiste que si, menciona a que alimento(s) por favor *

Selecciona todos los que correspondan.

- [ ] Lácteos (Lactosa)
- [ ] Mariscos
- [ ] Gluten
- [ ] Fresas
- [ ] Cacahuates
- [ ] Nueces
- [ ] No soy alérgico(a) a ningún alimento
- [ ] Otro: ______________________

Historia dietética y de actividad física

En este caso, queremos saber un poco más sobre tu alimentación y la actividad física que realizas. Recuerda que no serás juzgado por tus respuestas, y todas son confidenciales.

42. ¿Actualmente estás siguiendo una dieta específica? *

Marca solo un óvalo.

- ( ) Sí
- ( ) No

43. Si respondiste que si, menciona cuál por favor

Selecciona todos los que correspondan.

- [ ] Para bajar de peso
- [ ] Para subir de peso
- [ ] Cetogénica
- [ ] Vegetariana (ovo-lacto, ovo o lacto)
- [ ] Vegana (no consumes ningún alimento de origen animal, ni miel)
- [ ] Ayuno intermitente
- [ ] Mediterránea
- [ ] DASH o dieta para detener la hipertensión
- [ ] No sigo ninguna dieta en específico
- [ ] Otro: ______________________

44. ¿Desde hace cuánto tiempo que sigues esa dieta? *

Marca solo un óvalo.

- 1 semana
- 2 semanas
- 1 mes
- 2 meses
- 3 meses
- 6 meses
- 1 año
- 3 años
- 5 años
- Toda la vida la he seguido
- No sigo ningún tipo de dieta en particular
- Otro: ____________________

45. ¿Quién cocina los alimentos que consumes? *

Selecciona todos los que correspondan.

- [ ] Yo
- [ ] Algún familiar (mamá, papá, tía, tío, abuela, abuelo)
- [ ] Cocina económica
- [ ] Restaurantes
- [ ] Me la regalan
- [ ] Ayudante del hogar o cocinera
- [ ] Otro: ____________________

46. Del 1 al 5 ¿Sueles experimentar ansiedad por comer alimentos en específicos? *

Marca solo un óvalo.

Nada de ansiedad

- 1
- 2
- 3
- 4
- 5

Mucha ansiedad

47. Si experimentas ansiedad, menciona por favor, que alimentos la generan *

48. Durante el último año ¿con qué frecuencia has consumido los siguientes grupos de alimentos? Los alimentos entre paréntesis son ejemplos. Incluye por favor, todos los alimentos que consumas. Si comes varios dentro de un grupo, sumálos por favor. Por ejemplo, si comes huevo a la mexicana el lunes, y el domingo pozole, consumes alimentos y platillos mexicanos 2 veces por semana *

Marca solo un óvalo por fila.

	Nunca o casi nunca	1 a 3 veces al mes	1 vez a la semana	2 a 4 veces a la semana	5 a 6 veces a la semana	1 vez al día	2 a 3 veces al día	4 a vec al c
**Alimentos y platillos mexicanos (chilaquiles, huevo a la mexicana, menudo, quesadillas, tacos, sopes, tortas, tamles, flautas, tostadas, arroz con leche, capitorada, tejuino)**	◯	◯	◯	◯	◯	◯	◯	◯
**Frutas (ciruela criolla, capulín, nance, papaya, guayaba, tuna, pitaya, guanábana, piña, manzana)**	◯	◯	◯	◯	◯	◯	◯	◯
**Vegetales (jitomate, calabacita, calabaza, cebolla, tomate verde, champiñones, huitlacoche, chayote, jícama)**	◯	◯	◯	◯	◯	◯	◯	◯
**Cereales integrales y tubérculos (tortilla de maíz, amaranto, camote, arroz, pasta, pan integral)**	◯	◯	◯	◯	◯	◯	◯	◯
**Leguminosas (frijoles,**	◯	◯	◯	◯	◯	◯	◯	◯

lentejas, garbanzos)

49. Durante el último año ¿con qué frecuencia has consumido los siguientes grupos de alimentos? Los alimentos entre paréntesis son ejemplos. Incluye por favor, todos los alimentos que consumas. Si comes varios dentro de un grupo, sumálos por favor. Por ejemplo, si comes huevo a la mexicana el lunes, y el domingo pozole, consumes alimentos y platillos mexicanos 2 veces por semana *

Marca solo un óvalo por fila.

	Nunca o casi nunca	1 a 3 veces al mes	1 vez a la semana	2 a 4 veces a la semana	5 a 6 veces a la semana	1 vez al día	2 a 3 veces al día	4 a 6 veces al día
**Semillas y grasas saludables (aguacate, aceite de oliva, chía, cacahuate, semilla de calabaza)**	⬭	⬭	⬭	⬭	⬭	⬭	⬭	⬭
**Lácteos (leche, yogurt, requesón, queso fresco, panela**	⬭	⬭	⬭	⬭	⬭	⬭	⬭	⬭
**Huevos (de gallina entero, claras de huevo)**	⬭	⬭	⬭	⬭	⬭	⬭	⬭	⬭
**Pescados y mariscos (tilapia, leguado, pulpo, langostino de río)**	⬭	⬭	⬭	⬭	⬭	⬭	⬭	⬭
**Pollo (en filete, en piezas)**	⬭	⬭	⬭	⬭	⬭	⬭	⬭	⬭

50. Durante el último año ¿con qué frecuencia has consumido los siguientes grupos de alimentos? Los alimentos entre paréntesis son ejemplos. Incluye por favor, todos los alimentos que consumas. Si comes varios dentro de un grupo, sumálos por favor. Por ejemplo, si comes huevo a la mexicana el lunes, y el domingo pozole, consumes alimentos y platillos mexicanos 2 veces por semana *

Marca solo un óvalo por fila.

	Nunca o casi nunca	1 a 3 veces al mes	1 vez a la semana	2 a 4 veces a la semana	5 a 6 veces a la semana	1 vez al día	2 a 3 veces al día	4 a [illegible] vece[illegible] al dí[illegible]
**Carnes rojas y procesadas (carne de res, cerdo, salchichas, jamón)**	⬭	⬭	⬭	⬭	⬭	⬭	⬭	⬭
**Alimentos ultra-procesados (refresco, galletas, cereal de caja, sopas instantáneas, pastelitos, papas fritas, botanas fritas)**	⬭	⬭	⬭	⬭	⬭	⬭	⬭	⬭
**Azúcar añadido y alimentos altos en grasas trans y saturadas (azúcar de mesa, agua fresca endulzada, miel, mermeladas, manteca vegetal, margarina, mantequilla)**	⬭	⬭	⬭	⬭	⬭	⬭	⬭	⬭
**Bebidas alcoholicas (cerveza, tequila, vino tinto, pulque)**	⬭	⬭	⬭	⬭	⬭	⬭	⬭	⬭
**Insectos (grillos, gusanos)**	⬭	⬭	⬭	⬭	⬭	⬭	⬭	⬭
**Sal de mesa**	⬭	⬭	⬭	⬭	⬭	⬭	⬭	⬭

51. En promedio, ¿qué porción de alimentos, platillos o bebidas sueles consumir cada vez que los consumes POR DÍA? Por ejemplo, si comes 3 tortillas en el desayuno, comida y cena, comes 9 piezas por día *

Marca solo un óvalo por fila.

	0	1/4 pieza, taza, o porción	1/2 pieza, taza, o porción	1 pieza, taza, cucharada o porción	2 piezas, tazas, cucharadas o porciones	3 piezas, tazas, cucharadas o porciones	4 p[illegible] t[illegible] cuc[illegible] por[illegible]
**Tacos, sopes, enchiladas, flautas**	⬭	⬭	⬭	⬭	⬭	⬭	⬭
**Tamales, tortas, tostadas**	⬭	⬭	⬭	⬭	⬭	⬭	⬭
**Pozole, menudo, mole**	⬭	⬭	⬭	⬭	⬭	⬭	⬭
**Frutas en piezas (manzana, naranja)**	⬭	⬭	⬭	⬭	⬭	⬭	⬭
**Fruta picada o piezas pequeñas (melón, papaya, uvas)**	⬭	⬭	⬭	⬭	⬭	⬭	⬭

52. En promedio, ¿qué porción de alimentos, platillos o bebidas sueles consumir cada vez que los consumes POR DÍA? Por ejemplo, si comes 3 tortillas en el desayuno, comida y cena, comes 9 piezas por día *

Marca solo un óvalo por fila.

	0	1/4 pieza, taza, o porción	1/2 pieza, taza, o porción	1 pieza, taza, cucharada o porción	2 piezas, tazas, cucharadas o porciones	3 piezas, tazas, cucharadas o porciones	[illegible]
**Verduras en piezas (jitomate, pepino)**							
**Verduras en tazas (lechuga, espinacas)**							
**Cereales en piezas (tortillas de maíz, pan de caja)**							
**Cereales en tazas (arroz, pasta)**							
**Leguminosas (frijoles, lentejas)**							

53. En promedio, ¿qué porción de alimentos, platillos o bebidas sueles consumir cada vez que los consumes POR DÍA? Por ejemplo, si comes 3 tortillas en el desayuno, comida y cena, comes 9 piezas por día *

Marca solo un óvalo por fila.

	0	1/4 pieza, taza, o porción	1/2 pieza, taza, o porción	1 pieza, taza, cucharada o porción	2 piezas, tazas, cucharadas o porciones	3 piezas, tazas, cucharadas o porciones	4 p[illegible] t[illegible] cuch[illegible] por[illegible]
**Leche y yourt**							
**Quesos**							
**Nueces y cacahuates**							
**Semillas de chía y calabaza**							
**Huevo y claras de huevo**							

54. En promedio, ¿qué porción de alimentos, platillos o bebidas sueles consumir cada vez que los consumes POR DÍA? Por ejemplo, si comes 3 tortillas en el desayuno, comida y cena, comes 9 piezas por día *

Marca solo un óvalo por fila.

	0	1/4 pieza, taza, o porción	1/2 pieza, taza, o porción	1 pieza, taza, cucharada o porción	2 piezas, tazas, cucharadas o porciones	3 piezas, tazas, cucharadas o porciones	4 [illegible] cuc[illegible] po[illegible]
**Pescado (porción de 80 g del tamaño palma de la mano)**	⬭	⬭	⬭	⬭	⬭	⬭	
**Mariscos (camarones, pulpo)**	⬭	⬭	⬭	⬭	⬭	⬭	
**Pollo (pieza o porción de 80 g del tamaño palma de la mano)**	⬭	⬭	⬭	⬭	⬭	⬭	
**Carne de res o cerdo (porción de 80 g del tamaño palma de la mano)**	⬭	⬭	⬭	⬭	⬭	⬭	
**Pan dulce (concha)**	⬭	⬭	⬭	⬭	⬭	⬭	

55. En promedio, ¿qué porción de alimentos, platillos o bebidas sueles consumir cada vez que los consumes POR DÍA? Por ejemplo, si comes 3 tortillas en el desayuno, comida y cena, comes 9 piezas por día *

Marca solo un óvalo por fila.

	0	1/4 pieza, taza, o porción	1/2 pieza, taza, o porción	1 pieza, taza, cucharada o porción	2 piezas, tazas, cucharadas o porciones	3 piezas, tazas, cucharadas o porciones	4 [illegible] cu[illegible] p[illegible]
**Azúcar, mermeladas, dulces**							
**Refrescos, jugos de fruta**							
**Bebidas alcohólicas**							
**Agua natural**							
**Sal de mesa**							

56. ¿Realizas actividad física? Por ejemplo, caminar, trotar, correr, aeróbics, zumba, baile, ciclismo, natación, crossfit, pesas, etc. *

Marca solo un óvalo.

- Sí
- No

57. ¿Cuántos días a la semana realizas actividad física? (por ejemplo, caminar, correr, natación, pesas) *

Marca solo un óvalo.

- 0
- 1
- 2
- 3
- 4
- 5
- 6
- 7

58. ¿Qué tipo de actividad física practicas? *

Selecciona todos los que correspondan.

- [ ] Caminar
- [ ] Trotar
- [ ] Correr
- [ ] Aeróbics, zumba o baile
- [ ] Ciclismo
- [ ] Natación
- [ ] Crossfit
- [ ] Pesas
- [ ] Multifuncional
- [ ] Ejercicios focalizados (escribir cuales, por ejemplo abdominales, sentadillas, lagartijas)
- [ ] Calistenia
- [ ] No realizo acitividad física
- [ ] Otro: ____________________

59. ¿Cuántos minutos de actividad física realizas por día, cada vez que la realizas? *

Marca solo un óvalo.

- ( ) 0
- ( ) 10
- ( ) 20
- ( ) 30
- ( ) 40
- ( ) 50
- ( ) 60
- ( ) 90
- ( ) 120
- ( ) 150
- ( ) 180
- ( ) 210
- ( ) 240 o más
- ( ) Otro: ____________________

60. ¿A qué intensidad la realizas? *

Marca solo un óvalo.

- ( ) Leve (no agita la respiración)
- ( ) Moderada (agita la respiración)
- ( ) Intensa (agita mucho la respiración)
- ( ) Otro: ____________________

Detalles del estudio

Para poder conocer tus niveles de glucosa, colesterol, triglicéridos, microbiota intestinal y así analizar tu salud, es necesario que nos proporciones algunas muestras biológicas, respondas a algunos cuestionarios y realicemos algunas evaluaciones nutricionales, por ello, queremos preguntarte lo siguiente.

61. En caso de contar con las características que buscamos en nuestro estudio ¿Aceptarías participar en él? *

Marca solo un óvalo.

- Si
- No

62. Finalmente, escribe por favor tu número de celular para contactarte si eres selecionado para participar en este estudio. Recuerda que esa información es y siempre será confidencial *

____

63. También compartenos por favor tu correo electrónico *

____

Este contenido no ha sido creado ni aprobado por Google.

Google Formularios
