# Supplementary material for: Sustainable-psycho-nutritional intervention programme for a sustainable diet (the ‘NutriSOS’ study) and its effects on eating behaviour, diet quality, nutritional status, physical activity, metabolic biomarkers, gut microbiota and water and carbon footprints in Mexican population: study protocol of an mHealth randomised controlled trial
Source: Br J Nutr. 2023 Mar 30;130(10):1823–38. doi: 10.1017/S0007114523000843 (PMC10587386; doi:10.1017/S0007114523000843)
Supplement: Supplementary file 1 [file S0007114523000843sup.zip › S0007114523000843sup002.zip!/Online Supplementary material 6. Sustaiable nutrition questionnaire English.pdf]

NutriSó Sustainable Nutritional Knowledge Initial Questionnaire

Hello again :)

One of the objectives of the NutriSó study is to increase your knowledge of nutrition and food sustainability. For this, we will prepare a series of videos and webinars that will help you learn more about these topics, but first, we want to know a little about your current knowledge, in order to prepare material suitable for your requirements.

Therefore, we ask that you answer the following questions as honestly as possible. This is not an exam, but rather, a survey. So don't worry, and please answer without consulting sources of information.

Remember that all your data is private and is protected by the Federal Law on the Protection of Personal Data Held by Private Parties.

*Obligatorio

1. Correo *

2. Last name *

3. Mother's last name *

4. Name *

5. If you remember it, put your participant number please

____

Sustainable diets

Below are some questions about nutrition and sustainability. Please answer without consulting any source of information. Remember that you will not be judged at any time by your answers and this is not an exam.

6. A sustainable diet is... *

Marca solo un óvalo.

- ( ) A diet that supports many families at the same time
- ( ) A diet to lose weight quickly and keep it off
- ( ) They are dietary patterns that promote health and nutrition, have a low environmental impact, are accessible, affordable, and culturally acceptable.
- ( ) They are dietary patterns that promote a low environmental impact even if aspects such as nutrition and culture have to be ignored
- ( ) I am not sure

7. To achieve a sustainable diet, is it recommended that people consume, relative to their current intake... more, the same amount or less of the following foods? (please check one box per food) *

Marca solo un óvalo por fila.

	More	The same amount	Less	I am not sure
**Mexican foods and dishes with little or no meat, and based on corn and legumes such as beans**	⬭	⬭	⬭	⬭
**Fruits**	⬭	⬭	⬭	⬭
**Vegetables**	⬭	⬭	⬭	⬭
**Whole grains**	⬭	⬭	⬭	⬭
**Legumes**	⬭	⬭	⬭	⬭
**Dairy (milk, cheese and yogurt)**	⬭	⬭	⬭	⬭
**Seeds and healthy fats**	⬭	⬭	⬭	⬭
**Egg**	⬭	⬭	⬭	⬭
**Fish and shellfish**	⬭	⬭	⬭	⬭
**Chicken**	⬭	⬭	⬭	⬭
**Red and processed meats**	⬭	⬭	⬭	⬭
**Ultra-processed foods (soft drinks, cookies)**	⬭	⬭	⬭	⬭
**Trans and saturated fats and oils**	⬭	⬭	⬭	⬭
	⬭	⬭	⬭	⬭

Added sugar in ready-made foods	◯	◯	◯	◯
Salt added to already cooked food	◯	◯	◯	◯
Natural water	◯	◯	◯	◯
Alcoholic drinks	◯	◯	◯	◯

8. The water footprint of food is... *

Marca solo un óvalo.

- ◯ The water that is stored in the subsoil and is never used by humans
- ◯ Fresh water needed to produce food
- ◯ The salt water that is in the oceans
- ◯ I am not sure

9. Which of the following foods requires more water during production per kilogram of product? *

Marca solo un óvalo.

- ◯ Sugary or fatty cereals like cookies and cakes
- ◯ Beef
- ◯ Chicken egg
- ◯ Pork Meat
- ◯ Avocado
- ◯ I am not sure

10. The carbon footprint of a food is... *

Marca solo un óvalo.

- The amount of carbon generated by burning garbage or food scraps
- The amount of greenhouse gases emitted by the production of a food
- Smoke generated during fires
- I am not sure

11. Which of the following foods emits the most greenhouse gases during production per kilogram of product? *

Marca solo un óvalo.

- Vegetable oils
- Beef
- Chicken egg
- Pork Meat
- Corn tortilla
- I am not sure

12. Do you think the following foods are low or high in fiber? *

Marca solo un óvalo por fila.

	Low fiber content	High fiber content	I am not sure
**Oatmeal**			
**Amaranth**			
**Papaya**			
**White rice**			
**Egg**			
**Potato or sweet potato with peel**			
**Pasta**			

13. Do you think the following foods are a low or high source of protein? *

Marca solo un óvalo por fila.

	Low source of protein	High source of protein	I am not sure
**Chicken**			
**Fresh cheese or panela**			
**Fruits**			
**Cooked beans**			
**Butter**			
**Nuts**			

14. If you wanted to buy a sustainable food option in a restaurant, which option would you choose? *

Marca solo un óvalo.

- Beef tacos with onion and cilantro with fresh sweetened lemon water
- Sopitos of minced beef with carrot and potato with a sugar-free soft drink
- Enfrijoladas with fresh cheese and lettuce with jamaica water without sugar
- I am not sure

15. Which breakfast do you think is more sustainable? *

Marca solo un óvalo.

- Mexican style eggs with tomato, onion and nopales, with corn tortilla, beans from the pot and natural water
- Panela quesadillas with little beef and orange juice
- Red chilaquiles with beef and a boiled egg with green spinach juice and asparagus
- I am not sure

16. Which dessert option do you think is more sustainable? *

Marca solo un óvalo.

- Cooked rice with skimmed milk with piloncillo and cinnamon
- Chocolate cake with whipped cream
- Chocolate ice cream packed in a recyclable container
- I am not sure

Physical activity

Here are some questions about physical activity. Answer without consulting sources of information please. Remember that this is not an exam.

17. What is the minimum number of minutes of physical activity that should be performed daily to be healthy in the context of sustainable diets? *

Marca solo un óvalo.

- 5 minutes
- 10 minutes
- 20 minutes
- 30 minutes
- 40 minutes
- 50 minutes
- 60 minutes
- More than 60 minutes
- I am not sure

18. Regarding physical activity, it is true that... *

Marca solo un óvalo.

- It is recommended only to perform cardiovascular exercises such as walking or running
- It is advisable to combine cardiovascular exercises with strength exercises
- It is better not to do very vigorous physical activity and only eat healthy
- I am not sure

19. Using the bicycle or walking as a means of transport has a positive effect on the environment *

Marca solo un óvalo.

- In agreement
- In disagreement
- I am not sure

20. Point out the health benefits of physical activity *

Marca solo un óvalo.

- Lowers blood pressure and regulates blood glucose levels
- Causes you to lose body fat even if you consume more calories than you expend
- I am not sure

21. One way to save money on transportation is *

Marca solo un óvalo.

- Use fuel-efficient gasoline
- Walk and bike whenever possible
- Save to buy an electric car
- I am not sure

22. An appropriate way to perform physical activity without noticing it is... *

Marca solo un óvalo.

- Run for 20 minutes close to home
- Do housework and grow my own food
- Enroll in aerobics classes
- I am not sure

23. The best exercise is... *

Marca solo un óvalo.

- CrossFit or multifunctional exercises
- Zumba or Latin rhythms
- Walk or jog
- One that combines cardiovascular and strength exercises and enjoys doing it
- I am not sure

24. In order for exercise to help us prevent chronic disease, it must increase our heart rate and breathing *

Marca solo un óvalo.

- In agreement
- In disagreement
- I am not sure

Mexican food and dishes

The traditional Mexican diet is delicious, but we want to know what you think about it from a sustainability perspective. Please answer as honestly as possible.

25. Eating corn tortillas every day causes weight gain *

Marca solo un óvalo.

- In agreement
- In disagreement
- I am not sure

26. Fiber can decrease the chances of gaining weight *

Marca solo un óvalo.

- In agreement
- In disagreement
- I am not sure

27. Select the option that you consider to be more sustainable *

Marca solo un óvalo.

- Pozole of corn with chicken and lots of lettuce
- Eggplant and quinoa pozole
- Pozole of corn with pork and lots of lettuce
- It is not recommended to consume any version of pozole
- I am not sure

28. Corn has a water and carbon footprint... *

Marca solo un óvalo.

- Greater than meat
- Same as meat
- Less than meat
- I am not sure

29. They are Mexican cereals *

Marca solo un óvalo.

- Oatmeal and quinoa
- Corn and amaranth
- Corn and wheat
- I am not sure

30. The traditional Mexican diet is too expensive to consume every day *

Marca solo un óvalo.

- In agreement
- In disagreement
- I am not sure

Fruits and vegetables

Surely you have heard that fruits and vegetables are healthy, right? We want to know your opinion on some aspects related

31. How many servings of fruits and vegetables is recommended to consume, minimally, per day to achieve a sustainable diet? (A serving could be, for example, an apple or a cup of papaya) *

Marca solo un óvalo.

- 2
- 3
- 4
- 5 or more
- I am not sure

32. It is recommended that vegetables be consumed... *

Marca solo un óvalo.

- After eating protein such as meats
- Before starting to eat a dish, for example, before meat and rice
- In the middle of the meal, for example, start with rice, then vegetables and finally meat
- I am not sure

33. Which of the following salad combinations would provide the highest variety of vitamins and antioxidants? *

Marca solo un óvalo.

- Lettuce, spinach and cucumber
- Nopales, tomato, onion and carrot
- Red bell pepper, tomato and cherry tomato
- I am not sure

34. Which of these diseases is associated with low fiber intake? *

Marca solo un óvalo.

- Intestinal diseases
- Anemia
- Dental cavities
- I am not sure

35. The more fiber consumed, the diet generally has an environmental impact... *

Marca solo un óvalo.

- Higher
- Lower
- I am not sure

36. Eating fruits and vegetables every day is very expensive *

Marca solo un óvalo.

- In agreement
- In disagreement
- I am not sure

37. Select the fruits and vegetables that are considered Mexican *

Marca solo un óvalo.

- Nopal, tomato, asparagus, eggplant, watermelon and mango
- Nopal, tomato, chili, pumpkin, pineapple and papaya
- Carrot, mushrooms, broccoli, apple and nisperos
- I am not sure

Whole grains and tubers

We want to know your opinion about whole grains. Please answer as honestly as possible and without consulting sources of information.

38. How much of your plate is recommended to be whole grains or tubers, such as corn tortillas or sweet potatoes? *

Marca solo un óvalo.

- A quarter of the plate
- A third of the plate
- Half of the plate
- I am not sure

39. The corn tortilla... *

Marca solo un óvalo.

- Provides energy, complex carbohydrates, fiber and B vitamins
- Provides simple carbohydrates and vitamin b12
- Provides high amounts of protein and creatine
- It is an important source of healthy fats such as omega 3
- I am not sure

40. Potato and sweet potato are... *

Marca solo un óvalo.

- Low energy vegetables
- Tubers that provide complex carbohydrates
- Fruits that provide sugar
- Healthy fats that provide omega 3
- I am not sure

41. To achieve a sustainable diet, cereals and tubers must be present in our diet... *

Marca solo un óvalo.

- Every day
- 3 to 4 times a week
- 1 to 2 times a week
- Never
- I am not sure

42. Eating whole grains is very expensive *

Marca solo un óvalo.

- In agreement
- In disagreement
- I am not sure

Legumes

Now we want to know a little more about what you think about legumes. Answer honestly and remember that this is not a test.

43. The legumes provide *

Marca solo un óvalo.

- Vegetable protein and complex carbohydrates
- Saturated fats
- Sugars and protein of high biological value
- Vitamin b12 and fiber
- I am not sure

44. Of legumes it is true that... *

Marca solo un óvalo.

- They can replace meats when it comes to protein
- They can never replace meats for protein
- I am not sure

45. What benefits do legumes bring to health? *

Marca solo un óvalo.

- They prevent the development of type 2 diabetes and improve the composition of the intestinal microbiota (bacteria in the intestine)
- They prevent the development of cancer
- They make you lose weight without exercising
- I am not sure

46. At an environmental level, legumes... *

Marca solo un óvalo.

- They have a water and carbon footprint similar to that of red meat
- They have a lower carbon and water footprint than red meat
- Its environmental impact is higher than that of red meat
- I am not sure

47. Replacing meats with legumes is a way to save money *

Marca solo un óvalo.

- In agreement
- In disagreement
- I am not sure

48. They are examples of Mexican legumes, according to the milpa diet *

Marca solo un óvalo.

- Beans, lentils and broad beans
- Broad beans, chickpeas and beans
- Piñon, peanut and chia
- I am not sure

Dairy products

Here are some questions about dairy consumption. Please answer as honestly as possible.

49. When consuming dairy in a sustainable diet, it is recommended to consume... *

Marca solo un óvalo.

- Whole milk (with fat)
- Low-fat dairy (skim and part-skim)
- A mix of full-fat and non-fat dairy
- No dairy, as it is better to avoid them
- I am not sure

50. The amount of calcium in a glass of skim or light milk, compared to a glass of whole milk is: *

Marca solo un óvalo.

- Almost the same
- Much higher
- Much lower
- I am not sure

51. If you wanted to buy a yogurt in the market, which option would have less sugar? *

Marca solo un óvalo.

- Yogurt 0% fat strawberry flavor
- Natural yogurt
- Creamy fruit yogurt
- I am not sure

52. Only by not eating dairy can I achieve a sustainable diet *

Marca solo un óvalo.

- In agreement
- In disagreement
- I am not sure

53. In order to consume dairy, I must choose the most expensive ones to guarantee the highest possible quality *

Marca solo un óvalo.

- In agreement
- In disagreement
- I am not sure

54. They are examples of sustainable cheeses for Mexico *

Marca solo un óvalo.

- Mozzarella and Parmesan cheese
- Panela cheese and cottage cheese
- Gouda and American cheese
- I am not sure

Seeds and healthy fats

Here are some questions about seeds and healthy fats in our diet. Please answer without consulting sources of information.

55. Which of these types of fats do experts recommend people should eat less of to achieve a sustainable diet? (check one box per food) *

Marca solo un óvalo por fila.

	Eat less	Eat the same amount	I am not sure
**Unsaturated fats**			
**Trans fat**			
**Saturated fats**			

56. What is the main type of fat present in each of these foods? *

Marca solo un óvalo por fila.

	Polyunsaturated fats	Monounsaturated fats	Saturated fats	Cholesterol	I am not sure
**Extra virgin olive oil**					
**Butter**					
**Sunflower oil**					
**Eggs**					

57. They are sustainable seeds for the Mexican diet *

Marca solo un óvalo.

- Unsalted peanuts, chia and pumpkin seeds
- Pecan nut, almonds and peanuts
- Caramelized peanuts, chia and cashew nuts
- I am not sure

58. Avocado provides the following health benefits *

Marca solo un óvalo.

- Improves the composition of the gut microbiota and has healthy fats that prevent heart disease
- It is very low in calories and two pieces a day help reduce the percentage of body fat
- It is high in protein, so it strengthens the muscles
- I am not sure

59. From a sustainability perspective, can olive oil be included in the traditional Mexican diet? *

Marca solo un óvalo.

- Yes
- No
- Depends on the frequency and quantity of consumption
- I am not sure

60. One way to reduce the environmental impact of avocados from southern Jalisco is... *

Marca solo un óvalo.

- Consume only those that are given to me, if it is cheap or plant my own avocados
- Buy only those organic avocados of export quality
- I am not sure

61. With regard to price, nutrition, environment and culture, healthy seeds and fats can be sustainable options if... *

Marca solo un óvalo.

- Are consumed in the appropriate frequency and quantity
- They are consumed only on a monthly basis
- They cannot be sustainable options due to their high fat content
- I am not sure

Eggs

Now, some questions about chicken eggs are presented. Please answer as honestly as possible.

62. Egg yolk raises blood cholesterol levels *

Marca solo un óvalo.

- True
- False
- I am not sure

63. The egg is a high source of protein of high biological value *

Marca solo un óvalo.

- In agreement
- In disagreement
- I am not sure

64. The egg is a cheap source of protein *

Marca solo un óvalo.

- In agreement
- In disagreement
- I am not sure

65. A sustainable recipe with egg is.... *

Marca solo un óvalo.

- Egg in red sauce with tomato, beans from the pot and corn tortilla
- Mexican style egg with bacon and olive oil
- Eggs in omelette with asparagus
- I am not sure

66. How many eggs does the Diet for Planetary Health recommend eating at most per week? *

Marca solo un óvalo.

- 3 pieces
- 4 pieces
- 5 pieces
- 8 pieces
- It is recommended to avoid eating eggs

67. Ranch or locally produced eggs are generally... *

Marca solo un óvalo.

- Dirtier, so its consumption is not recommended
- A more sustainable option than those produced at an industrial level
- Less healthy and dangerous for health
- I am not sure

Fish and shellfish

On this occasion, some questions about fish and shellfish are presented. Please answer as honestly as possible.

68. How many times a week is it recommended to consume fish produced in Mexico, such as mojarra or tilapia, as part of a sustainable diet? *

Marca solo un óvalo.

- 1 to 2 times a week
- 3 to 4 times a week
- Every day
- I am not sure

69. Fish provides... *

Marca solo un óvalo.

- Omega 3 and high biological value protein
- Fiber and monounsaturated fats
- Vegetable protein and carbohydrates
- I am not sure

70. According to the Diet for Planetary Health, eating more than 196 grams of fish per week has a high environmental impact *

Marca solo un óvalo.

- In agreement
- In disagreement
- I am not sure

71. Only fish that is expensive provides enough nutrients to my body *

Marca solo un óvalo.

- In agreement
- In disagreement
- I am not sure

72. Generally, the further away the fish and/or shellfish come from, the higher their environmental impact will be *

Marca solo un óvalo.

- In agreement
- In disagreement
- I am not sure

Chicken

Now, some questions about chicken are presented, please answer without consulting sources of information.

73. Chicken has a lower carbon and water footprint than beef and pork *

Marca solo un óvalo.

- In agreement
- In disagreement
- I am not sure

74. Chicken provides protein of high biological value *

Marca solo un óvalo.

- In agreement
- In disagreement
- I am not sure

75. In the context of sustainable diets it is recommended... *

Marca solo un óvalo.

- Eat chicken maximum 1 to 2 times per week
- Eat chicken maximum 3 to 4 times a week
- Eat chicken more than 4 times a week
- Do not eat chicken

76. Chicken can be a sustainable option if... *

Marca solo un óvalo.

- We consume it to replace beef at all times
- We consume it with the appropriate frequency and quantity
- Chicken cannot be sustainable
- I am not sure

77. By substituting legumes for chicken in dishes, you could save up to 83% of the money you spend on chicken *

Marca solo un óvalo.

- In agreement
- In disagreement
- I am not sure

Red and processed meats

Now there are questions about red and processed meats. Remember that this is not an exam, but a survey, so we ask you to answer honestly and without consulting sources of information.

78. Beef is the food that uses the largest amount of water during its production *

Marca solo un óvalo.

- In agreement
- In disagreement
- I am not sure

79. Eating beef frequently increases the chances of getting cancer *

Marca solo un óvalo.

- In agreement
- In disagreement
- I am not sure

80. To maintain a healthy weight, people should eat a diet rich in protein *

Marca solo un óvalo.

- In agreement
- In disagreement
- I am not sure

81. Please select the foods that have the highest carbon footprint *

Marca solo un óvalo.

- Pork and bacon
- Beef and lamb
- Fish and shellfish
- Sausages and pork ham
- I am not sure

82. How many times a week is it recommended to eat beef? *

Marca solo un óvalo.

- 1 time per week or less
- 2 times per week
- 3 to 4 times a week
- I am not sure

83. By substituting legumes for beef, you can save up to *

Marca solo un óvalo.

- 20% of what I would spend if I eat meat
- Up to 50% of what I would spend if I eat meat
- Up to 90% of what I would spend if I eat meat
- I am not sure

Ultra-processed foods

Now, there are some questions about ultra-processed foods, that is, highly processed. Please answer honestly and without consulting sources of information.

84. Compared to unprocessed or minimally processed foods, ultra-processed foods are: *

Marca solo un óvalo.

- Higher in calories
- Higher in fiber
- Lower in salt
- I am not sure

85. Eating ultra-processed foods on a regular basis has been associated with... *

Marca solo un óvalo.

- Prevention of type 2 diabetes and heart disease
- Development of type 2 diabetes and heart disease
- Its consumption has not been associated with the development or prevention of any disease
- I am not sure

86. The consumption of ultra-processed foods alters the intestinal microbiota, which can decrease the body's immune response *

Marca solo un óvalo.

- In agreement
- In disagreement
- I am not sure

87. Large ultra-processed food factories generate high impacts on the environment *

Marca solo un óvalo.

- In agreement
- In disagreement
- I am not sure

88. In the context of sustainable diets, the consumption of ultra-processed foods *

Marca solo un óvalo.

- It is discouraged
- It is recommended to eat them once a week
- It is recommended to eat them 2 to 3 times a week
- I am not sure

89. Eating ultra-processed foods every day is *

Marca solo un óvalo.

- Cheaper than eating healthy food
- More expensive than eating healthy food
- Just as expensive or cheap as eating healthy food
- I am not sure

90. Ultra-processed foods can be part of a sustainable traditional Mexican diet *

Marca solo un óvalo.

- In agreement
- In disagreement
- I am not sure

91. Generally, the longer the list of ingredients in a product, the higher its environmental impact *

Marca solo un óvalo.

- In agreement
- In disagreement
- I am not sure

Sugar, salt, trans fats, saturated and alcoholic beverages

Finally, questions are presented about the consumption of sugar, salt, trans fats, saturated fats and alcoholic beverages.

92. Do you think the following foods are high or low in sugar? *

Marca solo un óvalo por fila.

	Low sugar content	High sugar content	I am not sure
**Light or sugar-free soft drinks**	◯	◯	◯
**Natural yogurt**	◯	◯	◯
**Ice cream**	◯	◯	◯
**Ketchup**	◯	◯	◯
**Melon**	◯	◯	◯

93. Which of these diseases is related to the amount of sugar people consume? *

Marca solo un óvalo.

- ◯ Arterial hypertension (high blood pressure)
- ◯ Dental caries and type 2 diabetes
- ◯ Anemia
- ◯ I am not sure

94. Which of these options do experts recommend to prevent type 2 diabetes? *

Marca solo un óvalo.

- ◯ Eat less refined foods
- ◯ Drink more fruit juices
- ◯ Eat more red and processed meats
- ◯ I am not sure

95. Which of these foods is classified with a high glycemic index? *

Marca solo un óvalo.

- Whole grains
- White bread
- Fruits and vegetables
- I am not sure

96. From an environmental perspective, the water and carbon footprint of sugars is... *

Marca solo un óvalo.

- Low compared to foods of animal origin
- High compared to foods of animal origin
- Same as food of animal origin
- I am not sure

97. Do you think the following foods are low or high in salt? *

Marca solo un óvalo por fila.

	Low salt content	High salt content	I am not sure
**Box cereals for breakfast**			
**Frozen vegetables**			
**Box bread**			
**Canned soups**			
**Potato or corn snacks**			

98. A healthy way to flavor food without adding salt is to add *

Marca solo un óvalo.

- Coconut milk
- Herbs like pepper, oregano, bay leaves
- Soy sauce
- I am not sure

99. Which of these diseases is related to the amount of salt (or sodium) that people consume? *

Marca solo un óvalo.

- Hypothyroidism
- Arterial hypertension (high blood pressure)
- Type 2 diabetes
- I am not sure

100. High-sodium foods are generally highly processed, so their environmental impact tends to be high *

Marca solo un óvalo.

- In agreement
- In disagreement
- I am not sure

101. Which of the following foods do you think are higher in trans fat? *

Marca solo un óvalo.

- Cookies and cupcakes
- Fish
- Canola oil
- Eggs
- I am not sure

102. Which of these options do experts recommend to prevent heart disease? *

Marca solo un óvalo.

- Take nutritional supplements
- Eat less fatty fish
- Eat less trans fat
- I am not sure

103. Which of these foods is most likely to raise people's blood cholesterol? *

Marca solo un óvalo.

- Eggs
- Vegetable oil
- Animal fats
- I am not sure

104. Generally, foods high in trans fats are ultra-processed foods and generate high environmental impacts *

Marca solo un óvalo.

- In agreement
- In disagreement
- I am not sure

105. Light foods are always considered a good option because they are low in calories *

Marca solo un óvalo.

- In agreement
- In disagreement
- I am not sure

106. To maintain a healthy weight, people must eliminate fat completely *

Marca solo un óvalo.

- In agreement
- In disagreement
- I am not sure

107. Approximately, what amount of alcoholic beverages is recommended to consume as a maximum per day? (The exact number depends on the size and type of the drink) *

Marca solo un óvalo.

- It is better to avoid its consumption, but a maximum of 1 drink is recommended for men and women
- 1 drink recommended for men and women
- 2 drinks are recommended for men and women
- 3 drinks for men and 2 for women

108. Fermented alcoholic beverages, consumed in moderation can... *

Marca solo un óvalo.

- Help improve the composition of the intestinal microbiota
- Reduce the risk of type 2 diabetes
- Reduce the risk of heart attacks
- I am not sure

109. In the long term, the consumption of foods high in sugars, salt, saturated and trans fats and alcoholic beverages *

Marca solo un óvalo.

- It is more expensive than eating healthy and sustainable
- It is less expensive than eating healthy and sustainable
- It costs the same as eating healthy and sustainable
- I am not sure

Este contenido no ha sido creado ni aprobado por Google.

Google Formularios
