# Supplementary material for: Sustainable-psycho-nutritional intervention programme for a sustainable diet (the ‘NutriSOS’ study) and its effects on eating behaviour, diet quality, nutritional status, physical activity, metabolic biomarkers, gut microbiota and water and carbon footprints in Mexican population: study protocol of an mHealth randomised controlled trial
Source: Br J Nutr. 2023 Mar 30;130(10):1823–38. doi: 10.1017/S0007114523000843 (PMC10587386; doi:10.1017/S0007114523000843)
Supplement: Supplementary file 1 [file S0007114523000843sup.zip › S0007114523000843sup002.zip!/Online Supplementary material 7. Sustaiable nutrition questionnaire Spanish.pdf]

Cuestionario Inicial de Conocimientos Nutricionales Sostenibles NutriSó

Hola de nuevo :)

Uno de los objetivos del estudio NutriSó, es incrementar tus conocimientos nutricionales y sobre sostenibilidad alimentaria. Para ello, prepararemos una serie de videos y webinars que te ayudarán a aprender más sobre estos temas, pero antes, queremos conocer un poco sobre tus conocimientos actuales, para así preparar material adecuado a tus requerimientos.

Por lo anterior, te pedimos que respondas a las siguientes preguntas de la manera más honesta posible. Este no es un examen, sino más bien, una encuesta. Así que no te preocupes, y por favor responde sin consultar fuentes de información.

Recuerda que todos tus datos son privados y están protegidos por la Ley Federal de Protección de Datos Personales en Posesión de los Particulares.

*Obligatorio

1. Correo *

____

2. Apellido paterno *

____

3. Apellido materno *

____

4. Nombre *

____

5. Si lo recuerdas, coloca tu número de participante por favor

____

Dietas sostenibles

A continuación, se presentan algunas preguntas sobre conocimientos nutricionales y de sosteniblidad. Por favor, responde sin consultar ninguna fuente de información. Recuerda que no serás juzgado en ningún momento por tus respuestas y este no es un examen.

6. Una dieta sostenible es.... *

Marca solo un óvalo.

- ◯ Una dieta que sostiene a muchas familias al mismo tiempo
- ◯ Una dieta para bajar de peso rapidamente y no recuperarlo
- ◯ Son patrones dietéticos que promueven la salud y nutrición, tienen bajo impacto medioambiental, son accesibles, asequibles y culturalmente aceptables
- ◯ Son patrones dietéticos que promueven un bajo impacto medioambiental aunque se tengan que ignorar aspectos como la nutrición y la cultura
- ◯ No estoy seguro/a/e

7. ¿Para lograr una dieta sostenible, se recomienda que las personas consuman, respecto a su ingesta actual... más, la misma cantidad o menos de los siguientes alimentos? (marca por favor una casilla por alimento) *

Marca solo un óvalo por fila.

	Más	La misma cantidad	Menos	No estoy seguro/a/e
**Alimentos y platillos mexicanos con baja o nula cantidad de carnes, y a base de maíz y leguminosas como frijoles**	⬭	⬭	⬭	⬭
**Frutas**	⬭	⬭	⬭	⬭
**Verduras**	⬭	⬭	⬭	⬭
**Cereales integrales**	⬭	⬭	⬭	⬭
**Leguminosas**	⬭	⬭	⬭	⬭
**Lácteos (leche, quesos y yogurt)**	⬭	⬭	⬭	⬭
**Semillas y grasas saludables**	⬭	⬭	⬭	⬭
**Huevo**	⬭	⬭	⬭	⬭
**Pescados y mariscos**	⬭	⬭	⬭	⬭
**Pollo**	⬭	⬭	⬭	⬭
**Carnes rojas y procesadas**	⬭	⬭	⬭	⬭
**Alimentos ultra-procesados**	⬭	⬭	⬭	⬭

(refrescos, galletas)				
Aceites y grasas trans y saturadas	⬭	⬭	⬭	⬭
Azúcar añadida en los alimentos ya preparados	⬭	⬭	⬭	⬭
Sal añadida a los alimentos ya cocinados	⬭	⬭	⬭	⬭
Agua natural	⬭	⬭	⬭	⬭
Bebidas alcohólicas	⬭	⬭	⬭	⬭

8. La huella hídrica de los alimentos es... *

Marca solo un óvalo.

- ⬭ El agua que se guarda en el subsuelo y nunca es utilizada por el ser humano
- ⬭ El agua dulce necesaria para producir un alimento
- ⬭ El agua salada que está en los oceanos
- ⬭ No estoy seguro/a/e

9. ¿Cuál de los siguientes alimentos requiere más agua durante su producción por kilogramo de producto? *

Marca solo un óvalo.

- Cereales azucarados o con grasa como galletas y pasteles
- Carne de res
- Huevo de gallina
- Carne de cerdo
- Aguacate
- No estoy seguro/a/e

10. La huella de carbono de un alimento es... *

Marca solo un óvalo.

- La cantidad de carbón que se genera al quemar basura o desechos alimenticios
- La cantidad de gases de efecto invernadero que se emiten por la producción de un alimento
- El humo que se genera durante los incendios
- No estoy seguro/a/e

11. ¿Cuál de los siguientes alimentos emite más gases de efecto invernadero durante su producción por kilogramo de producto? *

Marca solo un óvalo.

- Aceites vegetales
- Carne de res
- Huevo de gallina
- Carne de cerdo
- Tortilla de maíz
- No estoy seguro/a/e

12. ¿Crees que los siguientes alimentos tienen un contenido bajo o alto de fibra? *

Marca solo un óvalo por fila.

	Bajo contenido de fibra	Alto contenido de fibra	No estoy seguro/a/e
**Avena**			
**Amaranto**			
**Papaya**			
**Arroz blanco**			
**Huevos**			
**Papa o camote con cáscara**			
**Pasta**			

13. ¿Crees que los siguientes alimentos son una fuente baja o alta de proteína? *

Marca solo un óvalo por fila.

	Baja fuente de proteína	Alta fuente de proteína	No estoy seguro/a/e
**Pollo**	◯	◯	◯
**Queso fresco o panela**	◯	◯	◯
**Frutas**	◯	◯	◯
**Frijoles cocidos**	◯	◯	◯
**Mantequilla**	◯	◯	◯
**Nueces**	◯	◯	◯

14. Si quisieras comprar una opción de comida sostenible en un restaurante ¿qué opción elegirías? *

Marca solo un óvalo.

◯ Tacos de res con cebolla y cilantro con agua fresca de limón endulzada

◯ Sopitos de picadillo de res con zanahoria y papa con un refresco sin azúcar

◯ Enfrijoladas con queso fresco y lechuga con agua de jamaica sin azúcar

◯ No estoy seguro/a/e

15. ¿Que desayuno crees que es más sostenible? *

Marca solo un óvalo.

- Huevos a la mexicana con jitomate, cebolla y nopales, con tortilla de maíz, frijoles de la olla y agua natural
- Quesadillas de panela con poca carne de res y un jugo de naranja
- Chilaquiles rojos con arrachera y un huevo hervido con jugo verde de espinacas y espárragos
- No estoy seguro/a/e

16. ¿Qué opción de postre crees que sea más sostenible? *

Marca solo un óvalo.

- Arroz cocido con leche descremada con piloncillo y canela
- Pastel de chocolate con crema batida
- Nieve de chocolate empacada en un recipiente reciclable
- No estoy seguro/a/e

Actividad física

Ahora se presentan algunas preguntas sobre actividad física. Responde sin consultar fuentes de información por favor. Recuerda que este no es un examen.

17. ¿Cuál es la cantidad mínima de minutos de actividad física que se debe realizar diariamente para estar saludable en el contexto de las dietas sostenibles? *

Marca solo un óvalo.

- 5 minutos
- 10 minutos
- 20 minutos
- 30 minutos
- 40 minutos
- 50 minnutos
- 60 minutos
- Más de 60 minutos
- No estoy seguro/a/e

18. Sobre la actividad física es cierto que... *

Marca solo un óvalo.

- Se recomienda únicamente realizar ejercicios cardiovasculares como caminar o correr
- Es recomendable combinar ejercicios cardiovasculares con ejercicios de fuerza
- Es mejor no realizar actividad física muy vigorosa y sólo comer saludablemente
- No estoy seguro/a/e

19. Usar la bicicleta o caminar como medio de transporte tiene un efecto positivo sobre el medio ambiente *

Marca solo un óvalo.

- De acuerdo
- En desacuerdo
- No estoy seguro/a/e

20. Señala los beneficios para la salud de realizar actividad física *

Marca solo un óvalo.

- Disminuye la presión arterial y regula los niveles de glucosa en sangre
- Hace que pierdas grasa corporal aunque consumas más calorías de la que gastas
- No estoy seguro/a/e

21. Una manera de ahorrar dinero en transporte es *

Marca solo un óvalo.

- Utilizar gasolina de baja combustión
- Caminar y utilizar bicicleta siempre que sea posible
- Ahorrar para comprar un auto eléctrico
- No estoy seguro/a/e

22. Una manera adecuada de realizar actividad física sin notarlo es... *

Marca solo un óvalo.

- Correr por 20 minutos cerca de casa
- Realizar quehacer en casa y cultivar mis propios alimentos
- Inscribirme a clases de aeróbicos
- No estoy seguro/a/e

23. El mejor ejercicio es... *

Marca solo un óvalo.

- CrossFit o ejercicios multifuncionales
- Zumba o ritmos latinos
- Caminar o trotar
- Aquel que combine ejercicios cardiovasculares y de fuerza, y disfrute realizarlo
- No estoy seguro/a/e

24. Para que el ejercicio pueda ayudarnos a prevenir enfermedades crónicas, este debe incrementar nuestra frecuencia cardiaca y respiración *

Marca solo un óvalo.

- De acuerdo
- En desacuerdo
- No estoy seguro/a/e

Alimentos y platillos mexicanos

La dieta tradicional mexicana es deliciosa, pero queremos saber que piensas sobre ella desde una perspectiva de sostenibilidad. Por favor responde de la manera más honesta posible.

25. Comer tortilla de maíz todos los días provoca aumento de peso *

Marca solo un óvalo.

- De acuerdo
- En desacuerdo
- No estoy seguro/a/e

26. La fibra puede disminuir las posibilidades de aumentar de peso *

Marca solo un óvalo.

- De acuerdo
- En desacuerdo
- No estoy seguro/a/e

27. Selecciona la opción que consideres que sea más sosenible *

Marca solo un óvalo.

- Pozole de maíz con pollo y mucha lechuga
- Pozole de berenjena y quinoa
- Pozole de maíz con cerdo y mucha lechuga
- No es recomendable consumir ninguna versión de pozole
- No estoy seguro/a/e

28. El maíz tiene una huella hídrica y de carbono... *

Marca solo un óvalo.

- Mayor al de las carnes
- Igual al de las carnes
- Menor al de las carnes
- No estoy seguro/a/e

29. Son cereales mexicanos *

Marca solo un óvalo.

- Avena y quinoa
- Maíz y amaranto
- Maíz y trigo
- No estoy seguro/a/e

30. La dieta tradicional mexicana es muy cara como para consumirla todos los días *

Marca solo un óvalo.

- De acuerdo
- En desacuerdo
- No estoy seguro/a/e

Frutas y verduras

Seguramente has escuchado que las frutas y verduras son saludables, ¿cierto? Queremos saber tu opinión sobre algunos aspectos relacionados

31. ¿Cuántas porciones de frutas y verduras se recomienda consumir, mínimamente, al día para logar una dieta sostenible? (Una porción podría ser, por ejemplo, una manzana o una taza de papaya) *

Marca solo un óvalo.

- 2
- 3
- 4
- 5 o más
- No estoy seguro/a/e

32. Se recomienda que las verduras se consuman... *

Marca solo un óvalo.

- Después de comer proteína como carnes
- Antes de iniciar a comer un platillo, por ejemplo, antes de la carne y del arroz
- En medio de la comida, por ejemplo, iniciar con el arroz, luego verduras y al final la carne
- No estoy seguro/a/e

33. ¿Cuál de las siguientes combinaciones de ensalada aportaría la variedad más alta de vitaminas y antioxidantes? *

Marca solo un óvalo.

- Lechuga, espinacas y pepino
- Nopales, jitomate, cebolla y zanahoria
- Pimiento morrón rojo, jitomate y jitomate cherry
- No estoy seguro/a/e

34. ¿Cuál de estas enfermedades se relaciona con un bajo consumo de fibra? *

Marca solo un óvalo.

- Enfermedades intestinales
- Anemia
- Caries dentales
- No estoy seguro/a/e

35. Entre más fibra se consume, generalmente la dieta tiene un impacto medioambiental... *

Marca solo un óvalo.

- Más alto
- Más bajo
- No estoy seguro/a/e

36. Comer frutas y verduras todos los días es muy caro *

Marca solo un óvalo.

- De acuerdo
- En desacuerdo
- No estoy seguro/a/e

37. Selecciona las frutas y verduras que se consideran mexicanas *

Marca solo un óvalo.

- Nopal, jitomate, espárragos, berenjena, sandía y mango
- Nopal, jitomate, chile, calabaza, piña y papaya
- Zanahoria, champiñones, brócoli, manzana y nisperos
- No estoy seguro/a/e

Cereales integrales y tubérculos

Queremos saber tu opinión sobre los cereales integrales. Por favor, responde de la manera más honesta posible y sin consultar fuentes de información.

38. ¿Qué cantidad de tu plato se recomienda que sean cereales integrales o tubérculos, como tortilla de maíz o camote? *

Marca solo un óvalo.

- Un cuarto del plato
- Un tercio del plato
- La mitad del plato
- No estoy seguro/a/e

39. La tortilla de maíz... *

Marca solo un óvalo.

- Aporta energía, carbohidratos complejos, fibra y vitaminas del complejo B
- Aporta carbohidratos simples y vitamina b12
- Aporta altas cantidades de proteína y creatina
- Es una fuente importante de grasas saludables como omega 3
- No estoy seguro/a/e

40. La papa y el camote son... *

Marca solo un óvalo.

- Verduras que aportan poca energía
- Tubérculos que aportan carbohidratos complejos
- Frutas que aportan azucares
- Grasas saludables que aportan omega 3
- No estoy seguro/a/e

41. Para lograr una dieta sostenible, los cereales y tubérculos deben estar presentes en nuestra dieta... *

Marca solo un óvalo.

- Todos los días
- 3 a 4 veces a la semana
- 1 a 2 veces por semana
- Nunca
- No estoy seguro/a/e

42. Comer cereales integrales es muy caro *

Marca solo un óvalo.

- De acuerdo
- En desacuerdo
- No estoy seguro/a/e

Leguminosas

Ahora queremos conocer un poco más sobre lo que opinas sobre las leguminosas. Responde honestamente y recuerda que esto no es un examen.

43. Las leguminosas aportan *

Marca solo un óvalo.

- Proteína vegetal y carbohidratos complejos
- Grasas saturadas
- Azucares y proteína de alto valor biológico
- Vitamina b12 y fibra
- No estoy seguro/a/e

44. De las leguminosas es verdad que... *

Marca solo un óvalo.

- Pueden sustituir a las carnes en lo que respecta a proteína
- Nunca podrán sustituir a las carnes respecto a proteína
- No estoy seguro/a/e

45. ¿Qué beneficios aportan las leguminosas a la salud? *

Marca solo un óvalo.

- Previenen el desarrollo de diabetes tipo 2 y mejoran la composición de la microbiota intestinal (bacterias en el intestino)
- Previenen el desarrollo del cáncer
- Hacen que bajes de peso sin necesidad de hacer ejercicio
- No estoy seguro/a/e

46. A nivel medioambiental, las leguminosas... *

Marca solo un óvalo.

- Tienen una huella hídrica y de carbono similar a la de las carnes rojas
- Tienen una huella hídrica y de carbono más baja que la de las carnes rojas
- Su impacto medioambiental es más alto que el de las carnes rojas
- No estoy seguro/a/e

47. Sustituir las carnes por leguminosas es una manera de ahorrar dinero *

Marca solo un óvalo.

- De acuerdo
- En desacuerdo
- No estoy seguro/a/e

48. Son ejemplos de leguminosas mexicanas, según la dieta de la milpa, *

Marca solo un óvalo.

- Frijoles, lentejas y habas
- Habas, garbanzos y alubias
- Piñón, cacahuate y chía
- No estoy seguro/a/e

Lácteos

A continuación, se presentan algunas preguntas sobre el consumo de lácteos. Por favor, responde de la manera más honesta posible.

49. Cuando se consumen lácteos en una dieta sostenible, se recomienda consumir... *

Marca solo un óvalo.

- Lacteos enteros (con grasa)
- Lacteos reducidos en grasa (descremados o semidescremados)
- Una mezcla de lácteos grasosos y sin grasa
- Ningún lácteo, pues es mejor evitarlos
- No estoy seguro/a/e

50. La cantidad de calcio en un vaso de leche descremada o light, comparada con un vaso de leche entera es: *

Marca solo un óvalo.

- Casi la misma
- Mucho más alta
- Mucho más baja
- No estoy seguro/a/e

51. Si quisieras comprar un yogurt en el mercado ¿cuál opción tendría menos azucares? *

Marca solo un óvalo.

- Yogurt 0% grasa sabor fresa
- Yogurt natural
- Yogurt cremoso de frutas
- No estoy seguro/a/e

52. Únicamente dejando de comer lácteos puedo logar consumir una dieta sostenible *

Marca solo un óvalo.

- De acuerdo
- En desacuerdo
- No estoy seguro/a/e

53. Para poder consumir lácteos, debo elegir los más costosos para garantizar la mayor calidad posible *

Marca solo un óvalo.

- De acuerdo
- En desacuerdo
- No estoy seguro/a/e

54. Son ejemplos de quesos sostenibles para México *

Marca solo un óvalo.

- Queso mozzarella y parmesano
- Queso panela y requesón
- Queso gouda y americano
- No estoy seguro/a/e

Semillas y grasas saludables

A continuación, se presentan algunas preguntas sobre las semillas y grasas saludables en nuestra alimentación. Por favor, responde sin consultar fuentes de información.

55. ¿Cuáles de estos tipos de grasas recomiendan los expertos que las personas deben comer menos para lograr una dieta sostenible? (marque una casilla por alimento) *

Marca solo un óvalo por fila.

	Comer menos	Comer la misma cantidad	No estoy seguro/a/e
**Grasas insaturadas**	⬭	⬭	⬭
**Grasas trans**	⬭	⬭	⬭
**Grasas saturadas**	⬭	⬭	⬭

56. ¿Cuál es el principal tipo de grasa presente en cada uno de estos alimentos? *

Marca solo un óvalo por fila.

	Grasas poliinsaturadas	Grasas monoinsaturadas	Grasas saturadas	Colesterol	No estoy seguro/a/e
**Aceite de oliva extra virgen**	⬭	⬭	⬭	⬭	⬭
**Mantequilla**	⬭	⬭	⬭	⬭	⬭
**Aceite de girasol**	⬭	⬭	⬭	⬭	⬭
**Huevos**	⬭	⬭	⬭	⬭	⬭

57. Son semillas sostenibles para la dieta mexicana *

Marca solo un óvalo.

- Cacahuate sin sal, chía y semillas de calabaza
- Nuez pecana, almendras y cacahuates
- Cacahuates garapiñados, chía y nueces de la india
- No estoy seguro/a/e

58. El aguacate aporta los siguientes beneficios para la salud *

Marca solo un óvalo.

- Mejora la composición de la microbiota intestinal y tiene grasas saludables que previenen enfermedades del corazón
- Es muy bajo en calorías y dos piezas al día ayudan a disminuir el porcentaje de grasa corporal
- Es alto en proteína, por lo que fortalece los músculos
- No estoy seguro/a/e

59. Desde una perspectiva de sostenibilidad, ¿el aceite de oliva puede incluirse en la dieta tradicional mexicana? *

Marca solo un óvalo.

- Si
- No
- Depende de la frecuencia y cantidad de consumo
- No estoy seguro/a/e

60. Una manera para disminuir el impacto medioambiental del aguacate del Sur de Jalisco es… *

Marca solo un óvalo.

- Consumir sólo aquellos que me obsequían, si está barato o sembrar mis propios aguacates
- Comprar sólo aquellos aguacates orgánicos de calidad de exportación
- No estoy seguro/a/e

61. Con respecto a relación a precio, nutrición, medio ambiente y cultura, las semillas y grasas saludables pueden ser opciones sostenibles si… *

Marca solo un óvalo.

- Se consumen en la frecuencia y cantidad adecuados
- Se consumen sólo de manera mensual
- No pueden ser opciones sostenibles por su alto contenido de grasas
- No estoy seguro/a/e

Huevos

Ahora, se presentan algunas preguntas sobre los huevos de gallina. Por favor, responde de la manera más honesta.

62. La yema de huevo eleva los niveles de colesterol en sangre *

Marca solo un óvalo.

- Cierto
- Falso
- No estoy seguro/a/e

63. El huevo es una fuente elevada de proteína de alto valor biológico *

Marca solo un óvalo.

- De acuerdo
- En desacuerdo
- No estoy seguro/a/e

64. El huevo es una fuente barata de proteína *

Marca solo un óvalo.

- De acuerdo
- En desacuerdo
- No estoy seguro/a/e

65. Una receta sostenible con huevo es.... *

Marca solo un óvalo.

- Huevo en salsa roja con jitomate, frijoles de la olla y tortilla de maíz
- Huevo a la mexicana con tocino y aceite de oliva
- Huevos en omelette con espárragos
- No estoy seguro/a/e

66. ¿Cuántos huevos recomienda comer como máximo por semana, la dieta para salud planetaria? *

Marca solo un óvalo.

- 3 piezas
- 4 piezas
- 5 piezas
- 8 huevos
- Se recomienda evitar comer huevos

67. Los huevos de rancho o producidos localmente, generalmente son... *

Marca solo un óvalo.

- Más sucios, por lo que no se recomienda su consumo
- Una opción más sostenible que los producidos a nivel industrial
- Menos saludables y peligrosos para la salud
- No estoy seguro/a/e

Pescados y mariscos

En esta ocasión, se presentan algunas preguntas sobre pescados y mariscos. Por favor, responde de la manera más honesta posible.

68. ¿Cuántas veces a la semana se recomienda consumir pescados producidos en México, como mojarra o tilapia, dentro de una dieta sostenible? *

Marca solo un óvalo.

- 1 a 2 veces por semana
- 3 a 4 veces por semana
- Todos los días
- No estoy seguro/a/e

69. El pescado aporta... *

Marca solo un óvalo.

- Omega 3 y proteína de alto valor biológico
- Fibra y grasas monoinsaturadas
- Proteina vegetal y carbohidratos
- No estoy seguro/a/e

70. Según la dieta para salud planetaria, comer más de 196 gramos de pescado a la semana tiene un impacto medioambiental elevado *

Marca solo un óvalo.

- De acuerdo
- En desacuerdo
- No estoy seguro/a/e

71. Sólo el pescado que es caro aporta sufientes nutrientes a mi cuerpo *

Marca solo un óvalo.

- De acuerdo
- En desacuerdo
- No estoy seguro/a/e

72. Generalmente, entre más lejos venga el pescado y/o mariscos más alto será su impacto medioambiental *

Marca solo un óvalo.

- De acuerdo
- En desacuerdo
- No estoy seguro/a/e

Pollo

Ahora, se presentan algunas preguntas sobre el pollo, por favor responde sin consultar fuentes de información.

73. El pollo tiene una huella hídrica y de carbono más baja que la carne de res y el cerdo *

Marca solo un óvalo.

- De acuerdo
- En desacuerdo
- No estoy seguro/a/e

74. El pollo aporta proteína de alto valor biológico *

Marca solo un óvalo.

- De acuerdo
- En desacuerdo
- No estoy seguro/a/e

75. En el contexto de las dietas sostenibles se recomienda... *

Marca solo un óvalo.

- Comer pollo máximo 1 a 2 veces por semana
- Comer pollo máximo 3 a 4 veces por semana
- Comer pollo más de 4 veces por semana
- No comer pollo

76. El pollo puede ser una opción sostenible si... *

Marca solo un óvalo.

- Lo consumimos para sustituir la carne de res en todo momento
- Lo consumimos con la frecuencia y cantidad adecuada
- El pollo no puede ser sostenible
- No estoy seguro/a/e

77. Al sustituir el pollo por leguminosas en los platillos, podría ahorrar hasta un 83% del dinero que gasto en la compra del pollo *

Marca solo un óvalo.

- De acuerdo
- En desacuerdo
- No estoy seguro/a/e

Carnes rojas y procesadas

Ahora se presentan preguntas sobre carnes rojas y procesadas. Recuerda que este no es un examen, sino una encuesta, así que te pedimos que respondas honestamente y sin consultar fuentes de información.

78. La carne de res es el alimento que gasta la mayor cantidad de agua durante su producción *

Marca solo un óvalo.

- De acuerdo
- En desacuerdo
- No estoy seguro/a/e

79. Comer carne de res frecuentemente incrementa las posibilidades de enfermar de cáncer *

Marca solo un óvalo.

- De acuerdo
- En desacuerdo
- No estoy seguro/a/e

80. Para mantener un peso saludable, las personas deben comer una dieta rica en proteínas *

Marca solo un óvalo.

- De acuerdo
- En desacuerdo
- No estoy seguro/a/e

81. Selecciona por favor los alimentos que tienen la mayor huella de carbono *

Marca solo un óvalo.

- Carne de cerdo y tocino
- Carne de res y borrego
- Pescados y mariscos
- Salchichas y jamón del cerdo
- No estoy seguro/a/e

82. ¿Cuántas veces a la semana se recomienda comer carne de res? *

Marca solo un óvalo.

- 1 vez por semana o menos
- 2 veces a la semana
- 3 a 4 veces por semana
- No estoy seguro/a/e

83. Al sustituir la carne de res por leguminosas, es posible ahorrar hasta *

Marca solo un óvalo.

- Un 20% de lo que gastaría si como carne
- Hasta un 50% de lo que gastaría si como carne
- Hasta un 90% de lo que gastaría si como carne
- No estoy seguro/a/e

Alimentos ultra-procesados

Ahora, se presentan algunas preguntas sobre alimentos ulta-procesados, es decir, altamente procesados. Por favor, responde honestamente y sin consultar fuentes de información.

84. Comparados con los alimentos sin procesar o minimamente procesados, los alimentos ultra-procesados son: *

Marca solo un óvalo.

- Más altos en calorías
- Más altos en fibra
- Más bajos en sal
- No estoy seguro/a/e

85. Consumir alimentos ultra-procesados de manera regular se ha asociado con... *

Marca solo un óvalo.

- Prevención de diabetes tipo 2 y enfermedades del corazón
- Desarrollo de diabetes tipo 2 y enfermedades del corazón
- Su consumo no se ha asociado con el desarrollo ni prevención de ninguna enfermedad
- No estoy seguro/a/e

86. El consumo de alimentos ultra-procesados altera la microbiota intestinal, lo que puede diminuir la respuesta inmune del cuerpo *

Marca solo un óvalo.

- De acuerdo
- En desacuerdo
- No estoy seguro/a/e

87. Las grandes fábricas de alimentos ultra-procesados generan altos impactos hacia el medio ambiente *

Marca solo un óvalo.

- De acuerdo
- En desacuerdo
- No estoy seguro/a/e

88. En el contexto de las dietas sostenibles, el consumo de alimentos ultra-procesados *

Marca solo un óvalo.

- Se desaconseja
- Se recomienda comerlos 1 vez a la semana
- Se recomienda comerlos de 2 a 3 veces por semana
- No estoy seguro/a/e

89. Comer alimentos ultra-procesados todos los días resulta *

Marca solo un óvalo.

- Más barato que comer alimentos saludables
- Más caro que comer alimentos saludables
- Igual de caro o barato que comer alimentos saludables
- No estoy seguro

90. Los alimentos ultra-procesados pueden formar parte de una dieta tradicional mexicana sostenible *

Marca solo un óvalo.

- De acuerdo
- En desacuerdo
- No estoy seguro/a/e

91. Generalmente, entre más larga sea la lista de ingredientes del producto, más alto será su impacto medioambiental *

Marca solo un óvalo.

- De acuerdo
- En desacuerdo
- No estoy seguro/a/e

Azúcar, sal, grasas trans, saturadas y bebidas alcohólicas

Finalmente, se presentan preguntas sobre el consumo de azúcar, sal, grasas trans, saturadas y bebidas alcohólicas.

92. ¿Crees que los siguientes alimentos tienen un contenido alto o bajo de azúcares? *

Marca solo un óvalo por fila.

	Bajo contenido de azucares	Alto contenido de azucares	No estoy seguro/a/e
**Refrescos light o sin azúcar**			
**Yogurt natural**			
**Nieve**			
**Salsa ketchup**			
**Melón cantalú**			

93. ¿Cuál de estas enfermedades está relacionada con la cantidad de azúcar que consumen las personas? *

Marca solo un óvalo.

- Hipertensión arterial (presión alta)
- Caries dentales y diabetes tipo 2
- Anemia
- No estoy seguro/a/e

94. ¿Cuál de estas opciones recomiendan los expertos para prevenir la diabetes tipo 2? *

Marca solo un óvalo.

- Consumir menos alimentos refinados
- Beber más jugos de fruta
- Comer más carnes rojas y procesadas
- No estoy seguro/a/e

95. ¿Cuál de estos alimentos se clasifica con un índice glucémico alto? *

Marca solo un óvalo.

- Cereales integrales
- Pan blanco
- Frutas y vegetales
- No estoy seguro/a/e

96. Desde una perspectiva medioambiental la huella hídrica y de carbono de los azúcares es… *

Marca solo un óvalo.

- Baja en comparación con los alimentos de origen animal
- Alta en comparación con los alimentos de origen animal
- Igual que la de los alimentos de origen animal
- No estoy seguro/a/e

97. ¿Crees que los siguientes alimentos tienen un contenido bajo o alto de sal? *

Marca solo un óvalo por fila.

	Bajo contenido de sal	Alto contenido de sal	No estoy seguro/a/e
**Cereales de caja para el desayuno**			
**Vegetales congelados**			
**Pan de caja**			
**Sopas enlatadas**			
**Botanas de papa o maiz**			

98. Una manera saludable de darle sabor a los alimentos sin agregar sal es agregarle *

Marca solo un óvalo.

- Leche de coco
- Hierbas como pimienta, orégano, hojas de laurel
- Salsa de soya
- No estoy seguro/a/e

99. ¿Cuál de estas enfermedades está relacionada con la cantidad de sal (o sodio) que consumen las personas? *

Marca solo un óvalo.

- Hipotiroidismo
- Hipertensión arterial (presión alta)
- Diabetes tipo 2
- No estoy seguro/a/e

100. Generalmente los alimentos con alto contenido de sodio son altamente procesados, por lo que su impacto medioambiental tiende a ser elevado *

Marca solo un óvalo.

- De acuerdo
- En desacuerdo
- No estoy seguro/a/e

101. ¿Cuáles de los siguientes alimentos crees que tienen mayor contenido de grasas trans? *

Marca solo un óvalo.

- Galletas y pastelitos
- Pescado
- Aceite de canola
- Huevos
- No estoy seguro/a/e

102. ¿Cuál de estas opciones recomiendan los expertos para prevenir enfermedades del corazón? *

Marca solo un óvalo.

- Tomar suplementos nutricionales
- Comer menos pescados grasosos
- Consumir menos grasas trans
- No estoy seguro/a/e

103. ¿Cuál de estos alimentos es más probable que eleve el colesterol en la sangre de las personas? *

Marca solo un óvalo.

- Huevos
- Aceite vegetal
- Grasas animales
- No estoy seguro/a/e

104. Generalmente, los alimentos altos en grasas trans son alimentos ultra-procesados y generan impactos medioambientales altos *

Marca solo un óvalo.

- De acuerdo
- En desacuerdo
- No estoy seguro/a/e

105. Los alimentos light siempre se consideran una buena opción porque son bajos en calorías *

Marca solo un óvalo.

- De acuerdo
- En desacuerdo
- No estoy seguro/a/e

106. Para mantener un peso saludable, las personas deben eliminar la grasa por completo *

Marca solo un óvalo.

- De acuerdo
- En desacuerdo
- No estoy seguro/a/e

107. Aproximadamente, ¿qué cantidad de bebidas alcohólicas se recomienda consumir como máximo por día? (El número exacto depende del tamaño y el tipo de la bebida) *

Marca solo un óvalo.

- Es mejor evitar su consumo, pero, cómo máximo, se recomienda 1 bebida para hombres y mujeres
- Se recomienda 1 bebida para hombres y mujeres
- Se recomiendan 2 bebidas para hombres y mujeres
- 3 bebidas para hombres y 2 para mujeres

108. Las bebidas alcohólicas fermentadas, consumidas con moderación pueden... *

Marca solo un óvalo.

- Ayudar a mejorar la composición de la microbiota intestinal
- Disminuir el riesgo de padecer diabetes tipo 2
- Disminuir el riesgo de infartos al corazón
- No estoy seguro/a/e

109. A largo plazo, el consumo de alimentos altos en azúcares, sal, grasas saturadas y trans y bebidas alcohólicas *

Marca solo un óvalo.

- Resulta más costoso que comer saludable y sostenible
- Resulta menos costoso que comer saludable y sostenible
- Cuesta igual que comer saludable y sostenible
- No estoy seguro/a/e

Este contenido no ha sido creado ni aprobado por Google.

Google Formularios
