# Supplementary material for: Sustainable-psycho-nutritional intervention programme for a sustainable diet (the ‘NutriSOS’ study) and its effects on eating behaviour, diet quality, nutritional status, physical activity, metabolic biomarkers, gut microbiota and water and carbon footprints in Mexican population: study protocol of an mHealth randomised controlled trial
Source: Br J Nutr. 2023 Mar 30;130(10):1823–38. doi: 10.1017/S0007114523000843 (PMC10587386; doi:10.1017/S0007114523000843)
Supplement: Supplementary file 1 [file S0007114523000843sup.zip › S0007114523000843sup002.zip!/Online Supplementary Material 8. COM-B Questionnaire English.pdf]

NutriSOS Intervention: COM-B Initial Questionnaire

Thank you very much for being part of NutriSOS :)

Before starting the intervention, we want to get to know you a little more, in order to provide you with the most personalized attention possible. Therefore, we ask you to answer the following questions as honestly as possible. Remember that all the data you provide us is and will always be confidential, and is protected by the Federal Law on the Protection of Personal Data Held by Private Parties.

*Obligatorio

1. Last name *

2. Mother's last name *

3. Name *

4. If you remember it, put your participant number please

Skills and ability to prepare food

Here are some questions about food preparation skills, so we can prescribe your meal plan in a way that best suits you and your lifestyle.

5. Do you know how to cook? *

Marca solo un óvalo.

- Yes
- No

6. How many minutes do you have a day to prepare your breakfast? *

Marca solo un óvalo.

- 5 minutes or less
- Between 10 and 20 minutes
- Between 20 and 30 minutes
- Between 30 and 40 minutes
- An hour
- More than one hour
- I have no time to prepare my breakfast

7. How many minutes do you have a day to prepare your lunch? *

Marca solo un óvalo.

- 5 minutes or less
- Between 10 and 20 minutes
- Between 20 and 30 minutes
- Between 30 and 40 minutes
- An hour
- More than one hour
- I have no time to prepare my food

8. How many minutes do you have a day to prepare your dinner? *

Marca solo un óvalo.

- 5 minutes or less
- Between 10 and 20 minutes
- Between 20 and 30 minutes
- Between 30 and 40 minutes
- An hour
- More than one hour
- I have no time to prepare my dinner

9. How many minutes do you have a day to prepare your snacks? *

Marca solo un óvalo.

- 5 minutes or less
- Between 10 and 20 minutes
- Between 20 and 30 minutes
- Between 30 and 40 minutes
- An hour
- More than one hour
- I have no time to prepare snacks

10. Do you like to cook? *

Marca solo un óvalo.

- Yes
- No

11. Do you have a kitchen with a stove? *

Marca solo un óvalo.

- Yes
- No

12. Do you have a kitchen with an oven? *

Marca solo un óvalo.

- Yes
- No

13. Do you have a kitchen with a blender? *

Marca solo un óvalo.

- Yes
- No

14. Do you have a kitchen with a refrigerator? *

Marca solo un óvalo.

- Yes
- No

15. Do you have containers to store food (tupers)? *

Marca solo un óvalo.

- Yes
- No

Opportunity to consume food

Here are some questions related to the time you have to consume food. This can help us provide you with recipes suited to your lifestyle.

16. How many minutes a day do you have for breakfast? *

Marca solo un óvalo.

- 5 minutes or less
- Between 10 and 20 minutes
- Between 20 and 30 minutes
- Between 30 and 40 minutes
- An hour
- More than one hour
- I don't have any time to eat breakfast

17. How many minutes do you have a day to eat? *

Marca solo un óvalo.

- 5 minutes or less
- Between 10 and 20 minutes
- Between 20 and 30 minutes
- Between 30 and 40 minutes
- An hour
- More than one hour
- I have no time to eat

18. How many minutes a day do you have for dinner? *

Marca solo un óvalo.

- 5 minutes or less
- Between 10 and 20 minutes
- Between 20 and 30 minutes
- Between 30 and 40 minutes
- An hour
- More than one hour
- I don't have any time for dinner

19. How many minutes do you have a day to consume snacks? *

Marca solo un óvalo.

- 5 minutes or less
- Between 10 and 20 minutes
- Between 20 and 30 minutes
- Between 30 and 40 minutes
- An hour
- More than one hour
- I have no time to consume snacks

20. Are there places selling fresh food (fruits, vegetables) near your home? *

Marca solo un óvalo.

- Yes
- No
- I do not know

21. Are there non-perishable food outlets near your home (beans, oatmeal, rice, cookies, etc.) *

Marca solo un óvalo.

- Yes
- No
- I do not know

Motivation

Now we want to know how interested you are in improving your lifestyle and taking care of the planet's health at the same time. To do this, we ask you to answer the following questions as honestly as possible. Remember that you will never be judged by your answers.

22. From 1 to 5, how much would you like to improve your eating habits? *

Marca solo un óvalo.

I would not like

1 ◯

2 ◯

3 ◯

4 ◯

5 ◯

I would like very much

23. From 1 to 5, how much would you like to eat healthier? *

Marca solo un óvalo.

	I would not like
1	⬭
2	⬭
3	⬭
4	⬭
5	⬭
	I would like very much

24. From 1 to 5, how much would you like to be a more physically active person? *

Marca solo un óvalo.

I would not like

1 ◯
2 ◯
3 ◯
4 ◯
5 ◯

I would like very much

25. Do you eat more food when you are sad, angry, or happy? *

Marca solo un óvalo.

- ◯ Yes
- ◯ No
- ◯ I do not know
- ◯ Otro: ______________

26. Do you eat less food when you are sad, angry or happy? *

Marca solo un óvalo.

- Yes
- No
- I do not know
- Otro: ____________________

27. What feelings increase your food intake? *

Selecciona todos los que correspondan.

- Sadness
- Anger
- Happiness
- Otro: ____________________

28. What feelings decrease your food intake? *

Selecciona todos los que correspondan.

- Sadness
- Anger
- Happiness
- Otro: ____________________

29. What kind of foods do you eat the most when you experience these feelings? *

Marca solo un óvalo.

- [ ] Sweets (bread, cookies, popsicles, ice cream)
- [ ] Salty (fries, hamburgers, peanuts)
- [ ] Any food regardless of its flavor
- [ ] Otro: ____________________

30. What kind of foods do you consume less when you experience these feelings? *

Marca solo un óvalo.

- [ ] Sweets (bread, cookies, popsicles, ice cream)
- [ ] Salty (fries, hamburgers, peanuts)
- [ ] Any food regardless of its flavor
- [ ] Otro: ____________________

31. Please write the habits that you would be willing to change, for example, eat more fruits and vegetables, eat less beef, exercise more, etc. *

____

____

____

____

____

Reasons for sustainable food consumption

Please write how important the following aspects are to you when you are going to consume food. 1 is not very important and 5 is very important

32. Produced without animals suffering *

Marca solo un óvalo.

	Less important
1	◯
2	◯
3	◯
4	◯
5	◯
	Very important

33. Produced in an animal-friendly way *

Marca solo un óvalo.

Less important

1 ◯

2 ◯

3 ◯

4 ◯

5 ◯

Very important

34. It is a free range or free range product *

Marca solo un óvalo.

Less important
1 ◯
2 ◯
3 ◯
4 ◯
5 ◯
Very important

35. Produced without exploitation *

Marca solo un óvalo.

Less important	
1	
2	
3	
4	
5	
Very important	

36. Produced without child labor *

Marca solo un óvalo.

	Less important
1	⬭
2	⬭
3	⬭
4	⬭
5	⬭
	Very important

37. Traded fairly *

Marca solo un óvalo.

	Less important
1	⬭
2	⬭
3	⬭
4	⬭
5	⬭
	Very important

38. It is produced in an environmentally friendly way, without disturbing nature *

Marca solo un óvalo.

	Less important
1	◯
2	◯
3	◯
4	◯
5	◯
	Very important

39. Produced with minimal CO_2 emissions *

Marca solo un óvalo.

	Less important
1	⬭
2	⬭
3	⬭
4	⬭
5	⬭
	Very important

40. Little water is used for its production *

Marca solo un óvalo.

	Less important
1	◯
2	◯
3	◯
4	◯
5	◯
	Very important

41. It is packaged in an environmentally friendly way *

Marca solo un óvalo.

	Less important
1	⬭
2	⬭
3	⬭
4	⬭
5	⬭
	Very important

42. It is a local/regional product *

Marca solo un óvalo.

	Less important
1	◯
2	◯
3	◯
4	◯
5	◯
	Very important

43. It is a seasonal product *

Marca solo un óvalo.

Less important
1 ◯
2 ◯
3 ◯
4 ◯
5 ◯
Very important

44. Comes from nearby (short transportation distance) *

Marca solo un óvalo.

Less important
1 ◯
2 ◯
3 ◯
4 ◯
5 ◯
Very important

Este contenido no ha sido creado ni aprobado por Google.

Google Formularios
