# Supplementary material for: Sustainable-psycho-nutritional intervention programme for a sustainable diet (the ‘NutriSOS’ study) and its effects on eating behaviour, diet quality, nutritional status, physical activity, metabolic biomarkers, gut microbiota and water and carbon footprints in Mexican population: study protocol of an mHealth randomised controlled trial
Source: Br J Nutr. 2023 Mar 30;130(10):1823–38. doi: 10.1017/S0007114523000843 (PMC10587386; doi:10.1017/S0007114523000843)
Supplement: Supplementary file 1 [file S0007114523000843sup.zip › S0007114523000843sup002.zip!/Online Supplementary Material 9. COM-B Questionnaire Spanish.pdf]

Intervención NutriSó: Cuestionario Inicial COM-B

Muchas gracias por ser parte de NutriSó :)

Antes de comenzar con la intervención, queremos conocerte un poco más, para poder brindarte la atención más personalizada posible. Por ello, te pedimos que respondas de la manera más honesta las siguientes preguntas. Recuerda que todos los datos que nos proporciones son y serán siempre confidenciales, y están protegidos por la Ley Federal de Protección de Datos Personales en Posesión de los Particulares.

*Obligatorio

1. Correo *

____

2. Apellido paterno *

____

3. Apellido materno *

____

4. Nombre *

____

5. Si lo recuerdas, coloca tu número de participante por favor

____

Habilidades y capacidad para preparación de alimentos

A continuación se presentan algunas preguntas sobre habilidades para preparación de alimentos, para poder prescribir tu plan alimenticio de manera que mejor se adapte a ti y tu estilo de vida.

6. ¿Sabes conocinar? *

Marca solo un óvalo.

- Si
- No

7. ¿Con cuántos minutos cuentas al día para preparar tu desayuno? *

Marca solo un óvalo.

- 5 minutos o menos
- Entre 10 y 20 minutos
- Entre 20 y 30 minutos
- Entre 30 y 40 minutos
- Una hora
- Más de una hora
- No tengo ningún momento para preparar mi desayuno

8. ¿Con cuántos minutos cuentas al día para preparar tu comida? *

Marca solo un óvalo.

- 5 minutos o menos
- Entre 10 y 20 minutos
- Entre 20 y 30 minutos
- Entre 30 y 40 minutos
- Una hora
- Más de una hora
- No tengo ningún momento para preparar mi comida

9. ¿Con cuántos minutos cuentas al día para preparar tu cena? *

Marca solo un óvalo.

- 5 minutos o menos
- Entre 10 y 20 minutos
- Entre 20 y 30 minutos
- Entre 30 y 40 minutos
- Una hora
- Más de una hora
- No tengo ningún momento para preparar mi cena

10. ¿Con cuántos minutos cuentas al día para preparar tus colaciones? *

Marca solo un óvalo.

- 5 minutos o menos
- Entre 10 y 20 minutos
- Entre 20 y 30 minutos
- Entre 30 y 40 minutos
- Una hora
- Más de una hora
- No tengo ningún momento para preparar colaciones

11. ¿Te gusta cocinar? *

Marca solo un óvalo.

- Si
- No

12. ¿Tienes una cocina con estufa? *

Marca solo un óvalo.

- Si
- No

13. ¿Tienes una cocina con horno? *

Marca solo un óvalo.

- Si
- No

14. ¿Tienes una cocina con licuadora? *

Marca solo un óvalo.

- Si
- No

15. ¿Tienes una cocina con refrigerador? *

Marca solo un óvalo.

- Si
- No

16. ¿Cuentas con recipientes para almacenar alimentos (tupers)? *

Marca solo un óvalo.

- Si
- No

Oportunidad para consumir alimentos

A continuación, se presentan algunas preguntas relacionadas con el tiempo con el que dispones para consumir alimentos. Esto nos puede ayudar a proporcionarte recetas adecuadas a tu estilo de vida.

17. ¿Con cuántos minutos cuentas al día para desayunar? *

Marca solo un óvalo.

- 5 minutos o menos
- Entre 10 y 20 minutos
- Entre 20 y 30 minutos
- Entre 30 y 40 minutos
- Una hora
- Más de una hora
- No tengo ningún momento para desayunar

18. ¿Con cuántos minutos cuentas al día para comer? *

Marca solo un óvalo.

- 5 minutos o menos
- Entre 10 y 20 minutos
- Entre 20 y 30 minutos
- Entre 30 y 40 minutos
- Una hora
- Más de una hora
- No tengo ningún momento para comer

19. ¿Con cuántos minutos cuentas al día para cenar? *

Marca solo un óvalo.

- 5 minutos o menos
- Entre 10 y 20 minutos
- Entre 20 y 30 minutos
- Entre 30 y 40 minutos
- Una hora
- Más de una hora
- No tengo ningún momento para cenar

20. ¿Con cuántos minutos cuentas al día para consumir colaciones? *

Marca solo un óvalo.

- 5 minutos o menos
- Entre 10 y 20 minutos
- Entre 20 y 30 minutos
- Entre 30 y 40 minutos
- Una hora
- Más de una hora
- No tengo ningún momento para consumir colaciones

21. ¿Hay lugares de venta de alimentos frescos (frutas, verduras) cerca de tu hogar? *

Marca solo un óvalo.

- Si
- No
- No lo sé

22. ¿Hay lugares de venta de alimentos no perecederos cerca de tu hogar (frijol, avena, arroz, galletas, etc? *

Marca solo un óvalo.

- Si
- No
- No lo sé

Motivación

Ahora queremos saber que tan interesado estas en mejorar tu estilo de vida y cuidar la salud del planeta a la par. Para ello, te pedimos que respondas a las siguientes preguntas de la manera más honesta posible. Recuerda que nunca serás juzgado por tus respuestas.

23. Del 1 al 5 ¿Que tanto te gustaría mejorar tus hábitos alimentarios? *

Marca solo un óvalo.

	1	2	3	4	5	
No me gustaría						Me gustaría mucho

24. Del 1 al 5 ¿Que tanto te gustaría comer más saludable? *

Marca solo un óvalo.

	1	2	3	4	5	
No me gustaría						Me gustaría mucho

25. Del 1 al 5 ¿Que tanto te gustaría ser una persona más activa físicamente? *

Marca solo un óvalo.

	1	2	3	4	5	
No me gustaría	⬭	⬭	⬭	⬭	⬭	Me gustaría mucho

26. ¿Comes más alimentos cuando estás triste, enojado/a/e o feliz? *

Marca solo un óvalo.

- Sí
- No
- No lo sé
- Otro: ____________

27. ¿Comes menos alimentos cuando estás triste, enojado/a/e o feliz? *

Marca solo un óvalo.

- Sí
- No
- No lo sé
- Otro: ____________

28. ¿Qué sentimientos aumentan tu consumo de alimentos? *

Selecciona todos los que correspondan.

- [ ] Tristeza
- [ ] Enojo
- [ ] Felicidad
- [ ] Otro: ____________________

29. ¿Qué sentimientos disminuyen tu consumo de alimentos? *

Selecciona todos los que correspondan.

- [ ] Tristeza
- [ ] Enojo
- [ ] Felicidad
- [ ] Otro: ____________________

30. ¿Que tipo de alimentos son los que consumes más cuando experimentas esos sentimientos? *

Marca solo un óvalo.

- ( ) Dulces (pan, galletas, paletas, helado)
- ( ) Salados (papas fritas, hamburguesas, cacahuates)
- ( ) Cualquier alimento sin importar su sabor
- ( ) Otro: ____________________

31. ¿Que tipo de alimentos son los que consumes menos cuando experimentas esos sentimientos? *

Marca solo un óvalo.

- Dulces (pan, galletas, paletas, helado)
- Salados (papas fritas, hamburguesas, cacahuates)
- Cualquier alimento sin importar su sabor
- Otro: ______________________

32. Escriba por favor los hábitos que estarías dispuesto/a/e a modificar, por ejemplo, comer más frutas y verduras, comer menos carne de res, hacer más ejercicio, etc. *

____
____
____
____
____

Motivos de consumo sostenible de alimentos

Por favor escribe que tan importante son para ti los siguientes aspectos cuando vas a consumir alimentos. 1 es poco importante y 5 muy importante

33. Se produce sin que los animales sufran *

Marca solo un óvalo.

	1	2	3	4	5	
Poco importante	◯	◯	◯	◯	◯	Muy importante

34. Se produce de forma respetuosa con los animales *

Marca solo un óvalo.

	1	2	3	4	5	
Poco importante	◯	◯	◯	◯	◯	Muy importante

35. Es un producto de corral o campo libre *

Marca solo un óvalo.

	1	2	3	4	5	
Poco importante	◯	◯	◯	◯	◯	Muy importante

36. Se produce sin explotación *

Marca solo un óvalo.

	1	2	3	4	5	
Poco importante	◯	◯	◯	◯	◯	Muy importante

37. Se produce sin trabajo infantil *

Marca solo un óvalo.

	1	2	3	4	5	
Poco importante	◯	◯	◯	◯	◯	Muy importante

38. Se negocia de forma justa *

Marca solo un óvalo.

	1	2	3	4	5	
Poco importante	( )	( )	( )	( )	( )	Muy importante

39. Se produce de forma respetuosa con el medio ambiente, sin perturbar a la naturaleza *

Marca solo un óvalo.

	1	2	3	4	5	
Poco importante	( )	( )	( )	( )	( )	Muy importante

40. Se produce con mínimas emisiones de CO2 *

Marca solo un óvalo.

	1	2	3	4	5	
Poco importante	( )	( )	( )	( )	( )	Muy importante

41. Se utiliza poca agua para su producción *

Marca solo un óvalo.

	1	2	3	4	5	
Poco importante	( )	( )	( )	( )	( )	Muy importante

42. Está envasado de forma respetuosa con el medio ambiente *

Marca solo un óvalo.

	1	2	3	4	5	
Poco importante	⬭	⬭	⬭	⬭	⬭	Muy importante

43. Es un producto local / regional *

Marca solo un óvalo.

	1	2	3	4	5	
Poco importante	⬭	⬭	⬭	⬭	⬭	Muy importante

44. Es un producto de temporada *

Marca solo un óvalo.

	1	2	3	4	5	
Poco importante	⬭	⬭	⬭	⬭	⬭	Muy importante

45. Viene de cerca (poca distancia de transporte) *

Marca solo un óvalo.

	1	2	3	4	5	
Poco importante	⬭	⬭	⬭	⬭	⬭	Muy importante

Este contenido no ha sido creado ni aprobado por Google.

Google Formularios
