# Supplementary material for: Sustainable-psycho-nutritional intervention programme for a sustainable diet (the ‘NutriSOS’ study) and its effects on eating behaviour, diet quality, nutritional status, physical activity, metabolic biomarkers, gut microbiota and water and carbon footprints in Mexican population: study protocol of an mHealth randomised controlled trial
Source: Br J Nutr. 2023 Mar 30;130(10):1823–38. doi: 10.1017/S0007114523000843 (PMC10587386; doi:10.1017/S0007114523000843)
Supplement: Supplementary file 1 [file S0007114523000843sup.zip › S0007114523000843sup002.zip!/Online Supplementary material 10. Adherence questionnaire English.pdf]

NutriSó Adherence Questionnaire

Thank you very much for coming this far :) The NutriSó intervention is about to end, but first, we want to ask you a few questions. This is not a test, so please answer as honestly as possible.

*Obligatorio

1. Correo *

2. Last name *

3. Mother's last name *

4. Name *

5. If you remember it, put your participant number please

Frequency of food consumption and physical activity

Here are some questions about your food consumption and physical activity during the last 2 months. Please answer as honestly as possible.

6. During the last 2 months, how often have you eaten the following food groups? *
Foods in parentheses are examples. Please include all the food you eat. If you eat several within a group, add them please. For example, if you eat Mexican-style eggs on Monday and pozole on Sunday, you consume Mexican foods and dishes twice a week.

Marca solo un óvalo por fila.

	Never or almost never	1 to 3 times a month	1 time per week	2 to 4 times a week	5 to 6 times a week	Once a day	2 to 3 times a day	4 to 6 times a day
**Mexican food and dishes (chilaquiles, Mexican style egg, menudo, quesadillas, tacos, sopes, tortas, tamles, flutes, toast, rice pudding, capitorada, tejuino)**	◯	◯	◯	◯	◯	◯	◯	◯
**Fruits (criolla plum, capulín, nance, papaya, guava, prickly pear, pitaya, soursop, pineapple, apple)**	◯	◯	◯	◯	◯	◯	◯	◯
**Vegetables (tomato, zucchini,**	◯	◯	◯	◯	◯	◯	◯	◯

squash, onion, green tomato, mushrooms, huitlacoche, chayote, jicama)								
Whole grains and tubers (corn tortilla, amaranth, sweet potato, rice, pasta, whole wheat bread)	◯	◯	◯	◯	◯	◯	◯	◯
Legumes (beans, lentils, chickpeas)	◯	◯	◯	◯	◯	◯	◯	◯

7. During the last 2 months, how often have you eaten the following food groups? *
Foods in parentheses are examples. Please include all the food you eat. If you eat several within a group, add them please. For example, if you eat Mexican-style eggs on Monday and pozole on Sunday, you consume Mexican foods and dishes twice a week.

Marca solo un óvalo por fila.

	Never or almost never	1 to 3 times a month	1 time per week	2 to 4 times a week	5 to 6 times a week	Once a day	2 to 3 times a day	4 to 6 times a day	M[illegible] tha[illegible] tir[illegible] a [illegible]
**Seeds and healthy fats (avocado, olive oil, chia, peanuts, pumpkin seed)**	⬭	⬭	⬭	⬭	⬭	⬭	⬭	⬭	⬭
**Dairy products (milk, yogurt, cottage cheese, fresh cheese, panela**	⬭	⬭	⬭	⬭	⬭	⬭	⬭	⬭	⬭
**Eggs (whole chicken, egg whites)**	⬭	⬭	⬭	⬭	⬭	⬭	⬭	⬭	⬭
**Fish and shellfish (tilapia, leguado, octopus,**	⬭	⬭	⬭	⬭	⬭	⬭	⬭	⬭	⬭

river prawn)									
Chicken (in fillet, in pieces)									

8. During the last 2 months, how often have you eaten the following food groups? *
Foods in parentheses are examples. Please include all the food you eat. If you eat several within a group, add them please. For example, if you eat Mexican-style eggs on Monday and pozole on Sunday, you consume Mexican foods and dishes twice a week.

Marca solo un óvalo por fila.

	Never or almost never	1 to 3 times a month	1 time per week	2 to 4 times a week	5 to 6 times a week	Once a day	2 to 3 times a day	4 to 6 times a day	t
**Red and processed meats (beef, pork, sausage, ham)**									
**Ultra-processed foods (soda, cookies, box cereal, instant soups, cupcakes, chips, snack chips)**									
**Added sugar and foods high in trans and saturated fats (table sugar, fresh sweetened water, honey, jams,**									

vegetable shortening, margarine, butter)								
Alcoholic beverages (beer, tequila, red wine, pulque)								
Insects (crickets, worms)								

9. On average, what portion of foods, dishes or drinks do you usually consume each time you consume them PER DAY? For example, if you eat 3 tortillas at breakfast, lunch and dinner, you eat 9 pieces per day *

Selecciona todos los que correspondan.

	0	1/4 piece, cup, or portion	1/2 piece, cup, or portion	1 piece, cup, tablespoon or portion	2 pieces, cups, tablespoons or portions	3 pieces, cups, tablespoons or portions	ta o
**Tacos, sopes, enchiladas, flautas**	☐	☐	☐	☐	☐	☐	
**Tamales, tortas, tostadas**	☐	☐	☐	☐	☐	☐	
**Pozole, menudo, mole**	☐	☐	☐	☐	☐	☐	
**Fruits in pieces (apple, orange)**	☐	☐	☐	☐	☐	☐	
**Chopped fruit or small pieces (melon, papaya, grapes)**	☐	☐	☐	☐	☐	☐	

10. On average, what portion of foods, dishes or drinks do you usually consume each time you consume them PER DAY? For example, if you eat 3 tortillas at breakfast, lunch and dinner, you eat 9 pieces per day *

Selecciona todos los que correspondan.

	0	1/4 piece, cup, or portion	1/2 piece, cup, or portion	1 piece, cup, tablespoon or portion	2 pieces, cups, tablespoons or portions	3 pieces, cups, tablespoons or portions	ta o
**Vegetables in pieces (tomato, cucumber)**	☐	☐	☐	☐	☐	☐	
**Vegetables in cups (lettuce, spinach)**	☐	☐	☐	☐	☐	☐	
**Cereals in pieces (corn tortillas, box bread)**	☐	☐	☐	☐	☐	☐	
**Cereals in cups (rice, pasta)**	☐	☐	☐	☐	☐	☐	
**Legumes (beans, lentils)**	☐	☐	☐	☐	☐	☐	

11. On average, what portion of foods, dishes or drinks do you usually consume each time you consume them PER DAY? For example, if you eat 3 tortillas at breakfast, lunch and dinner, you eat 9 pieces per day *

Selecciona todos los que correspondan.

	0	1/4 piece, cup, or portion	1/2 piece, cup, or portion	1 piece, cup, tablespoon or portion	2 pieces, cups, tablespoons or portions	3 pieces, cups, tablespoons or portions	4 [illegible] tabl[illegible] or [illegible]
**Milk and yoghurt**	☐	☐	☐	☐	☐	☐	
**Cheeses**	☐	☐	☐	☐	☐	☐	
**Nuts and peanuts**	☐	☐	☐	☐	☐	☐	
**Chia and pumpkin seeds**	☐	☐	☐	☐	☐	☐	
**Egg and egg whites**	☐	☐	☐	☐	☐	☐	

12. On average, what portion of foods, dishes or drinks do you usually consume each time you consume them PER DAY? For example, if you eat 3 tortillas at breakfast, lunch and dinner, you eat 9 pieces per day *

Selecciona todos los que correspondan.

	0	1/4 piece, cup, or portion	1/2 piece, cup, or portion	1 piece, cup, tablespoon or portion	2 pieces, cups, tablespoons or portions	3 pieces, cups, tablespoons or portions	4 p[illegible] c[illegible] table[illegible] or p[illegible]
**Fish (80 g palm-sized portion)**	☐	☐	☐	☐	☐	☐	
**Seafood (shrimp, octopus)**	☐	☐	☐	☐	☐	☐	
**Chicken (piece or portion of 80 g of the size of the palm of the hand)**	☐	☐	☐	☐	☐	☐	
**Beef or pork (palm-sized 80g portion)**	☐	☐	☐	☐	☐	☐	
**Sweet bread (concha)**	☐	☐	☐	☐	☐	☐	

13. On average, what portion of foods, dishes or drinks do you usually consume each time you consume them PER DAY? For example, if you eat 3 tortillas at breakfast, lunch and dinner, you eat 9 pieces per day *

Selecciona todos los que correspondan.

	0	1/4 piece, cup, or portion	1/2 piece, cup, or portion	1 piece, cup, tablespoon or portion	2 pieces, cups, tablespoons or portions	3 pieces, cups, tablespoons or portions	4 [illegible] [illegible] tabl[illegible] or [illegible]
**Sugar, jams, sweets**	☐	☐	☐	☐	☐	☐	
**Soft drinks, fruit juices**	☐	☐	☐	☐	☐	☐	
**Alcoholic drinks**	☐	☐	☐	☐	☐	☐	
**Natural water**	☐	☐	☐	☐	☐	☐	

14. During the last 2 months, how many days a week did you do physical activity? (for example, walking, running, swimming, weights) *

Marca solo un óvalo.

- 0
- 1
- 2
- 3
- 4
- 5
- 6
- 7

15. During the last 2 months, what type of physical activity did you practice? *

Selecciona todos los que correspondan.

- [ ] To walk
- [ ] Jogging
- [ ] Run
- [ ] Aerobics, Zumba or dance
- [ ] Cycling
- [ ] Swimming
- [ ] Crossfit
- [ ] Weight lifting
- [ ] Multifunctional
- [ ] Calisthenics
- [ ] Focused exercises (write which ones, e.g. sit-ups, sit-ups, push-ups)
- [ ] I do not do physical activity
- [ ] Otro: ____________________

16. During the last 2 months, how many minutes of physical activity did you do per day, each time you did it? *

Marca solo un óvalo.

- 0
- 10
- 20
- 30
- 40
- 50
- 60
- 90
- 120
- 150
- 180
- 210
- 240 or more
- Otro: ____________________

17. At what intensity do you do it? *

Marca solo un óvalo.

- Mild (no labored breathing)
- Moderate (shakes breath)
- Intense (shakes your breath a lot)
- Otro: ____________________

Reasons for food consumption

Now, we want to know a little bit about the reasons why you chose to eat certain foods, or the reasons why you avoided eating others. Please answer as honestly as possible.

18. During the last 2 months, did you ever prefer to eat a particular food because of its environmental impact? *

Marca solo un óvalo.

- Yes
- No
- Otro: ______

19. If you answered yes, mention what food or foods, please *

______
______
______
______
______

20. During the last 2 months, did you ever avoid eating a particular food because of its environmental impact? *

Marca solo un óvalo.

- Yes
- No
- Otro: ______

21. If you answered yes, mention what food or foods, please *

22. Did you ever prefer to eat a particular food because of its impact on health and nutrition? *

Marca solo un óvalo.

- Yes
- No
- Otro: __________

23. If you answered yes, mention what food or foods, please *

24. During the last 2 months, did you ever avoid eating a particular food because of its impact on health and nutrition? *

Marca solo un óvalo.

- Yes
- No
- Otro: __________

25. If you answered yes, mention what food or foods, please *

26. During the last 2 months, on any occasion did you prefer to eat a particular food because of its role in the food culture of Mexico? *

Marca solo un óvalo.

- Yes
- No
- Otro: ______________

27. If you answered yes, mention what food or foods, please *

28. During the last 2 months, did you ever avoid eating a particular food because of its role in the food culture of Mexico? *

Marca solo un óvalo.

- Yes
- No
- Otro: ______________

29. If you answered yes, mention what food or foods, please *

30. During the last 2 months, did you ever prefer to eat a particular food because of its price? (for example, if it was very cheap) *

Marca solo un óvalo.

- Yes
- No
- Otro: ____________

31. If you answered yes, mention what food or foods, please *

32. During the last 2 months, did you ever avoid eating a particular food because of its price? (for example, if it is expensive) *

Marca solo un óvalo.

- Yes
- No
- Otro: ____________

33. If you answered yes, mention what food or foods, please *

Adequacy of the eating plan and physical activity

On this occasion, we want to know what you thought of the food plan that we prepared for you. Please answer honestly.

34. During the last 2 months, there was any food that you did not like and therefore did not consume it? *

Marca solo un óvalo.

- Yes
- No
- Otro: __________

35. If you answered yes, mention what food or foods, please *

36. From 1 to 5, how much would you say you followed the meal plan that was given to you? *

Marca solo un óvalo.

	1	2	3	4	5	
I didn't follow it at all	⬭	⬭	⬭	⬭	⬭	I fully followed

37. From 1 to 5, how satiated did you feel during the last 7 weeks? *

Marca solo un óvalo.

	1	2	3	4	5	
Very hungry	⬭	⬭	⬭	⬭	⬭	Very satiated

38. From 1 to 5, how much would you say you liked the meal plan? *

Marca solo un óvalo.

	1	2	3	4	5	
I did not like anything	⬭	⬭	⬭	⬭	⬭	I loved it

39. From 1 to 5, how did you feel during these last 7 weeks, regarding your energy levels? *

Marca solo un óvalo.

	1	2	3	4	5	
Very bad	⬭	⬭	⬭	⬭	⬭	Very well

40. From 1 to 5, during the last 7 weeks, did you experience anxiety about eating any particular food? *

Marca solo un óvalo.

	1	2	3	4	5	
No anxiety	( )	( )	( )	( )	( )	A lot of anxiety

41. If the answer is yes, indicate what food or foods caused you anxiety *

Other habits

Here are some questions about other habits. Please answer honestly.

42. During the last 7 weeks, who cooked the food you ate? *

Selecciona todos los que correspondan.

- [ ] Me
- [ ] Some relative (mom, dad, aunt, uncle, grandmother, grandfather)
- [ ] Economic Kitchen
- [ ] Restaurants
- [ ] Somebody give it to me
- [ ] Home helper or cook
- [ ] Otro: ____________

43. During the last 7 weeks, where did you buy your groceries most often? *

Marca solo un óvalo.

- Tianguis
- Market
- Retail supermercado (Soriana, Waltmart)
- Wholesale supermarket (Sam's, Costco)
- Butcher shop
- Creamery
- Fruit store
- Otro: ______________________

Este contenido no ha sido creado ni aprobado por Google.

Google Formularios
