# Supplementary material for: Sustainable-psycho-nutritional intervention programme for a sustainable diet (the ‘NutriSOS’ study) and its effects on eating behaviour, diet quality, nutritional status, physical activity, metabolic biomarkers, gut microbiota and water and carbon footprints in Mexican population: study protocol of an mHealth randomised controlled trial
Source: Br J Nutr. 2023 Mar 30;130(10):1823–38. doi: 10.1017/S0007114523000843 (PMC10587386; doi:10.1017/S0007114523000843)
Supplement: Supplementary file 1 [file S0007114523000843sup.zip › S0007114523000843sup002.zip!/Online Supplementary material 11. Adherence questionnaire Spanish.pdf]

Cuestionario de Adherencia NutriSó

Muchas gracias por haber llegar hasta aquí :) La intervención NutriSó está por terminar, pero antes, queremos hacerte unas preguntas. Este no es un examen, así que responde de la manera más honesta posible.

*Obligatorio

1. Correo *

2. Apellido paterno *

3. Apellido materno *

4. Nombre *

5. Si lo recuerdas, coloca tu número de participante por favor

Frecuencia de consumo de alimentos y realización de actividad física

A continuación, se presentan algunas preguntas sobre tu consumo de alimentos y realización de actividad física durante estos últimos 2 meses. Responde de la manera más honesta por favor.

6. Durante los últimos 2 meses ¿con qué frecuencia has consumido los siguientes grupos de alimentos? Los alimentos entre paréntesis son ejemplos. Incluye por favor, todos los alimentos que consumas. Si comes varios dentro de un grupo, sumálos por favor. Por ejemplo, si comes huevo a la mexicana el lunes, y el domingo pozole, consumes alimentos y platillos mexicanos 2 veces por semana *

Marca solo un óvalo por fila.

	Nunca o casi nunca	1 a 3 veces al mes	1 vez a la semana	2 a 4 veces a la semana	5 a 6 veces a la semana	1 vez al día	2 a 3 veces al día	4 [illegible]
**Alimentos y platillos mexicanos (chilaquiles, huevo a la mexicana, menudo, quesadillas, tacos, sopes, tortas, tamles, flautas, tostadas, arroz con leche, capitorada, tejuino)**								
**Frutas (ciruela criolla, capulín, nance, papaya, guayaba, tuna, pitaya, guanábana, piña, manzana)**								
**Vegetales (jitomate, calabacita, calabaza,**								

cebolla, tomate verde, champiñones, huitlacoche, chayote, jícama)								
Cereales integrales y tubérculos (tortilla de maíz, amaranto, camote, arroz, pasta, pan integral)								
Leguminosas (frijoles, lentejas, garbanzos)								

7. Durante los últimos 2 meses ¿con qué frecuencia has consumido los siguientes grupos de alimentos? Los alimentos entre paréntesis son ejemplos. Incluye por favor, todos los alimentos que consumas. Si comes varios dentro de un grupo, sumálos por favor. Por ejemplo, si comes huevo a la mexicana el lunes, y el domingo pozole, consumes alimentos y platillos mexicanos 2 veces por semana *

Marca solo un óvalo por fila.

	Nunca o casi nunca	1 a 3 veces al mes	1 vez a la semana	2 a 4 veces a la semana	5 a 6 veces a la semana	1 vez al día	2 a 3 veces al día	4 a 6 veces al día
**Semillas y grasas saludables (aguacate, aceite de oliva, chía, cacahuate, semilla de calabaza)**	◯	◯	◯	◯	◯	◯	◯	◯
**Lácteos (leche, yogurt, requesón, queso fresco, panela**	◯	◯	◯	◯	◯	◯	◯	◯
**Huevos (de gallina entero, claras de huevo)**	◯	◯	◯	◯	◯	◯	◯	◯
**Pescados y mariscos (tilapia, leguado, pulpo, langostino de río)**	◯	◯	◯	◯	◯	◯	◯	◯
**Pollo (en**	◯	◯	◯	◯	◯	◯	◯	◯

filete, en
piezas)

8. Durante los últimos 2 meses ¿con qué frecuencia has consumido los siguientes grupos de alimentos? Los alimentos entre paréntesis son ejemplos. Incluye por favor, todos los alimentos que consumas. Si comes varios dentro de un grupo, sumálos por favor. Por ejemplo, si comes huevo a la mexicana el lunes, y el domingo pozole, consumes alimentos y platillos mexicanos 2 veces por semana *

Marca solo un óvalo por fila.

	Nunca o casi nunca	1 a 3 veces al mes	1 vez a la semana	2 a 4 veces a la semana	5 a 6 veces a la semana	1 vez al día	2 a 3 veces al día	4 a vec al c
**Carnes rojas y procesadas (carne de res, cerdo, salchichas, jamón)**	◯	◯	◯	◯	◯	◯	◯	◯
**Alimentos ultra-procesados (refresco, galletas, cereal de caja, sopas instantáneas, pastelitos, papas fritas, botanas fritas)**	◯	◯	◯	◯	◯	◯	◯	◯
**Azúcar añadido y alimentos altos en grasas trans y saturadas (azúcar de mesa, agua fresca endulzada, miel, mermeladas, manteca**	◯	◯	◯	◯	◯	◯	◯	◯

vegetal, margarina, mantequilla)								
Bebidas alcoholicas (cerveza, tequila, vino tinto, pulque)								
Insectos (grillos, gusanos)								

9. En promedio, ¿qué porción de alimentos, platillos o bebidas sueles consumir cada vez que los consumes POR DÍA? Por ejemplo, si comes 3 tortillas en el desayuno, comida y cena, comes 9 piezas por día *

Marca solo un óvalo por fila.

	0	1/4 pieza, taza, o porción	1/2 pieza, taza, o porción	1 pieza, taza, cucharada o porción	2 piezas, tazas, cucharadas o porciones	3 piezas, tazas, cucharadas o porciones	4 [illegible]
**Tacos, sopes, enchiladas, flautas**							
**Tamales, tortas, tostadas**							
**Pozole, menudo, mole**							
**Frutas en piezas (manzana, naranja)**							
**Fruta picada o piezas pequeñas (melón, papaya, uvas)**							

10. En promedio, ¿qué porción de alimentos, platillos o bebidas sueles consumir cada vez que los consumes POR DÍA? Por ejemplo, si comes 3 tortillas en el desayuno, comida y cena, comes 9 piezas por día *

Marca solo un óvalo por fila.

	0	1/4 pieza, taza, o porción	1/2 pieza, taza, o porción	1 pieza, taza, cucharada o porción	2 piezas, tazas, cucharadas o porciones	3 piezas, tazas, cucharadas o porciones	[illegible]
**Verduras en piezas (jitomate, pepino)**							
**Verduras en tazas (lechuga, espinacas)**							
**Cereales en piezas (tortillas de maíz, pan de caja)**							
**Cereales en tazas (arroz, pasta)**							
**Leguminosas (frijoles, lentejas)**							

11. En promedio, ¿qué porción de alimentos, platillos o bebidas sueles consumir cada vez que los consumes POR DÍA? Por ejemplo, si comes 3 tortillas en el desayuno, comida y cena, comes 9 piezas por día *

Marca solo un óvalo por fila.

	0	1/4 pieza, taza, o porción	1/2 pieza, taza, o porción	1 pieza, taza, cucharada o porción	2 piezas, tazas, cucharadas o porciones	3 piezas, tazas, cucharadas o porciones	4 [illegible]
**Leche y yourt**							
**Quesos**							
**Nueces y cacahuates**							
**Semillas de chía y calabaza**							
**Huevo y claras de huevo**							

12. En promedio, ¿qué porción de alimentos, platillos o bebidas sueles consumir cada vez que los consumes POR DÍA? Por ejemplo, si comes 3 tortillas en el desayuno, comida y cena, comes 9 piezas por día *

Marca solo un óvalo por fila.

	0	1/4 pieza, taza, o porción	1/2 pieza, taza, o porción	1 pieza, taza, cucharada o porción	2 piezas, tazas, cucharadas o porciones	3 piezas, tazas, cucharadas o porciones	4 [illegible] cu[illegible] p[illegible]
**Pescado (porción de 80 g del tamaño palma de la mano)**							
**Mariscos (camarones, pulpo)**							
**Pollo (pieza o porción de 80 g del tamaño palma de la mano)**							
**Carne de res o cerdo (porción de 80 g del tamaño palma de la mano)**							
**Pan dulce (concha)**							

13. En promedio, ¿qué porción de alimentos, platillos o bebidas sueles consumir cada vez que los consumes POR DÍA? Por ejemplo, si comes 3 tortillas en el desayuno, comida y cena, comes 9 piezas por día *

Marca solo un óvalo por fila.

	0	1/4 pieza, taza, o porción	1/2 pieza, taza, o porción	1 pieza, taza, cucharada o porción	2 piezas, tazas, cucharadas o porciones	3 piezas, tazas, cucharadas o porciones	[illegible]
**Azúcar, mermeladas, dulces**							
**Refrescos, jugos de fruta**							
**Bebidas alcohólicas**							
**Agua natural**							

14. Durante los últimos 2 meses ¿cuántos días a la semana realizas actividad física? (por ejemplo, caminar, correr, natación, pesas) *

Marca solo un óvalo.

- 0
- 1
- 2
- 3
- 4
- 5
- 6
- 7

15. Durante los últimos 2 meses ¿qué tipo de actividad física practicaste? *

Selecciona todos los que correspondan.

- [ ] Caminar
- [ ] Trotar
- [ ] Correr
- [ ] Aeróbics, zumba o baile
- [ ] Ciclismo
- [ ] Natación
- [ ] Crossfit
- [ ] Pesas
- [ ] Multifuncional
- [ ] Ejercicios focalizados (escribir cuales, por ejemplo abdominales, sentadillas, lagartijas)
- [ ] Calistenia
- [ ] No realizo acitividad física
- [ ] Otro: ______________________

16. Durante los últimos 2 meses ¿cuántos minutos de actividad física realizaste por día, cada vez que la realizaste? *

Marca solo un óvalo.

- 0
- 10
- 20
- 30
- 40
- 50
- 60
- 90
- 120
- 150
- 180
- 210
- 240 o más
- Otro: _______________

17. ¿A qué intensidad la realizas? *

Marca solo un óvalo.

- Leve (no agita la respiración)
- Moderada (agita la respiración)
- Intensa (agita mucho la respiración)
- Otro: _______________

Motivos de consumo de alimentos

Ahora, queremos saber un poco sobre los motivos por los cuales elegiste comer ciertos alimentos, o las razones por las cuales evitaste consumir otros. Responde de la manera más honesta posible por favor.

18. Durante los últimos 2 meses ¿en alguna ocasión preferiste comer algún alimento en particular por su impacto medioambiental? *

Marca solo un óvalo.

- Si
- No
- Otro: ____________

19. Si respondiste que si, menciona que alimento o alimentos, por favor *

____
____
____
____
____

20. Durante los últimos 2 meses ¿en alguna ocasión evitaste comer algún alimento en particular por su impacto medioambiental? *

Marca solo un óvalo.

- Si
- No
- Otro: ____________

21. Si respondiste que si, menciona que alimento o alimentos, por favor *

____
____
____
____
____

22. Durante los últimos 2 meses ¿en alguna ocasión preferiste comer algún alimento en particular por su impacto sobre la salud y nutrición? *

Marca solo un óvalo.

- Sí
- No
- Otro: ____________________

23. Si respondiste que si, menciona que alimento o alimentos, por favor *

24. Durante los últimos 2 meses ¿en alguna ocasión evitaste comer algún alimento en particular por su impacto sobre la salud y nutrición? *

Marca solo un óvalo.

- Sí
- No
- Otro: ____________________

25. Si respondiste que si, menciona que alimento o alimentos, por favor *

26. Durante los últimos 2 meses ¿en alguna ocasión preferiste comer algún alimento en particular por su papel en la cultura alimentaria de México? *

Marca solo un óvalo.

- Sí
- No
- Otro: ____________________

27. Si respondiste que si, menciona que alimento o alimentos, por favor *

____
____
____
____
____

28. Durante los últimos 2 meses ¿en alguna ocasión evitaste comer algún alimento en particular por su papel en la cultura alimentaria de México? *

Marca solo un óvalo.

- Sí
- No
- Otro: ____________________

29. Si respondiste que si, menciona que alimento o alimentos, por favor *

____
____
____
____
____

30. Durante los últimos 2 meses ¿en alguna ocasión preferiste comer algún alimento en particular por su precio? (por ejemplo, si estaba muy barato) *

Marca solo un óvalo.

- ( ) Sí
- ( ) No
- ( ) Otro: ____________________

31. Si respondiste que si, menciona que alimento o alimentos, por favor *

____
____
____
____
____

32. Durante los últimos 2 meses ¿en alguna ocasión evitaste comer algún alimento en particular por su precio? (por ejemplo, si está caro) *

Marca solo un óvalo.

- ( ) Sí
- ( ) No
- ( ) Otro: ____________________

33. Si respondiste que si, menciona que alimento o alimentos, por favor *

Adecuación del plan alimenticio y actividad física

En ésta ocasión, queremos saber que te pareció el plan alimenticio que elaboramos para ti. Por favor, responde honestamente.

34. Durante los últimos 2 meses ¿hubo algún alimento que no te haya gustado y por ello no lo consumiste? *

Marca solo un óvalo.

- Sí
- No
- Otro: ________

35. Si respondiste que si, menciona que alimento o alimentos, por favor *

36. Del 1 al 5, ¿qué tanto dirías que seguiste el plan alimenticio que se te brindó? *

Marca solo un óvalo.

	1	2	3	4	5	
No lo seguí en absoluto	◯	◯	◯	◯	◯	Lo seguí totalmente

37. Del 1 al 5, ¿qué tan saciado/a/e te sentiste durante estas últimas 7 semanas? *

Marca solo un óvalo.

	1	2	3	4	5	
Con mucha hambre	◯	◯	◯	◯	◯	Muy saciado

38. Del 1 al 5, ¿qué tanto dirías que te gustó el plan alimenticio? *

Marca solo un óvalo.

	1	2	3	4	5	
No me gustó nada	◯	◯	◯	◯	◯	Me gustó mucho

39. Del 1 al 5, ¿cómo te sentiste durante estás últimas 7 semanas, respecto a tus niveles de energía? *

Marca solo un óvalo.

	1	2	3	4	5	
Muy mal	◯	◯	◯	◯	◯	Muy bien

40. Del 1 al 5 ¿durante las últimas 7 semanas experiementaste ansiedad por comer algún alimento en particular? *

Marca solo un óvalo.

	1	2	3	4	5	
Sin ansiedad	◯	◯	◯	◯	◯	Mucha ansiedad

41. Si la respuesta es si, señala que alimento o alimentos te generaron ansiedad *

____
____
____
____
____

Otros hábitos

A continuación se presentan algunas preguntas sobre otros hábitos. Por favor responde honestamente.

42. Durante las últimas 7 semanas ¿quién cocinó los alimentos que consumiste? *

Selecciona todos los que correspondan.

- [ ] Yo
- [ ] Algún familiar (mamá, papá, tía, tío, abuela, abuelo)
- [ ] Cocina económica
- [ ] Restaurantes
- [ ] Me la regalan
- [ ] Ayudante del hogar o cocinera
- [ ] Otro: ______________________

43. Durante las últimas 7 semanas, ¿dónde compraste tus alimentos más frecuentemente? *

Marca solo un óvalo.

- Tianguis
- Mercado
- Supermercado menudeo (Soriana, Waltmart)
- Supermercado mayoreo (Sam’s, Costco)
- Carnicería
- Cremería
- Frutería
- Otro: ______________________

Este contenido no ha sido creado ni aprobado por Google.

Google Formularios
